# Supplementary material for: A novel LRR receptor-like kinase BRAK reciprocally phosphorylates PSKR1 to enhance growth and defense in tomato
Source: EMBO J. 2024 Oct 24;43(23):16. doi: 10.1038/s44318-024-00278-z (PMC11612273; doi:10.1038/s44318-024-00278-z)
Supplement: Supplementary file 1 — Appendix [file 44318_2024_278_MOESM1_ESM.pdf]

A novel LRR receptor-like kinase BRAK reciprocally phosphorylates PSKR1 to enhance growth and defense in tomato

Shuting Ding, Shuxian Feng, Shibo Zhou, Zhengran Zhao, Xiao Liang, Jiao Wang, Ruishuang Fu, Rui Deng, Tao Zhang, Shujun Shao, Jingquan Yu, Christine H. Foyer, Kai Shi

Appendix Contents

Appendix Figure S1 page 1

Appendix Figure S2 page 2

Appendix Figure S3 page 3

Appendix Figure S4 page 4

Appendix Figure S1

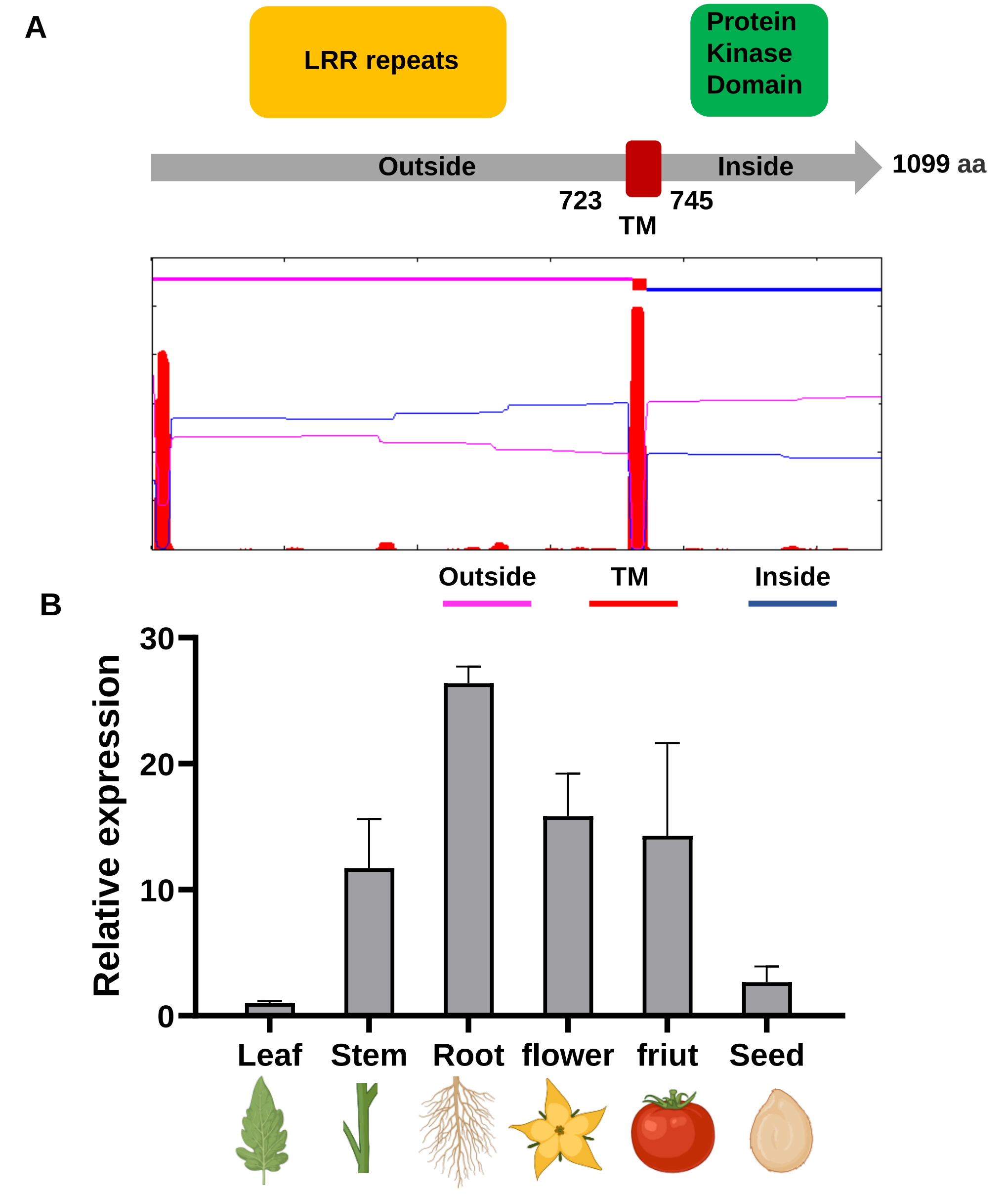

Appendix Figure S1. Key features and the expression pattern of tomato *B. cinerea* resistance-associated kinase (BRAK). (Supports Figure 1)

(A) The functional domains of BRAK were determined by Pfam and Smart. The Illustration of the putative transmembrane (TM) region of BRAK was predicted by TMHMM V2.0. (B) BRAK gene expression in tomato leaf, root, stem, flower, fruit and seed using qRT-PCR. *n* = 3 independent pooled samples with each sample being from two plants. The gene expression level in leaf was defined as 1.

Appendix Figure S2

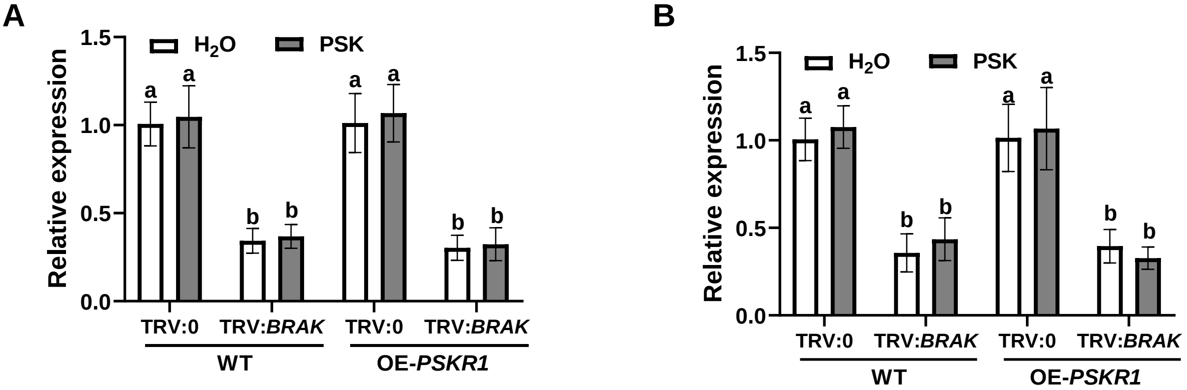

Appendix Figure S2. BRAK gene silencing efficiency in in WT and OE-*PSKR1* tomato plants. (Supports Figure 5)

(A) Gene silencing efficiency in VIGS tomato plants used in Figure 5A and B; (B) Gene silencing efficiency in VIGS tomato plants used in Figure 5C-F. The transcript abundance of *BRAK* in TRV: 0 was defined as 1.

The results in A and B are presented as mean values ± SD, *n* = 8 tomato seedings in (A), *n* = 4 independent pooled samples with each sample being from two plants in (B). Different letters indicate significant differences between treatments (*P* < 0.05, Tukey's test). These experiments were performed three times with similar results.

Appendix Figure S3

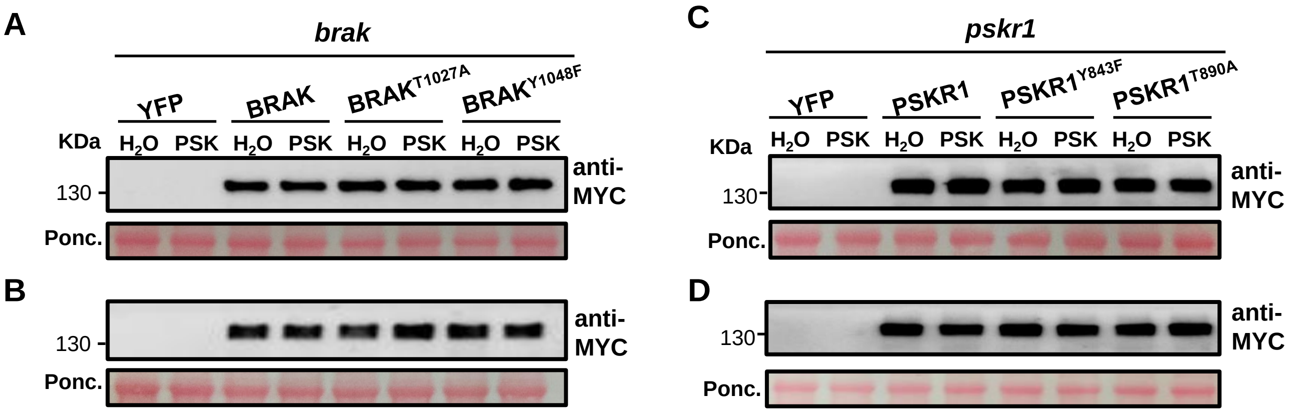

Appendix Figure S3. Identification of BRAK and PSKR1 plants. (Supports Figure 6 and 7)
(A-B) Identification of the expression of YFP or phospho-mutated versions of BRAK in the *brak* background by western blotting using an anti-MYC antibody. Ponceau S staining was used as the loading control.
(C-D) Identification of the expression of YFP or phospho-mutated versions of PSKR1 in the *pskr1* background by western blotting using an anti-MYC antibody. Ponceau S staining was used as the loading control.

Appendix Figure S4

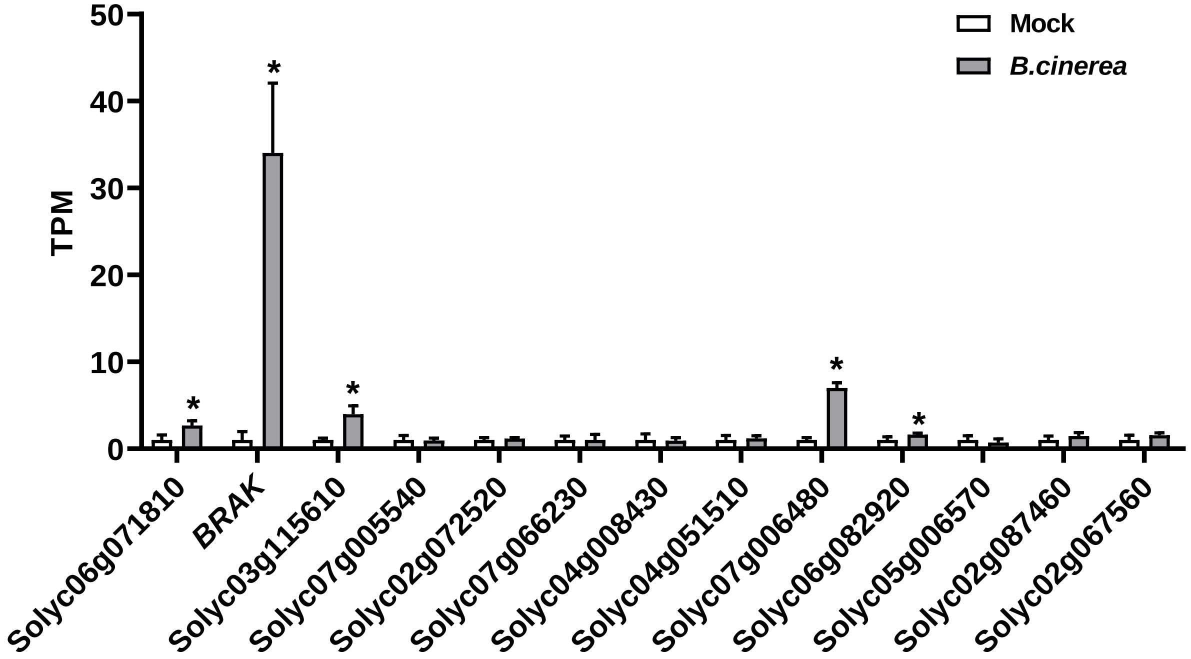

Appendix Figure S4. The transcript abundance of tomato LRR-RLK subfamily IV genes in leaves at 24 hours after *B. cinerea infection*. The transcript abundance under mock condition was defined as 1. Different letters indicate significant differences between treatments ($P < 0.05$, Tukey's test). Data are from our RNA Seq (Table EV4).
