# Supplementary figures and images for: A novel LRR receptor-like kinase BRAK reciprocally phosphorylates PSKR1 to enhance growth and defense in tomato (part 1 of 2)
Source: EMBO J. 2024 Oct 24;43(23):16. doi: 10.1038/s44318-024-00278-z (PMC11612273; doi:10.1038/s44318-024-00278-z)

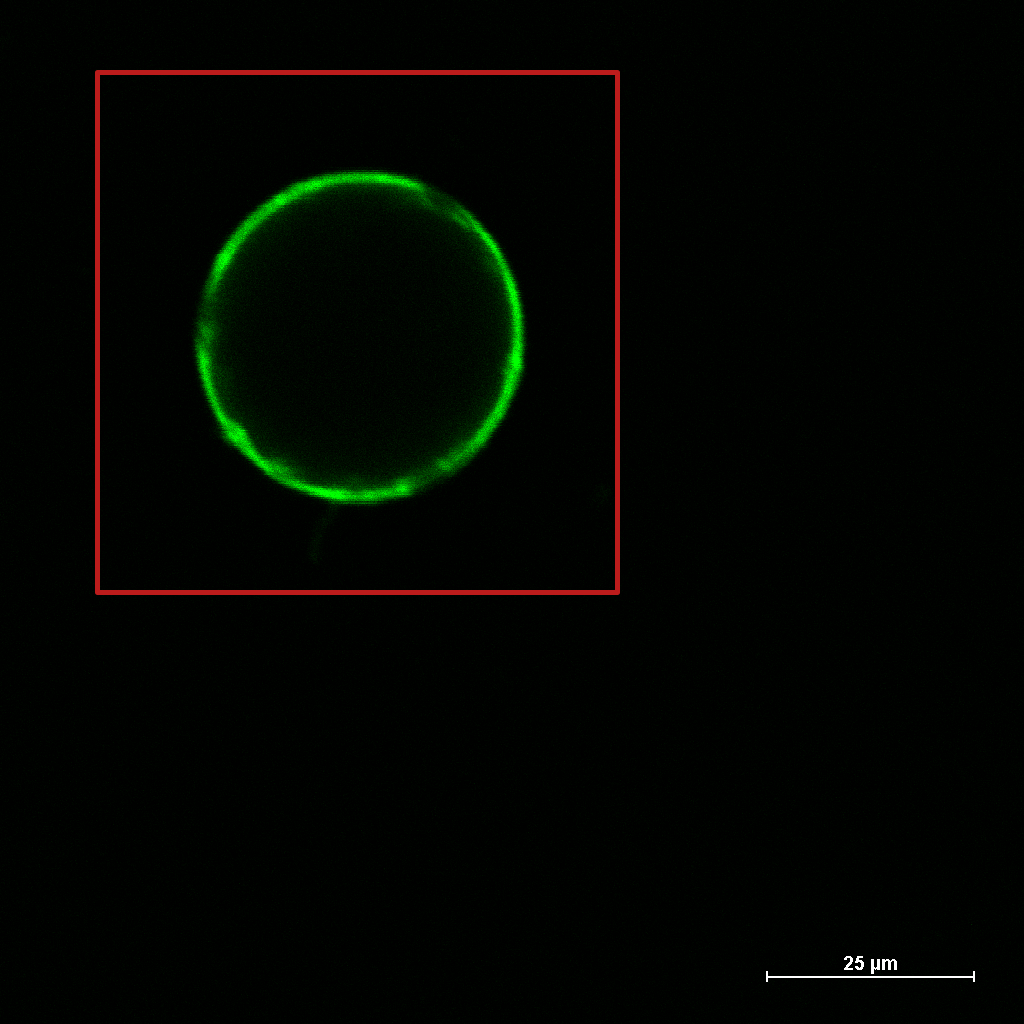

Supplement: Supplementary file 10 — Source data Fig. 1 [file 44318_2024_278_MOESM10_ESM.zip › Figure 1C/BRAK/1_BRAK_FLS2_GFP.tif]

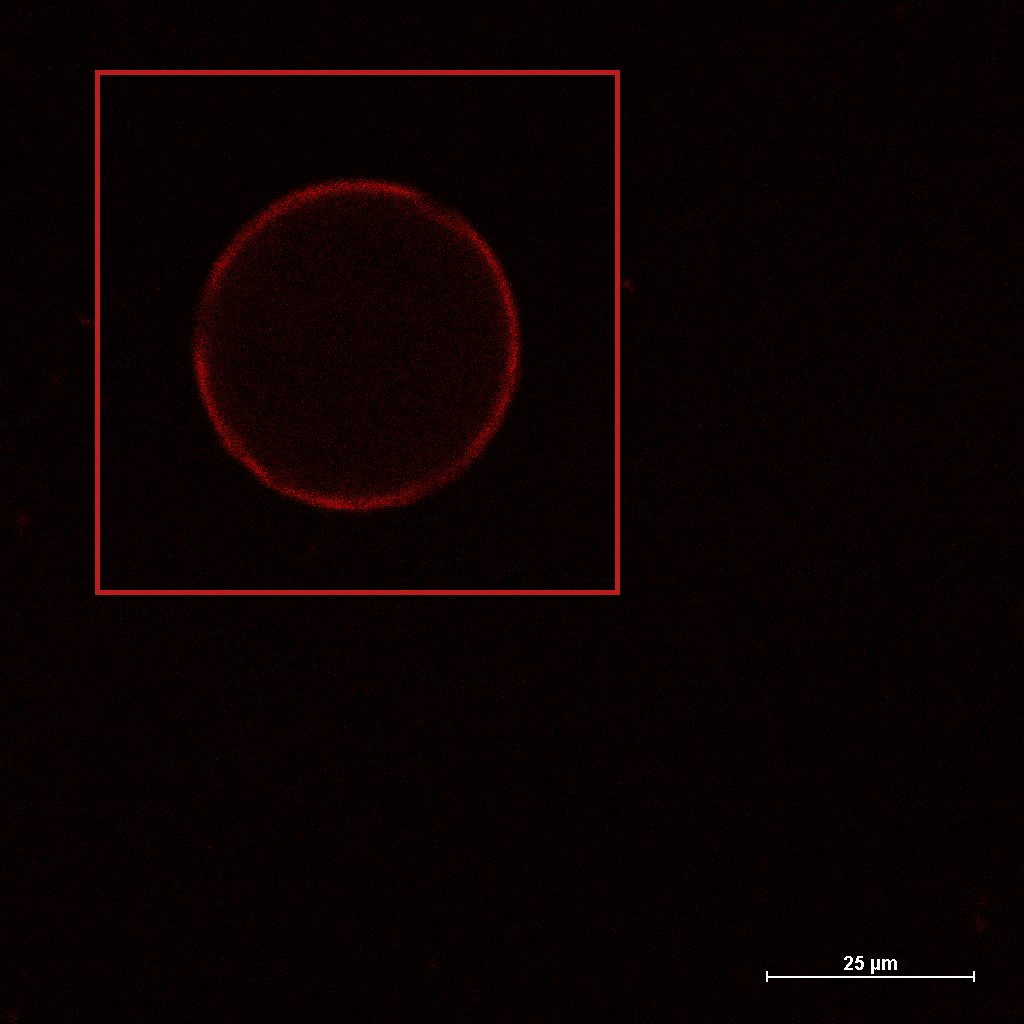

Supplement: Supplementary file 10 — Source data Fig. 1 [file 44318_2024_278_MOESM10_ESM.zip › Figure 1C/BRAK/2_BRAK_FLS2_RFP.tif]

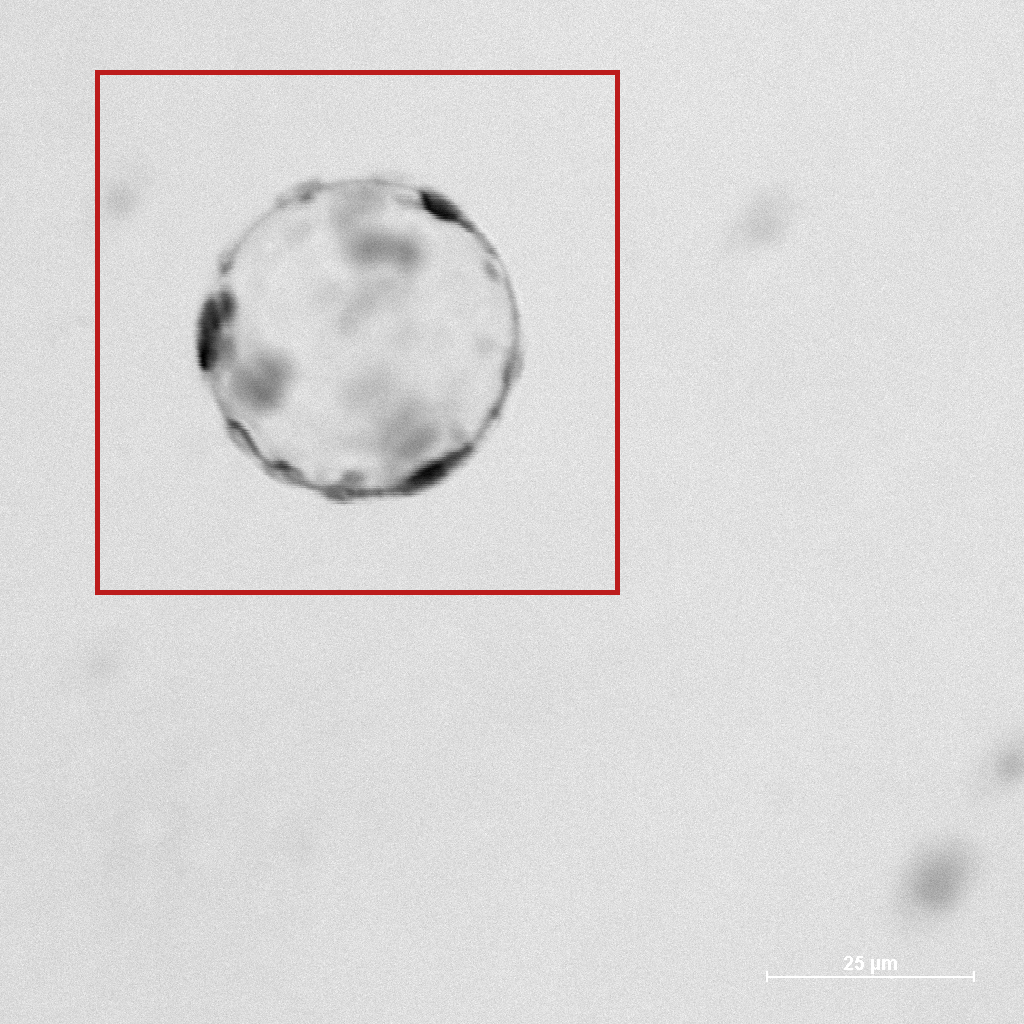

Supplement: Supplementary file 10 — Source data Fig. 1 [file 44318_2024_278_MOESM10_ESM.zip › Figure 1C/BRAK/3_BRAK_FLS2_bright.tif]

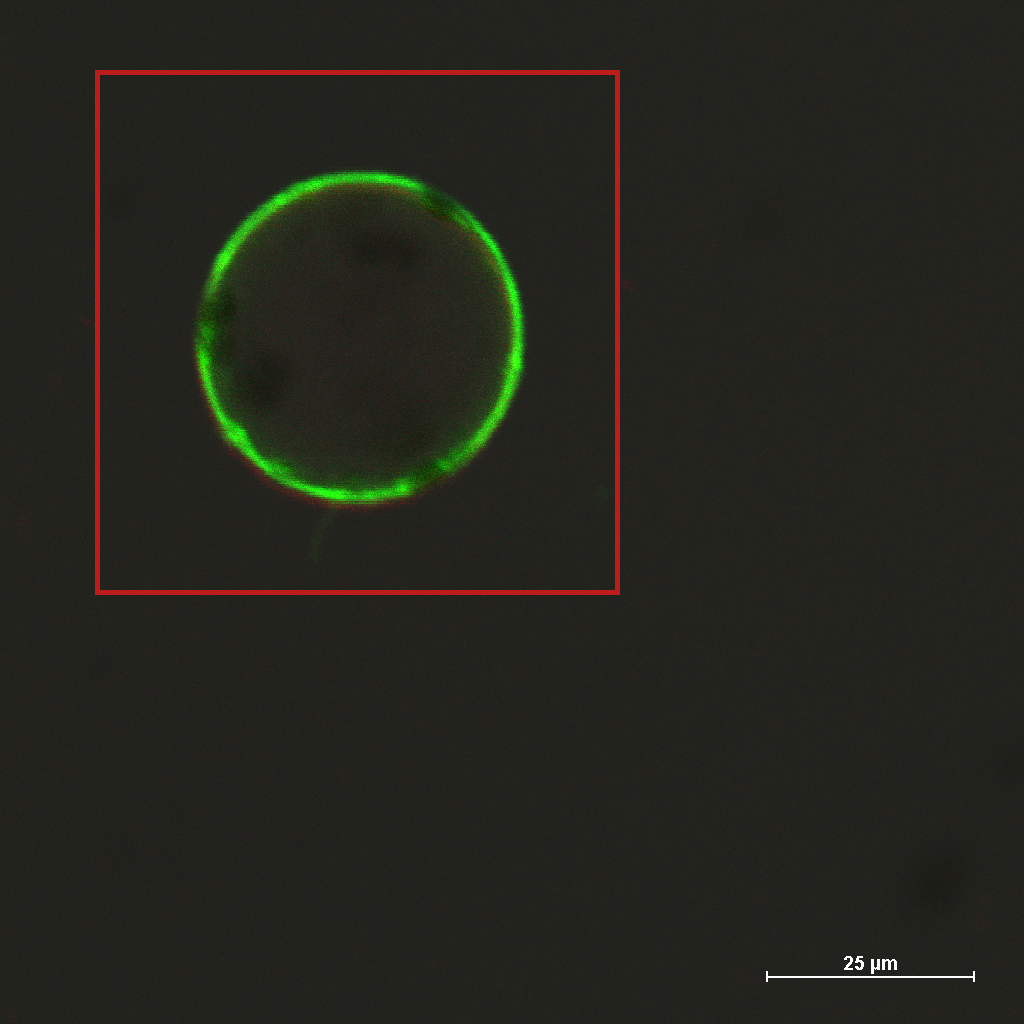

Supplement: Supplementary file 10 — Source data Fig. 1 [file 44318_2024_278_MOESM10_ESM.zip › Figure 1C/BRAK/4_BRAK_FLS2_merge.tif]

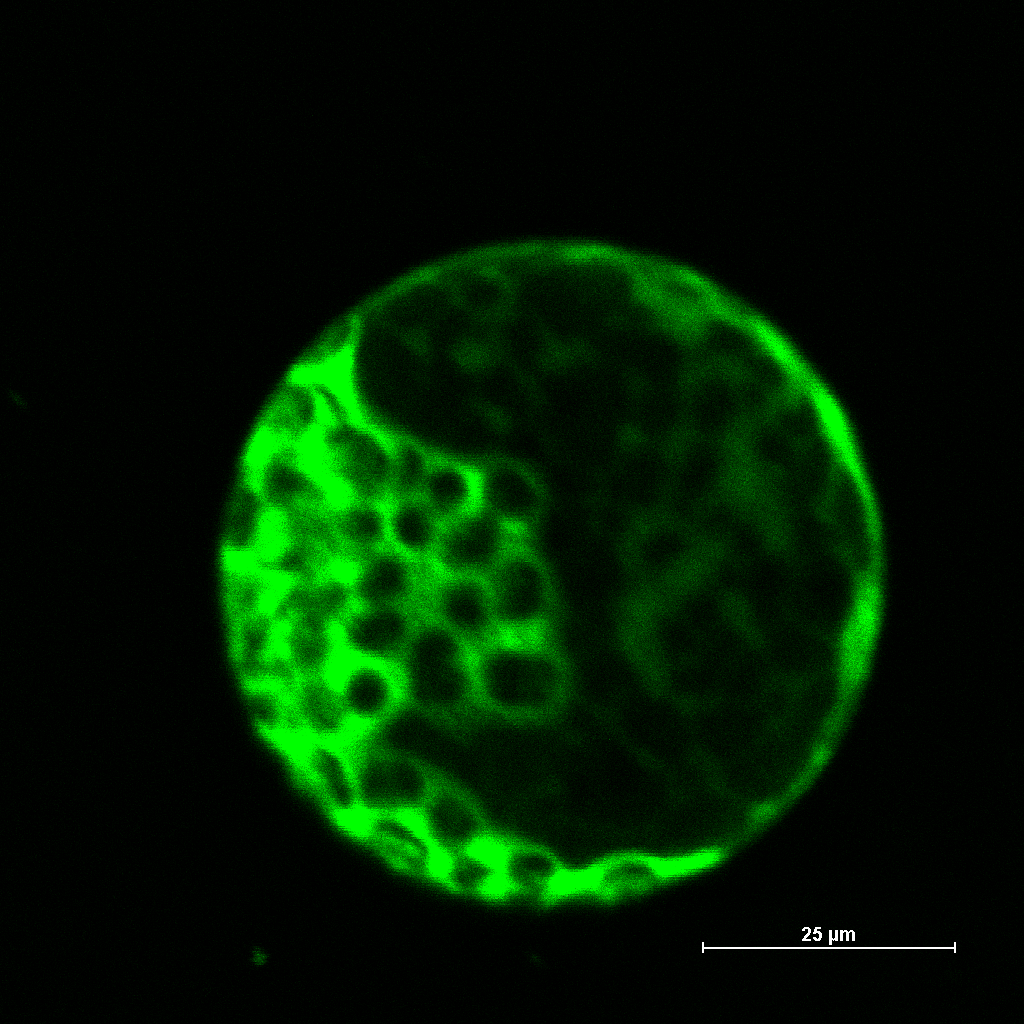

Supplement: Supplementary file 10 — Source data Fig. 1 [file 44318_2024_278_MOESM10_ESM.zip › Figure 1C/EV/1_GFP.tif]

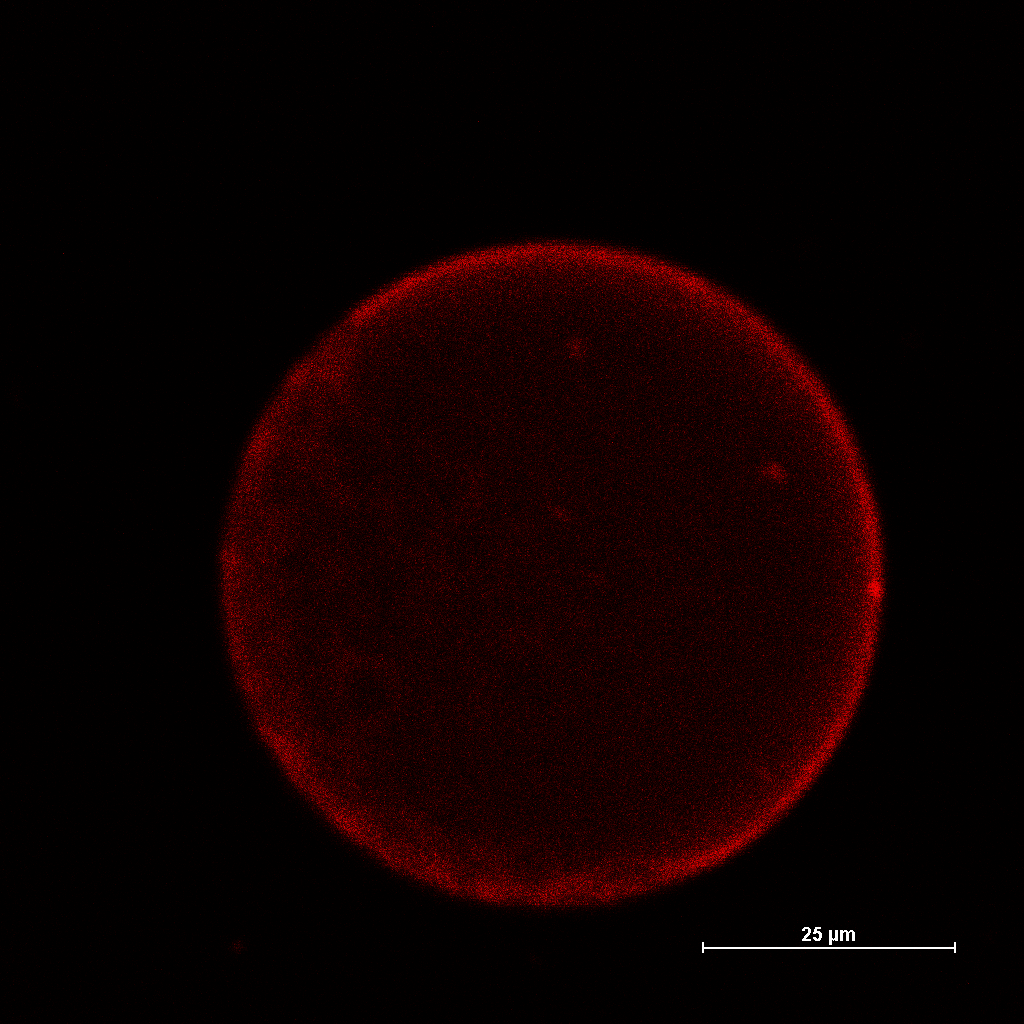

Supplement: Supplementary file 10 — Source data Fig. 1 [file 44318_2024_278_MOESM10_ESM.zip › Figure 1C/EV/2_mCherry.tif]

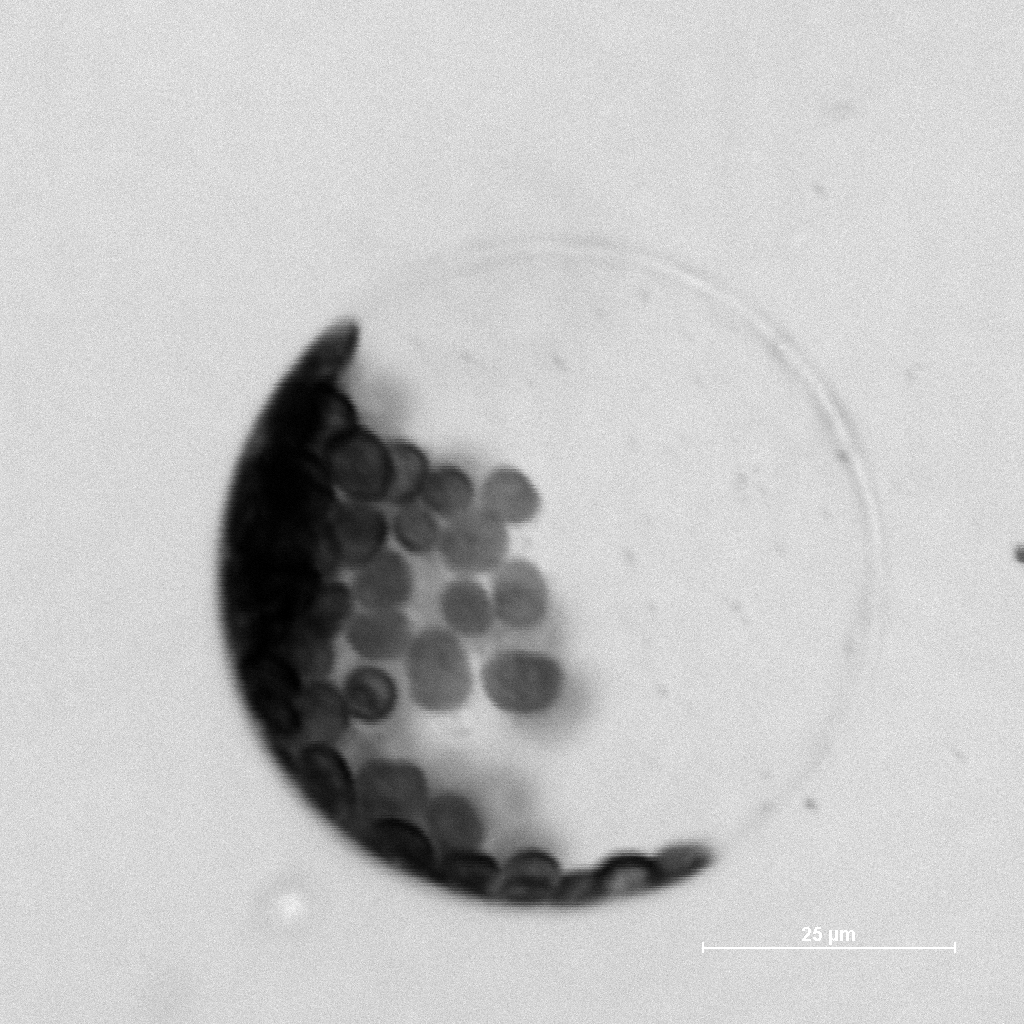

Supplement: Supplementary file 10 — Source data Fig. 1 [file 44318_2024_278_MOESM10_ESM.zip › Figure 1C/EV/3_Bright field.tif]

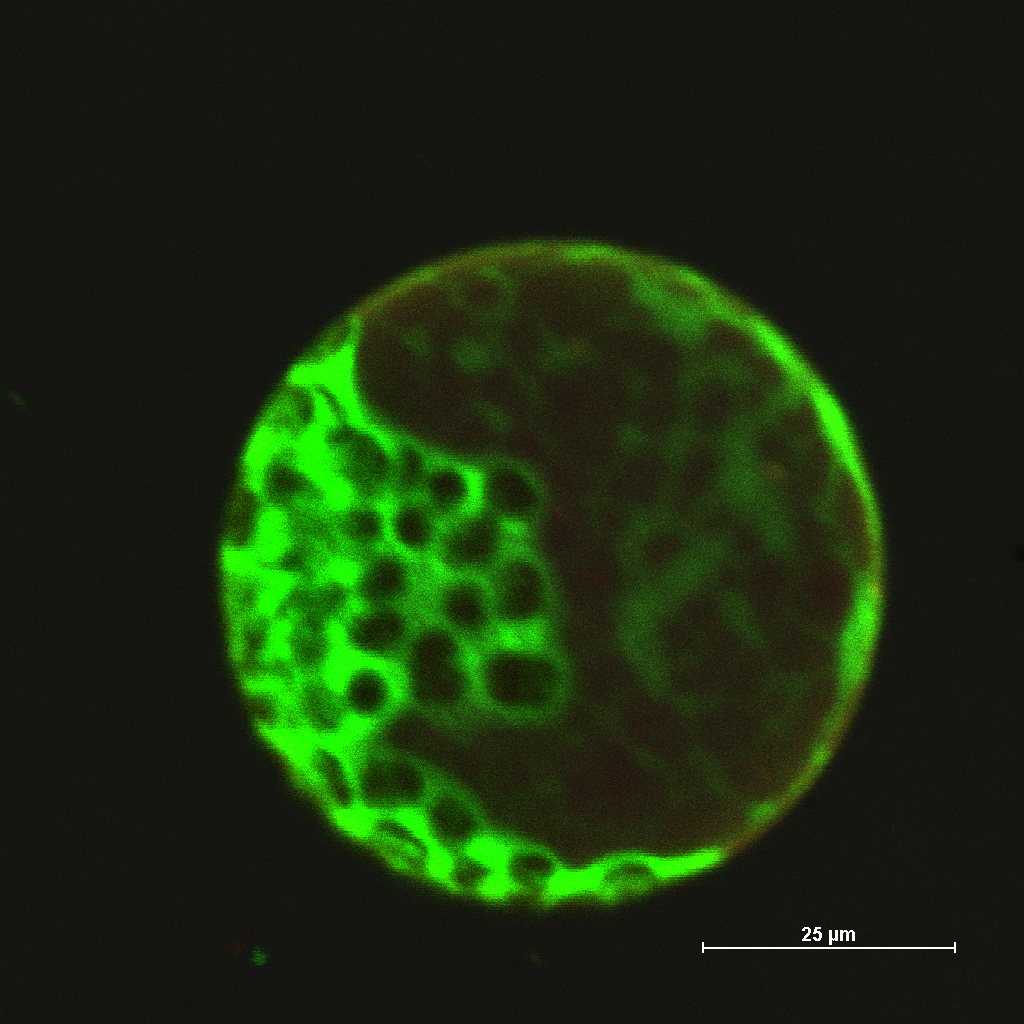

Supplement: Supplementary file 10 — Source data Fig. 1 [file 44318_2024_278_MOESM10_ESM.zip › Figure 1C/EV/4_Merge.tif]

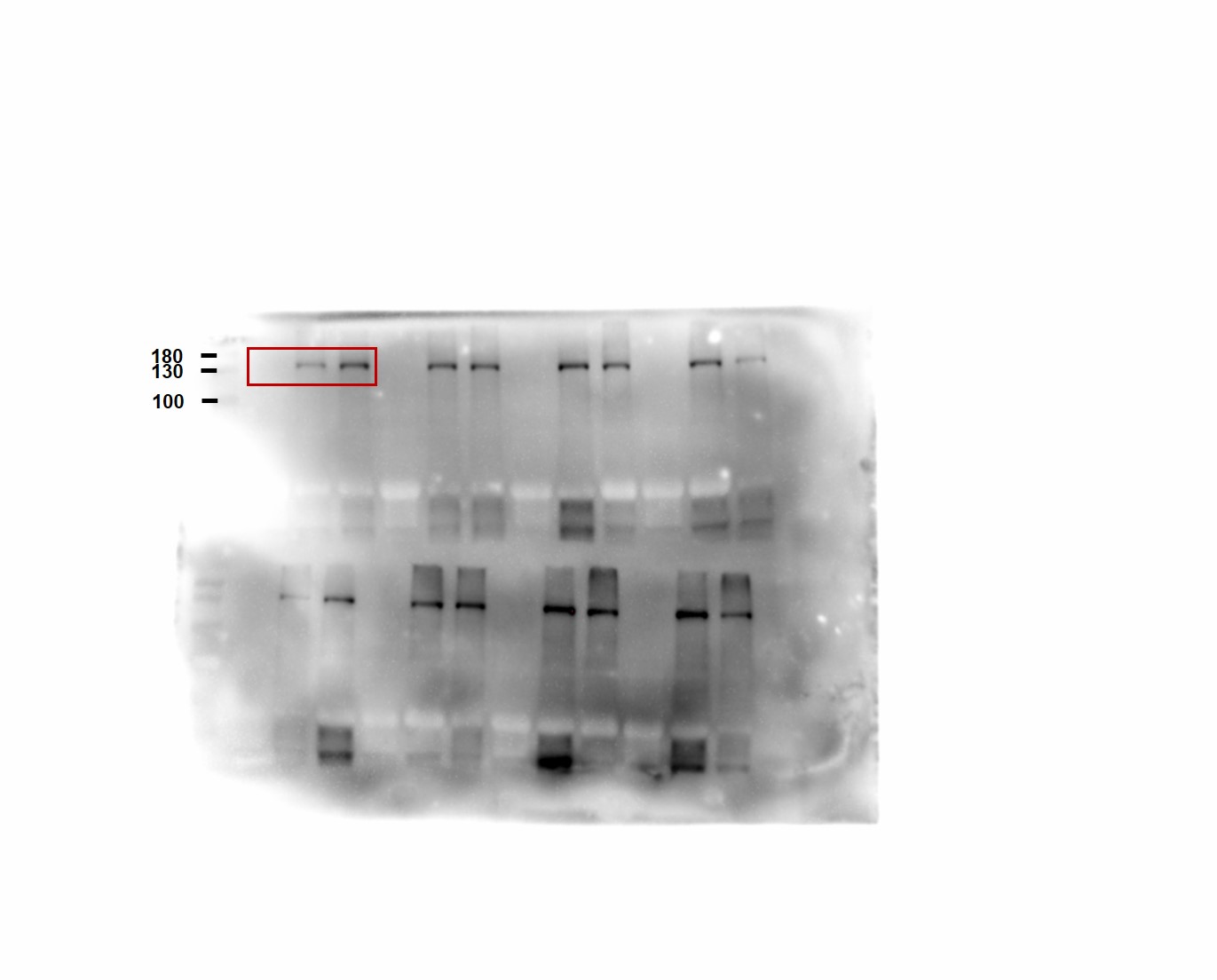

Supplement: Supplementary file 11 — Source data Fig. 2 [file 44318_2024_278_MOESM11_ESM.zip › Figure 2B/anti-HA.jpg]

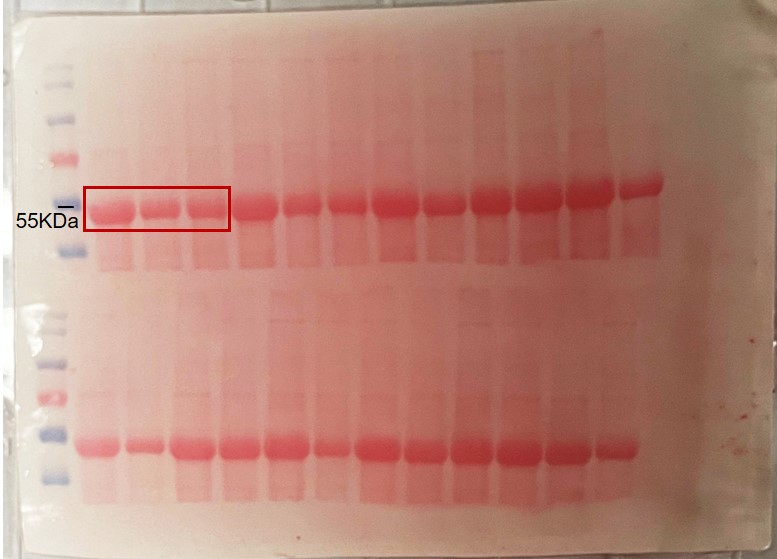

Supplement: Supplementary file 11 — Source data Fig. 2 [file 44318_2024_278_MOESM11_ESM.zip › Figure 2B/Ponc.jpg]

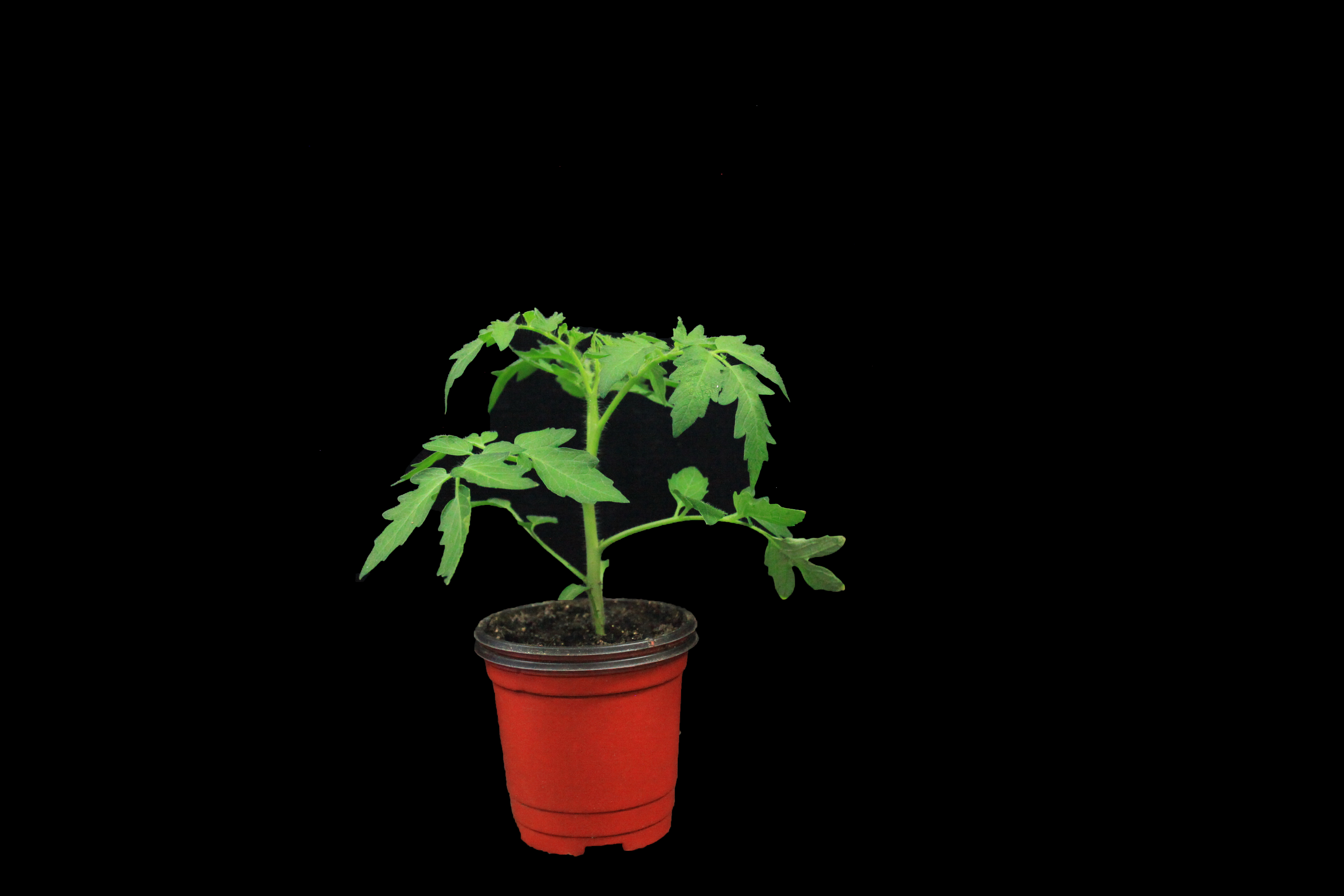

Supplement: Supplementary file 11 — Source data Fig. 2 [file 44318_2024_278_MOESM11_ESM.zip › Figure 2C/1_WT.jpg]

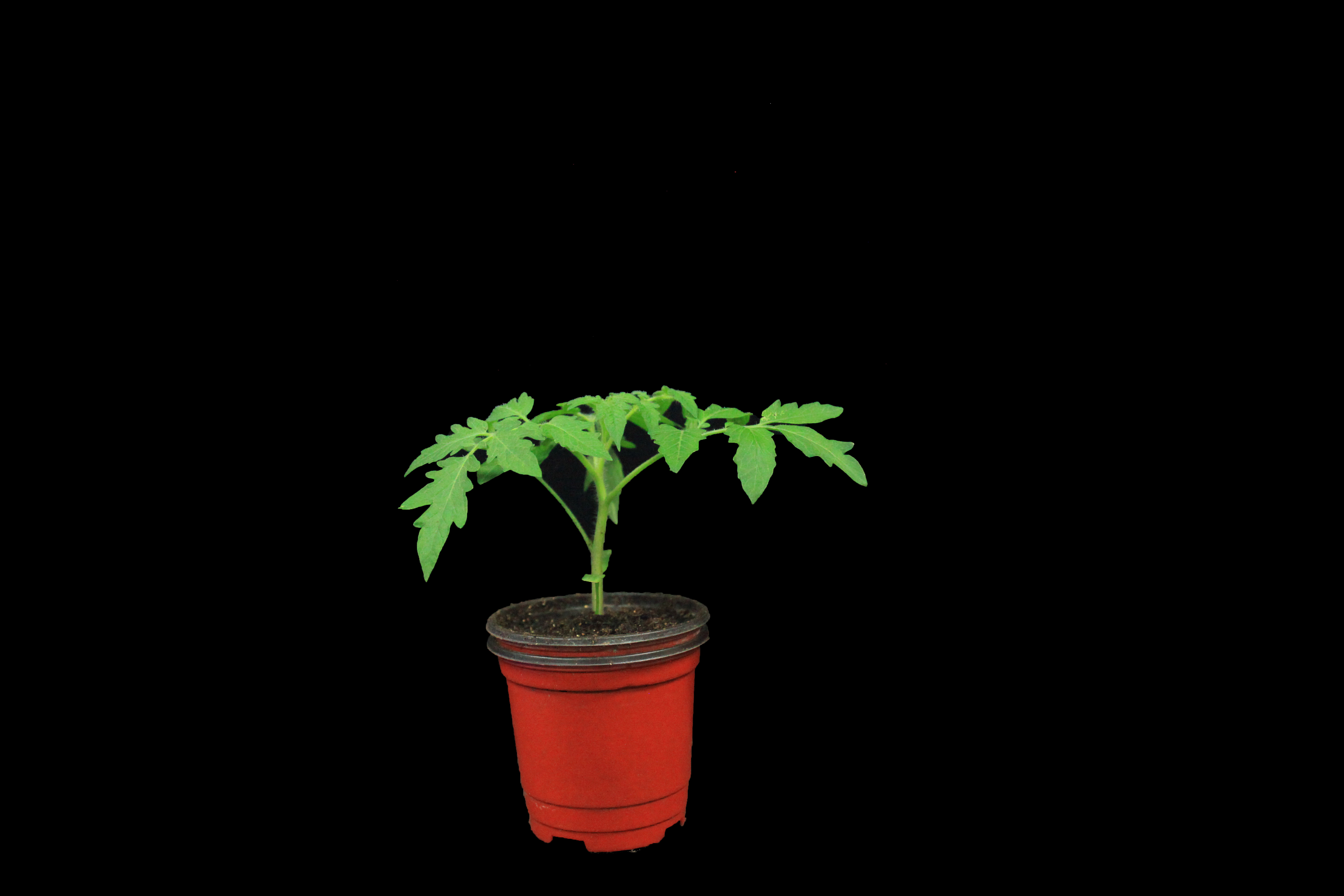

Supplement: Supplementary file 11 — Source data Fig. 2 [file 44318_2024_278_MOESM11_ESM.zip › Figure 2C/2_brak#4.jpg]

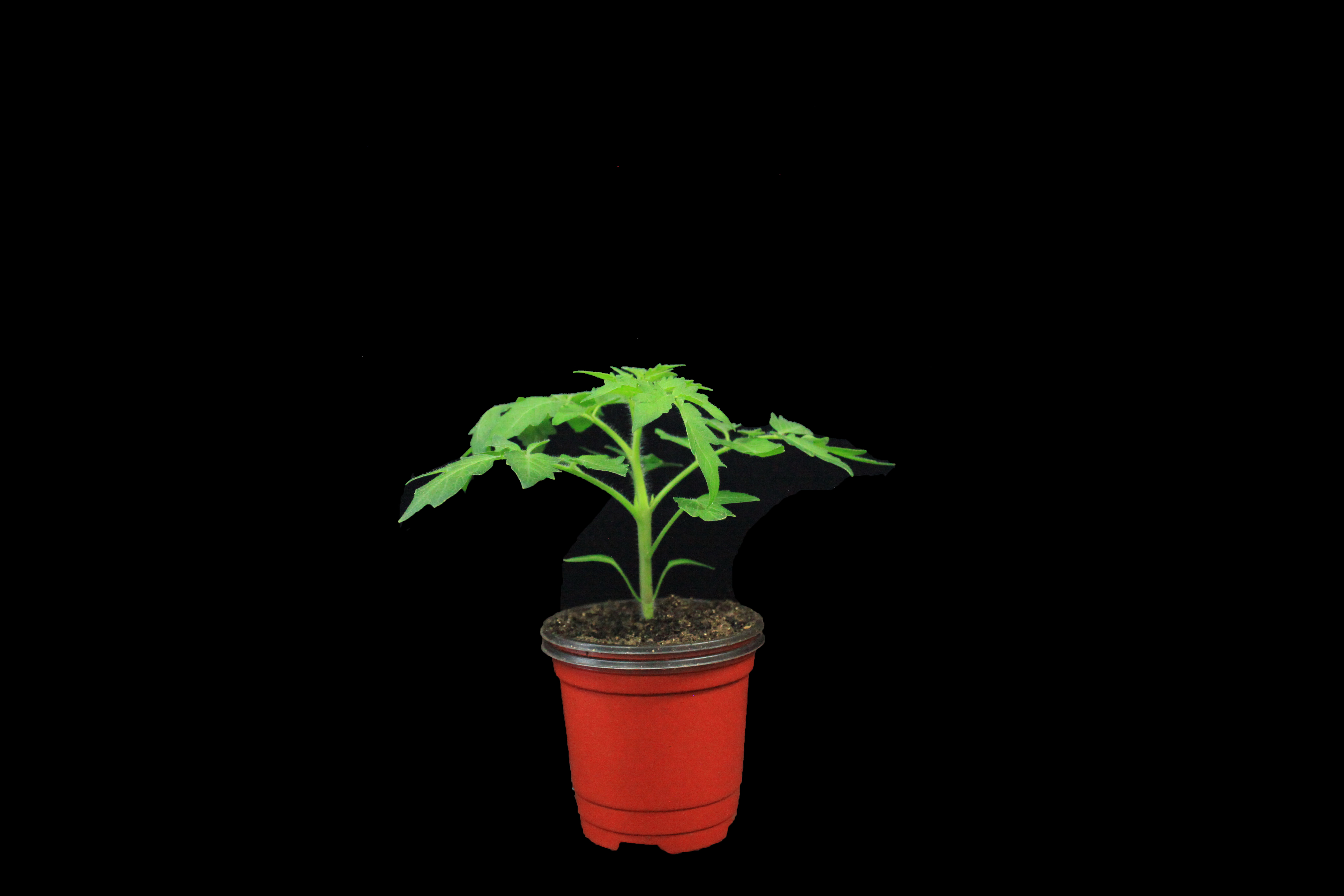

Supplement: Supplementary file 11 — Source data Fig. 2 [file 44318_2024_278_MOESM11_ESM.zip › Figure 2C/3_brak#5.jpg]

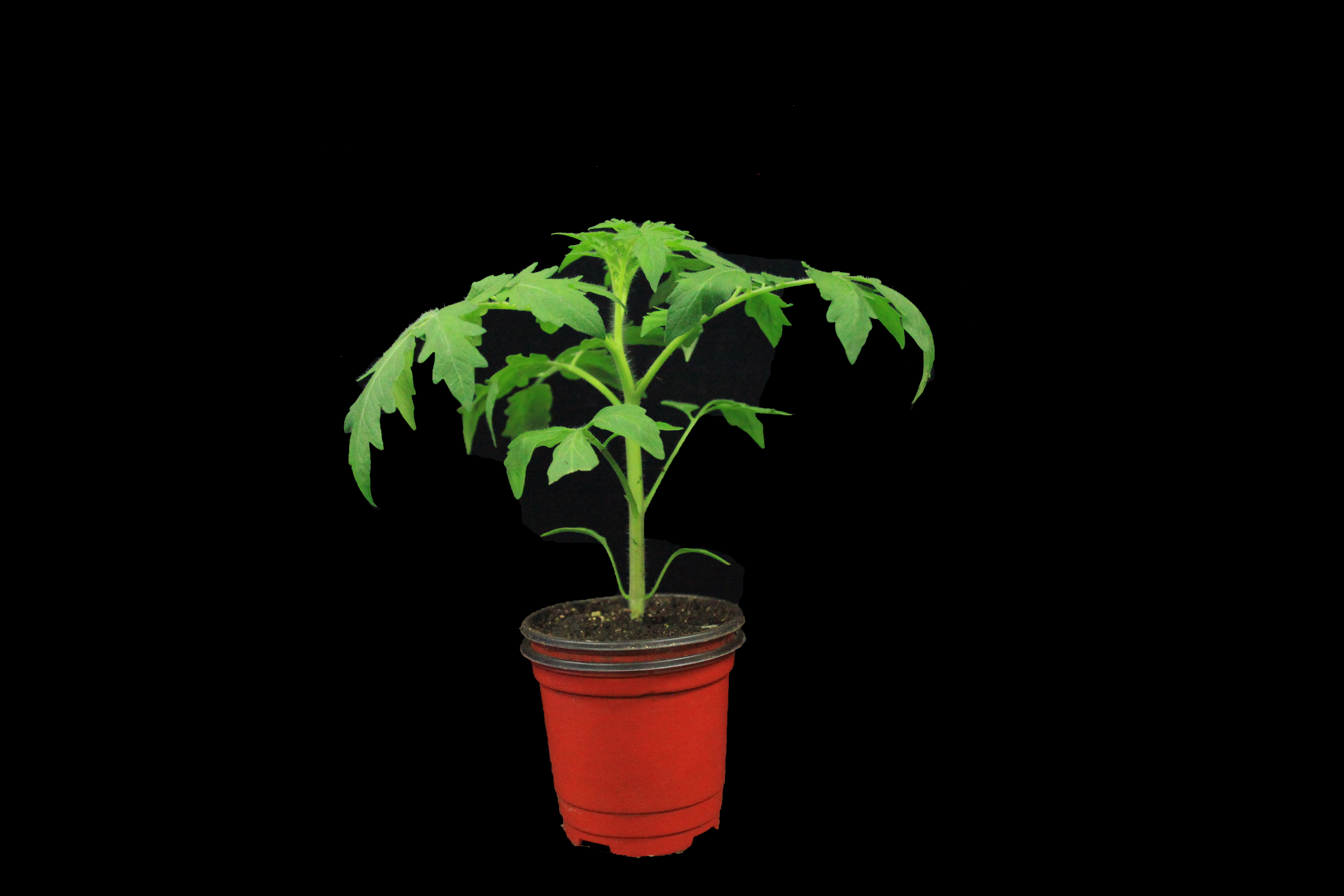

Supplement: Supplementary file 11 — Source data Fig. 2 [file 44318_2024_278_MOESM11_ESM.zip › Figure 2C/4_OE-BRAK#3.jpg]

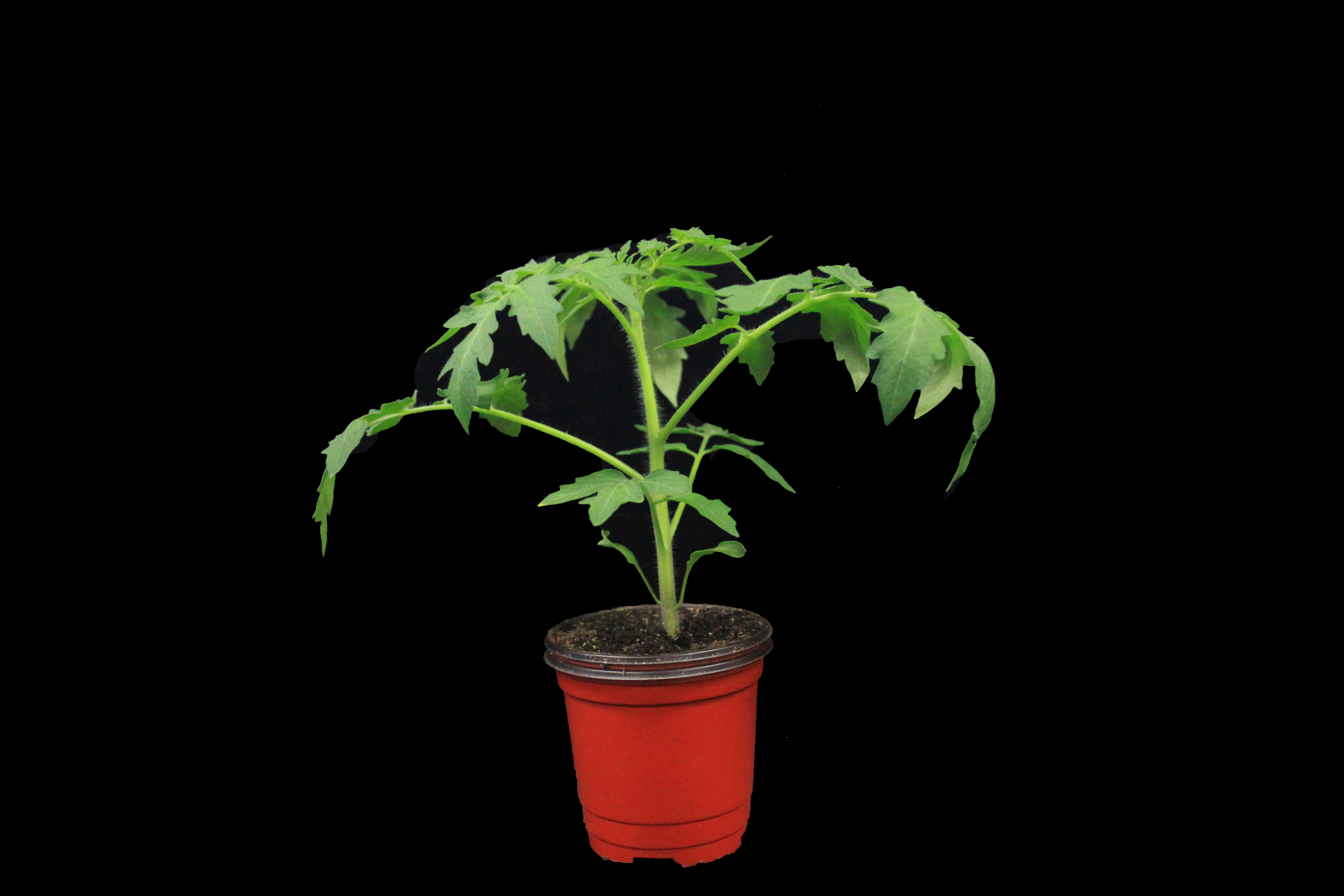

Supplement: Supplementary file 11 — Source data Fig. 2 [file 44318_2024_278_MOESM11_ESM.zip › Figure 2C/5_OE-BRAK#6.jpg]

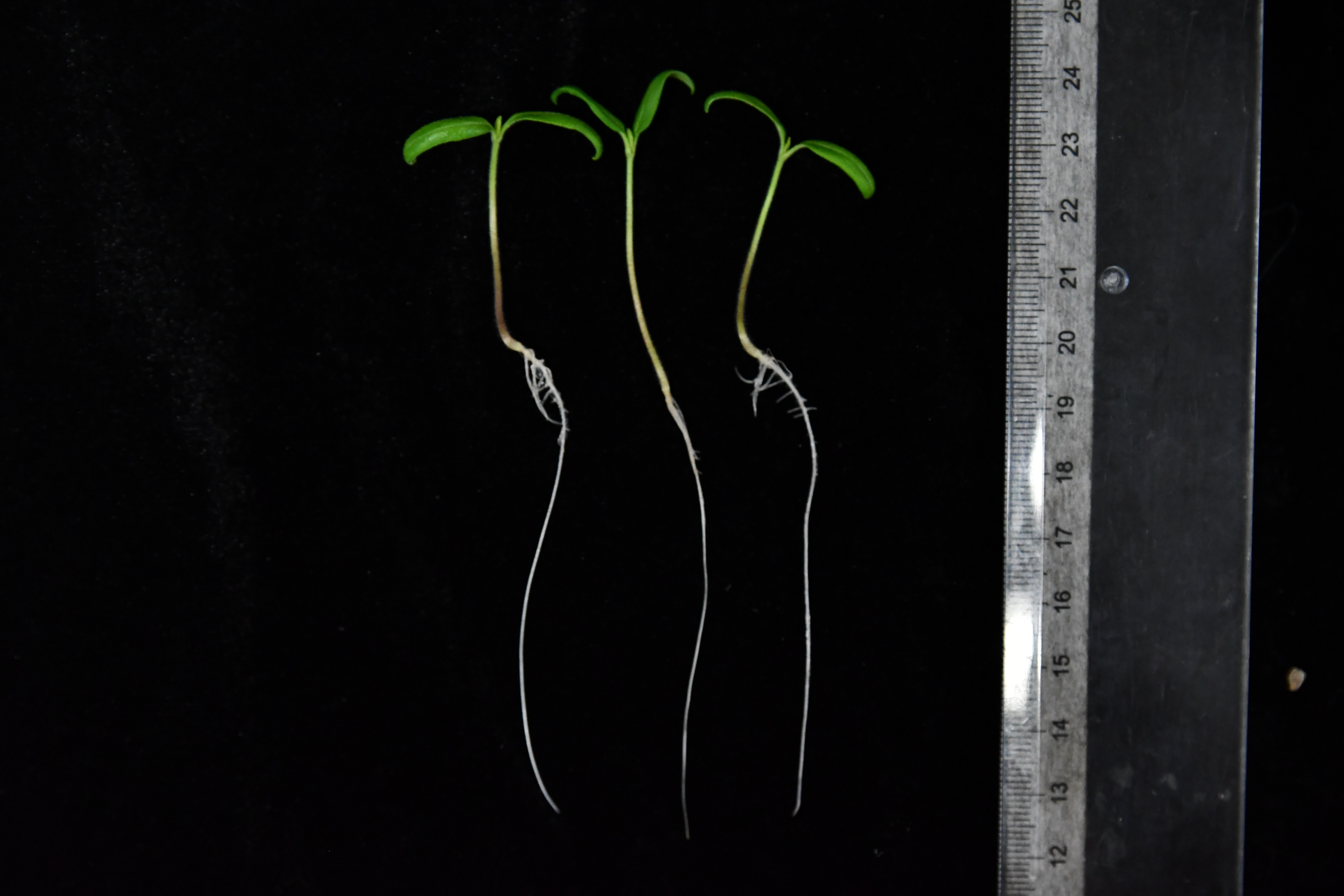

Supplement: Supplementary file 11 — Source data Fig. 2 [file 44318_2024_278_MOESM11_ESM.zip › Figure 2E/1_WT.JPG]

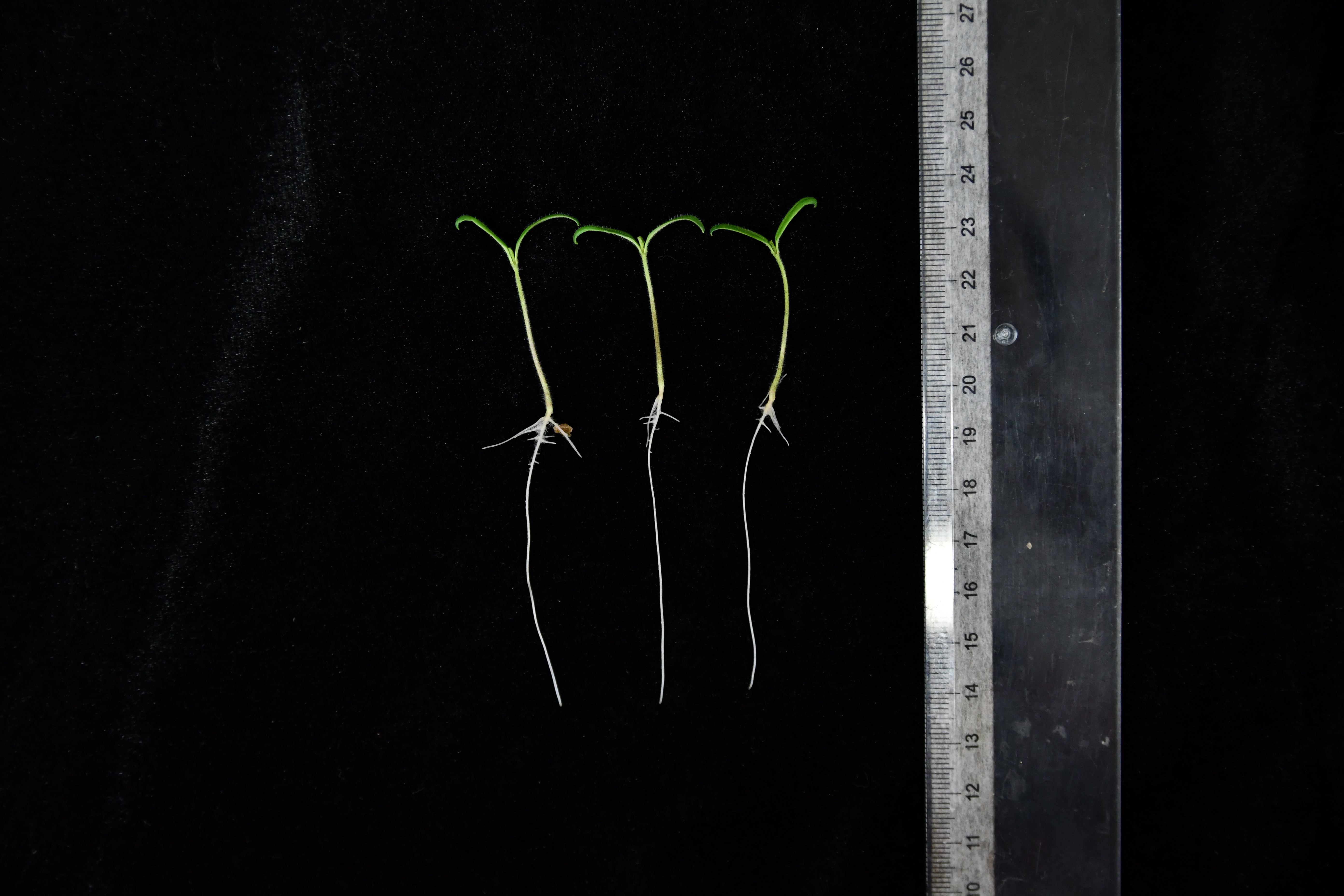

Supplement: Supplementary file 11 — Source data Fig. 2 [file 44318_2024_278_MOESM11_ESM.zip › Figure 2E/2_brak#4.JPG]

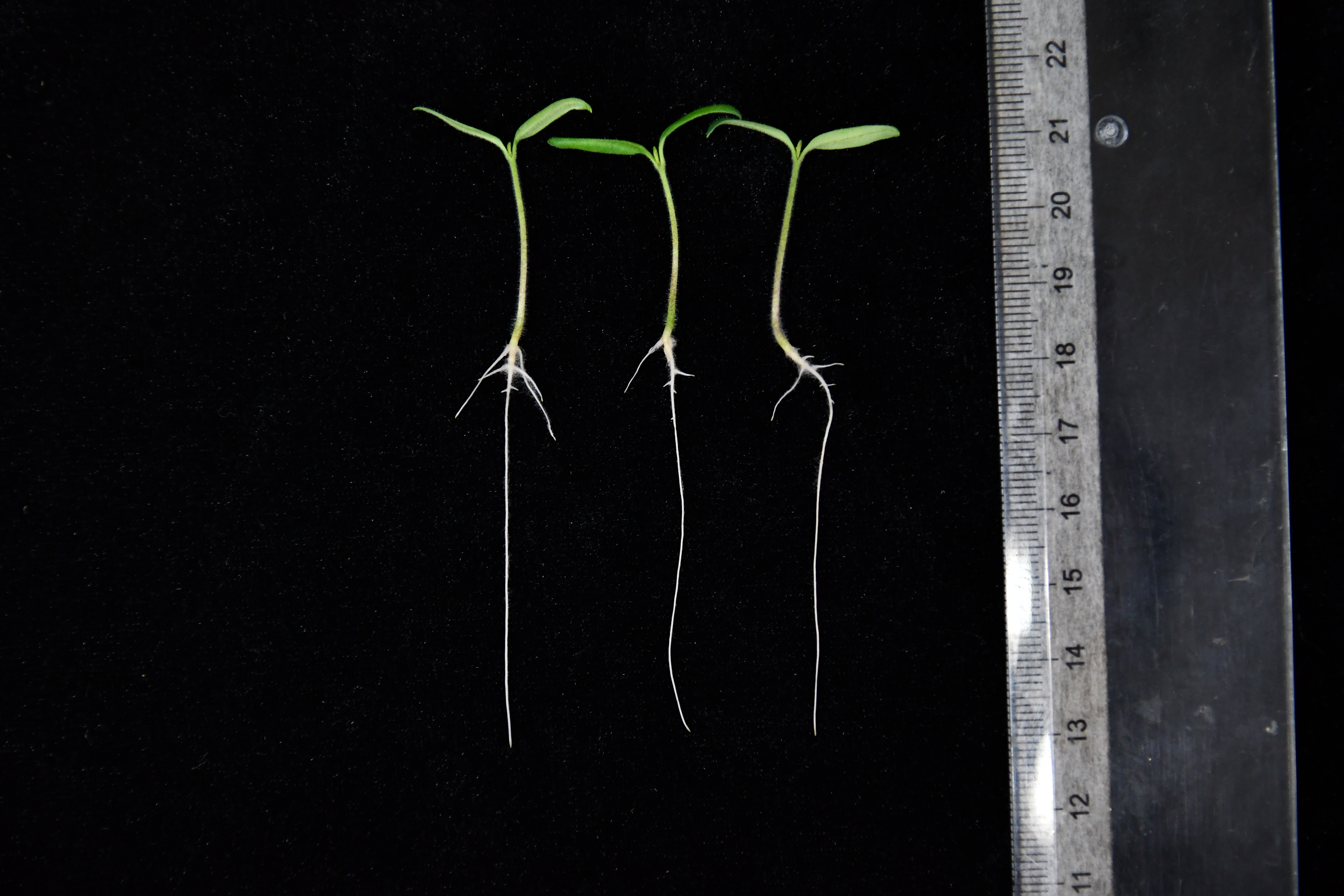

Supplement: Supplementary file 11 — Source data Fig. 2 [file 44318_2024_278_MOESM11_ESM.zip › Figure 2E/3_ brak#5.JPG]

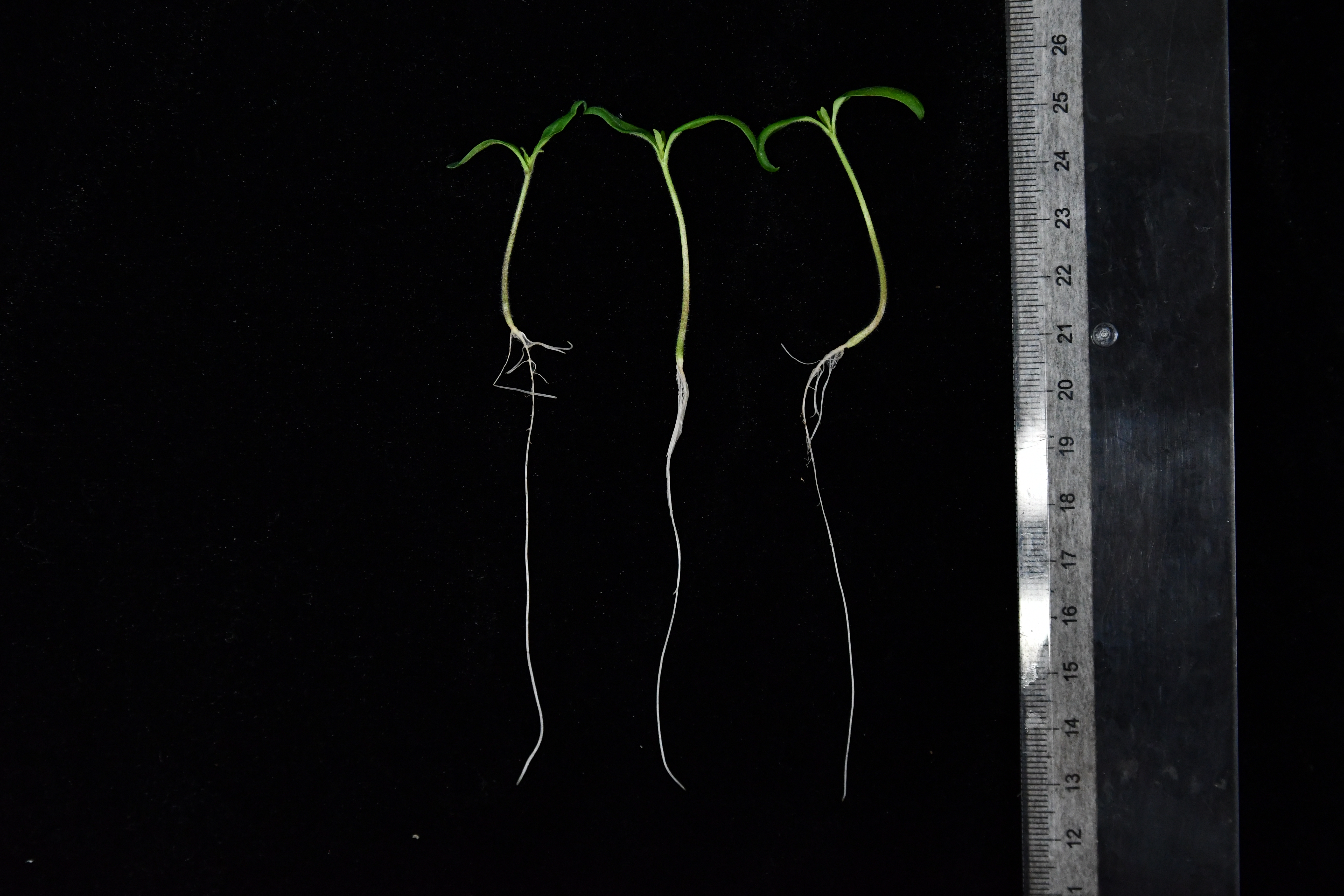

Supplement: Supplementary file 11 — Source data Fig. 2 [file 44318_2024_278_MOESM11_ESM.zip › Figure 2E/4_OE_BRAK#3.JPG]

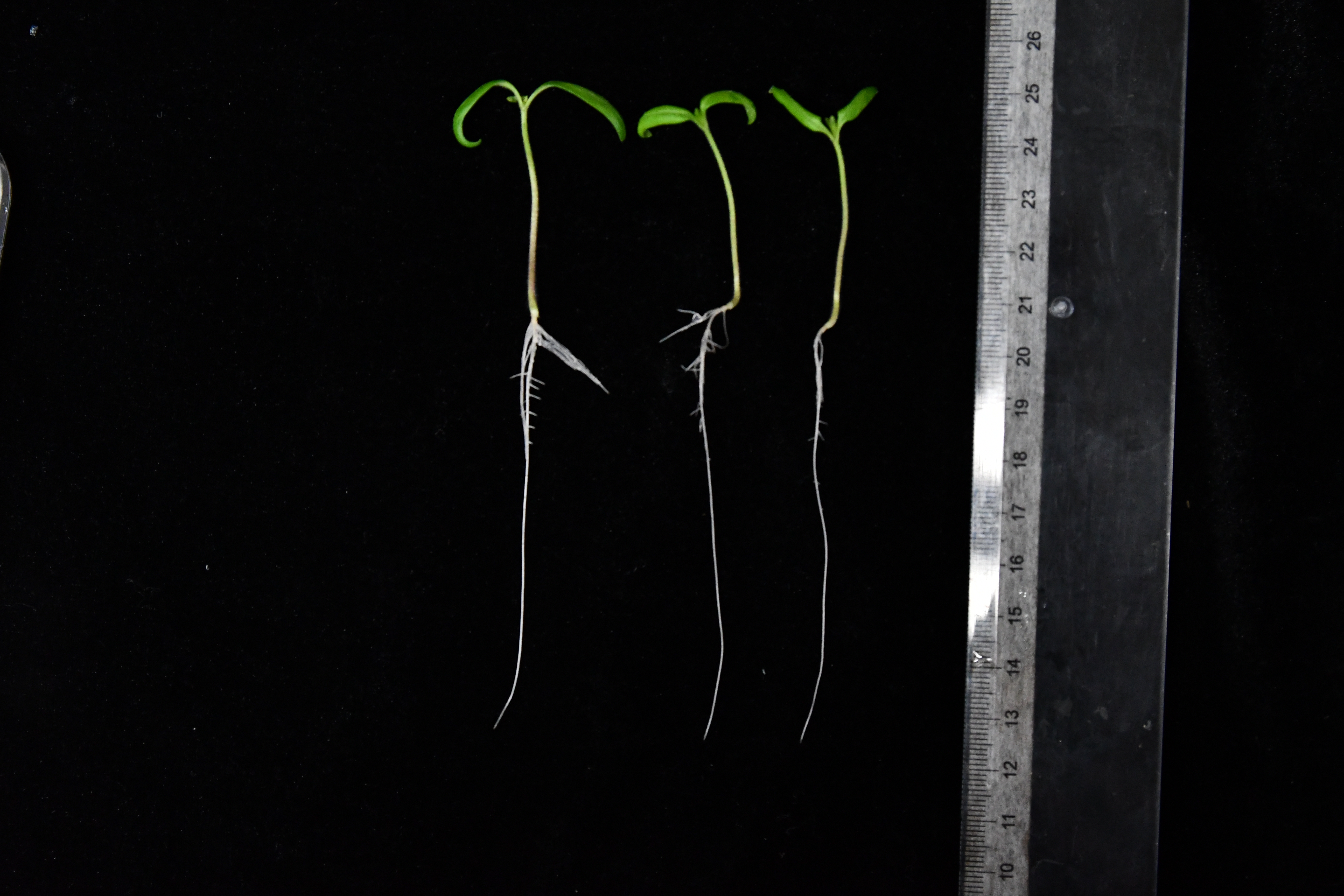

Supplement: Supplementary file 11 — Source data Fig. 2 [file 44318_2024_278_MOESM11_ESM.zip › Figure 2E/5_OE_BRAK#6.JPG]

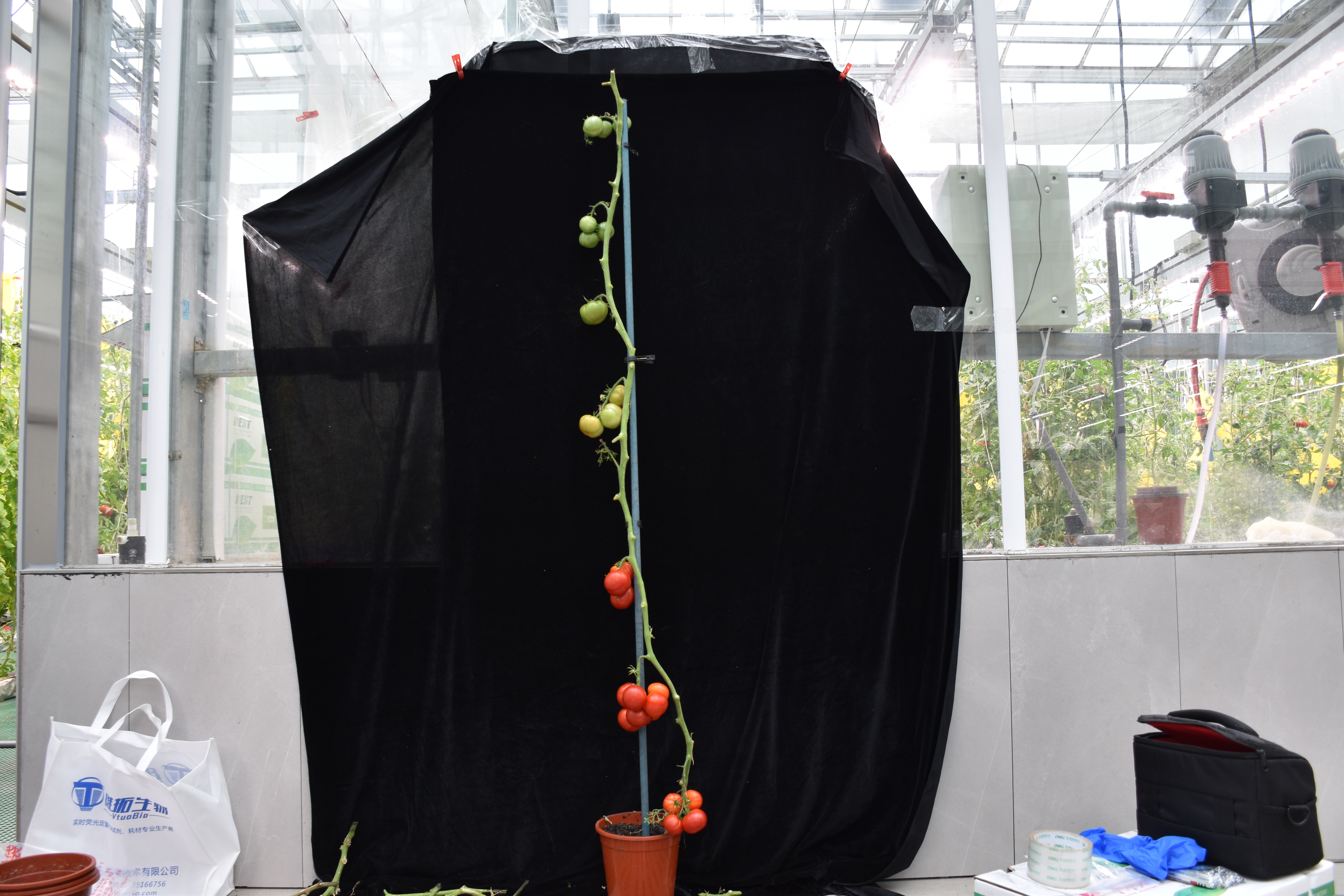

Supplement: Supplementary file 11 — Source data Fig. 2 [file 44318_2024_278_MOESM11_ESM.zip › Figure 2G/1_WT.JPG]

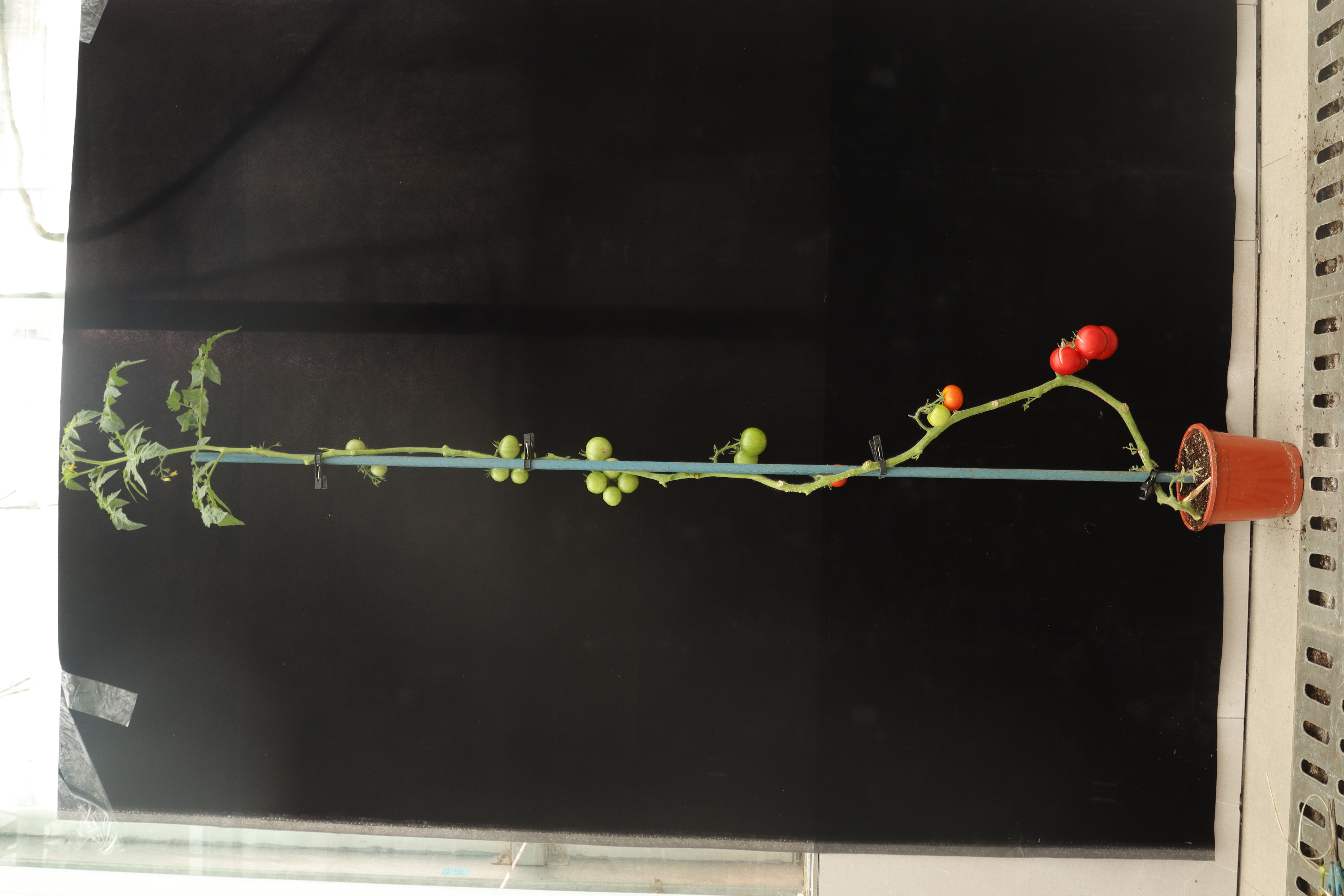

Supplement: Supplementary file 11 — Source data Fig. 2 [file 44318_2024_278_MOESM11_ESM.zip › Figure 2G/2_brak#4.JPG]

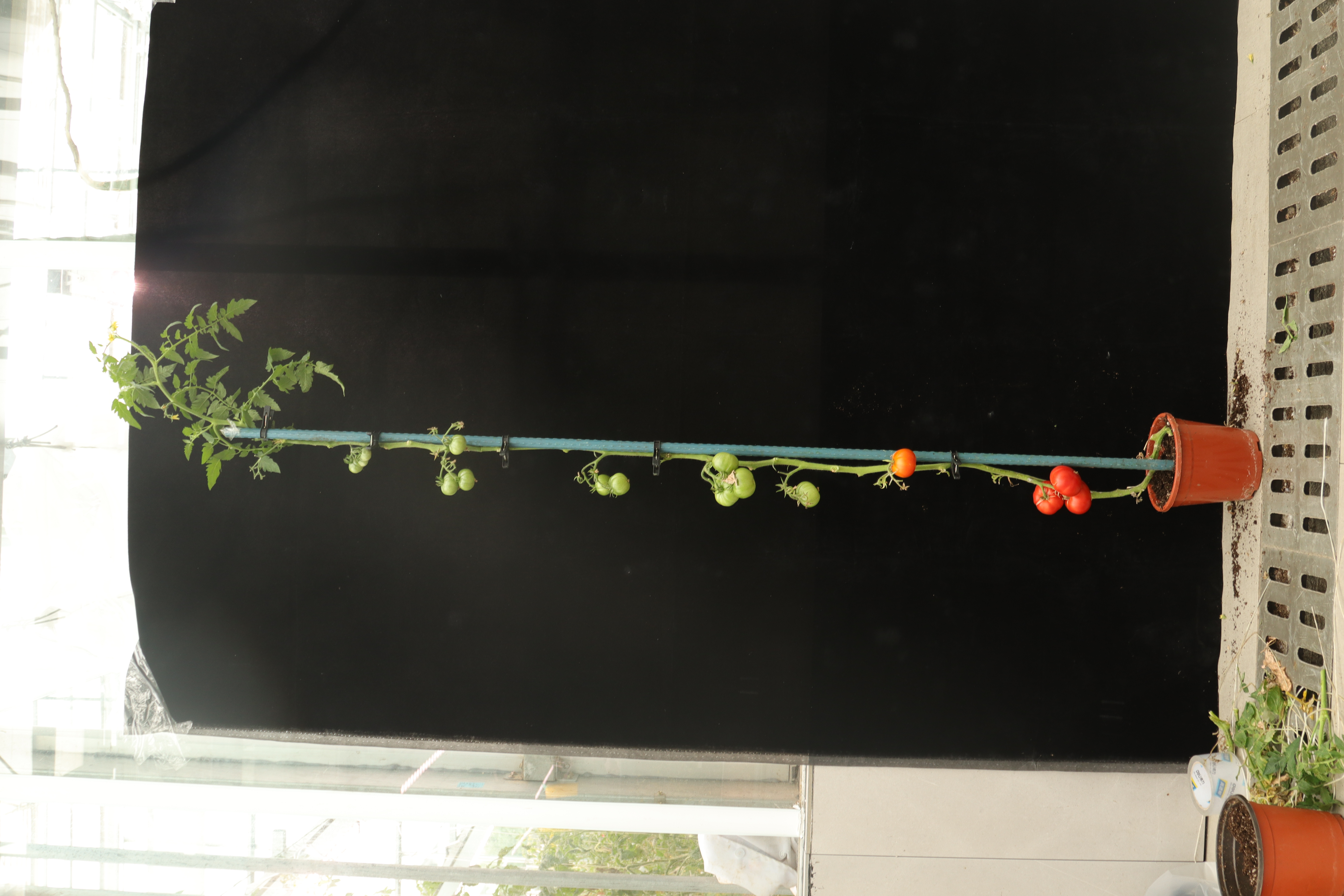

Supplement: Supplementary file 11 — Source data Fig. 2 [file 44318_2024_278_MOESM11_ESM.zip › Figure 2G/3_brak#5_IMG_7580.JPG]

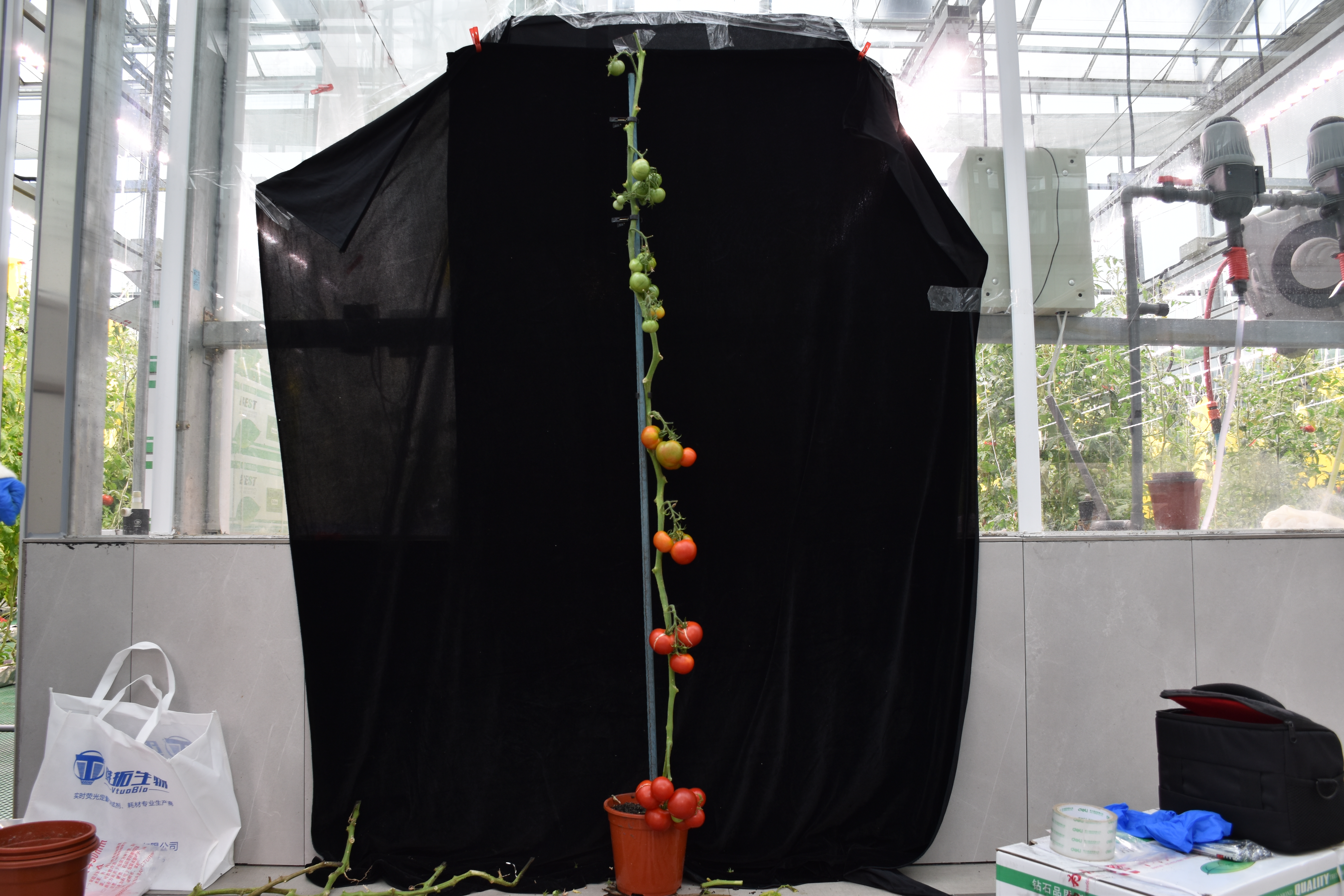

Supplement: Supplementary file 11 — Source data Fig. 2 [file 44318_2024_278_MOESM11_ESM.zip › Figure 2G/4_OE_BRAK#3_DSC_0084.JPG]

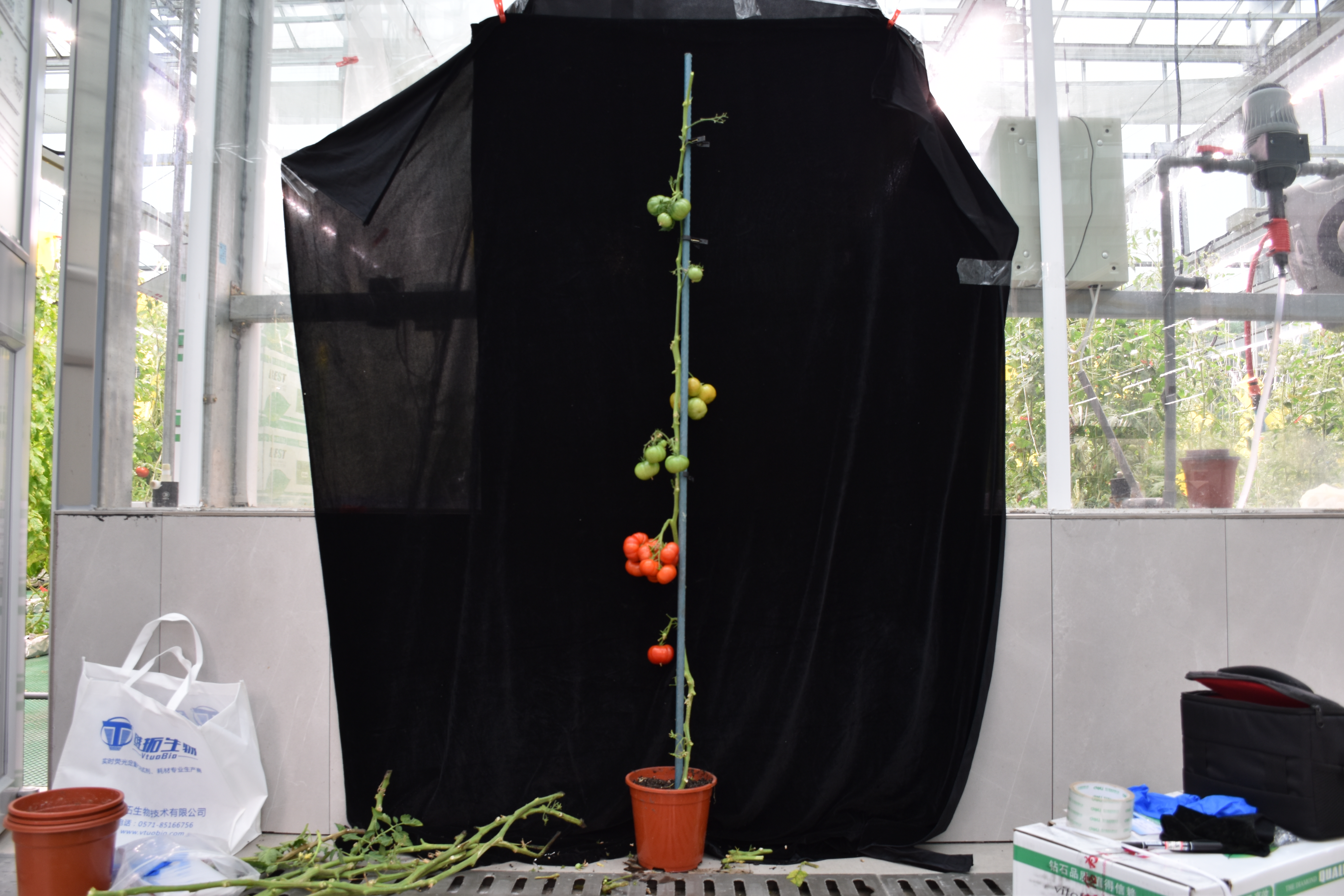

Supplement: Supplementary file 11 — Source data Fig. 2 [file 44318_2024_278_MOESM11_ESM.zip › Figure 2G/5_OE_BRAK#6_DSC_0112.JPG]

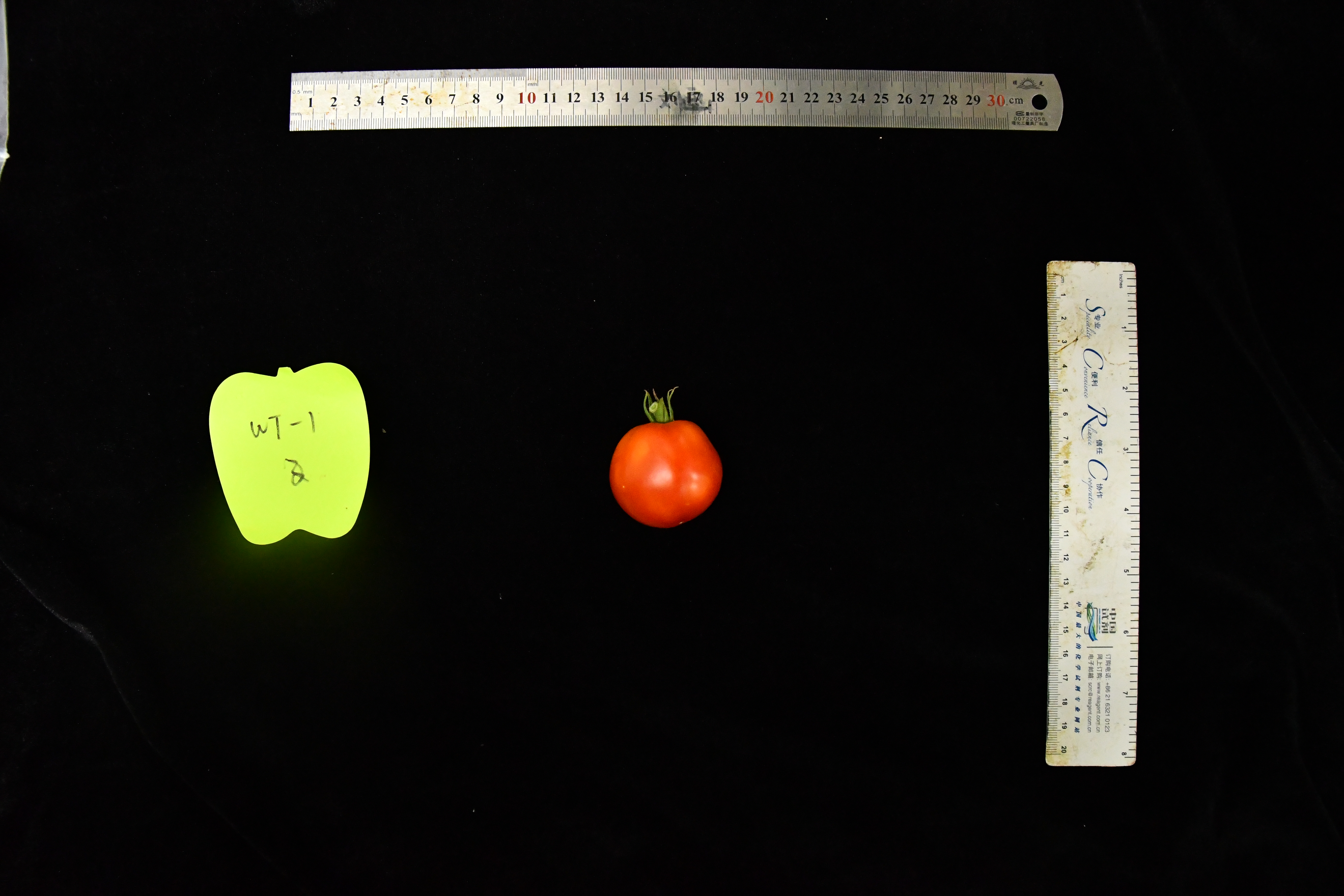

Supplement: Supplementary file 11 — Source data Fig. 2 [file 44318_2024_278_MOESM11_ESM.zip › Figure 2H/1.WT.JPG]

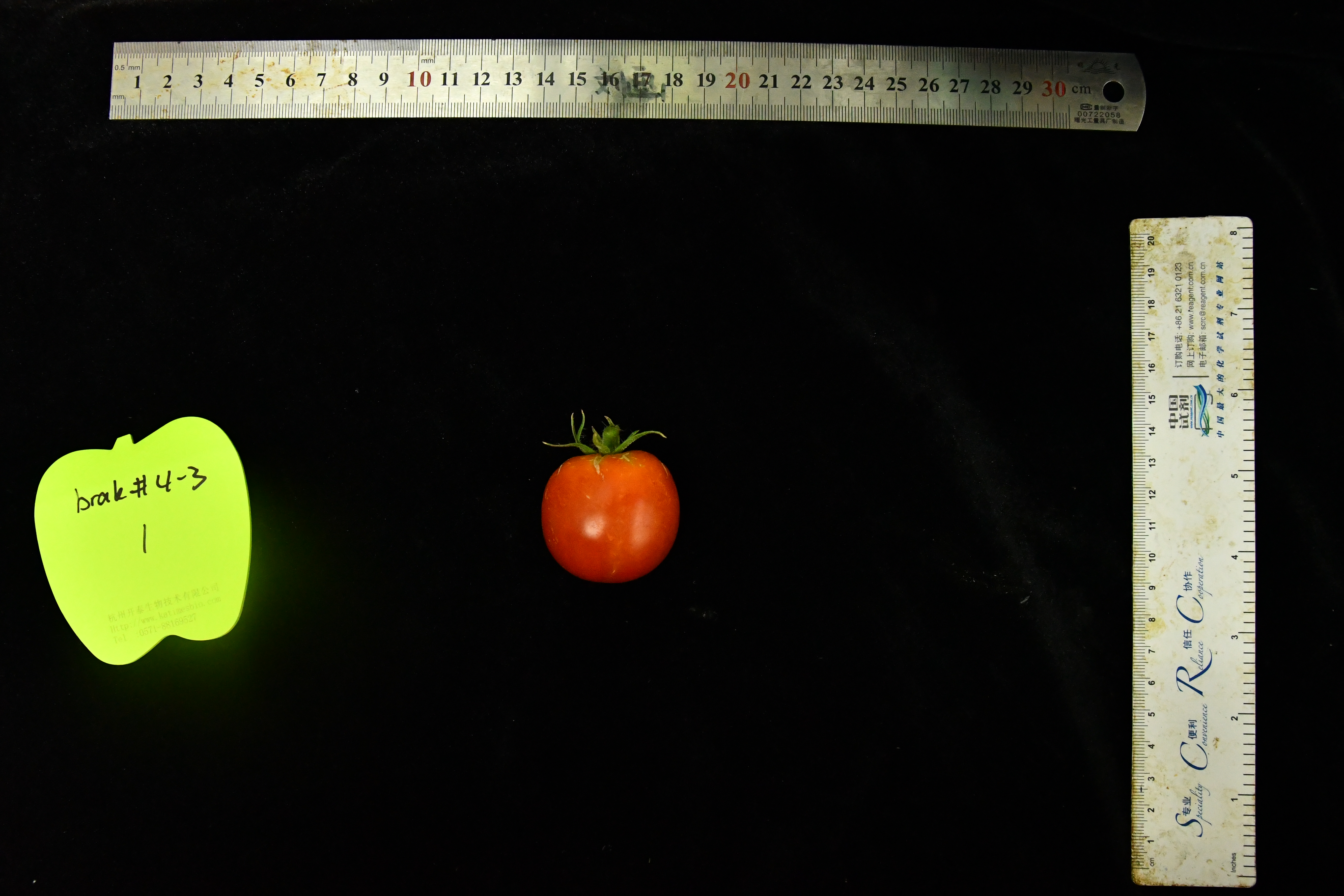

Supplement: Supplementary file 11 — Source data Fig. 2 [file 44318_2024_278_MOESM11_ESM.zip › Figure 2H/2_brak#4.JPG]

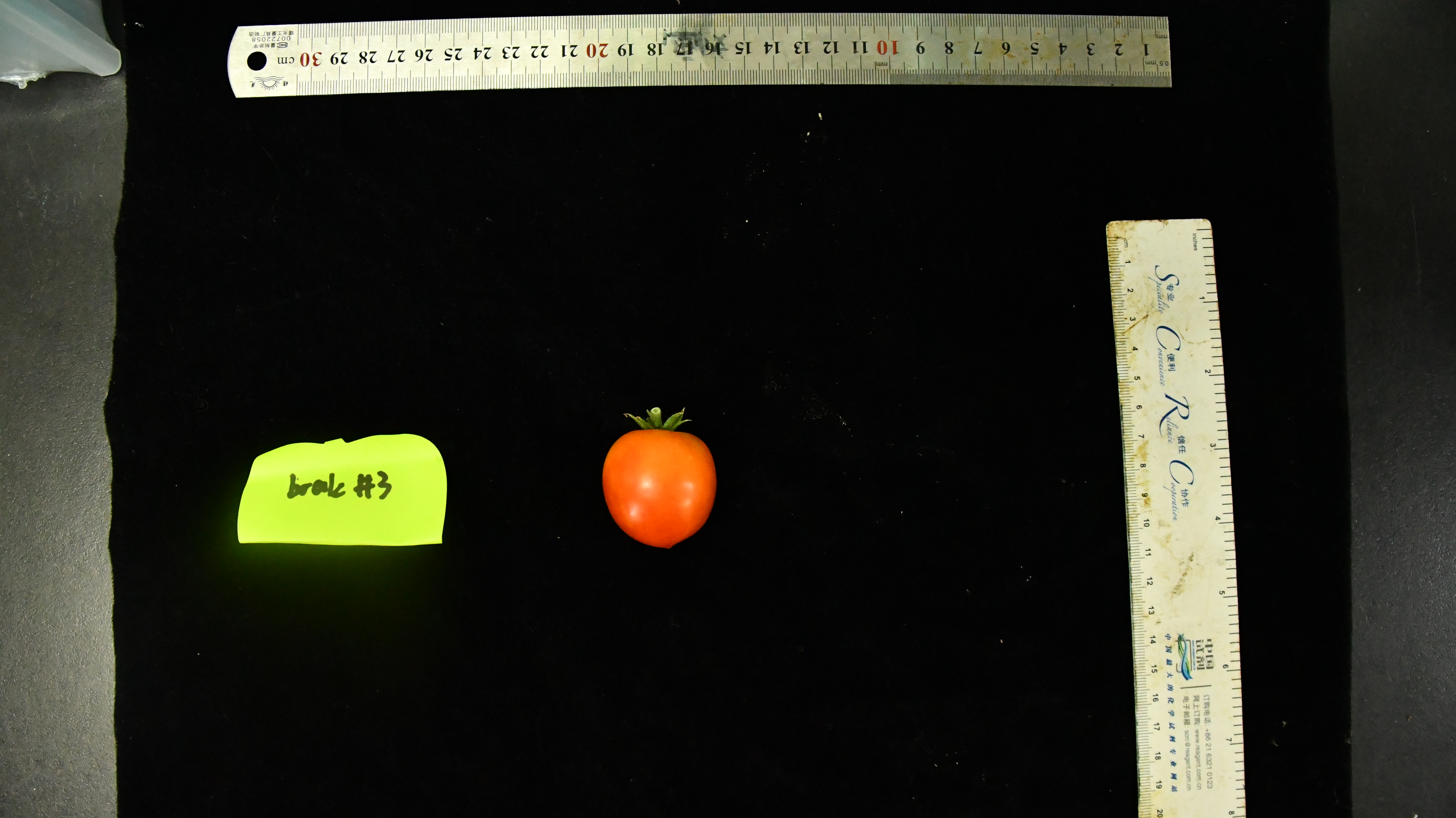

Supplement: Supplementary file 11 — Source data Fig. 2 [file 44318_2024_278_MOESM11_ESM.zip › Figure 2H/3_brak#5.JPG]

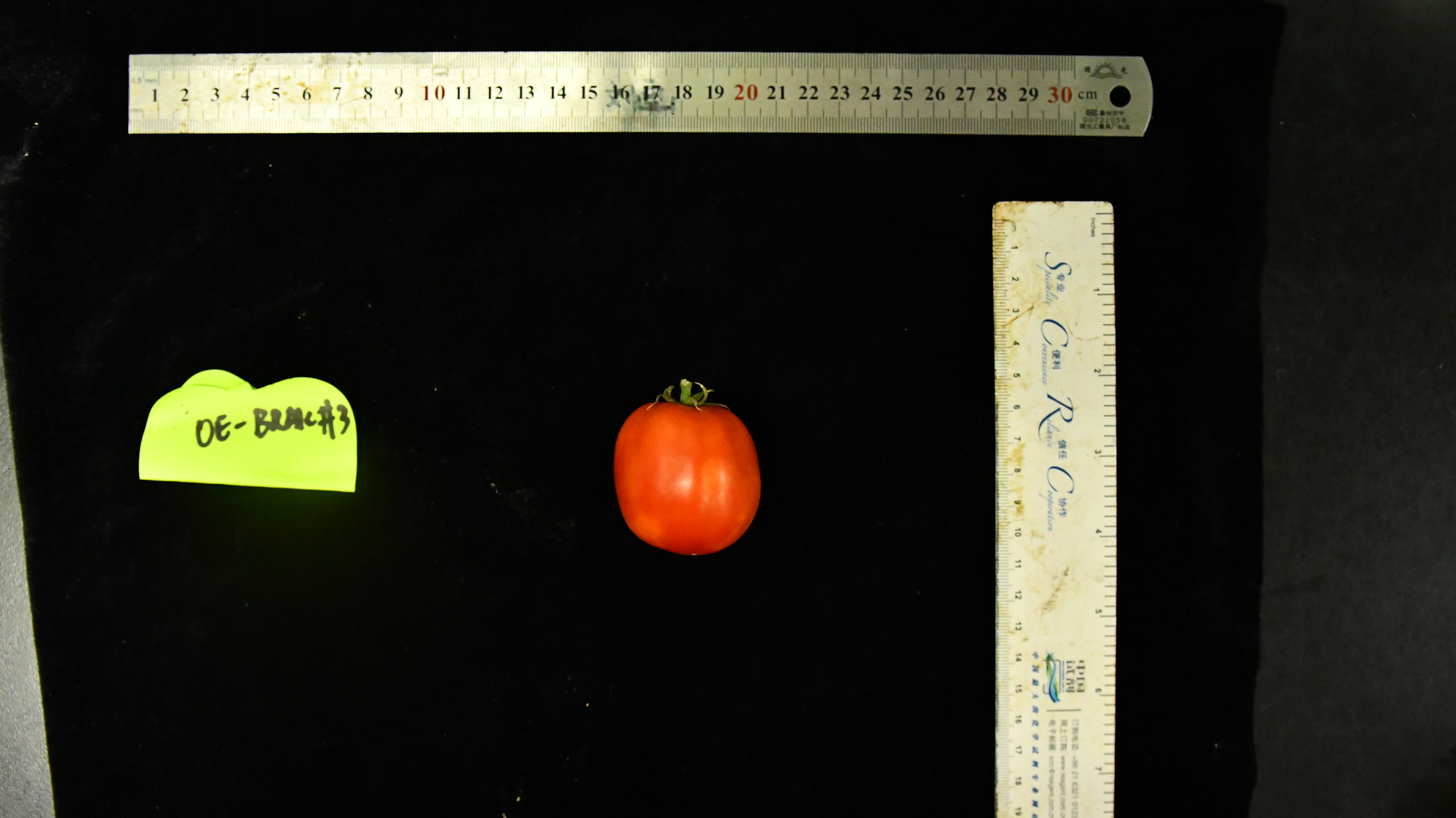

Supplement: Supplementary file 11 — Source data Fig. 2 [file 44318_2024_278_MOESM11_ESM.zip › Figure 2H/4_OE_BRAK#3_DSC_0318(选).JPG]

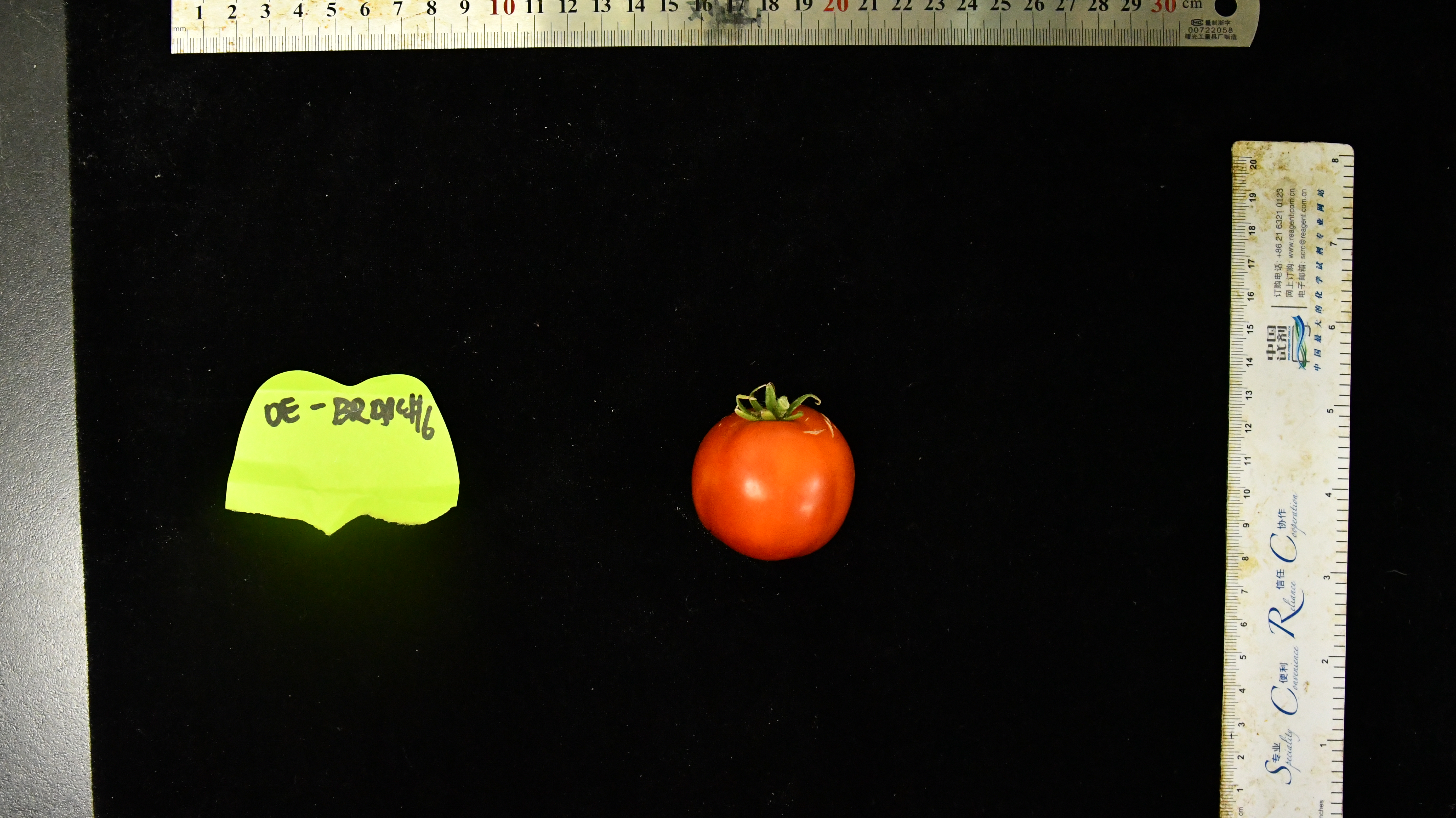

Supplement: Supplementary file 11 — Source data Fig. 2 [file 44318_2024_278_MOESM11_ESM.zip › Figure 2H/5_OE_BRAK#6_DSC_0391(选).JPG]

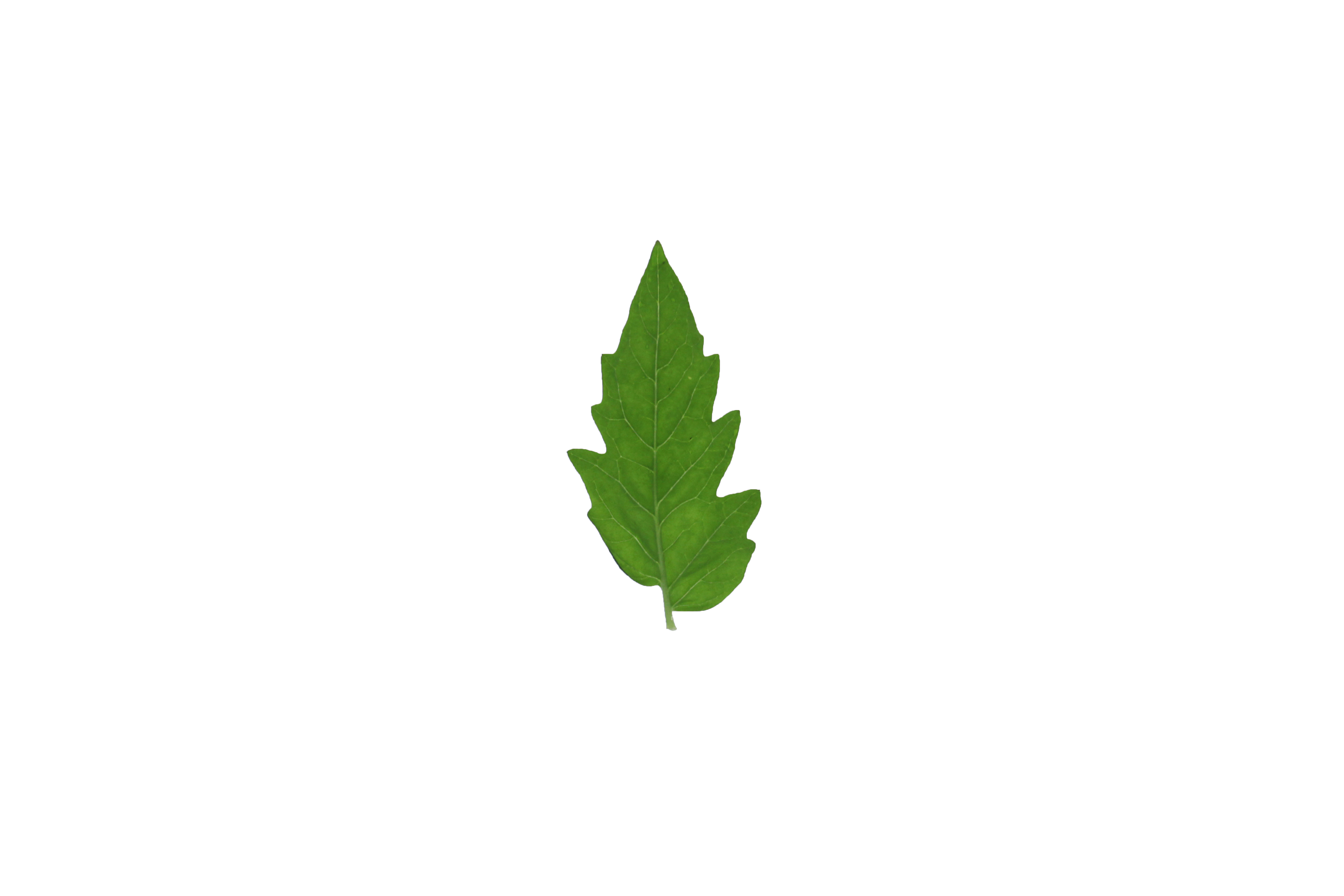

Supplement: Supplementary file 11 — Source data Fig. 2 [file 44318_2024_278_MOESM11_ESM.zip › Figure 2L/1_WT Mock.jpg]

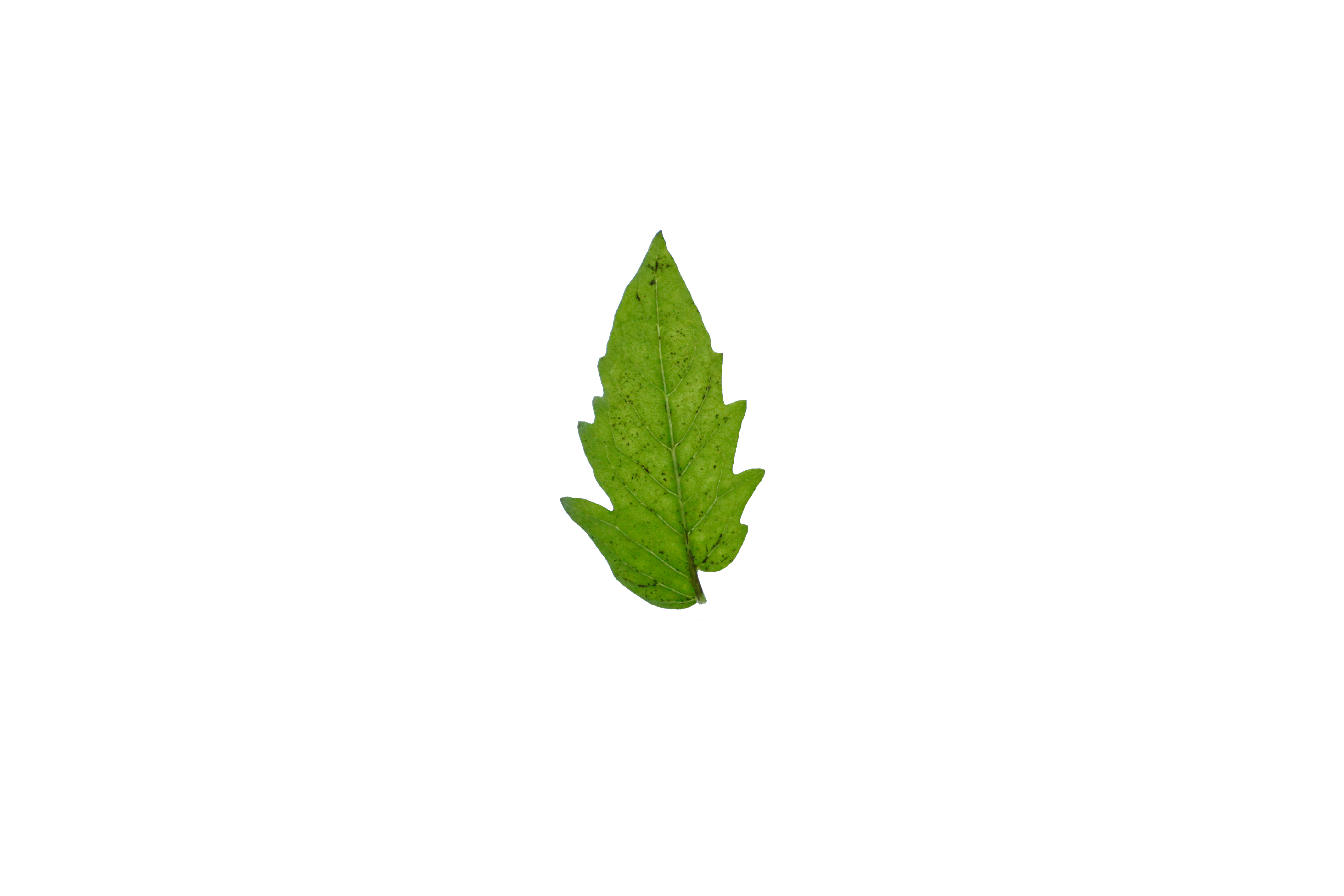

Supplement: Supplementary file 11 — Source data Fig. 2 [file 44318_2024_278_MOESM11_ESM.zip › Figure 2L/2_WT B.c.jpg]

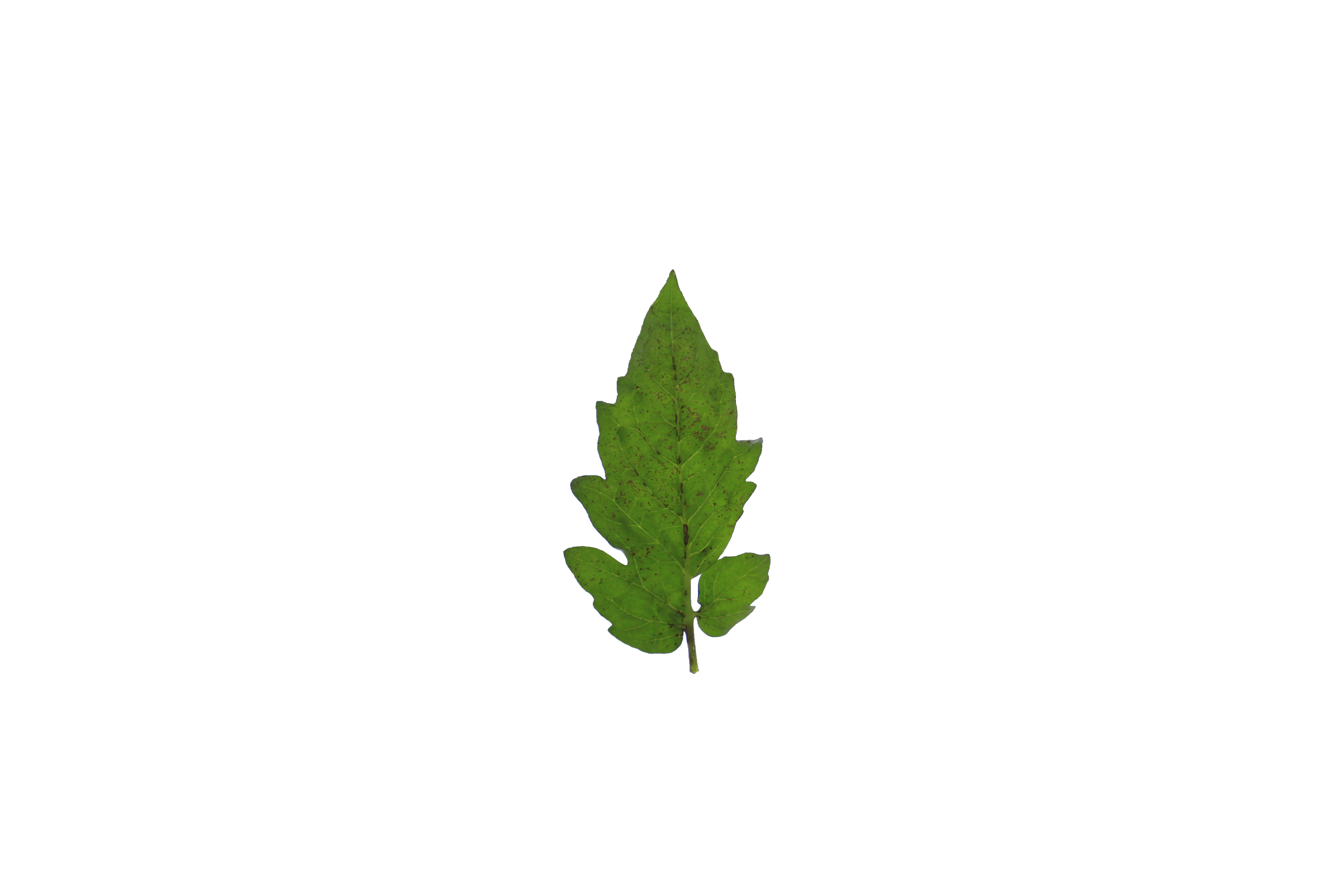

Supplement: Supplementary file 11 — Source data Fig. 2 [file 44318_2024_278_MOESM11_ESM.zip › Figure 2L/3_brak#4 B.c.jpg]

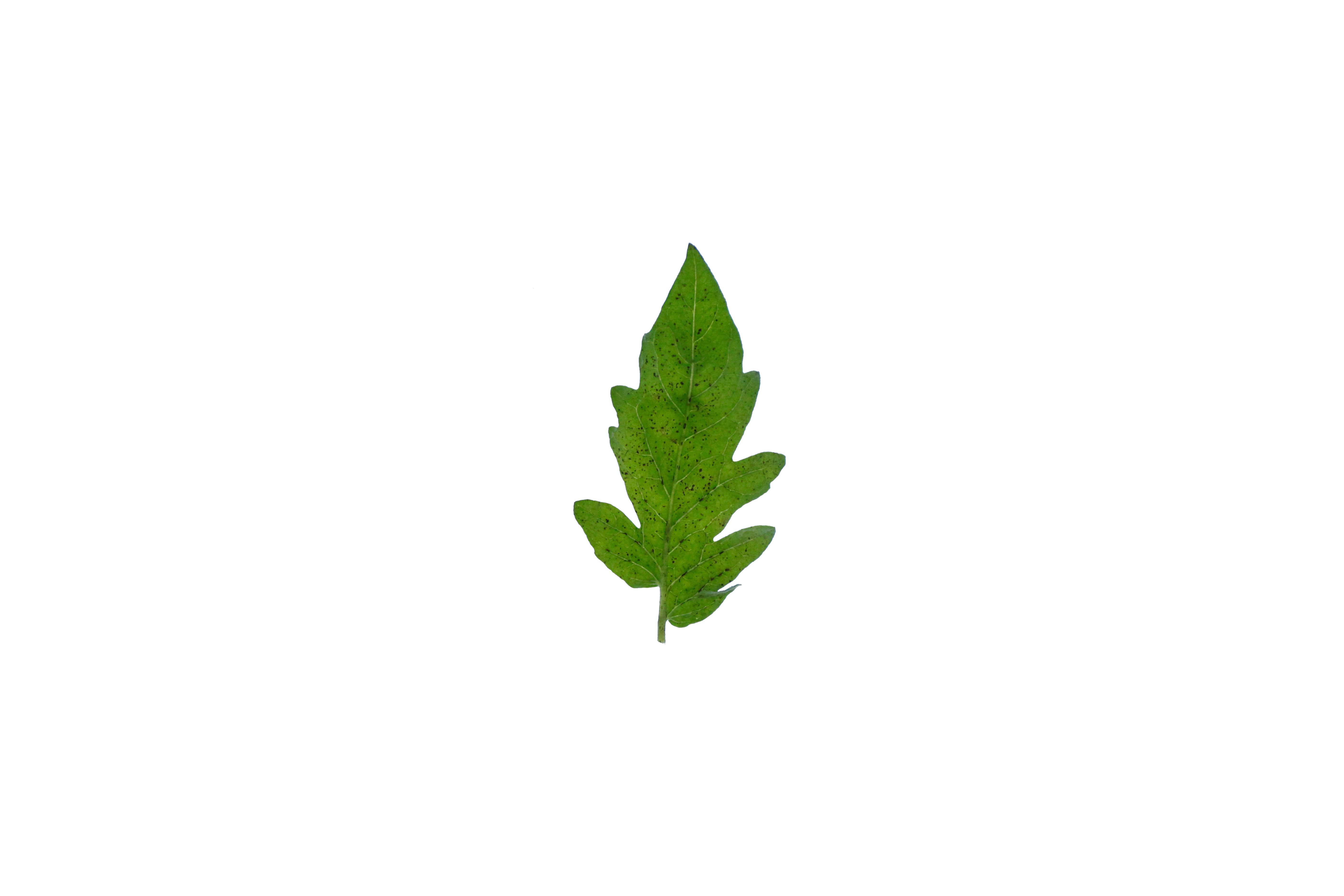

Supplement: Supplementary file 11 — Source data Fig. 2 [file 44318_2024_278_MOESM11_ESM.zip › Figure 2L/4_brak#5 B.c.jpg]

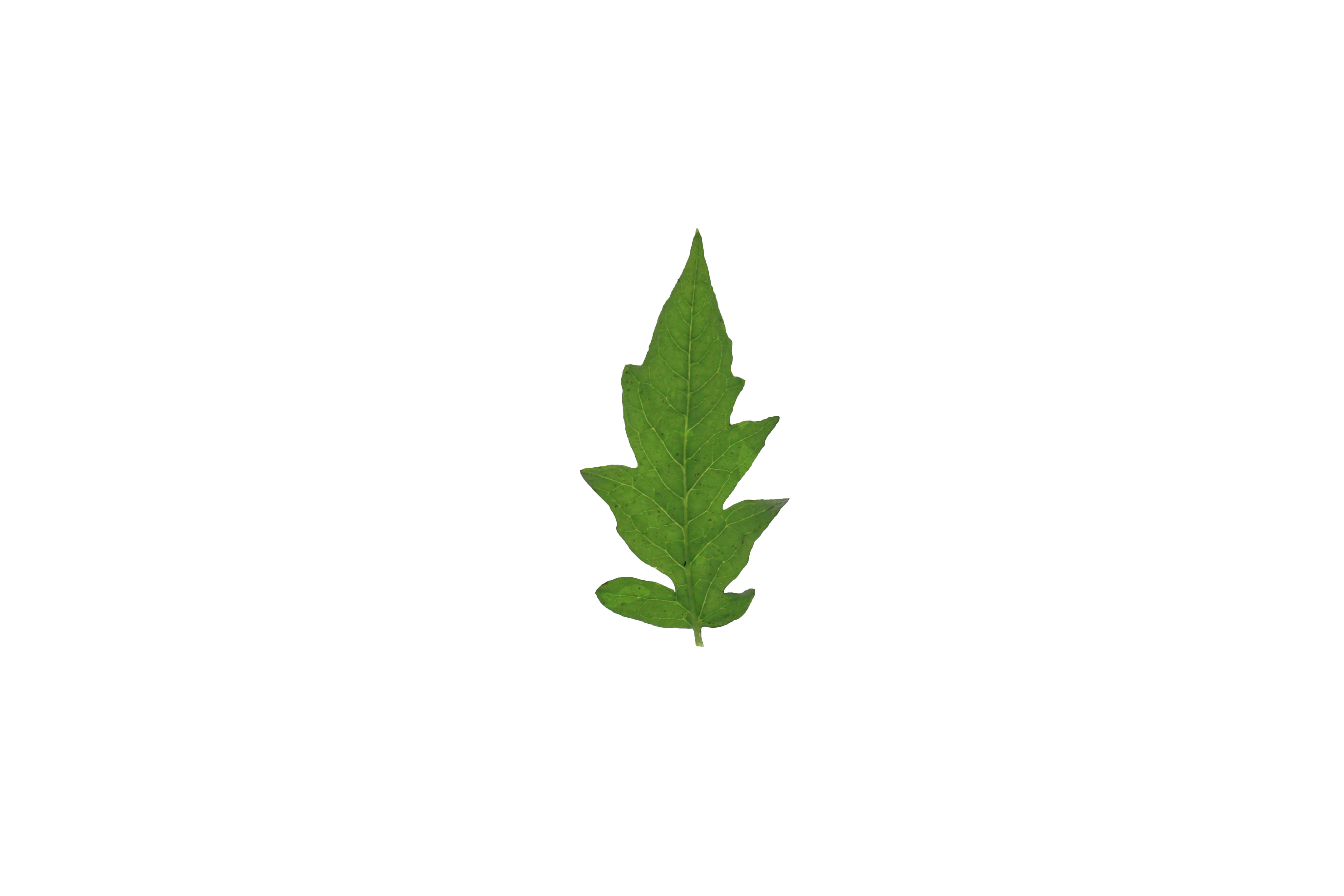

Supplement: Supplementary file 11 — Source data Fig. 2 [file 44318_2024_278_MOESM11_ESM.zip › Figure 2L/5_OE-BRAK#3 B.c.jpg]

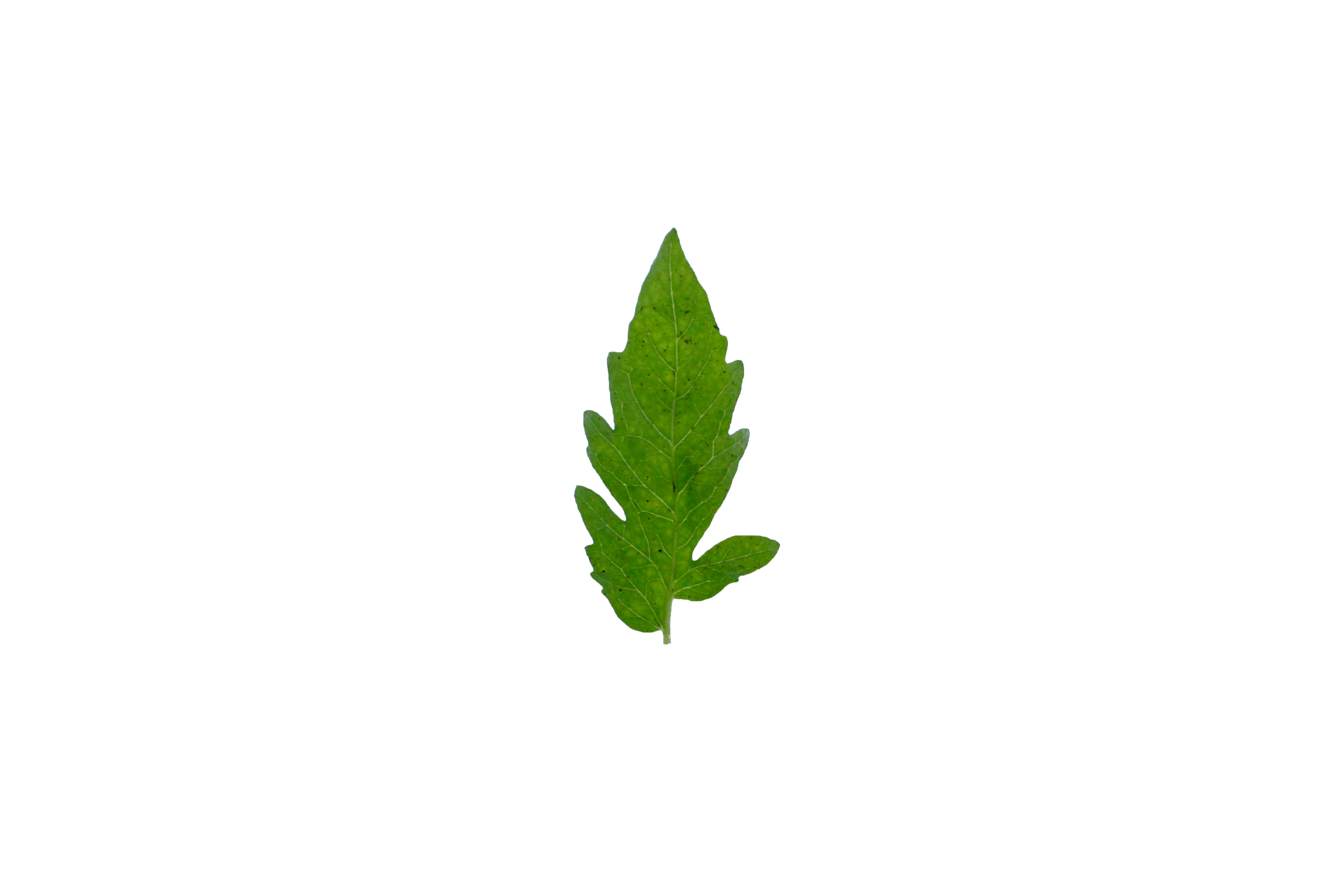

Supplement: Supplementary file 11 — Source data Fig. 2 [file 44318_2024_278_MOESM11_ESM.zip › Figure 2L/6_OE-BRAK#6.jpg]

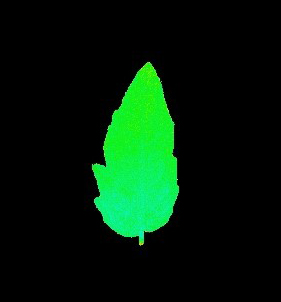

Supplement: Supplementary file 11 — Source data Fig. 2 [file 44318_2024_278_MOESM11_ESM.zip › Figure 2N/1_WT mock.jpg]

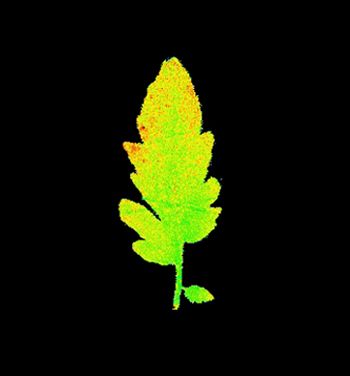

Supplement: Supplementary file 11 — Source data Fig. 2 [file 44318_2024_278_MOESM11_ESM.zip › Figure 2N/2_WT B.c.jpg]

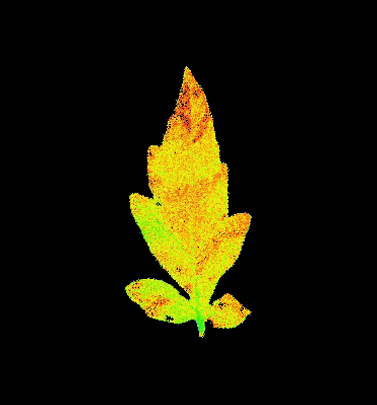

Supplement: Supplementary file 11 — Source data Fig. 2 [file 44318_2024_278_MOESM11_ESM.zip › Figure 2N/3_brak#4 B.c.jpg]

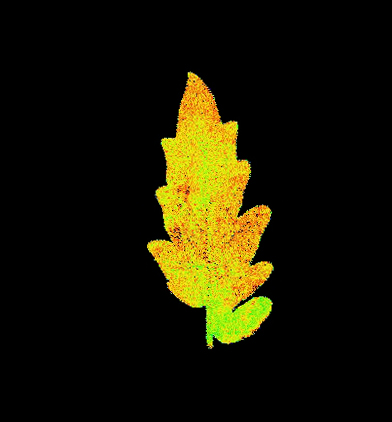

Supplement: Supplementary file 11 — Source data Fig. 2 [file 44318_2024_278_MOESM11_ESM.zip › Figure 2N/4_brak#5 B.c.jpg]

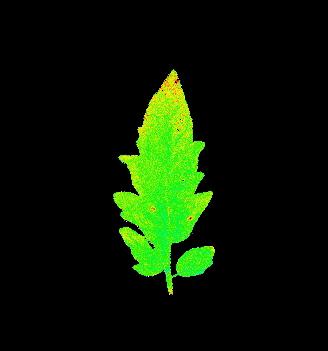

Supplement: Supplementary file 11 — Source data Fig. 2 [file 44318_2024_278_MOESM11_ESM.zip › Figure 2N/5_OE-BRAK#3 B.c.jpg]

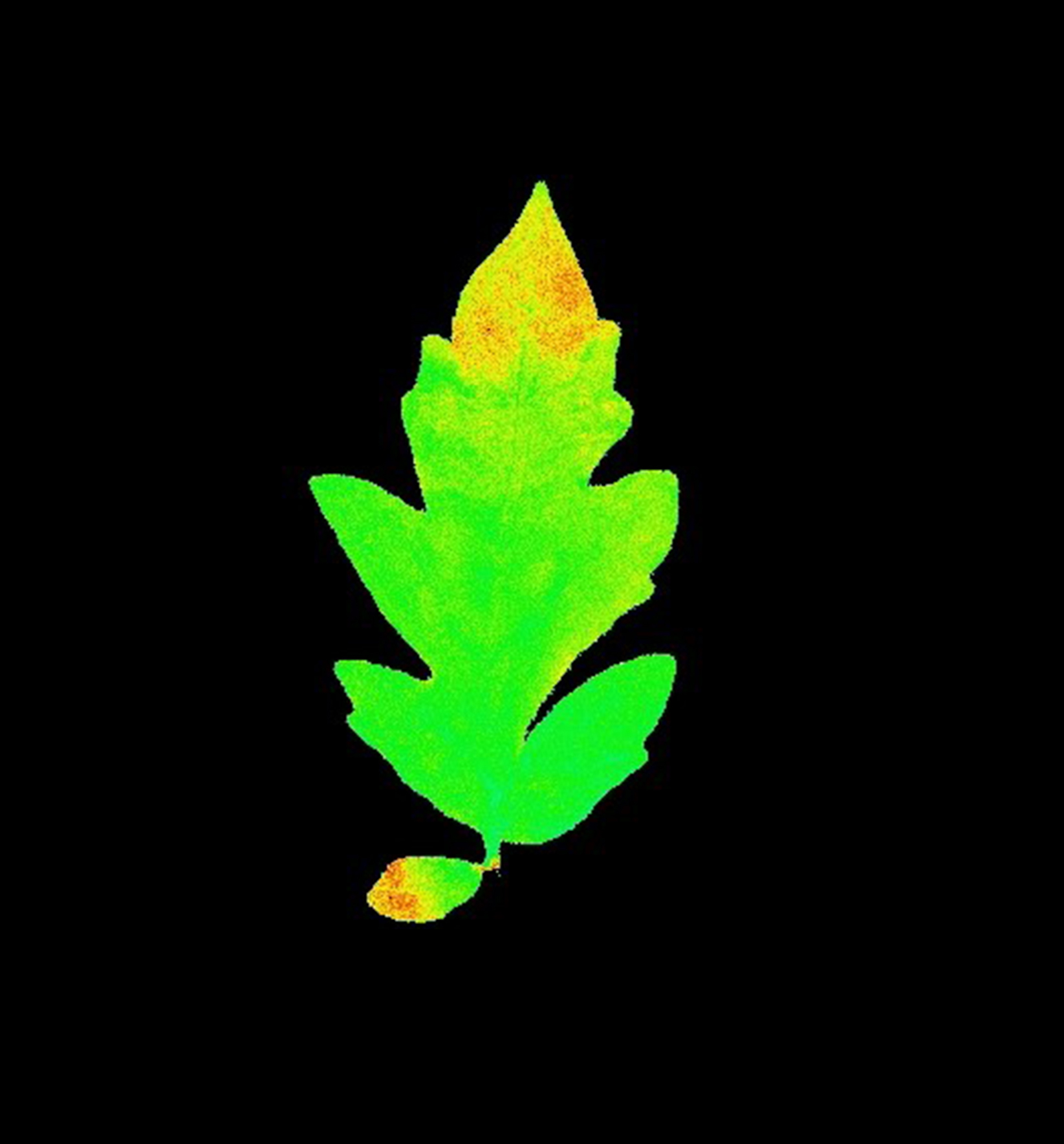

Supplement: Supplementary file 11 — Source data Fig. 2 [file 44318_2024_278_MOESM11_ESM.zip › Figure 2N/6_OE-BRAK#6 B.c.jpg]

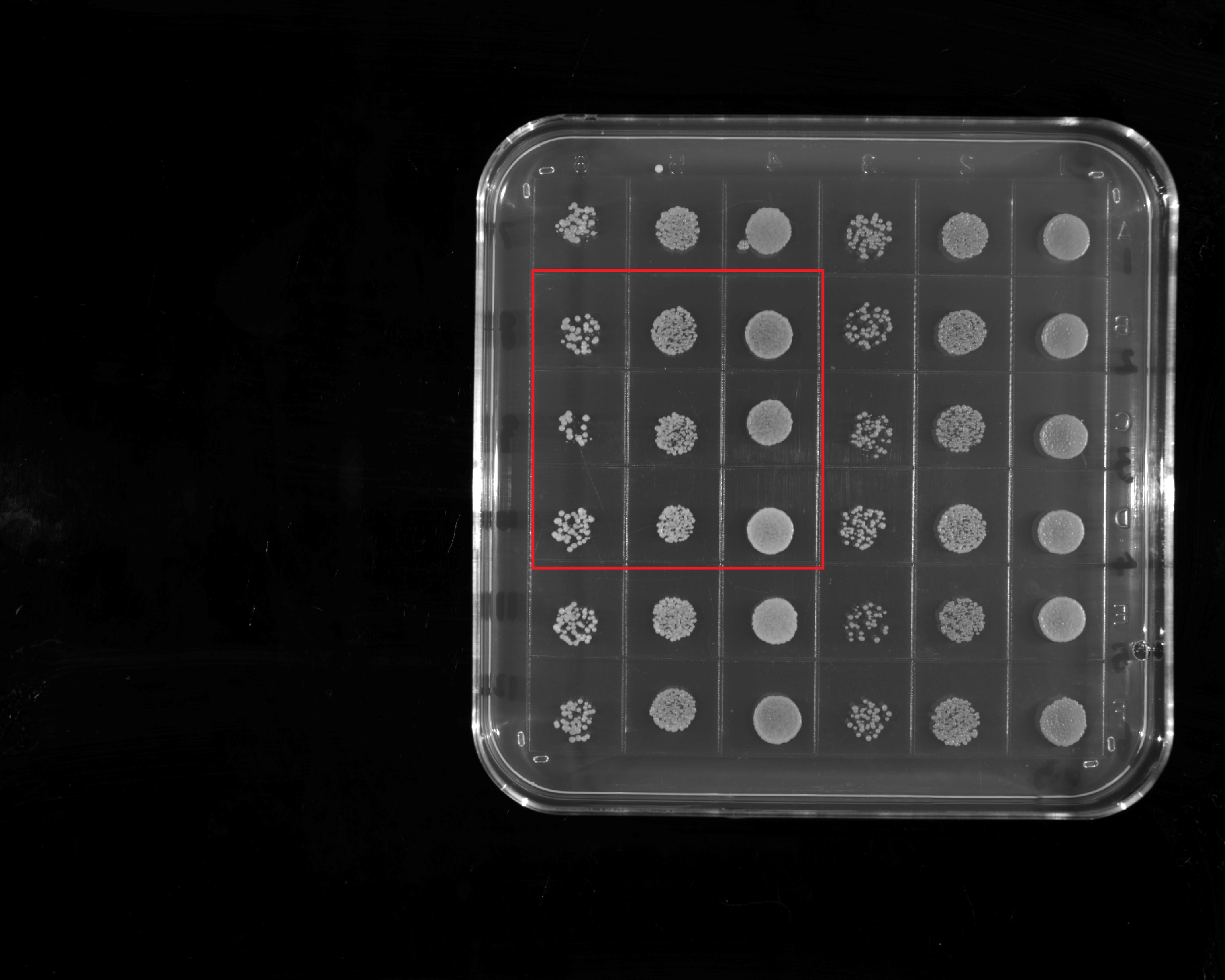

Supplement: Supplementary file 12 — Source data Fig. 3 [file 44318_2024_278_MOESM12_ESM.zip › Figure 3A/left panel.tif]

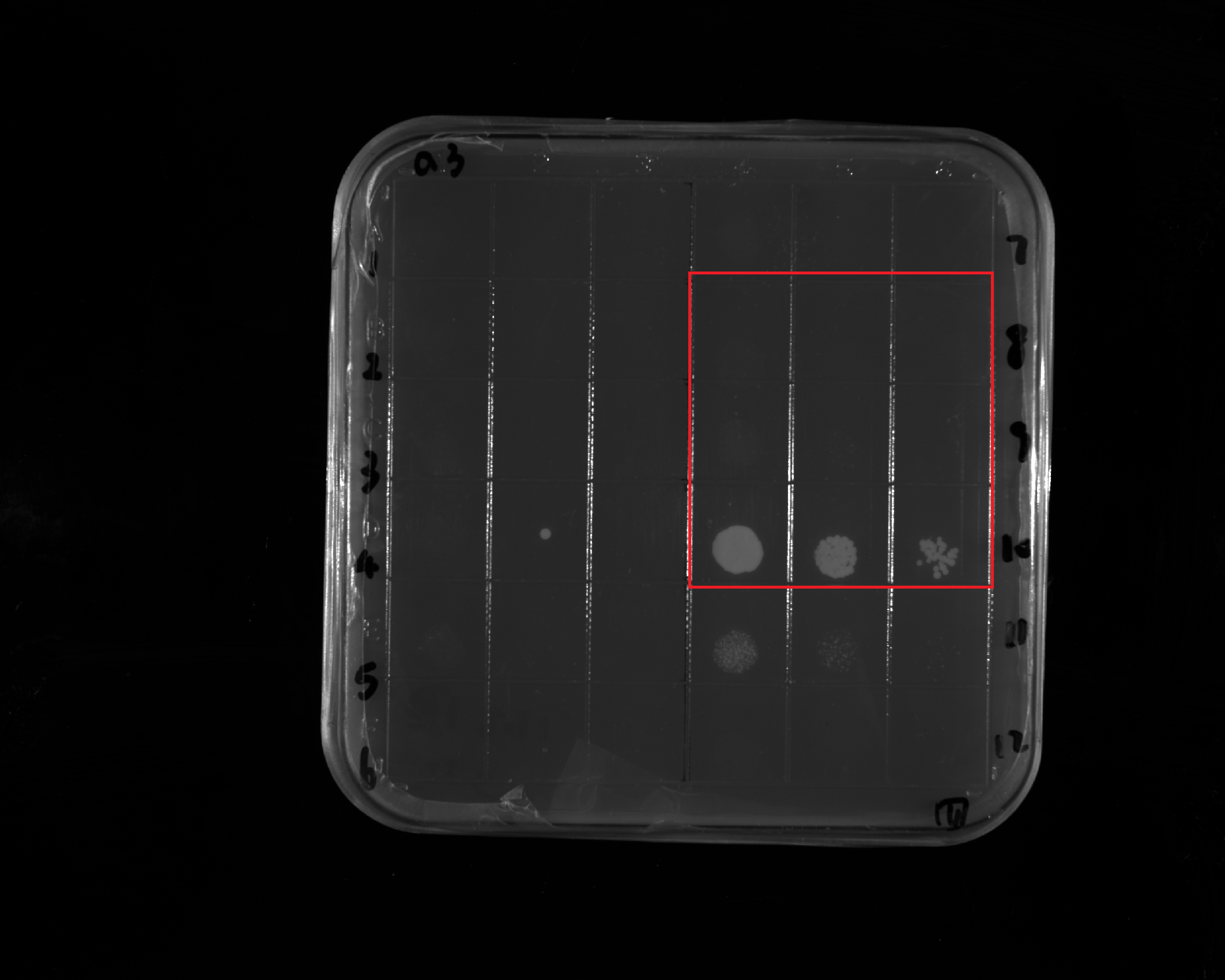

Supplement: Supplementary file 12 — Source data Fig. 3 [file 44318_2024_278_MOESM12_ESM.zip › Figure 3A/right panel.tif]

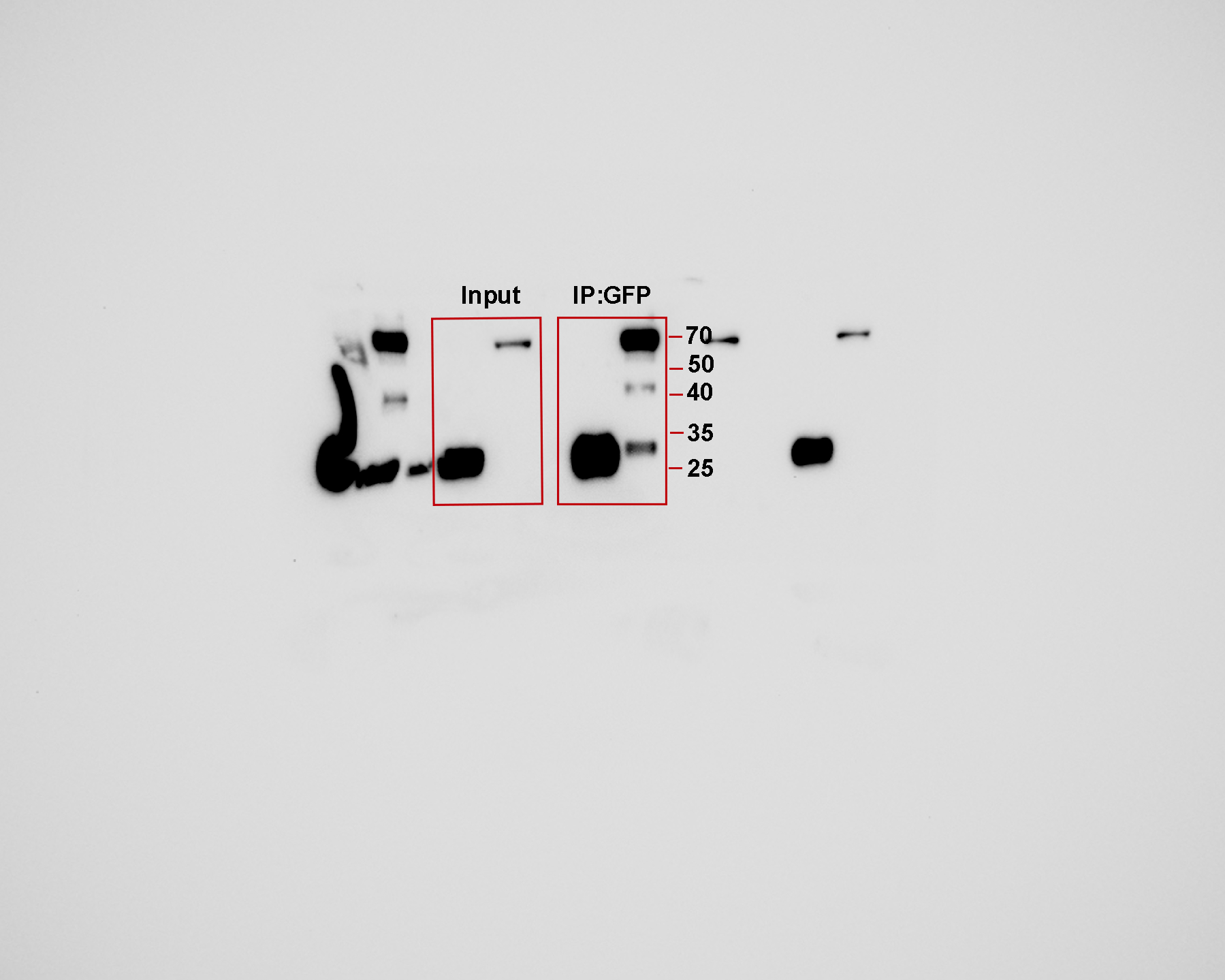

Supplement: Supplementary file 12 — Source data Fig. 3 [file 44318_2024_278_MOESM12_ESM.zip › Figure 3B/anti-GFP.tif]

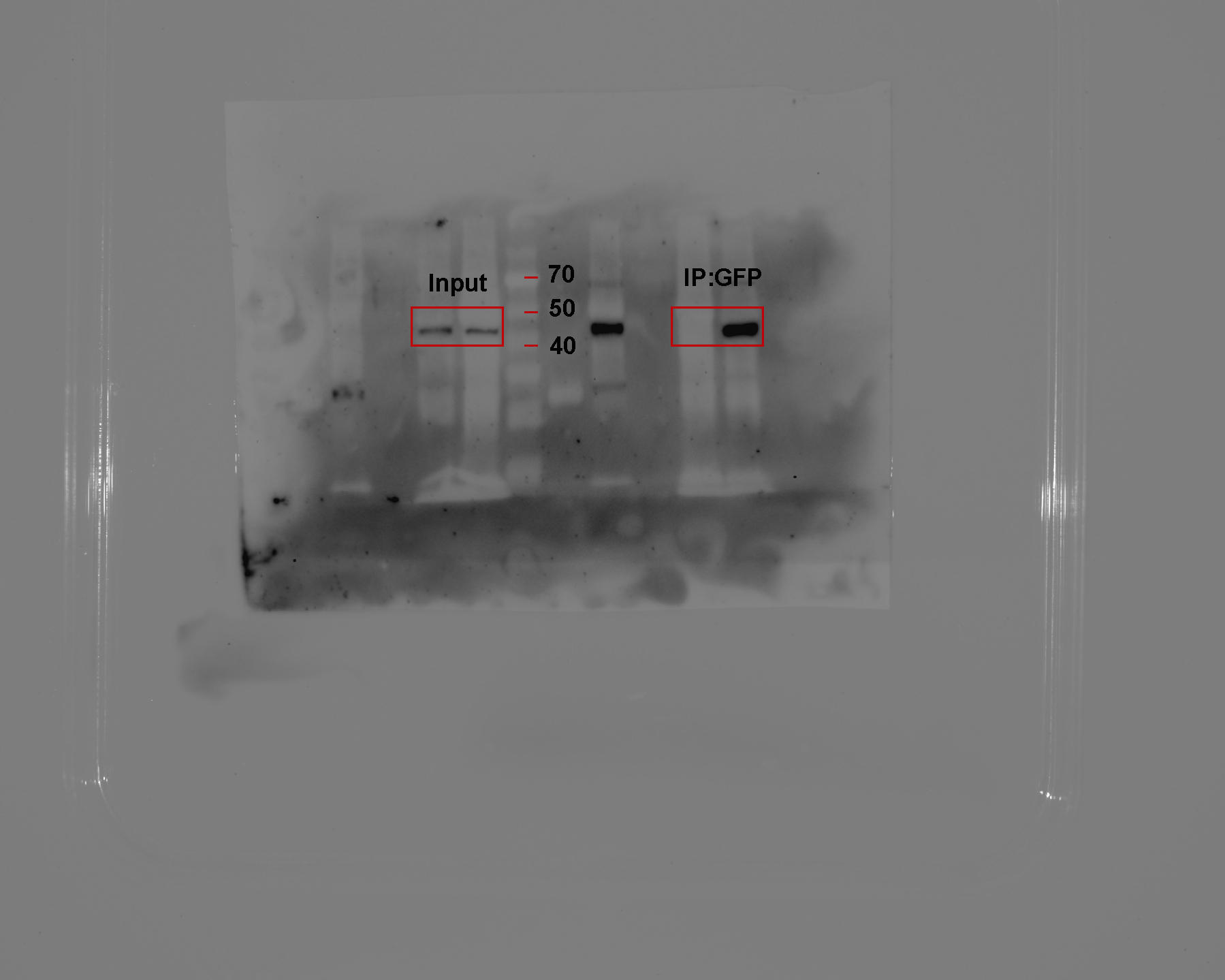

Supplement: Supplementary file 12 — Source data Fig. 3 [file 44318_2024_278_MOESM12_ESM.zip › Figure 3B/anti-HA.tif]

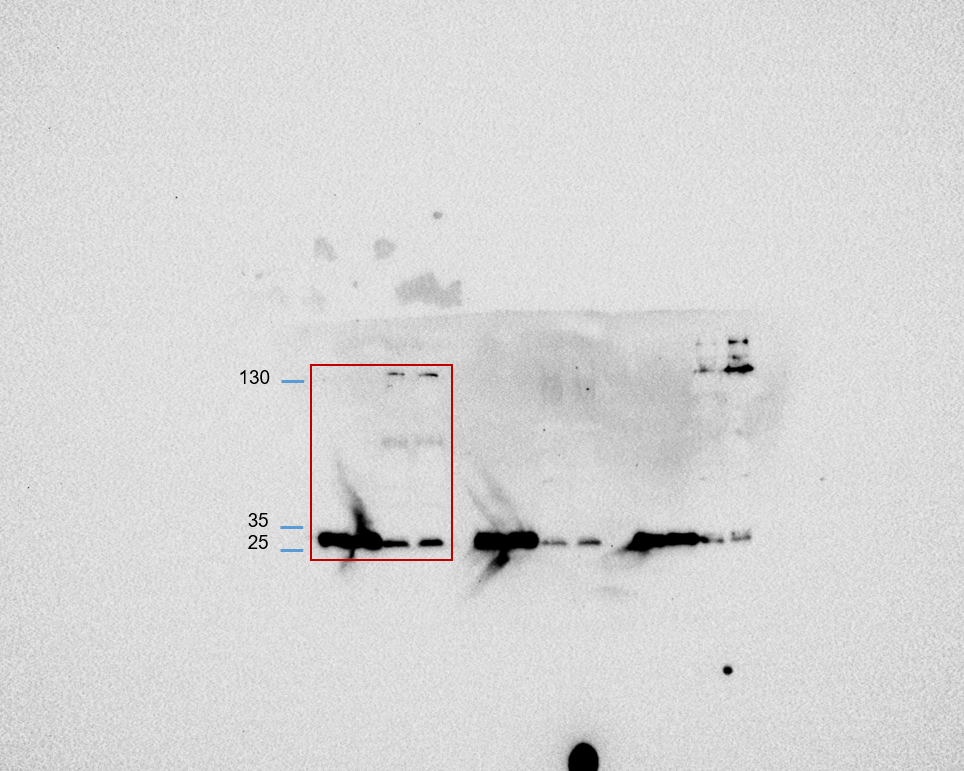

Supplement: Supplementary file 12 — Source data Fig. 3 [file 44318_2024_278_MOESM12_ESM.zip › Figure 3C/Input_anti_GFP.tif]

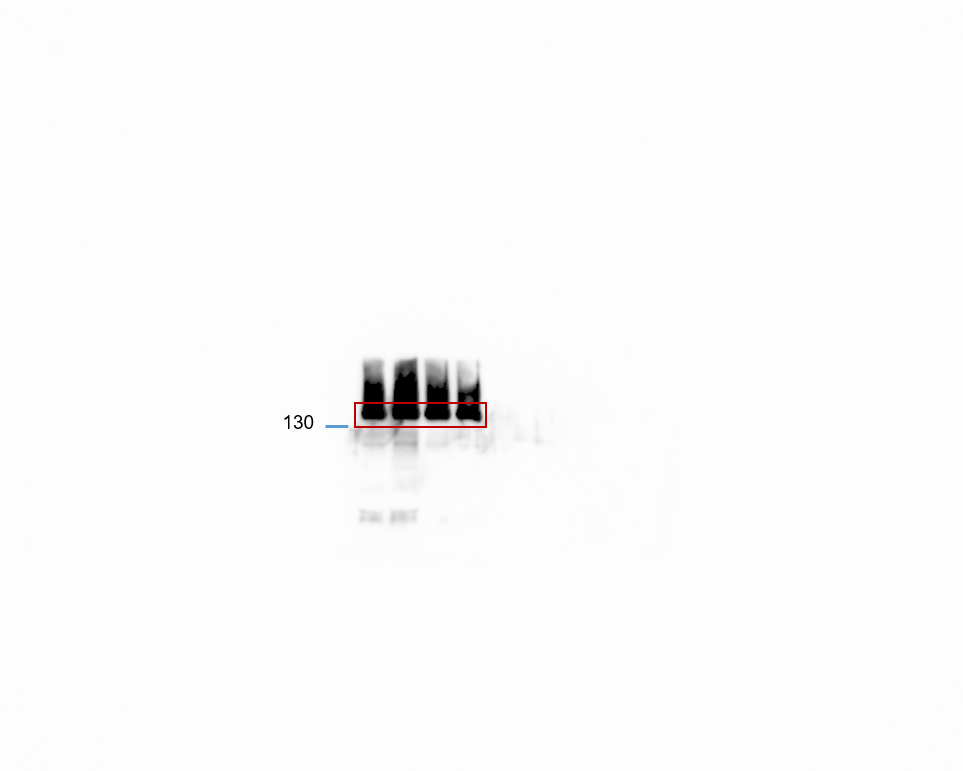

Supplement: Supplementary file 12 — Source data Fig. 3 [file 44318_2024_278_MOESM12_ESM.zip › Figure 3C/Input_anti_HA.tif]

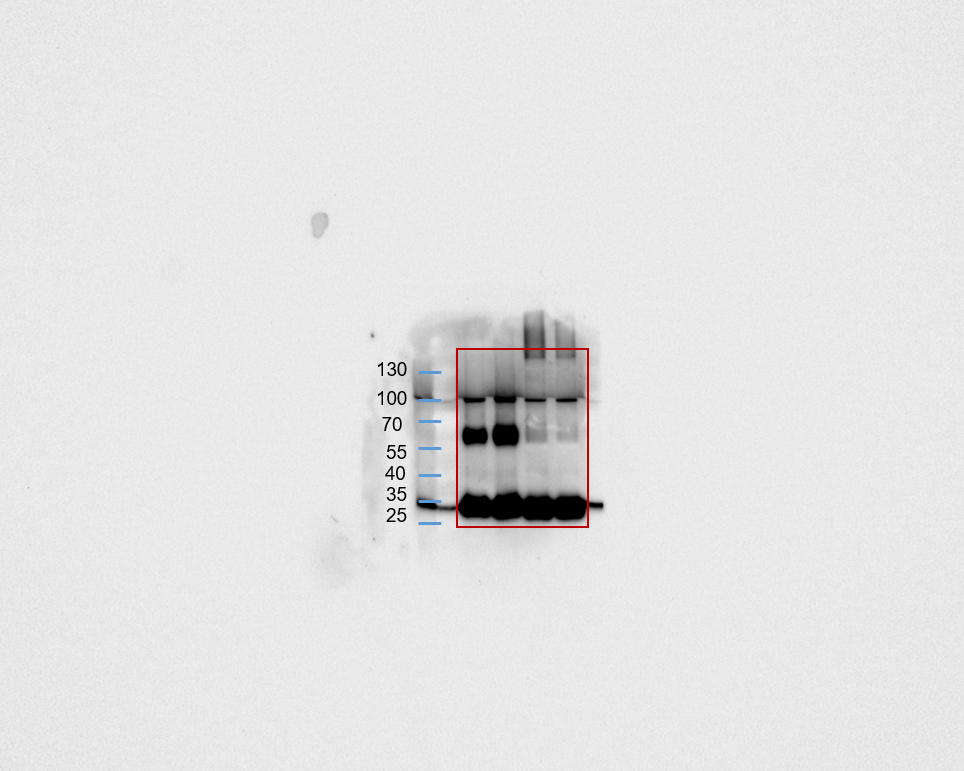

Supplement: Supplementary file 12 — Source data Fig. 3 [file 44318_2024_278_MOESM12_ESM.zip › Figure 3C/IP_anti_GFP.tif]

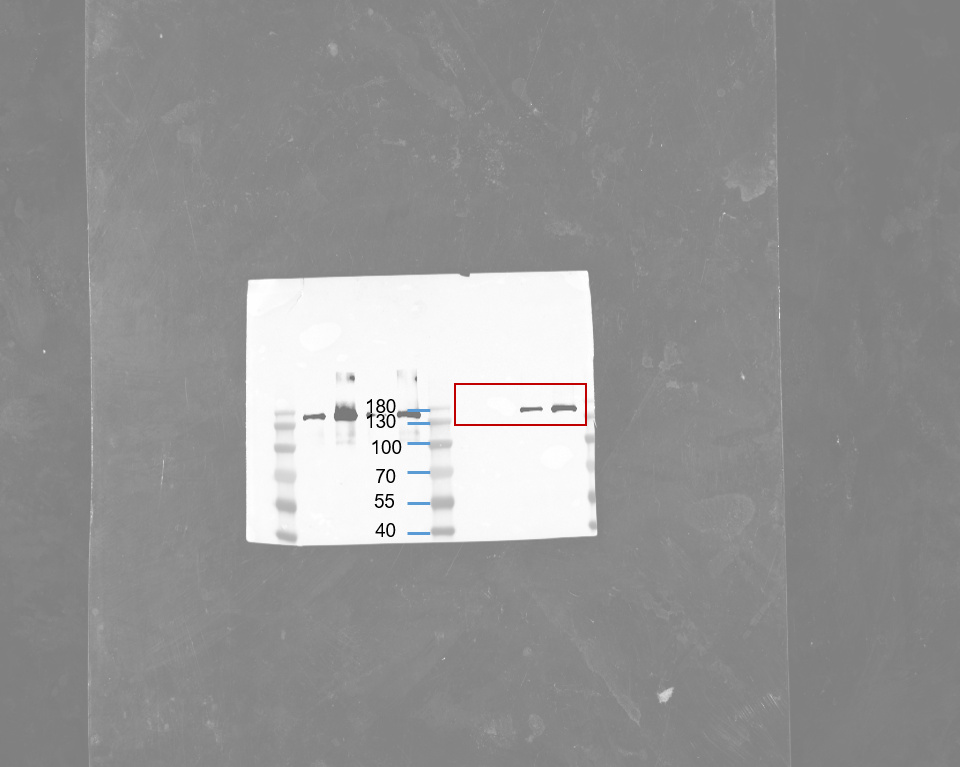

Supplement: Supplementary file 12 — Source data Fig. 3 [file 44318_2024_278_MOESM12_ESM.zip › Figure 3C/IP_anti_HA.tif]

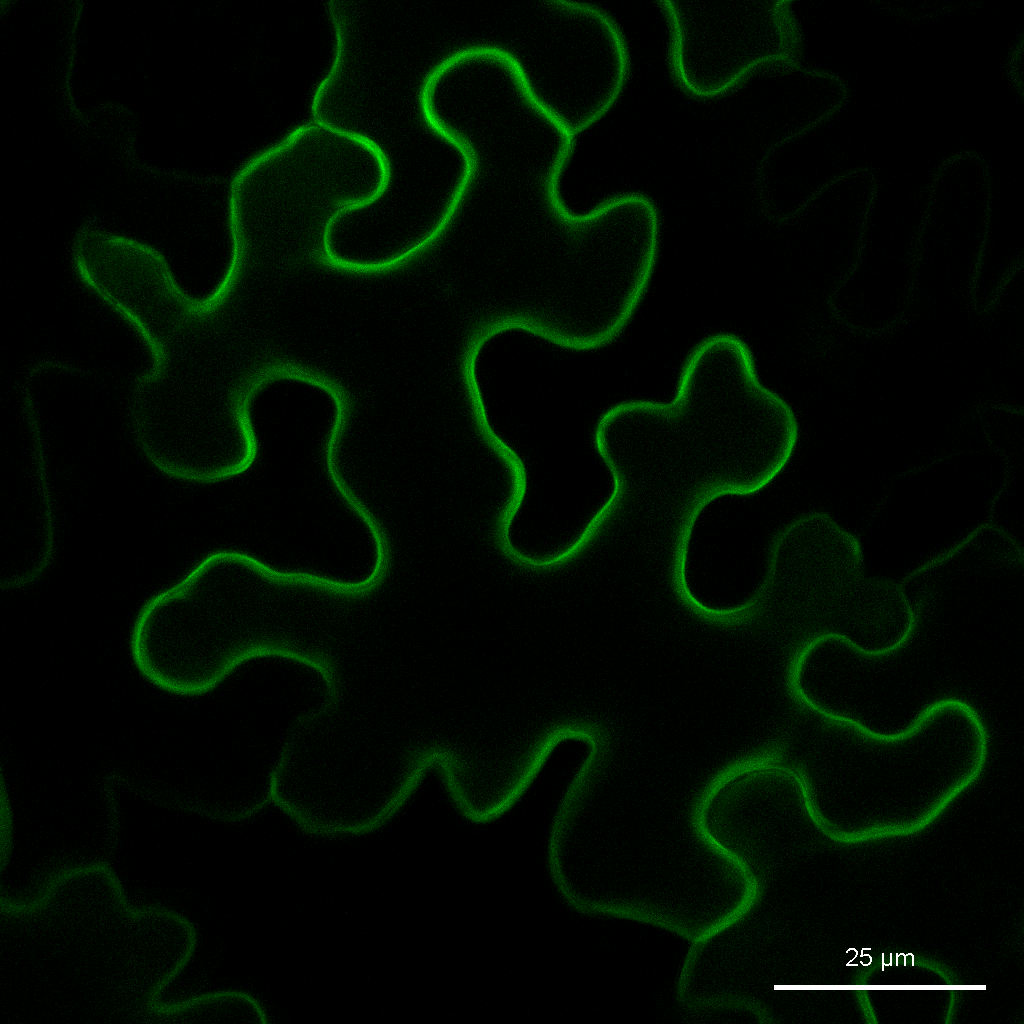

Supplement: Supplementary file 12 — Source data Fig. 3 [file 44318_2024_278_MOESM12_ESM.zip › Figure 3D/1.tif]

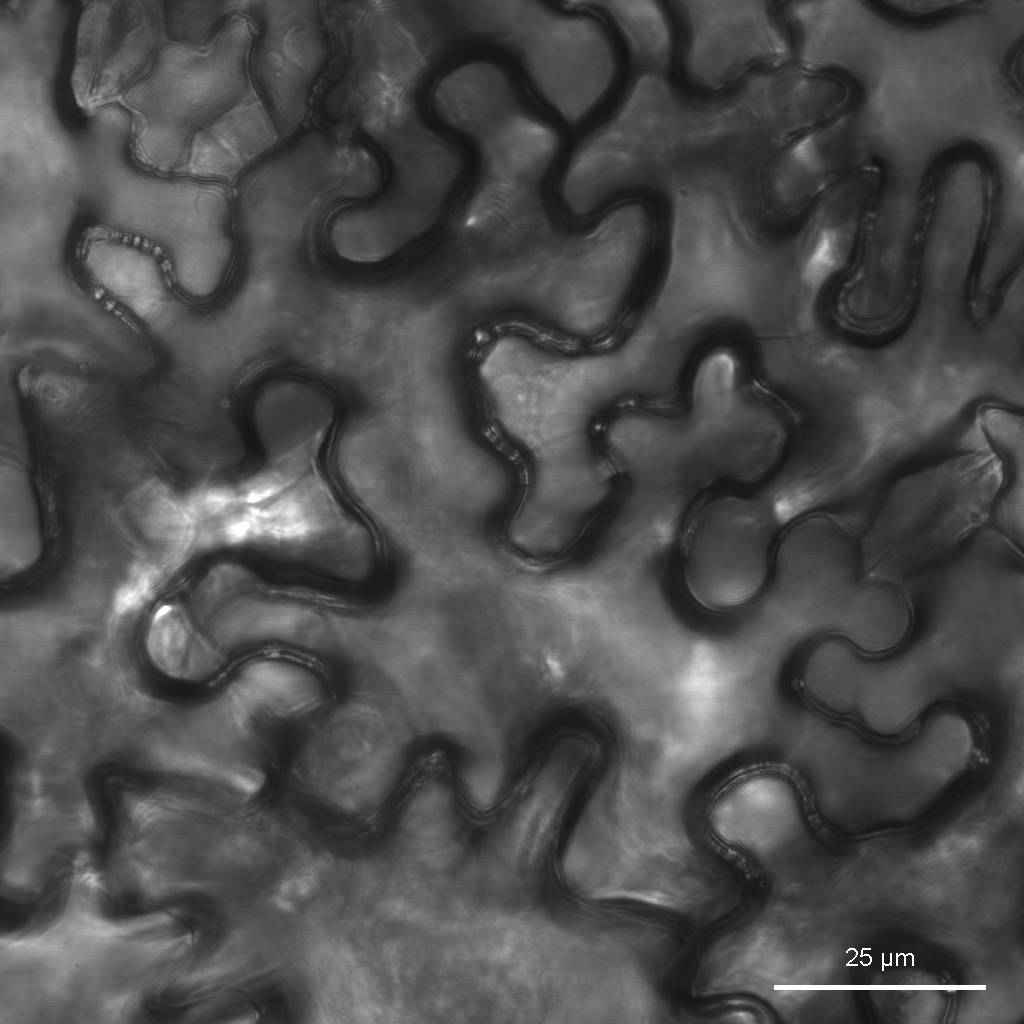

Supplement: Supplementary file 12 — Source data Fig. 3 [file 44318_2024_278_MOESM12_ESM.zip › Figure 3D/2.tif]

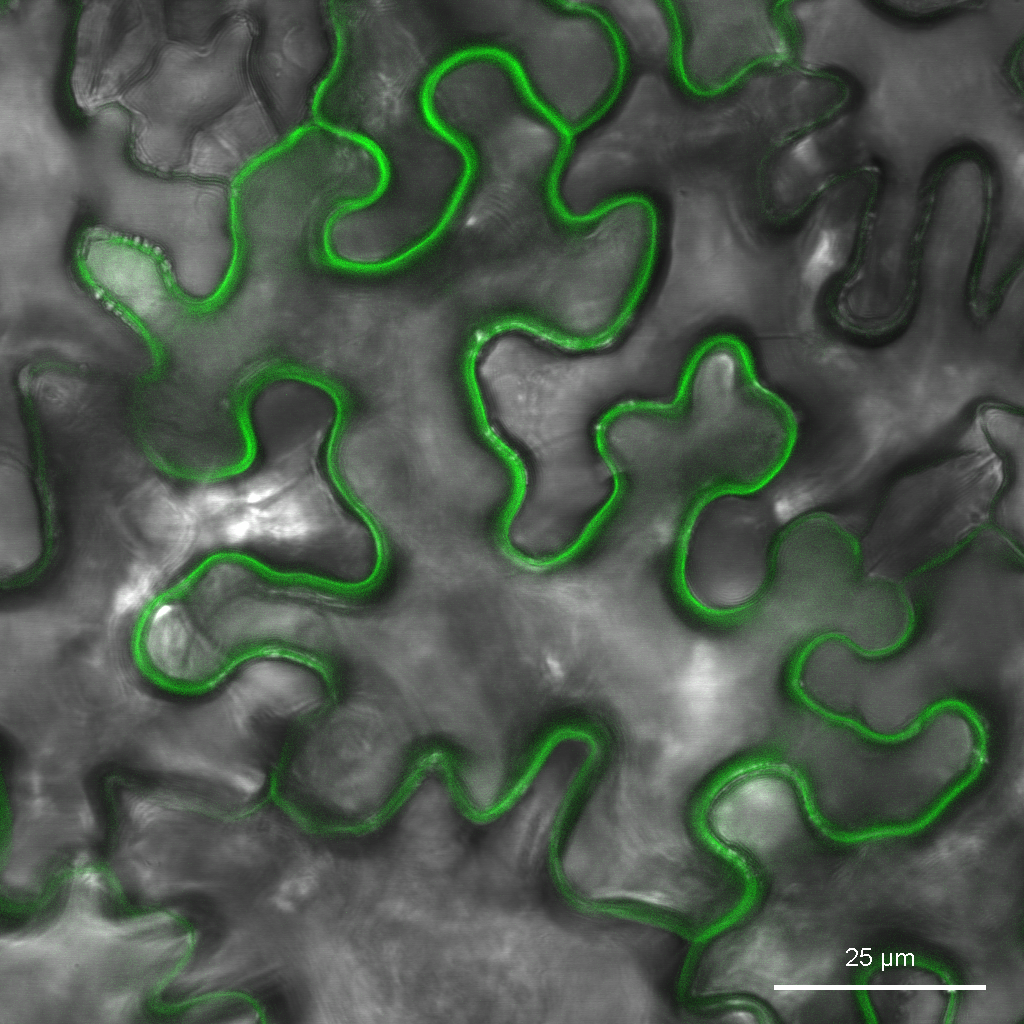

Supplement: Supplementary file 12 — Source data Fig. 3 [file 44318_2024_278_MOESM12_ESM.zip › Figure 3D/3.tif]

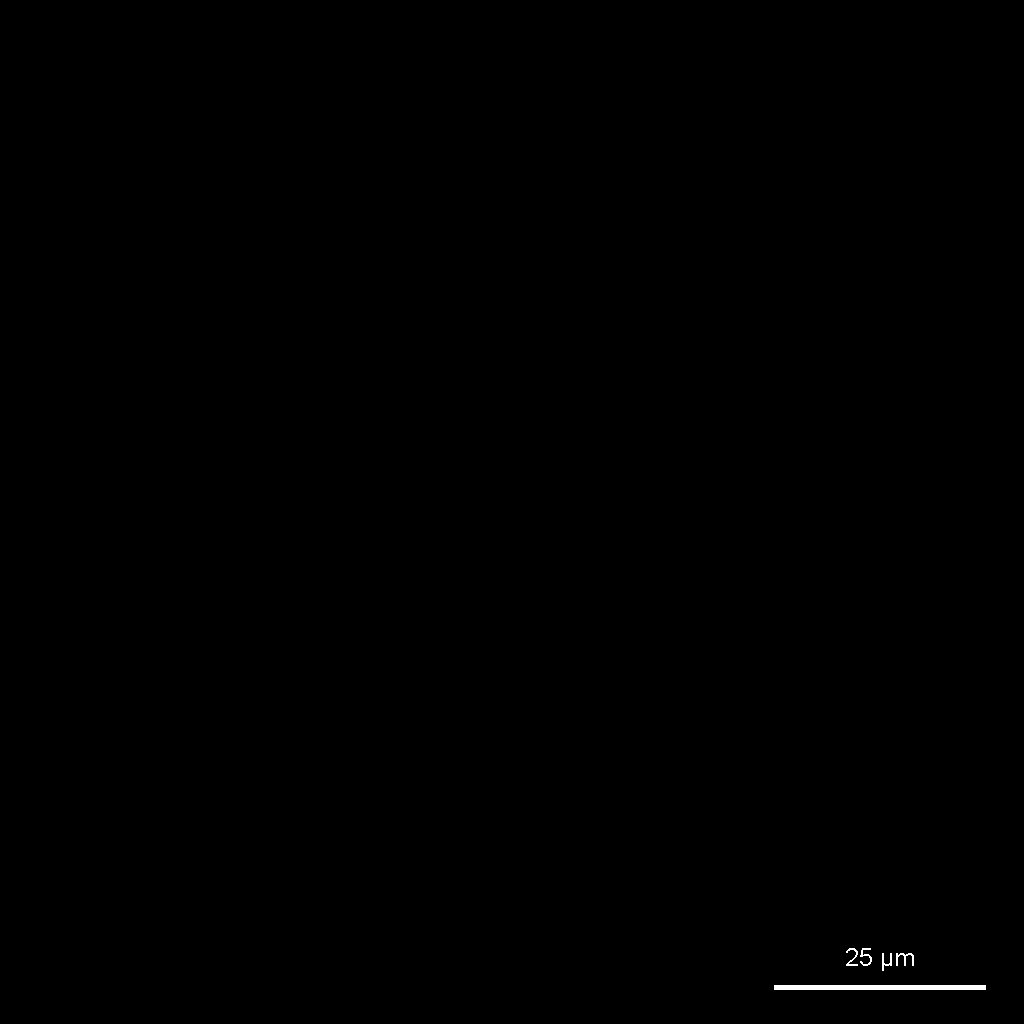

Supplement: Supplementary file 12 — Source data Fig. 3 [file 44318_2024_278_MOESM12_ESM.zip › Figure 3D/4.tif]

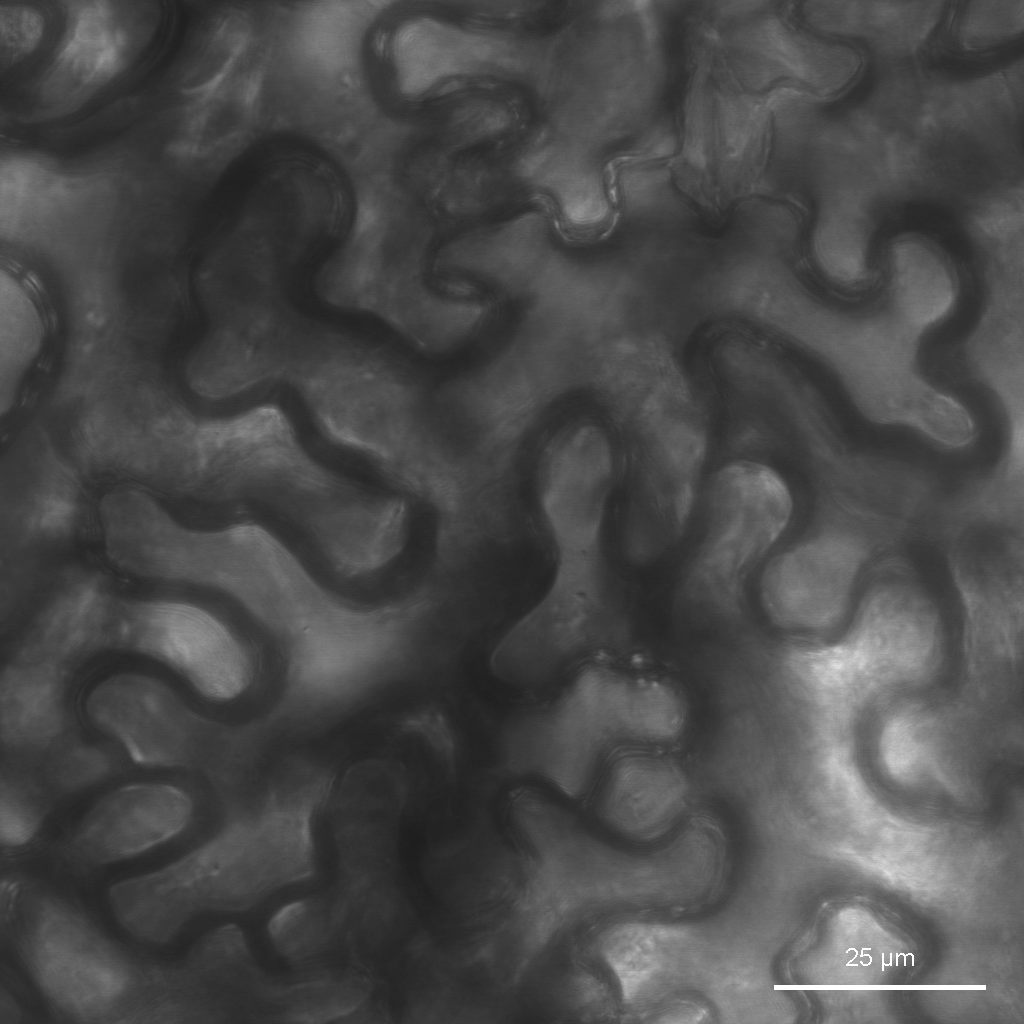

Supplement: Supplementary file 12 — Source data Fig. 3 [file 44318_2024_278_MOESM12_ESM.zip › Figure 3D/5.tif]

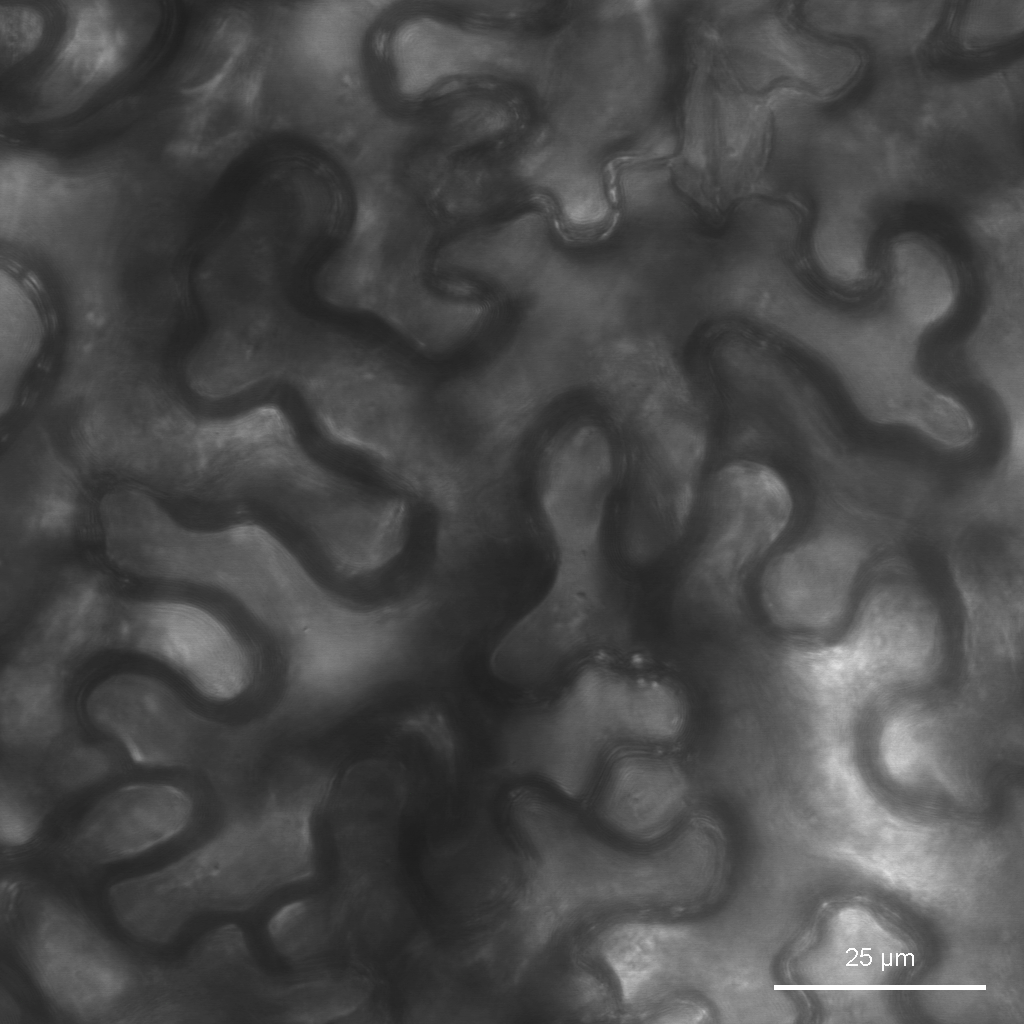

Supplement: Supplementary file 12 — Source data Fig. 3 [file 44318_2024_278_MOESM12_ESM.zip › Figure 3D/6.tif]

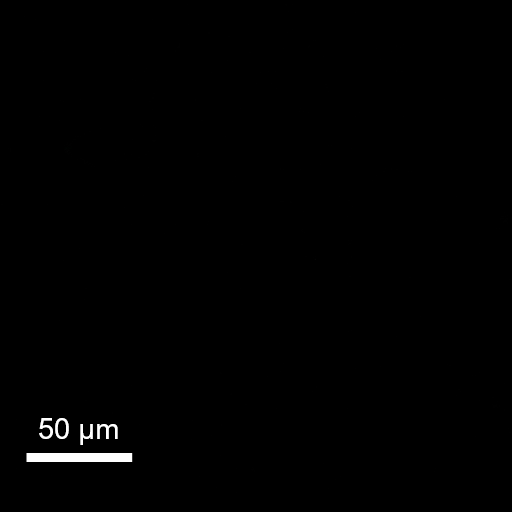

Supplement: Supplementary file 12 — Source data Fig. 3 [file 44318_2024_278_MOESM12_ESM.zip › Figure 3D/7.tif]

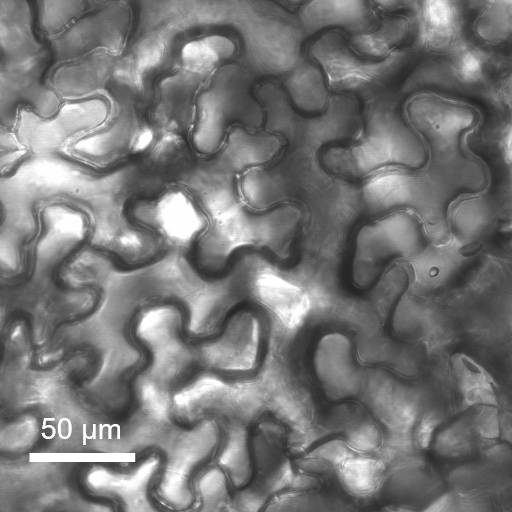

Supplement: Supplementary file 12 — Source data Fig. 3 [file 44318_2024_278_MOESM12_ESM.zip › Figure 3D/8.tif]

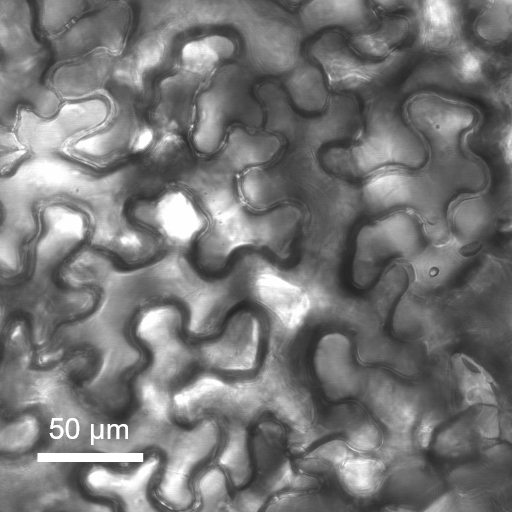

Supplement: Supplementary file 12 — Source data Fig. 3 [file 44318_2024_278_MOESM12_ESM.zip › Figure 3D/9.tif]

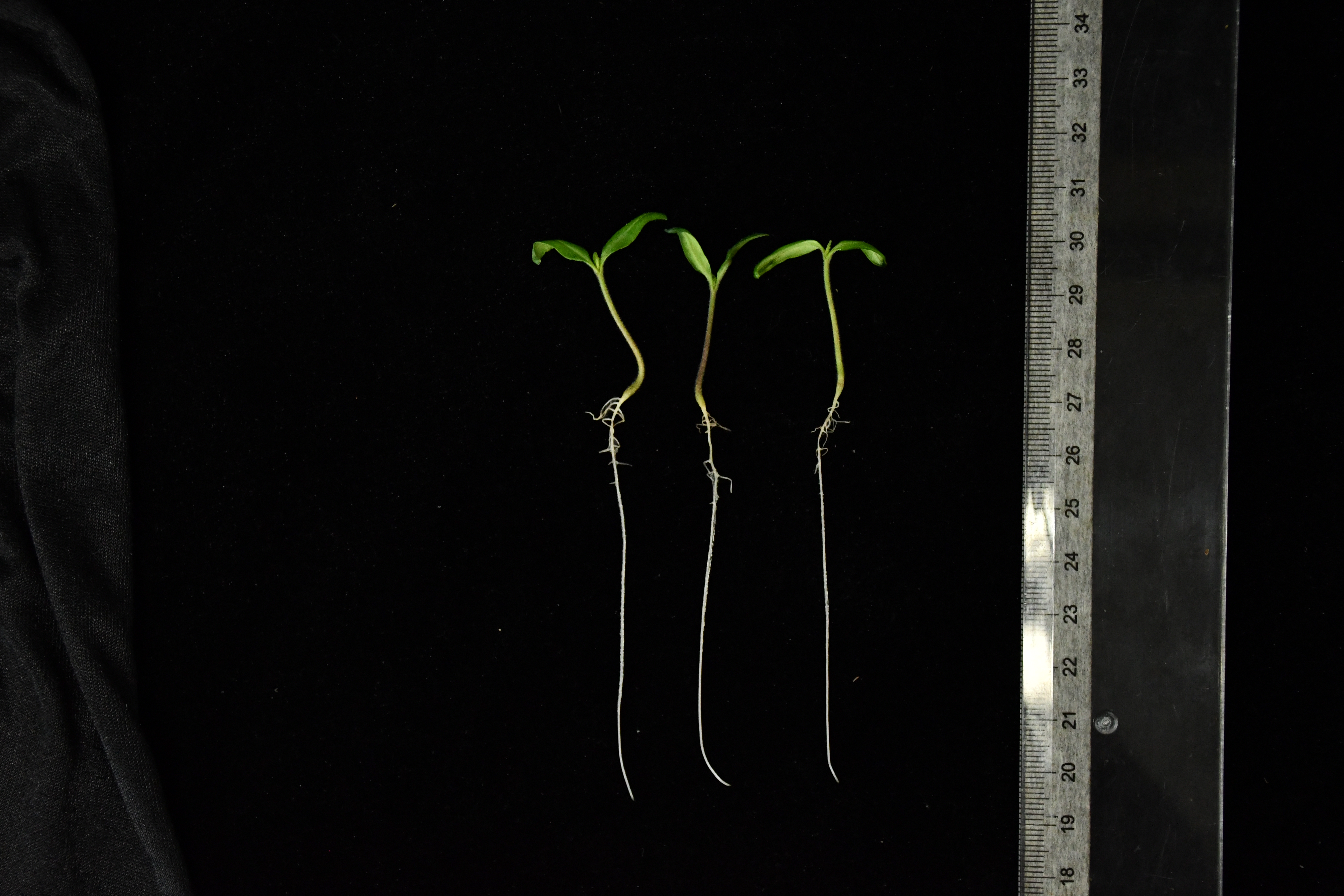

Supplement: Supplementary file 13 — Source data Fig. 4 [file 44318_2024_278_MOESM13_ESM.zip › Figure 4A/1. WT H2O.JPG]

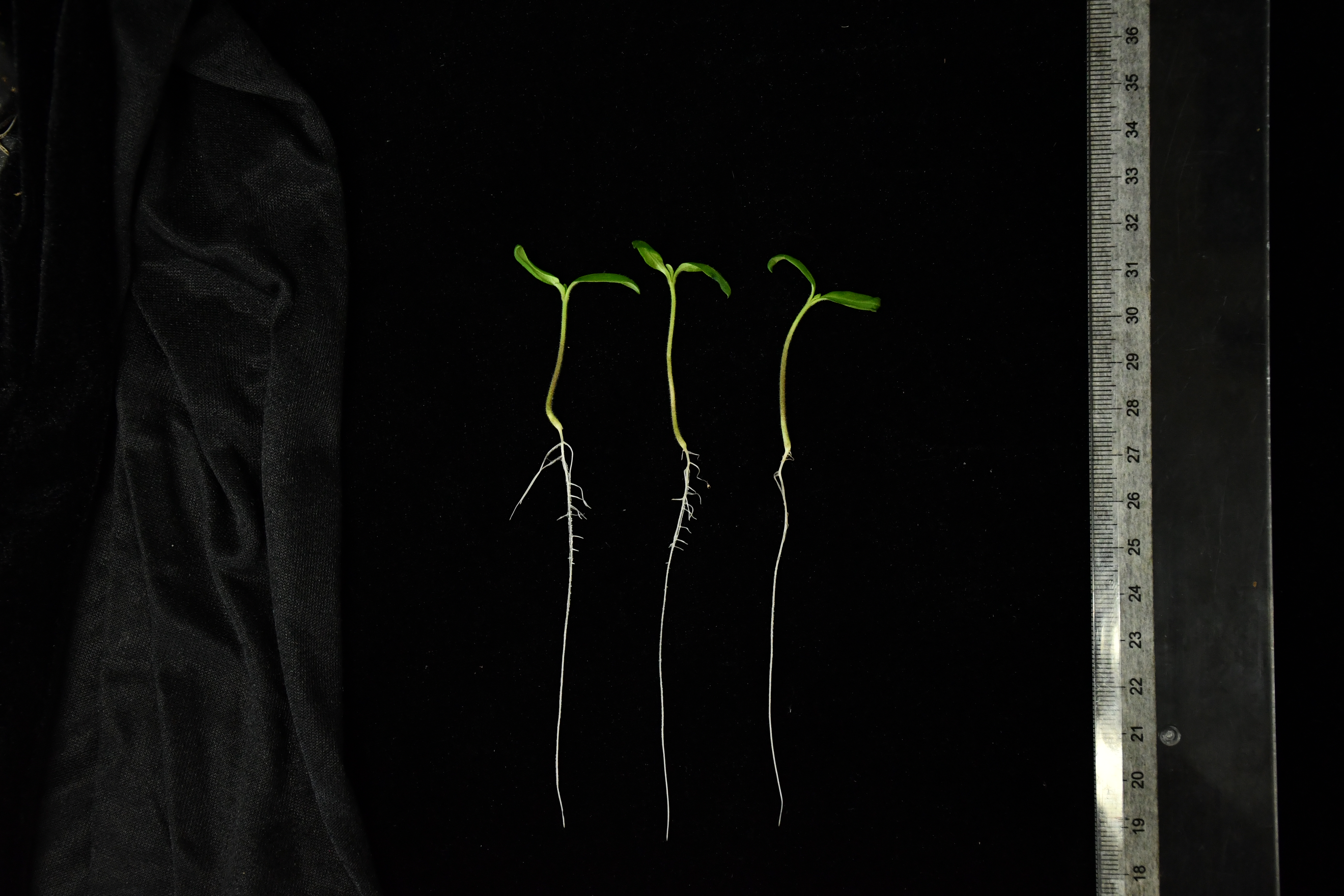

Supplement: Supplementary file 13 — Source data Fig. 4 [file 44318_2024_278_MOESM13_ESM.zip › Figure 4A/2. WT PSK.JPG]

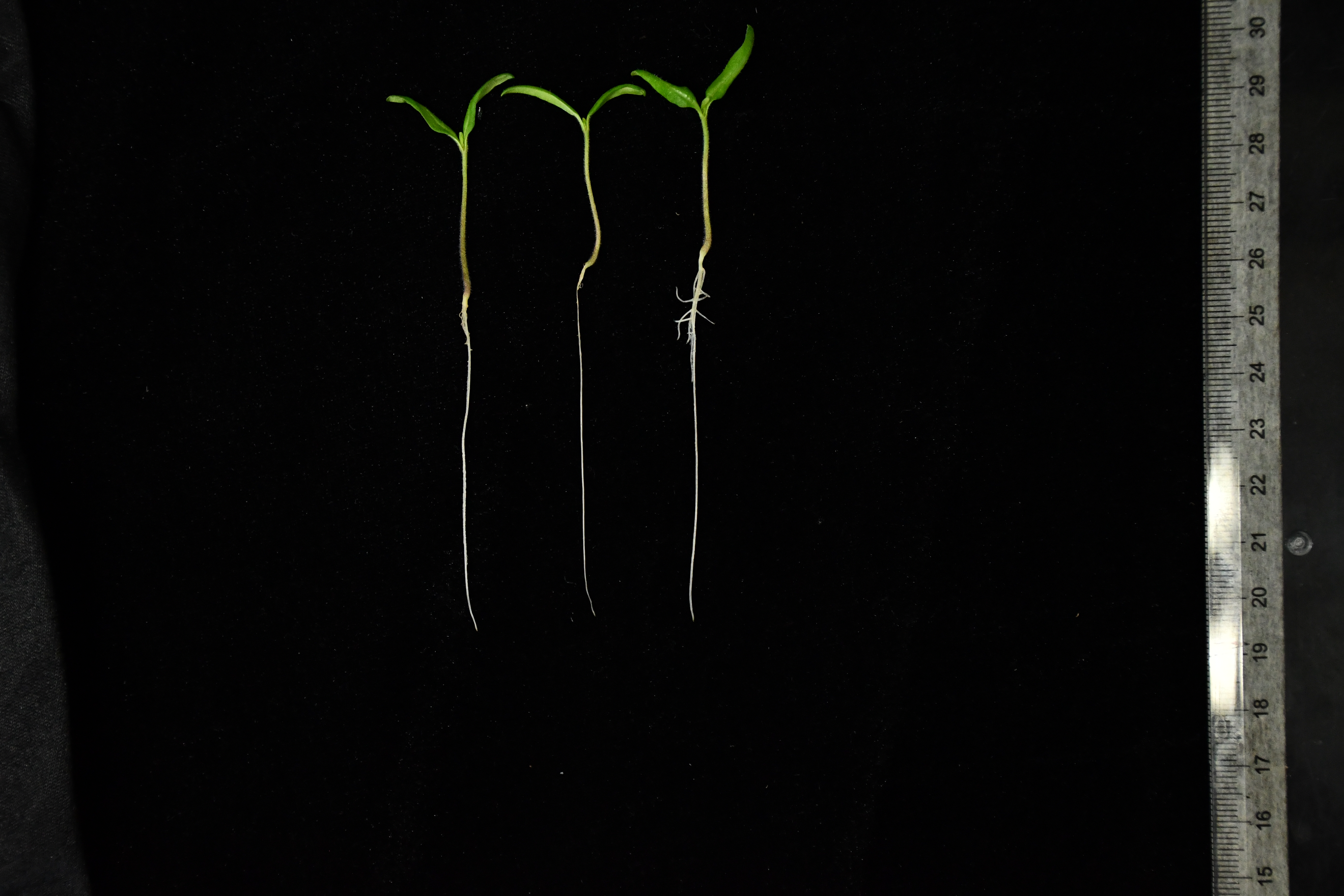

Supplement: Supplementary file 13 — Source data Fig. 4 [file 44318_2024_278_MOESM13_ESM.zip › Figure 4A/3. pskr1 H2O.JPG]

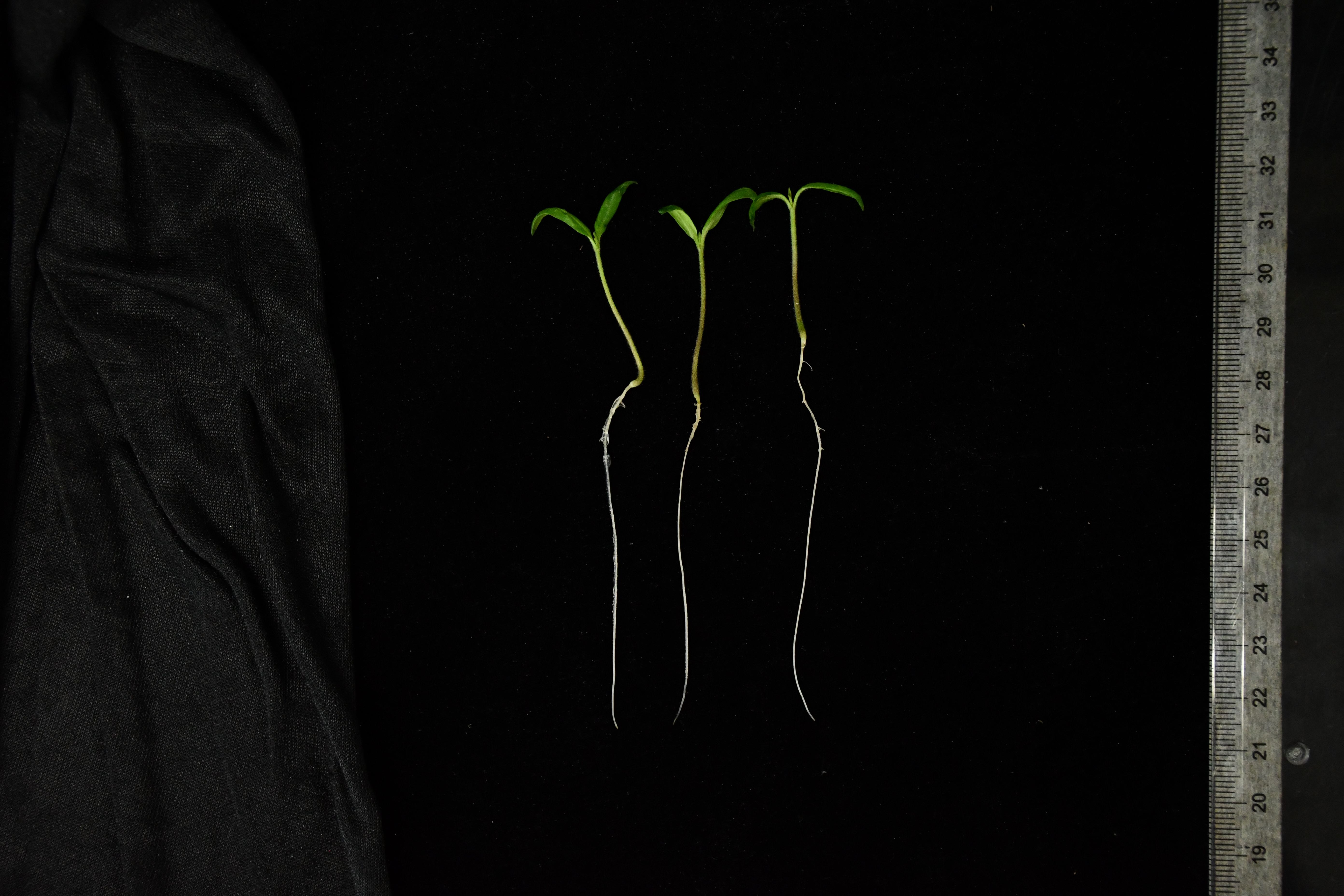

Supplement: Supplementary file 13 — Source data Fig. 4 [file 44318_2024_278_MOESM13_ESM.zip › Figure 4A/4. pskr1 PSK.JPG]

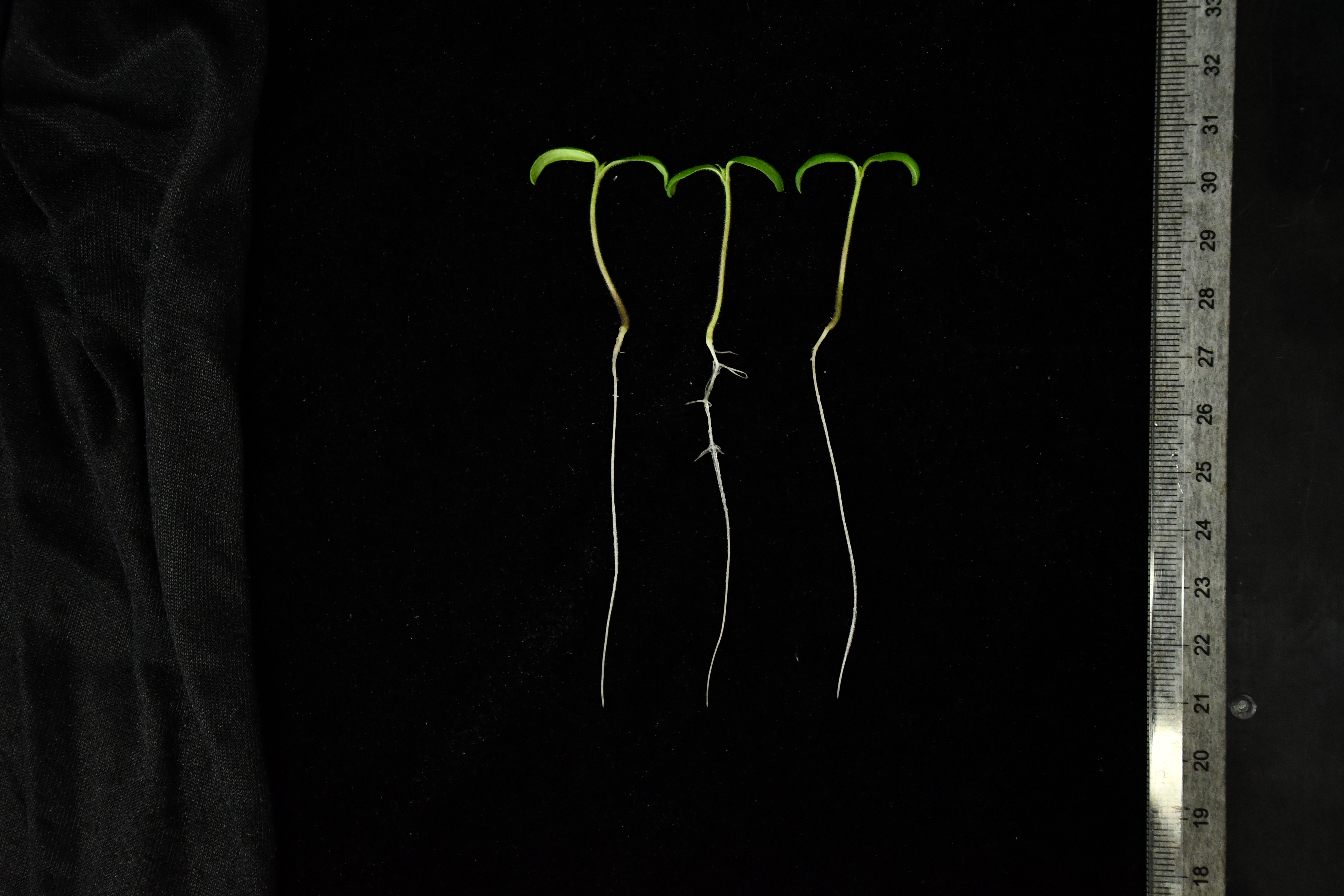

Supplement: Supplementary file 13 — Source data Fig. 4 [file 44318_2024_278_MOESM13_ESM.zip › Figure 4A/5. brak H2O.JPG]

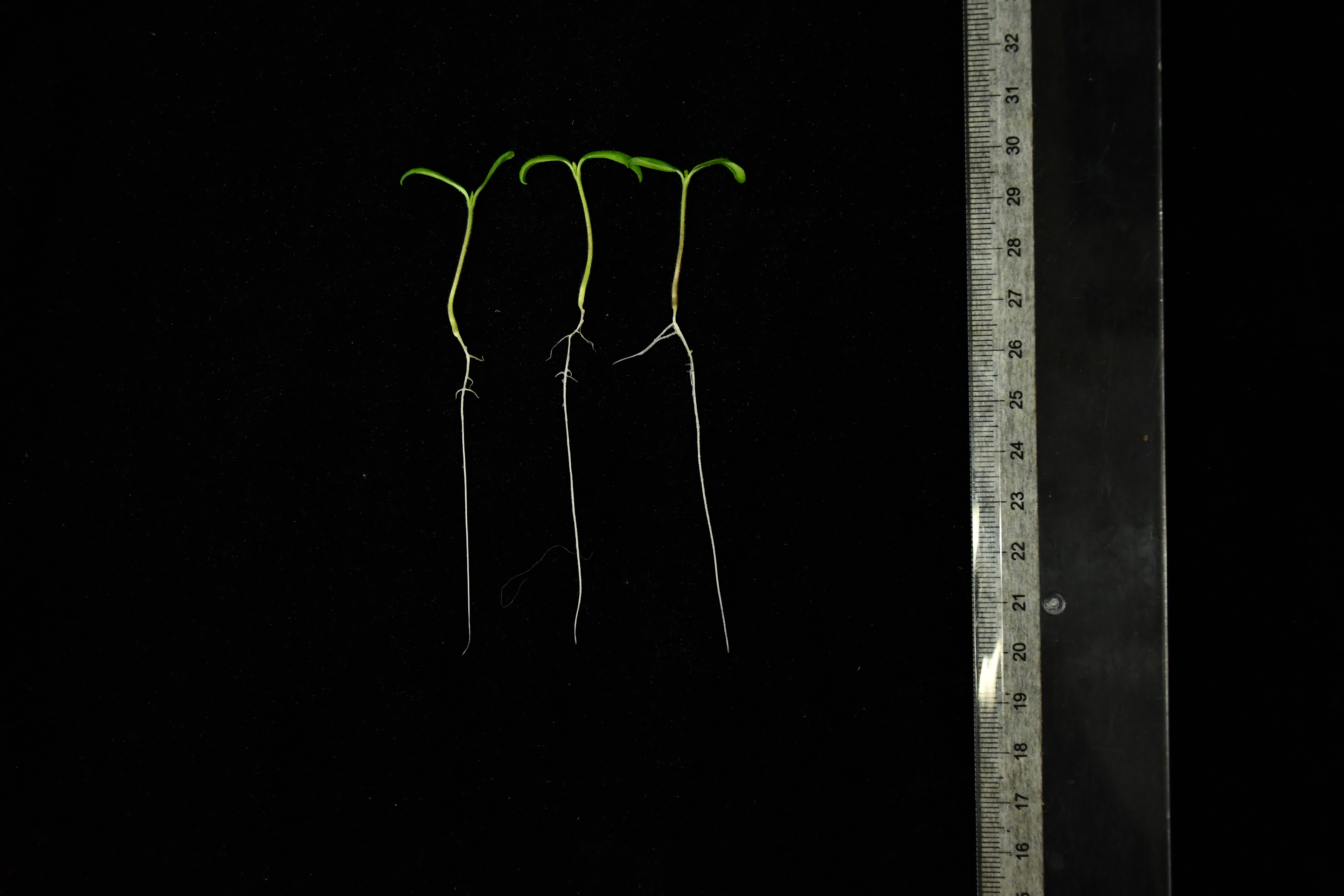

Supplement: Supplementary file 13 — Source data Fig. 4 [file 44318_2024_278_MOESM13_ESM.zip › Figure 4A/6. brak PSK.JPG]

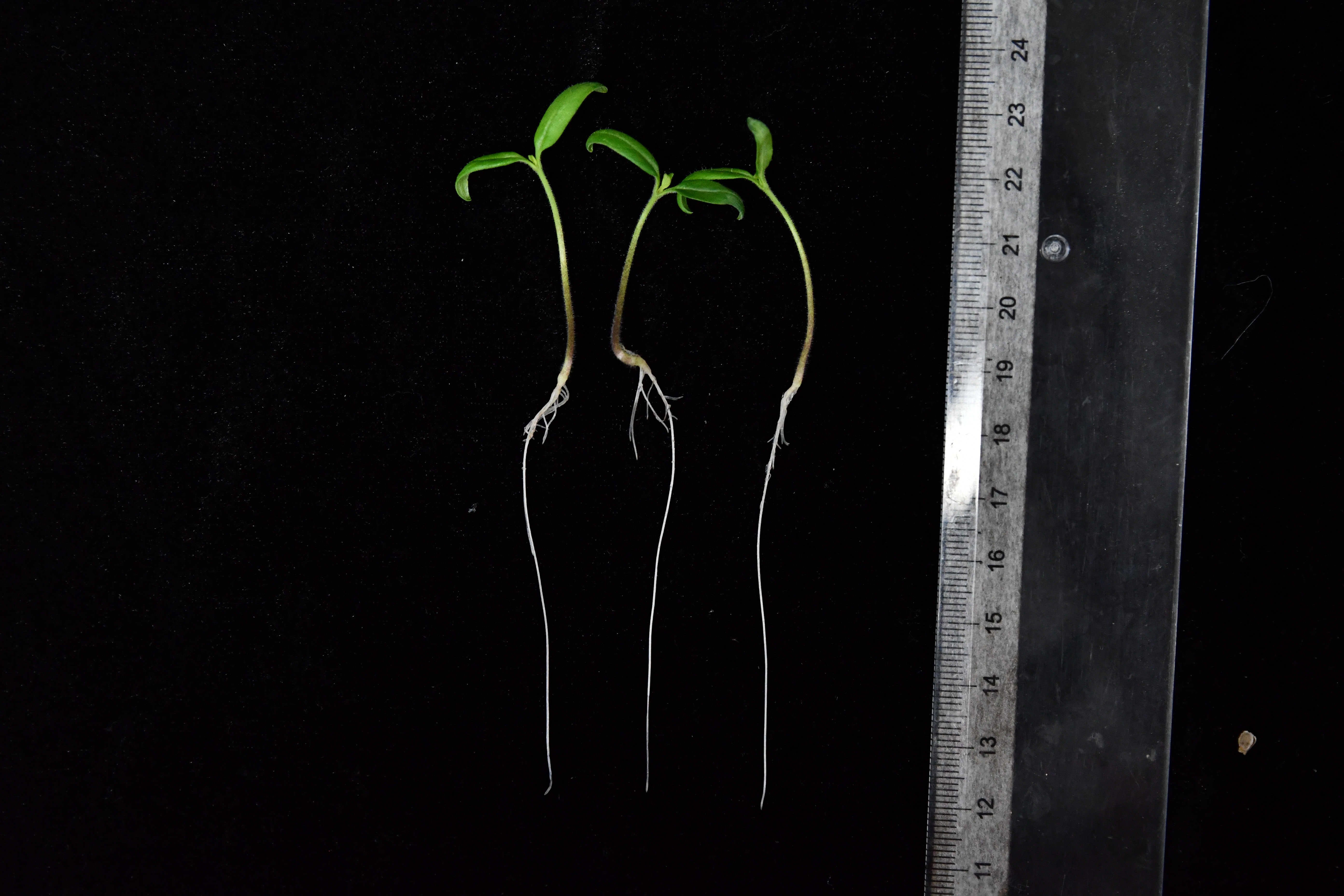

Supplement: Supplementary file 13 — Source data Fig. 4 [file 44318_2024_278_MOESM13_ESM.zip › Figure 4A/7. pskr1 brak H2O.JPG]

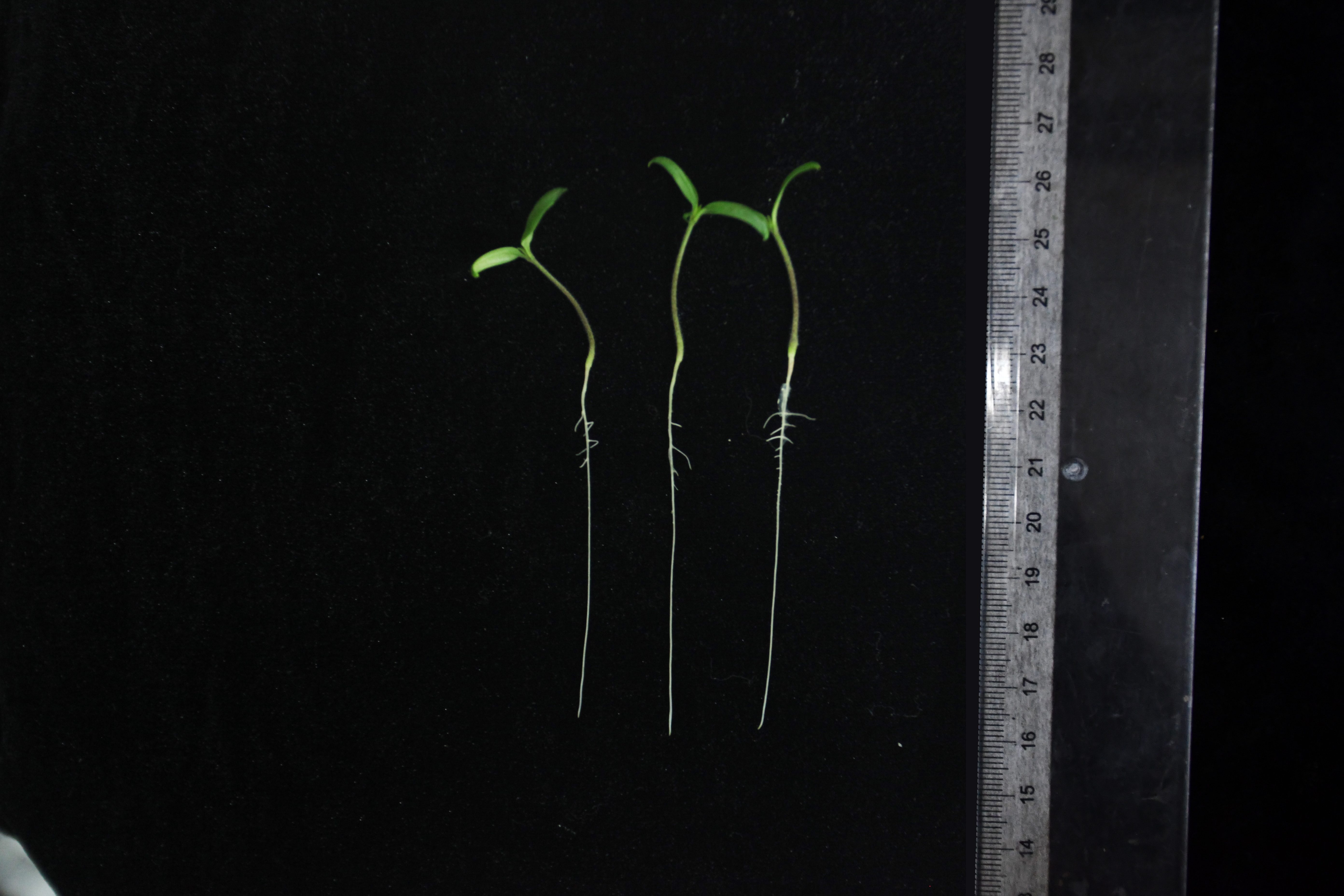

Supplement: Supplementary file 13 — Source data Fig. 4 [file 44318_2024_278_MOESM13_ESM.zip › Figure 4A/8. pskr1 brak PSK.jpg]

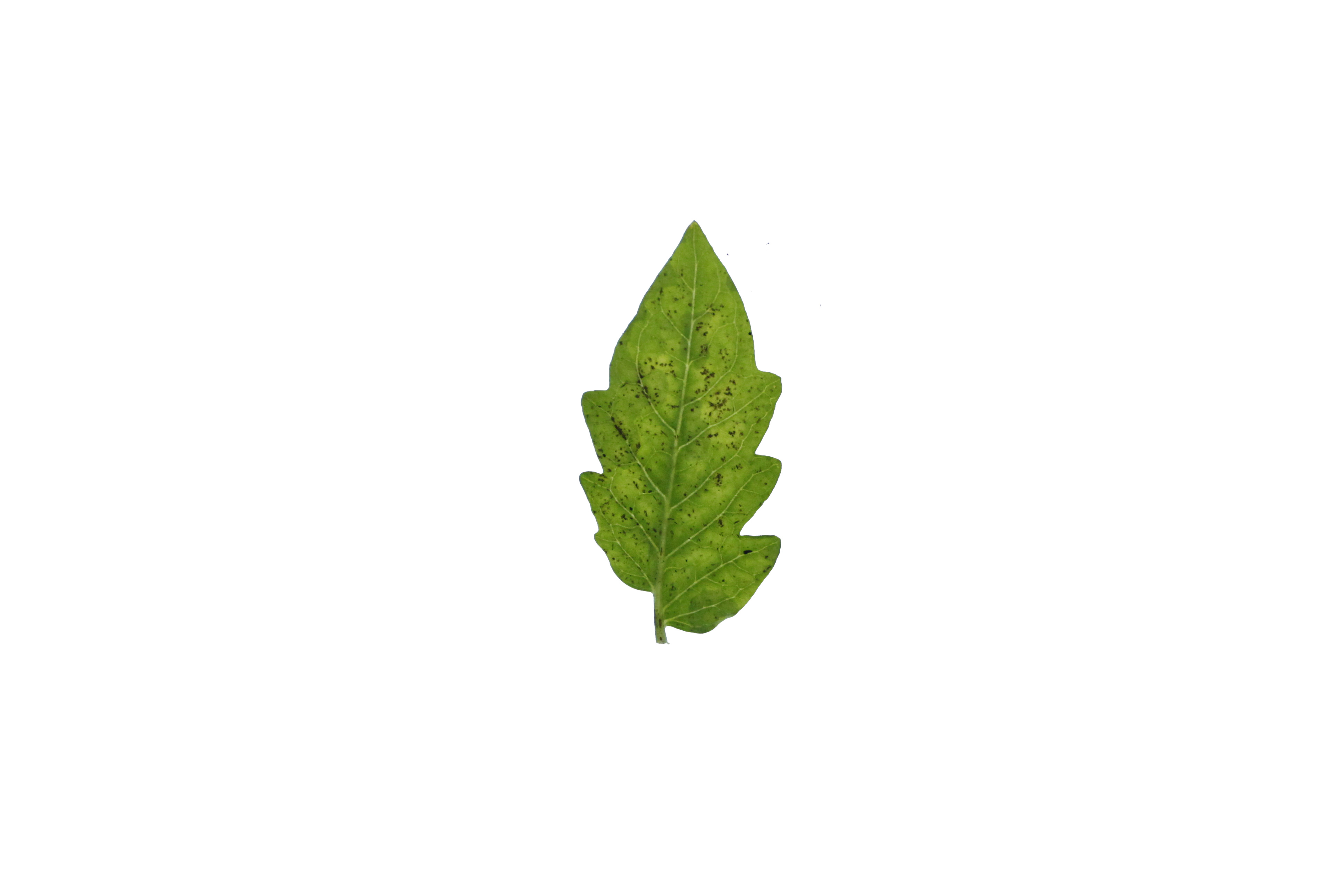

Supplement: Supplementary file 13 — Source data Fig. 4 [file 44318_2024_278_MOESM13_ESM.zip › figure 4C/10_B.c pskr1 brak PSK.jpg]

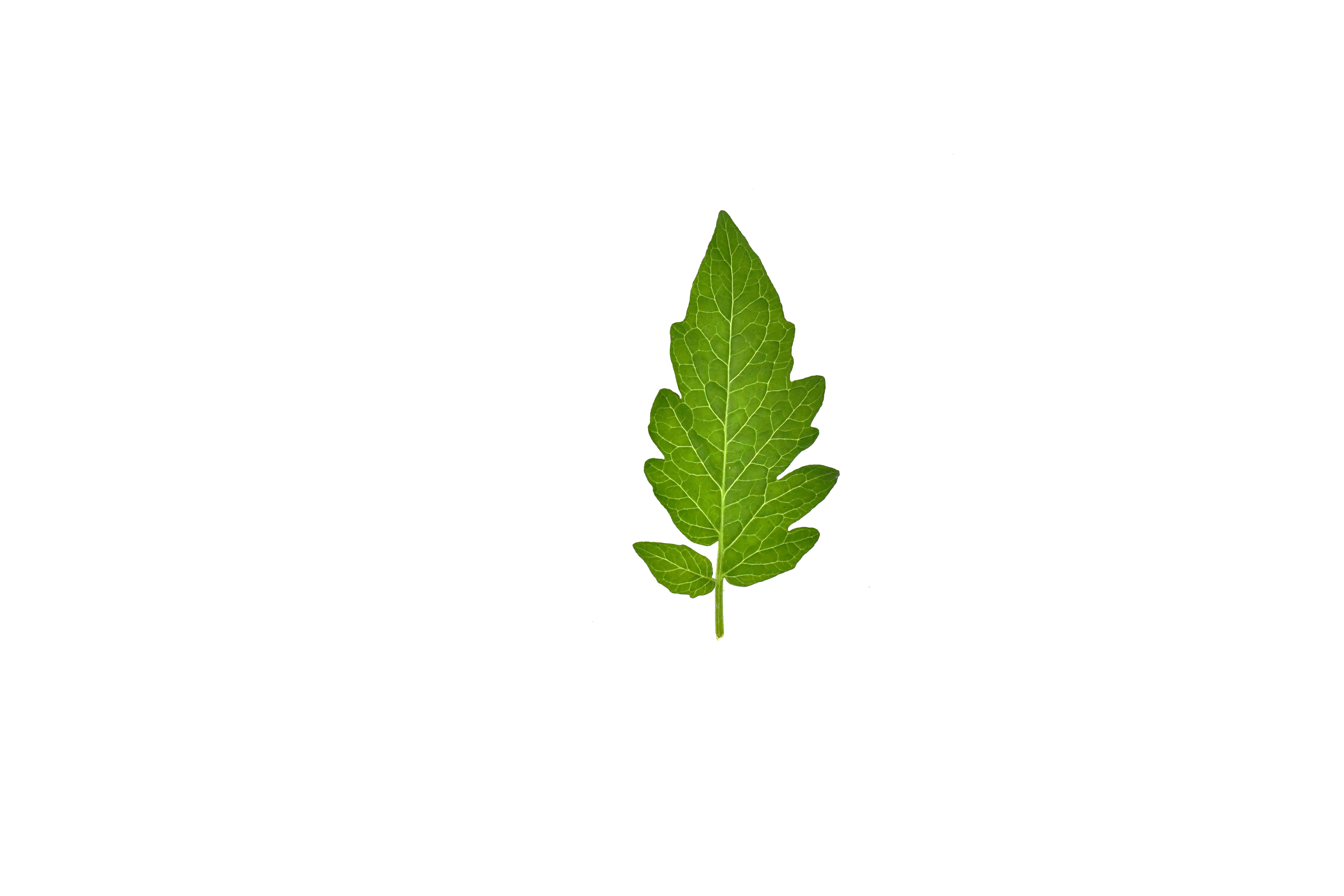

Supplement: Supplementary file 13 — Source data Fig. 4 [file 44318_2024_278_MOESM13_ESM.zip › figure 4C/1_Mock WT H2O.jpg]

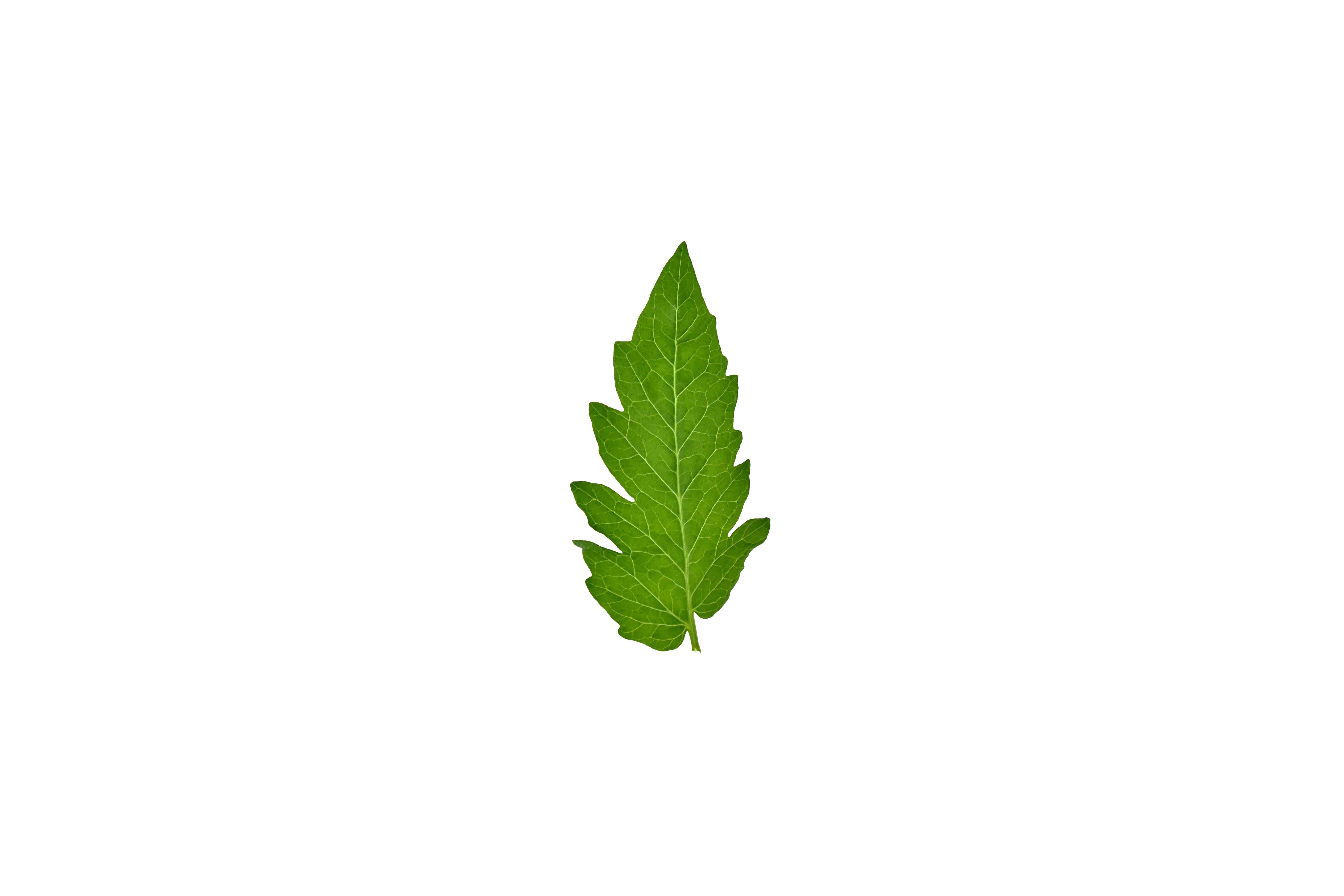

Supplement: Supplementary file 13 — Source data Fig. 4 [file 44318_2024_278_MOESM13_ESM.zip › figure 4C/2_Mock WT PSK.jpg]

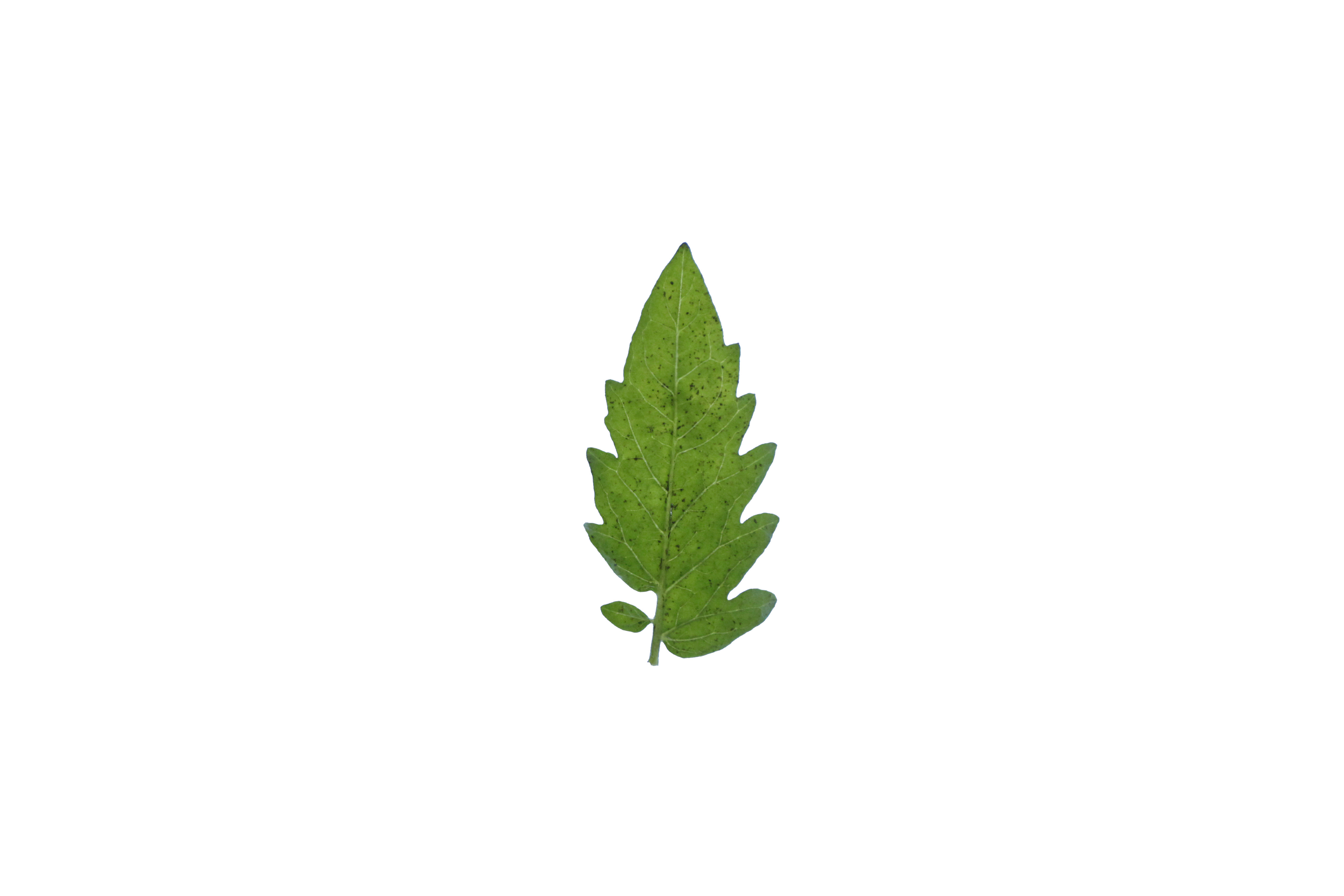

Supplement: Supplementary file 13 — Source data Fig. 4 [file 44318_2024_278_MOESM13_ESM.zip › figure 4C/3_B.c WT H2O.jpg]

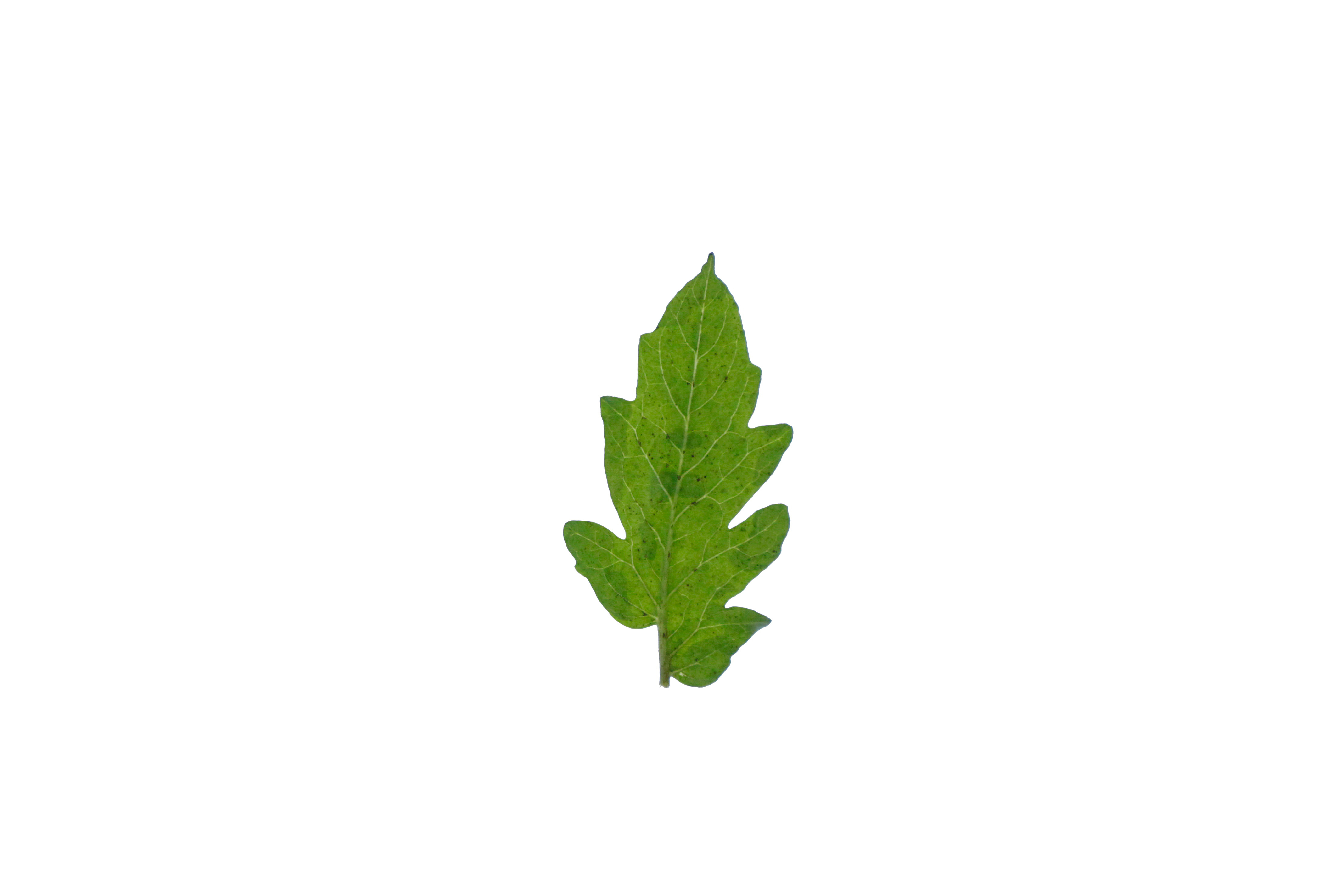

Supplement: Supplementary file 13 — Source data Fig. 4 [file 44318_2024_278_MOESM13_ESM.zip › figure 4C/4_B.c WT PSK.jpg]

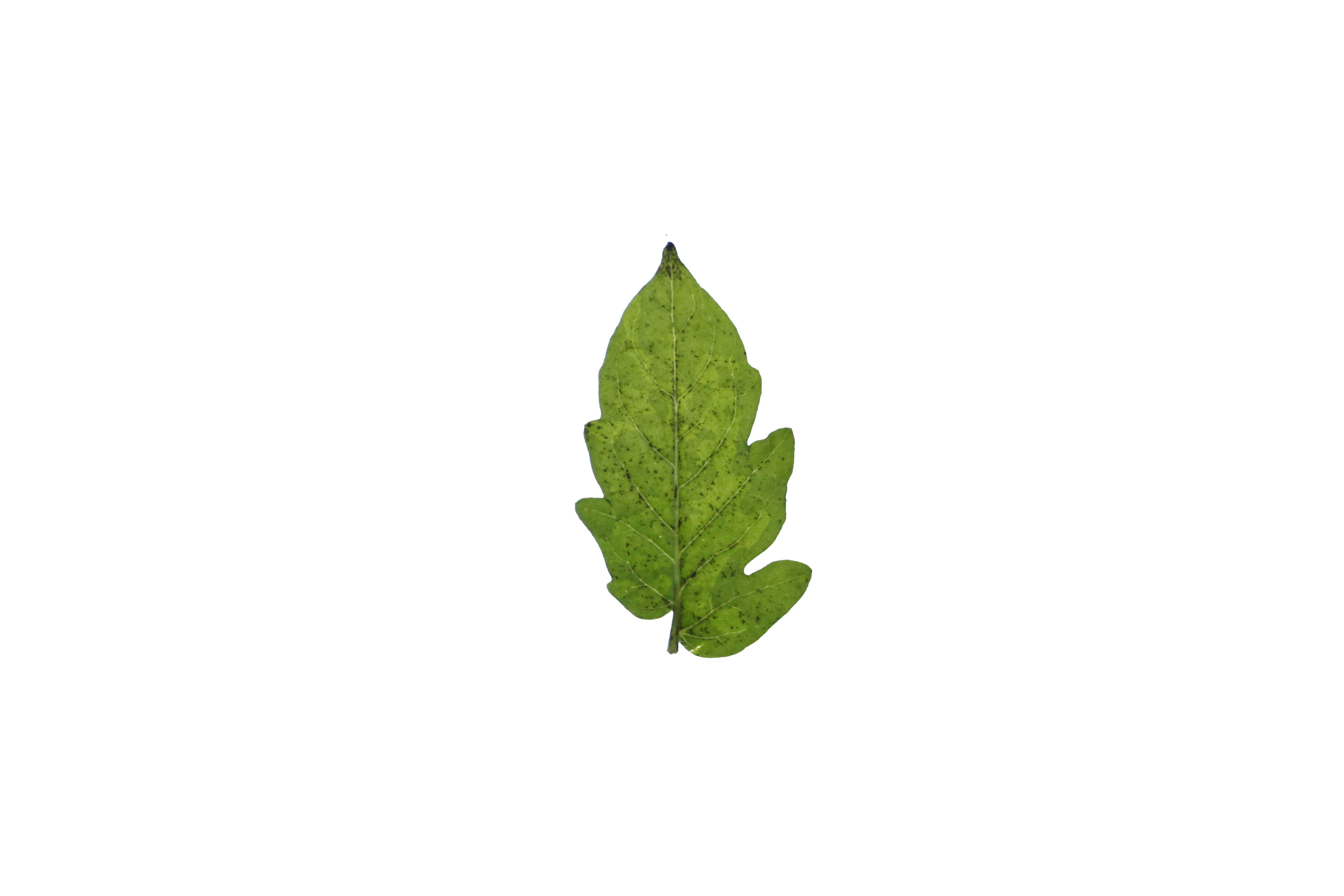

Supplement: Supplementary file 13 — Source data Fig. 4 [file 44318_2024_278_MOESM13_ESM.zip › figure 4C/5_B.c pskr1 H2O.jpg]

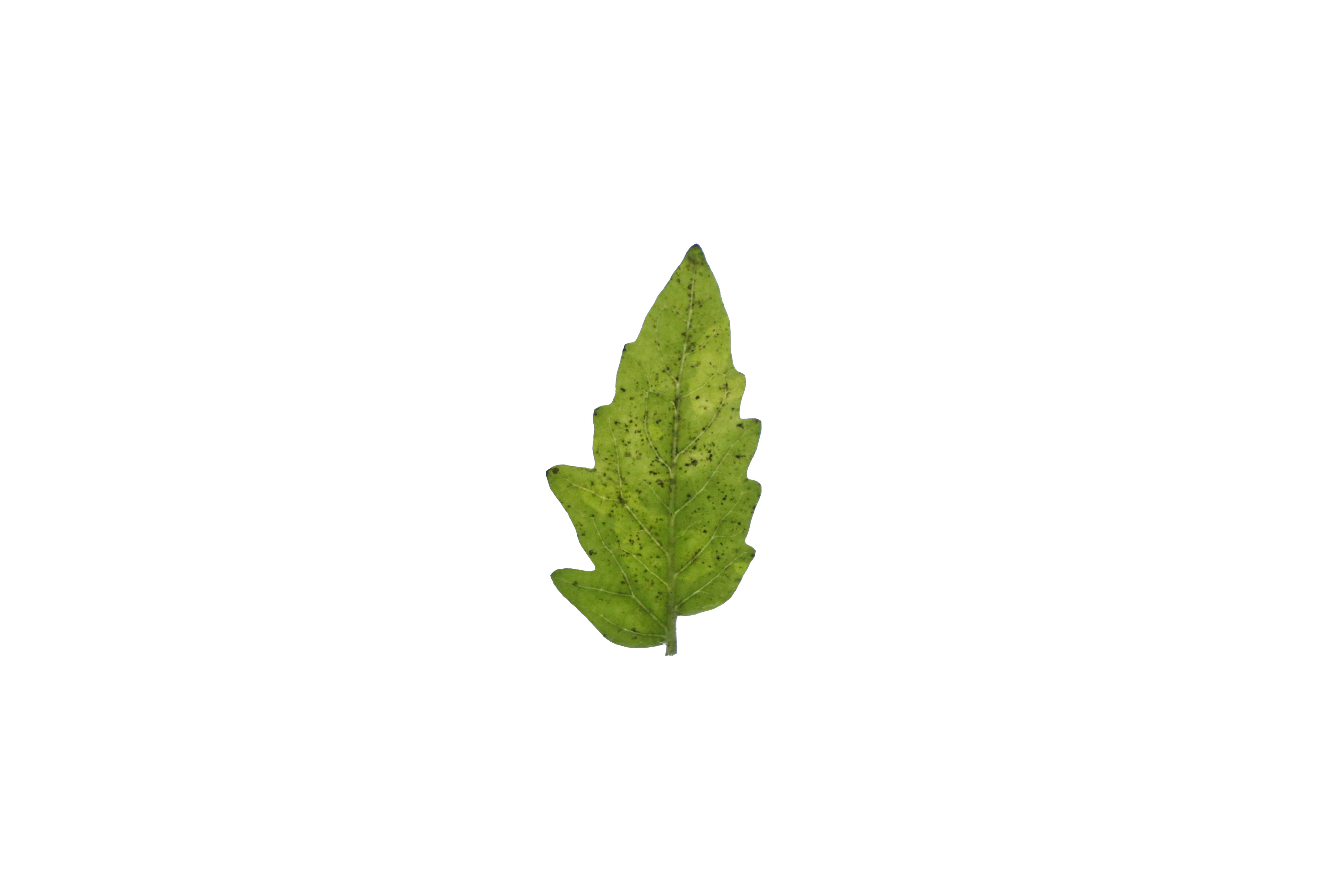

Supplement: Supplementary file 13 — Source data Fig. 4 [file 44318_2024_278_MOESM13_ESM.zip › figure 4C/6_B.c pskr1 PSK.jpg]

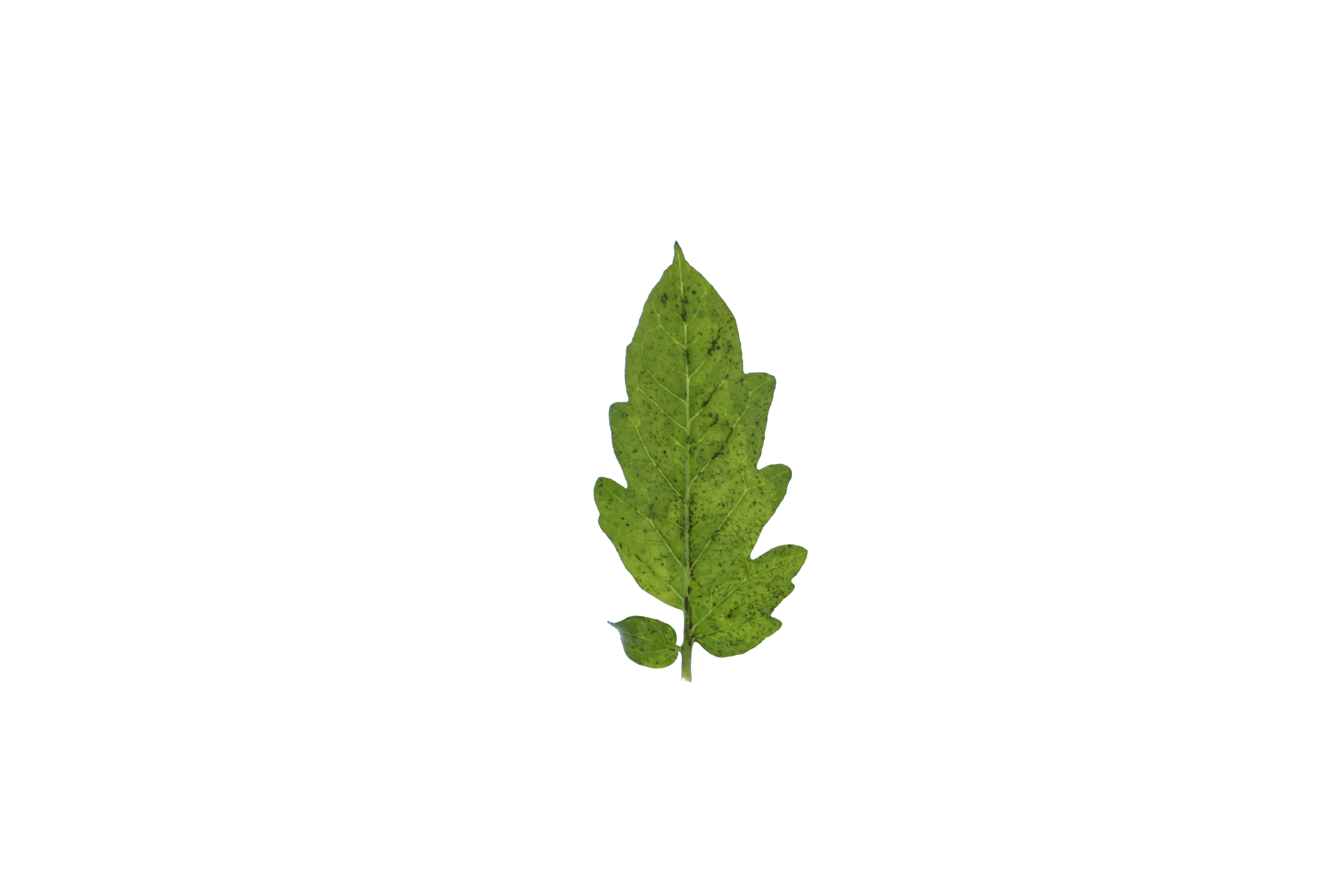

Supplement: Supplementary file 13 — Source data Fig. 4 [file 44318_2024_278_MOESM13_ESM.zip › figure 4C/7_B.c brak H2O.jpg]

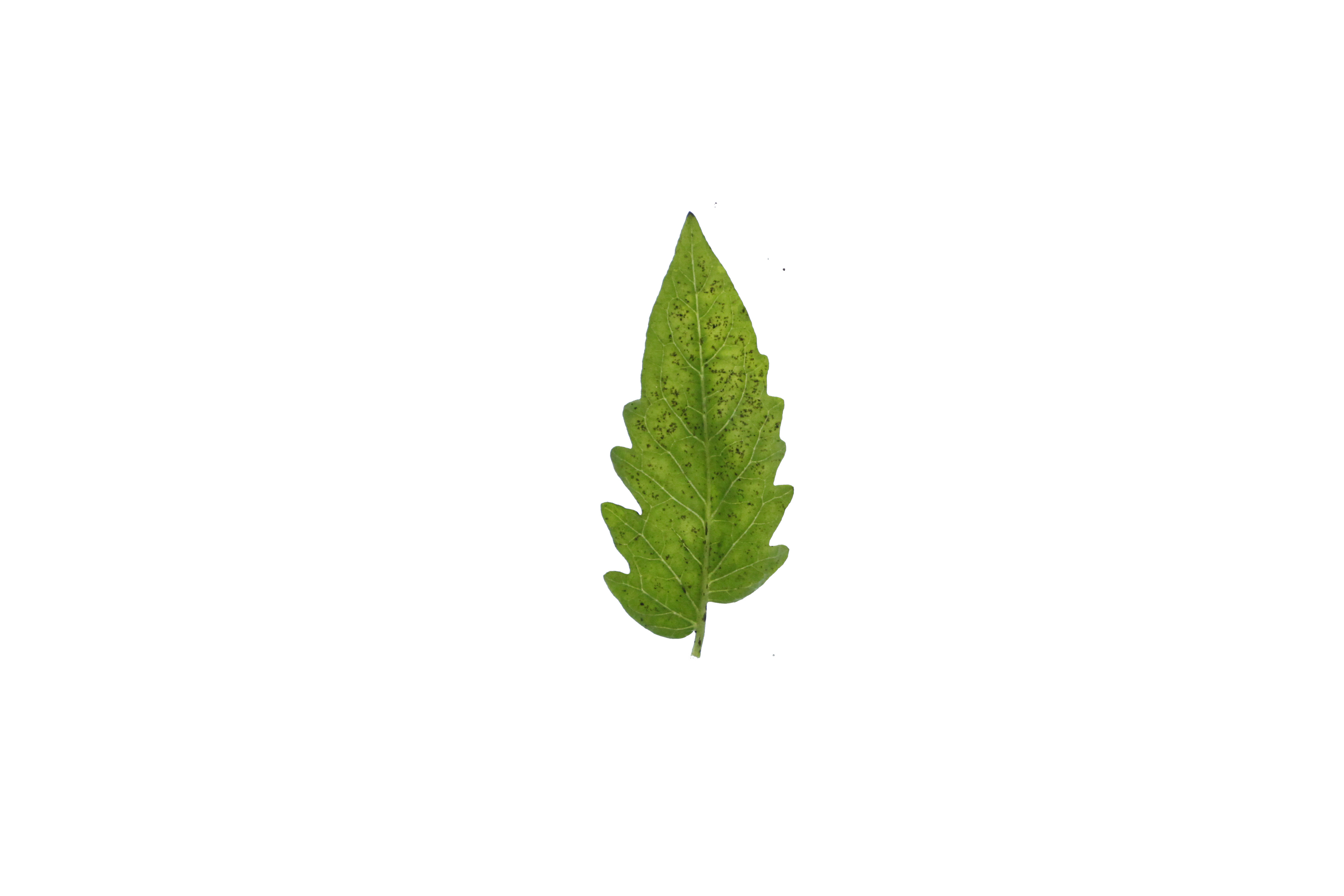

Supplement: Supplementary file 13 — Source data Fig. 4 [file 44318_2024_278_MOESM13_ESM.zip › figure 4C/8_B.c brak PSK.jpg]

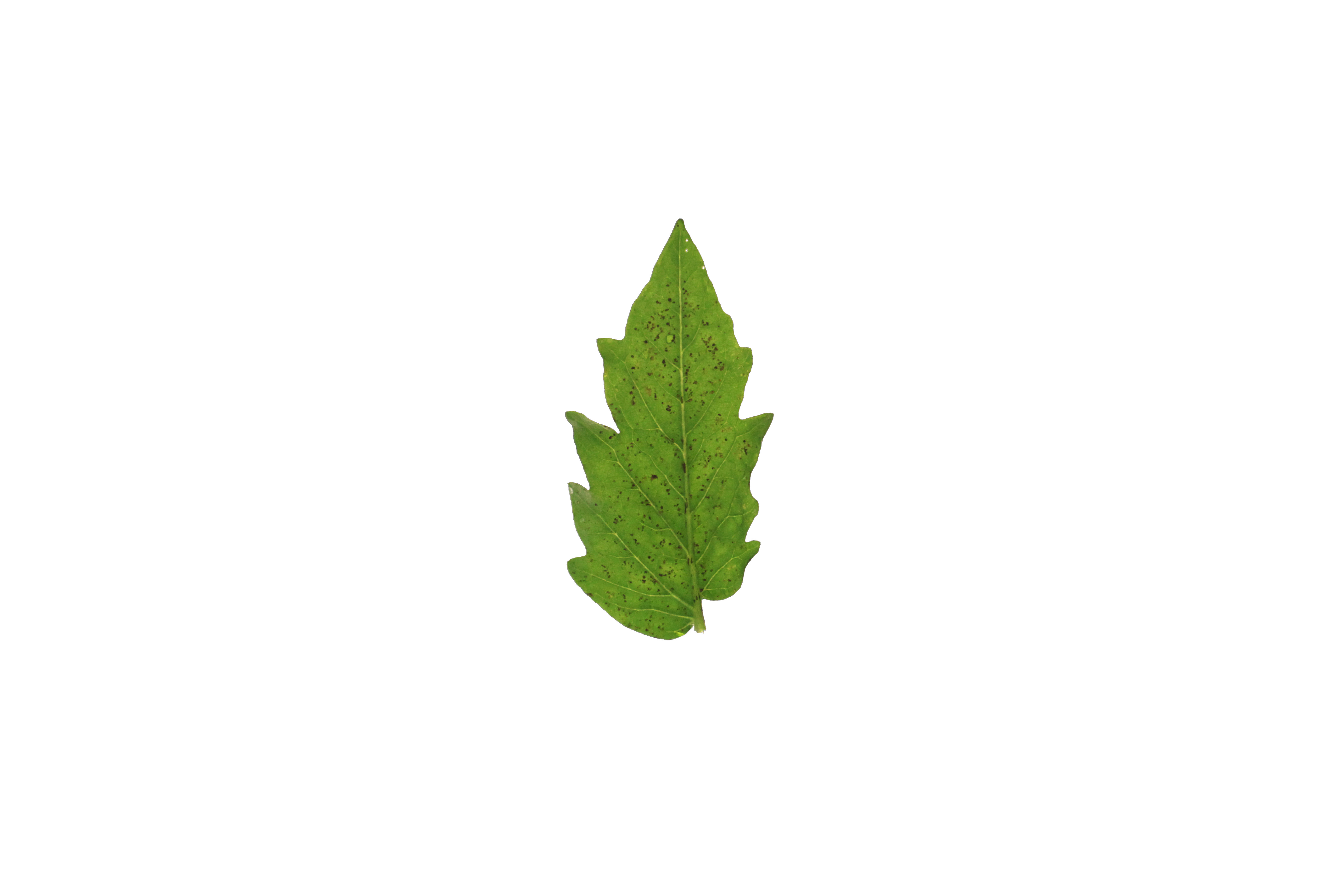

Supplement: Supplementary file 13 — Source data Fig. 4 [file 44318_2024_278_MOESM13_ESM.zip › figure 4C/9_B.c pskr1 brak H2O.jpg]

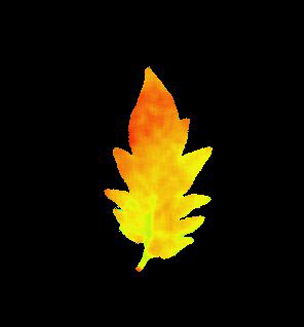

Supplement: Supplementary file 13 — Source data Fig. 4 [file 44318_2024_278_MOESM13_ESM.zip › Figure 4D/10_B.c pskr1 brak PSK.jpg]

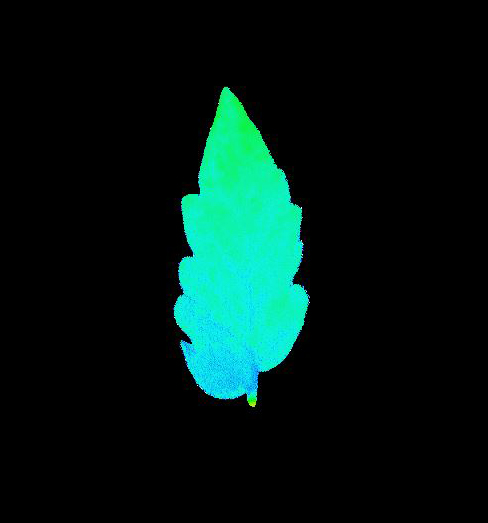

Supplement: Supplementary file 13 — Source data Fig. 4 [file 44318_2024_278_MOESM13_ESM.zip › Figure 4D/1_Mock WT H2O.jpg]

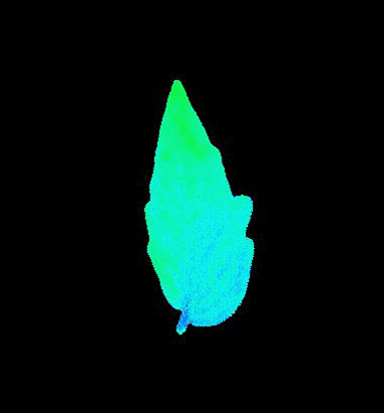

Supplement: Supplementary file 13 — Source data Fig. 4 [file 44318_2024_278_MOESM13_ESM.zip › Figure 4D/2_Mock WT PSK.jpg]

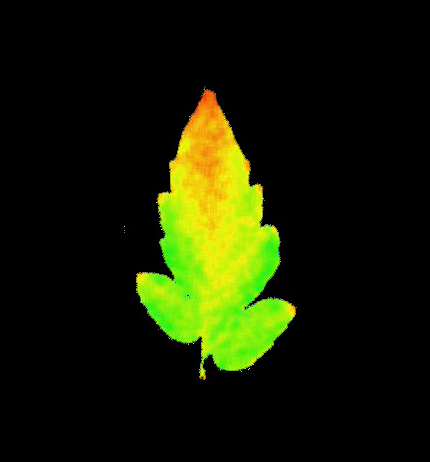

Supplement: Supplementary file 13 — Source data Fig. 4 [file 44318_2024_278_MOESM13_ESM.zip › Figure 4D/3_B.c WT H2O.jpg]

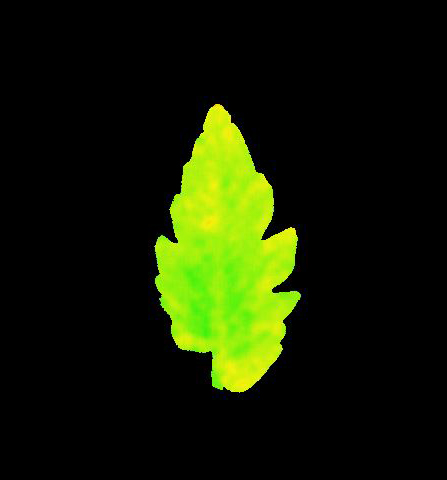

Supplement: Supplementary file 13 — Source data Fig. 4 [file 44318_2024_278_MOESM13_ESM.zip › Figure 4D/4_B.c WT PSK.jpg]

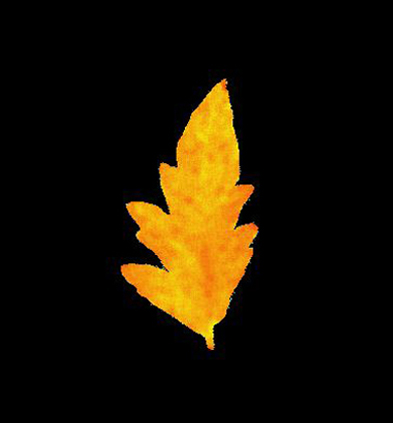

Supplement: Supplementary file 13 — Source data Fig. 4 [file 44318_2024_278_MOESM13_ESM.zip › Figure 4D/5_B.c pskr1 H2O.jpg]

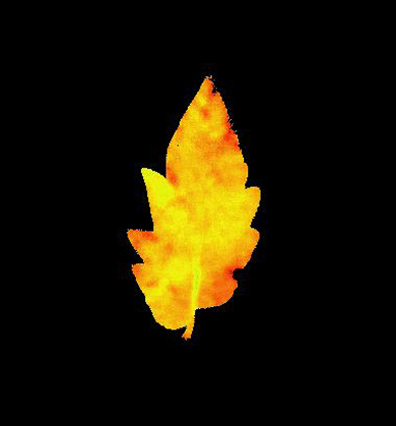

Supplement: Supplementary file 13 — Source data Fig. 4 [file 44318_2024_278_MOESM13_ESM.zip › Figure 4D/6_B.c pskr1 PSK.jpg]

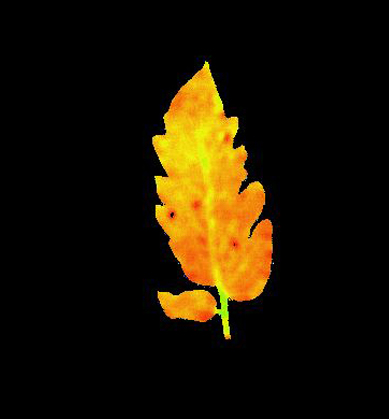

Supplement: Supplementary file 13 — Source data Fig. 4 [file 44318_2024_278_MOESM13_ESM.zip › Figure 4D/7_B.c brak H2O.jpg]

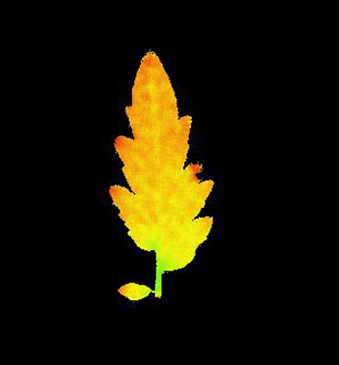

Supplement: Supplementary file 13 — Source data Fig. 4 [file 44318_2024_278_MOESM13_ESM.zip › Figure 4D/8_B.c brak PSK.jpg]

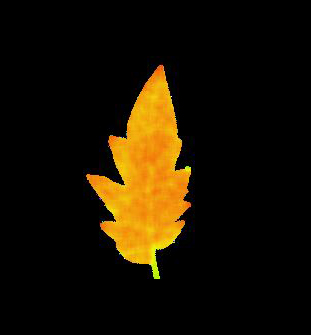

Supplement: Supplementary file 13 — Source data Fig. 4 [file 44318_2024_278_MOESM13_ESM.zip › Figure 4D/9_B.c pskr1 brak H2O.jpg]

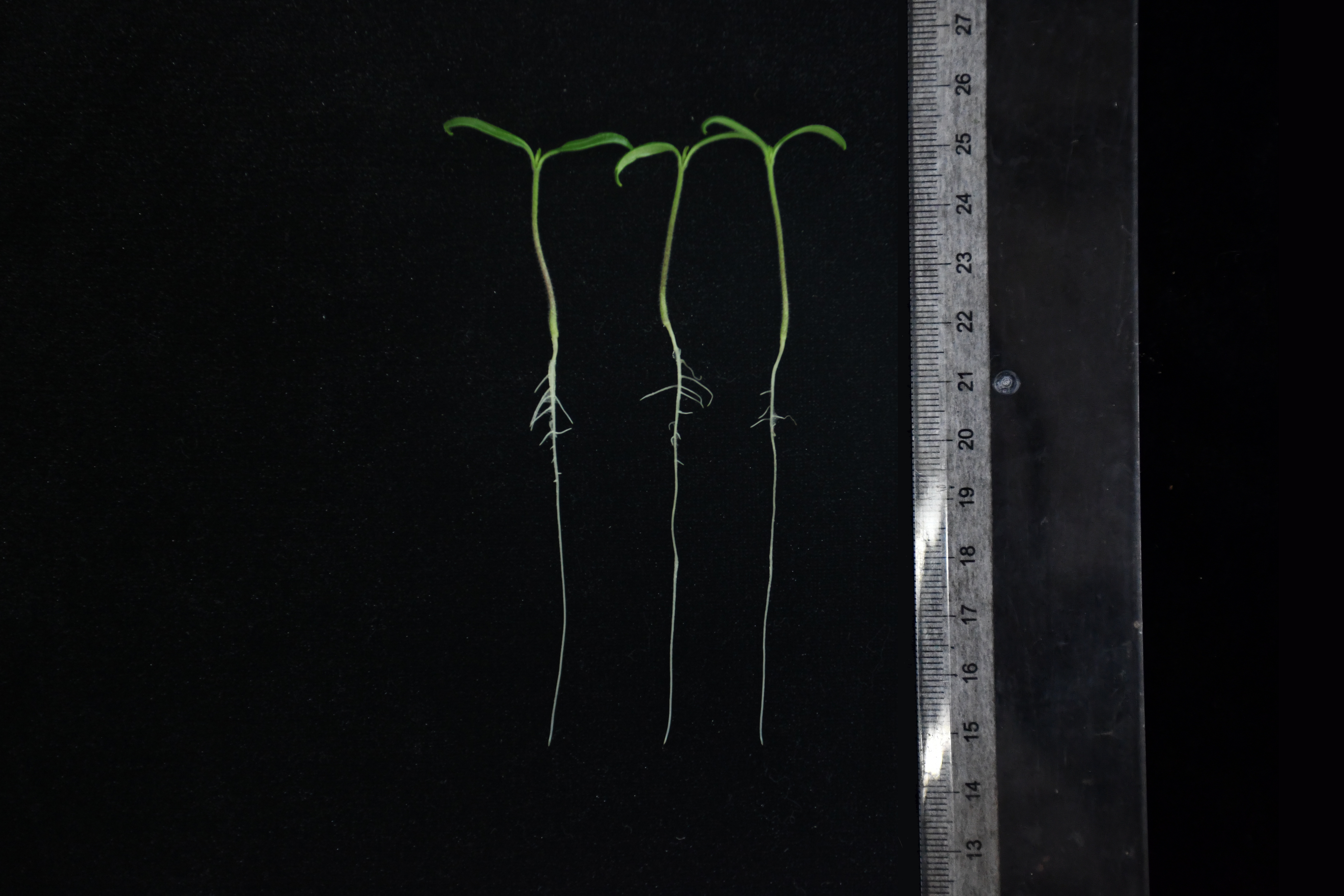

Supplement: Supplementary file 14 — Source data Fig. 5 [file 44318_2024_278_MOESM14_ESM.zip › Figure 5A/1_WT_TRV0 _H2O.jpg]

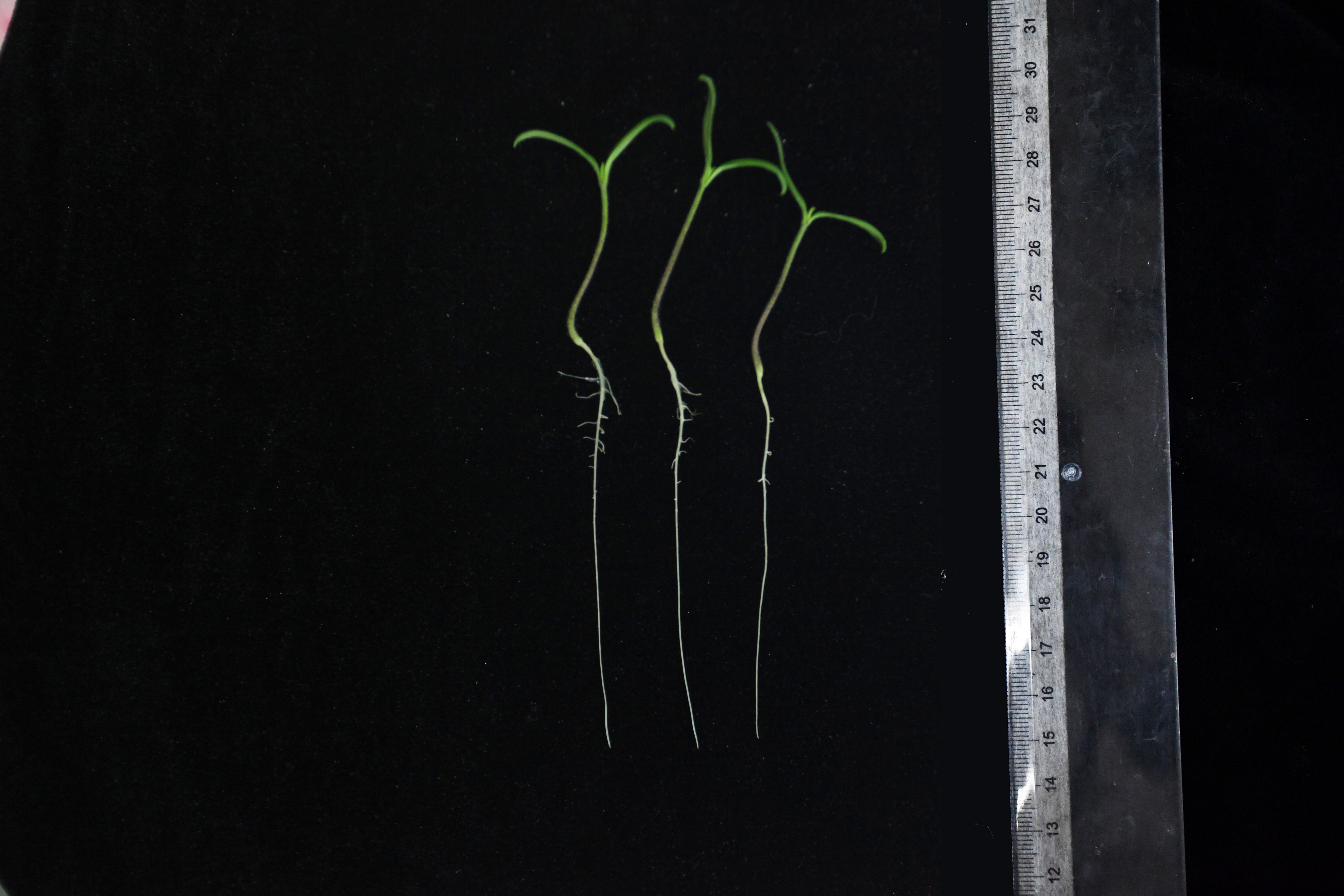

Supplement: Supplementary file 14 — Source data Fig. 5 [file 44318_2024_278_MOESM14_ESM.zip › Figure 5A/2_WT_TRV0 _PSK.jpg]

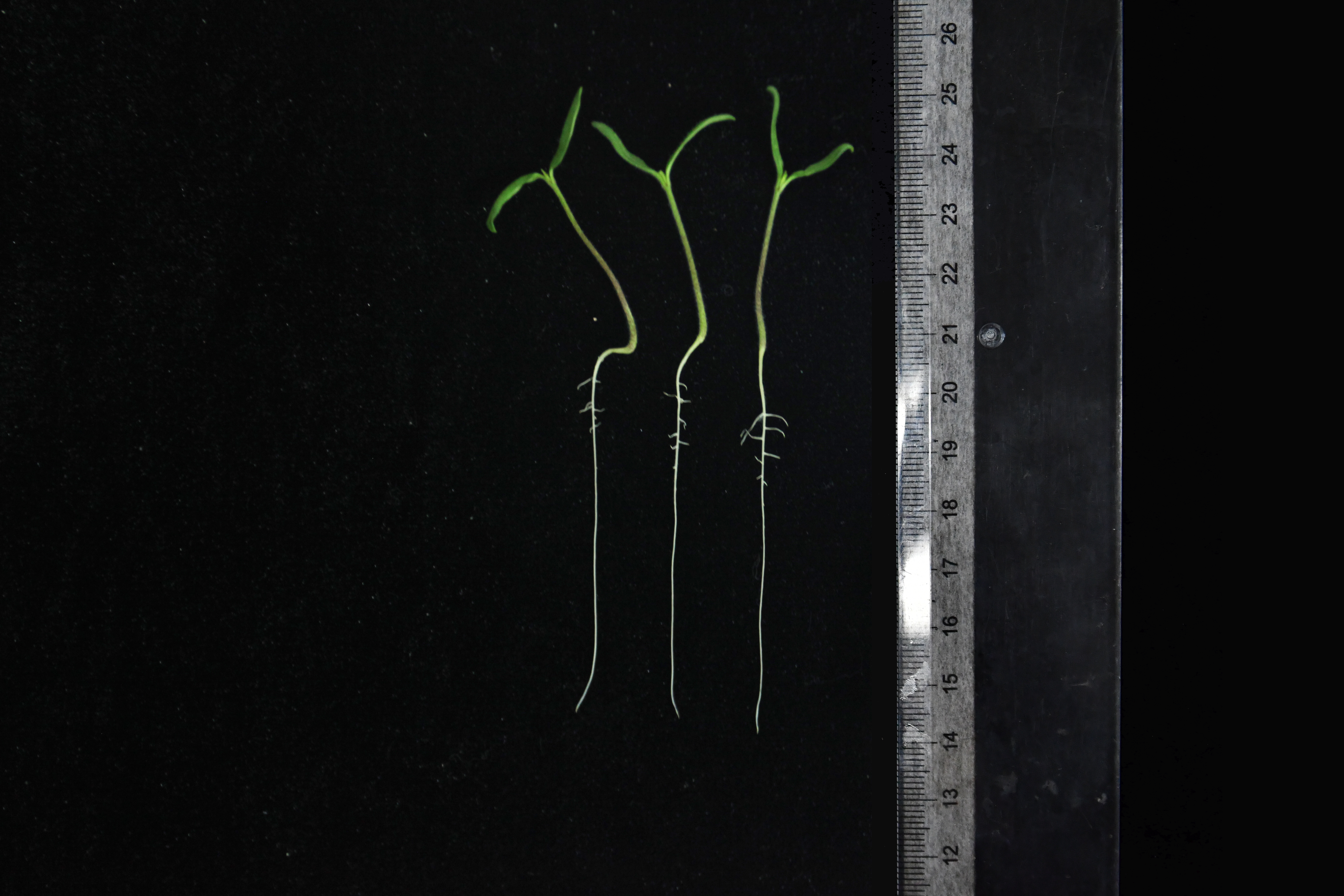

Supplement: Supplementary file 14 — Source data Fig. 5 [file 44318_2024_278_MOESM14_ESM.zip › Figure 5A/3_WT_TRVBRAK _H2O.jpg]

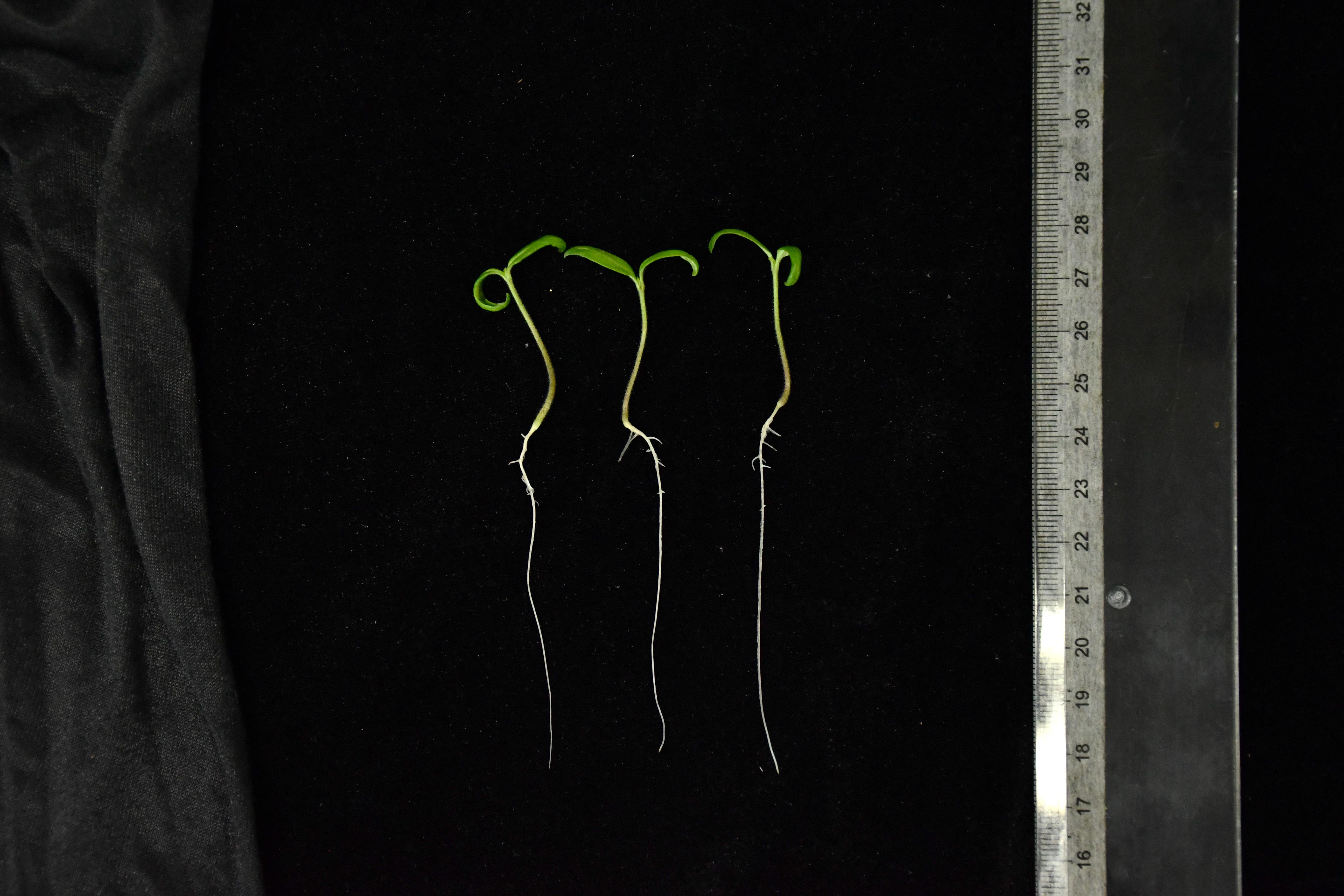

Supplement: Supplementary file 14 — Source data Fig. 5 [file 44318_2024_278_MOESM14_ESM.zip › Figure 5A/4_WT_TRVBRAK _PSK.jpg]

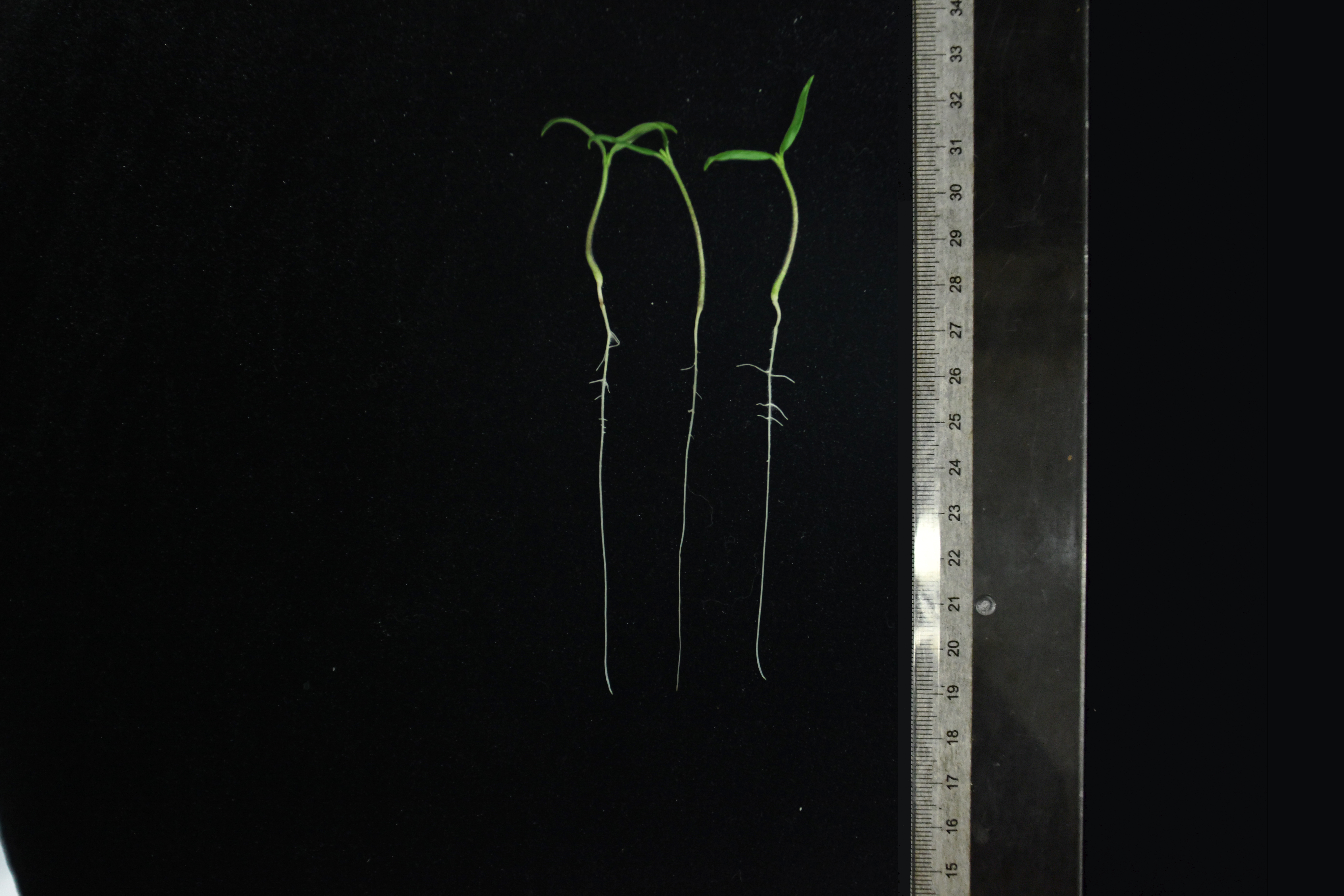

Supplement: Supplementary file 14 — Source data Fig. 5 [file 44318_2024_278_MOESM14_ESM.zip › Figure 5A/5_OEPSKR1_TRV0_H2O.jpg]

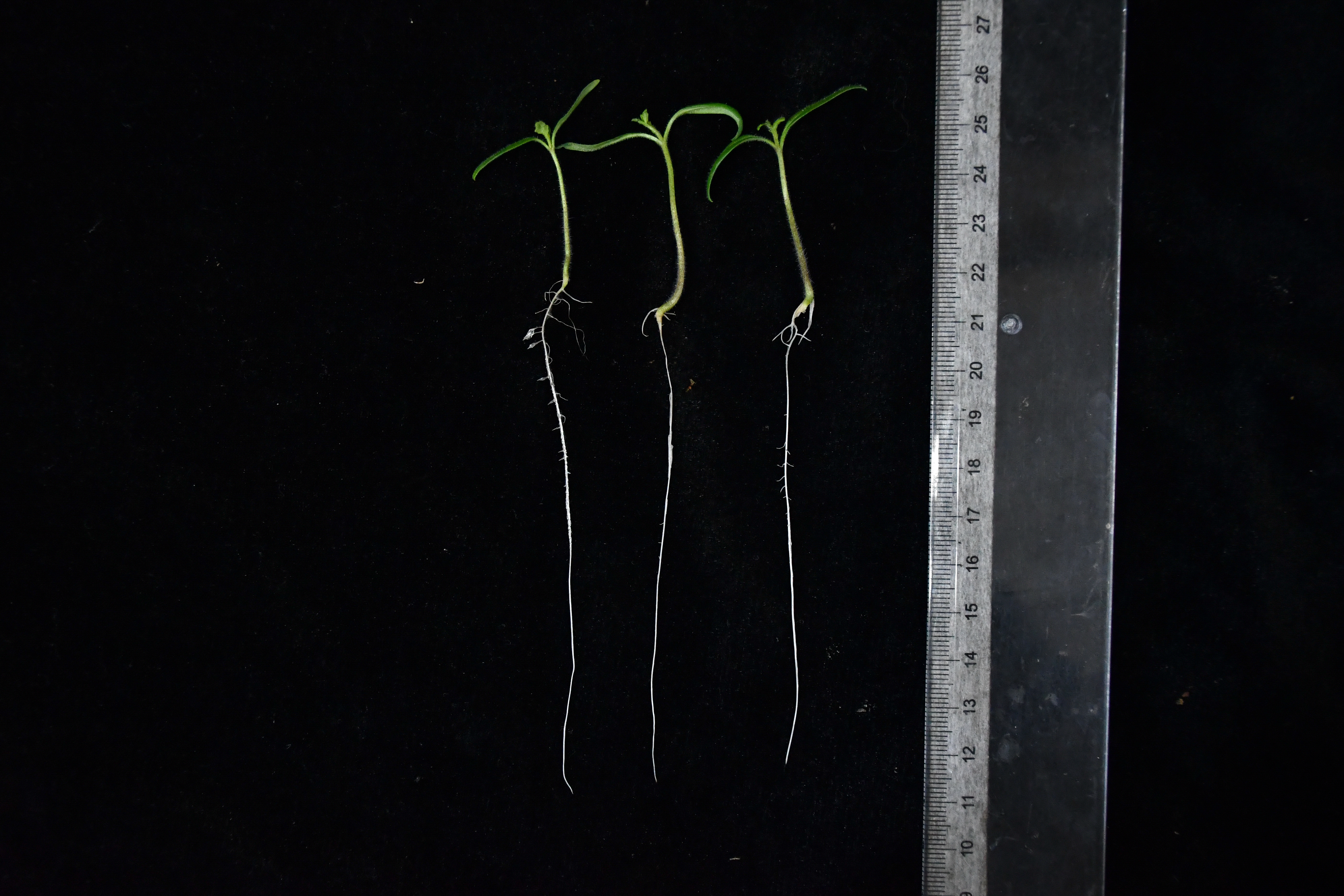

Supplement: Supplementary file 14 — Source data Fig. 5 [file 44318_2024_278_MOESM14_ESM.zip › Figure 5A/6_OEPSKR1_TRV0_PSK.jpg]

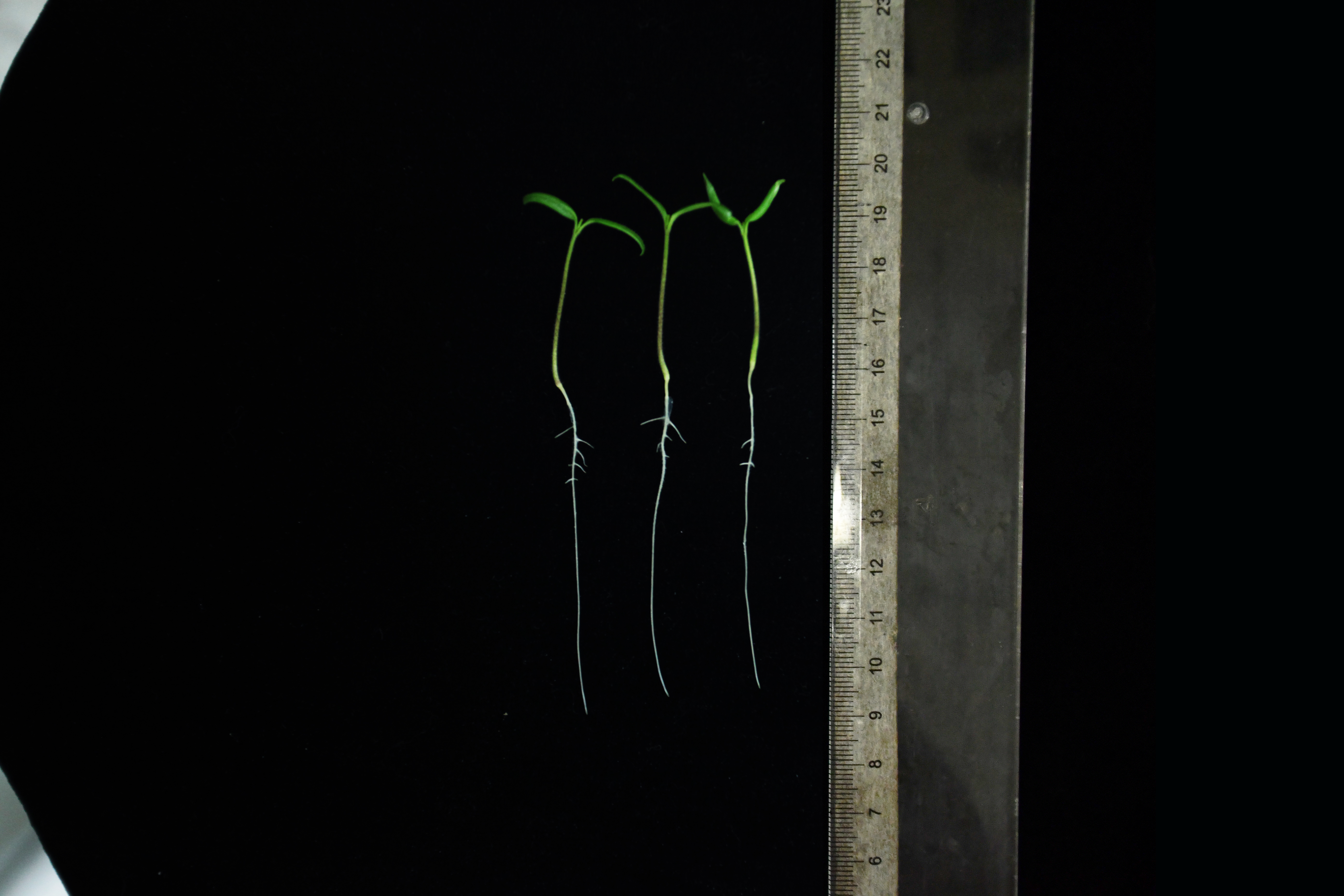

Supplement: Supplementary file 14 — Source data Fig. 5 [file 44318_2024_278_MOESM14_ESM.zip › Figure 5A/7_OEPSKR1 _TRVBRAK _H2O.jpg]

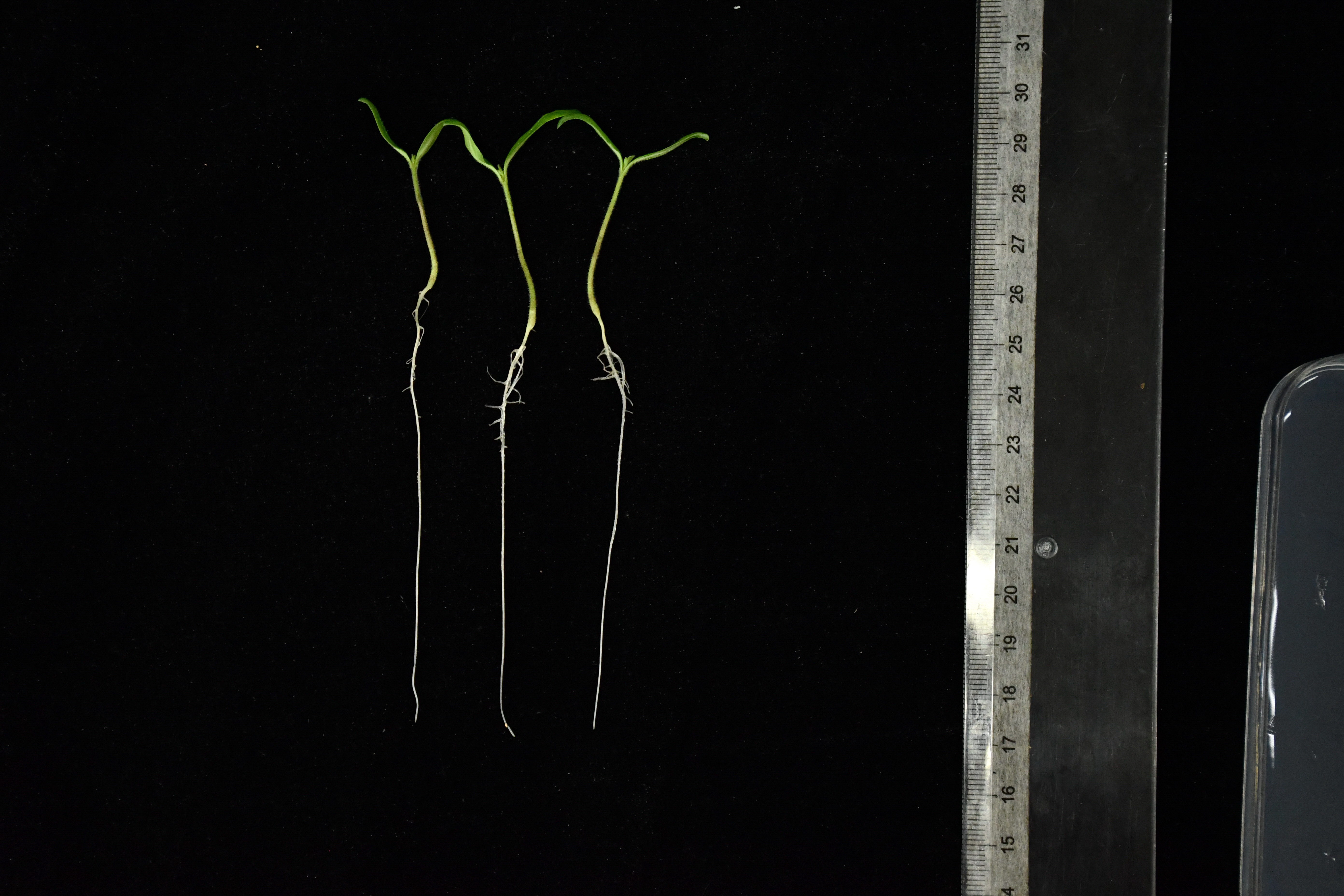

Supplement: Supplementary file 14 — Source data Fig. 5 [file 44318_2024_278_MOESM14_ESM.zip › Figure 5A/8_OEPSKR1 _TRVBRAK _PSK.jpg]

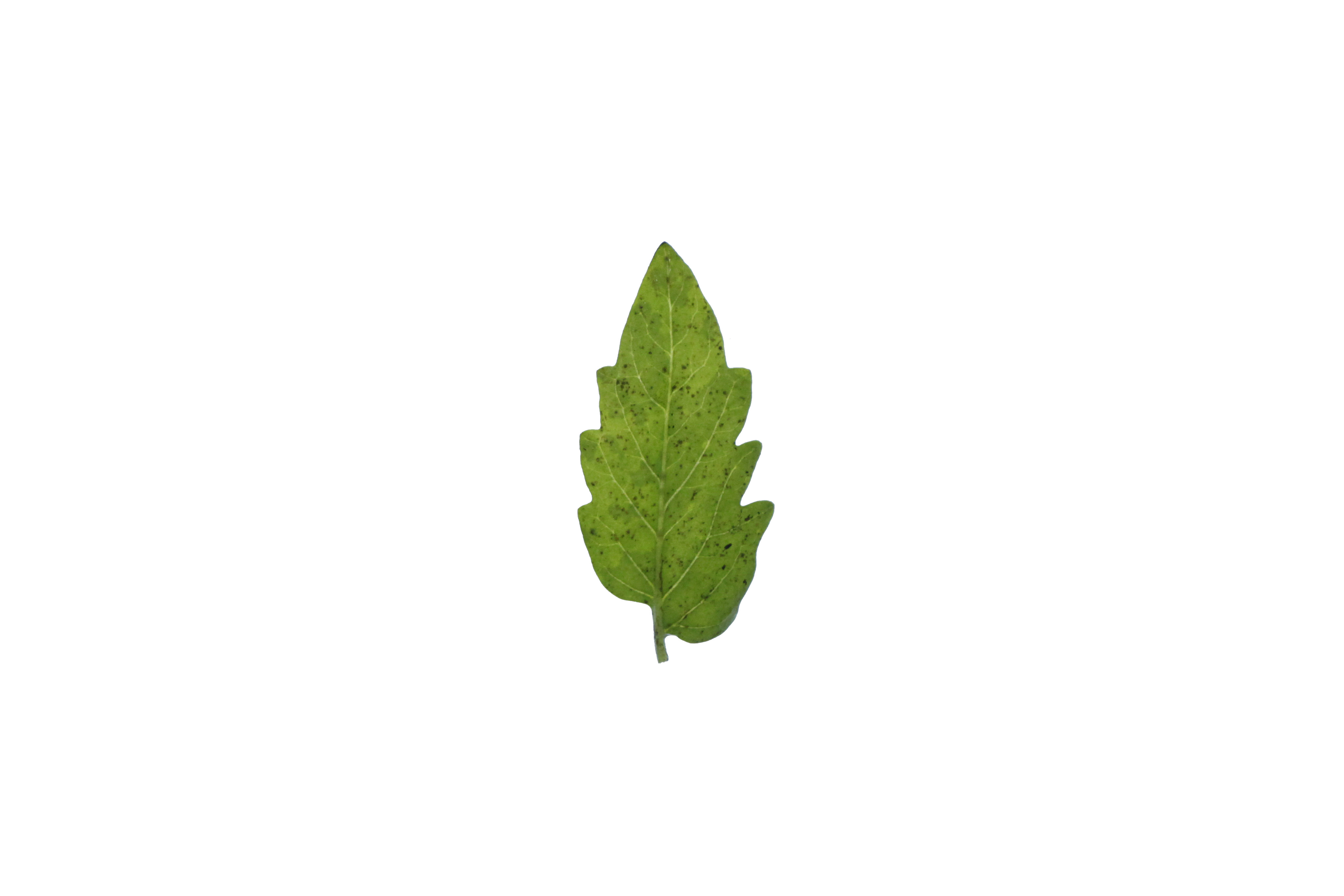

Supplement: Supplementary file 14 — Source data Fig. 5 [file 44318_2024_278_MOESM14_ESM.zip › Figure 5C/1_WT_TRV0 _H2O.jpg]

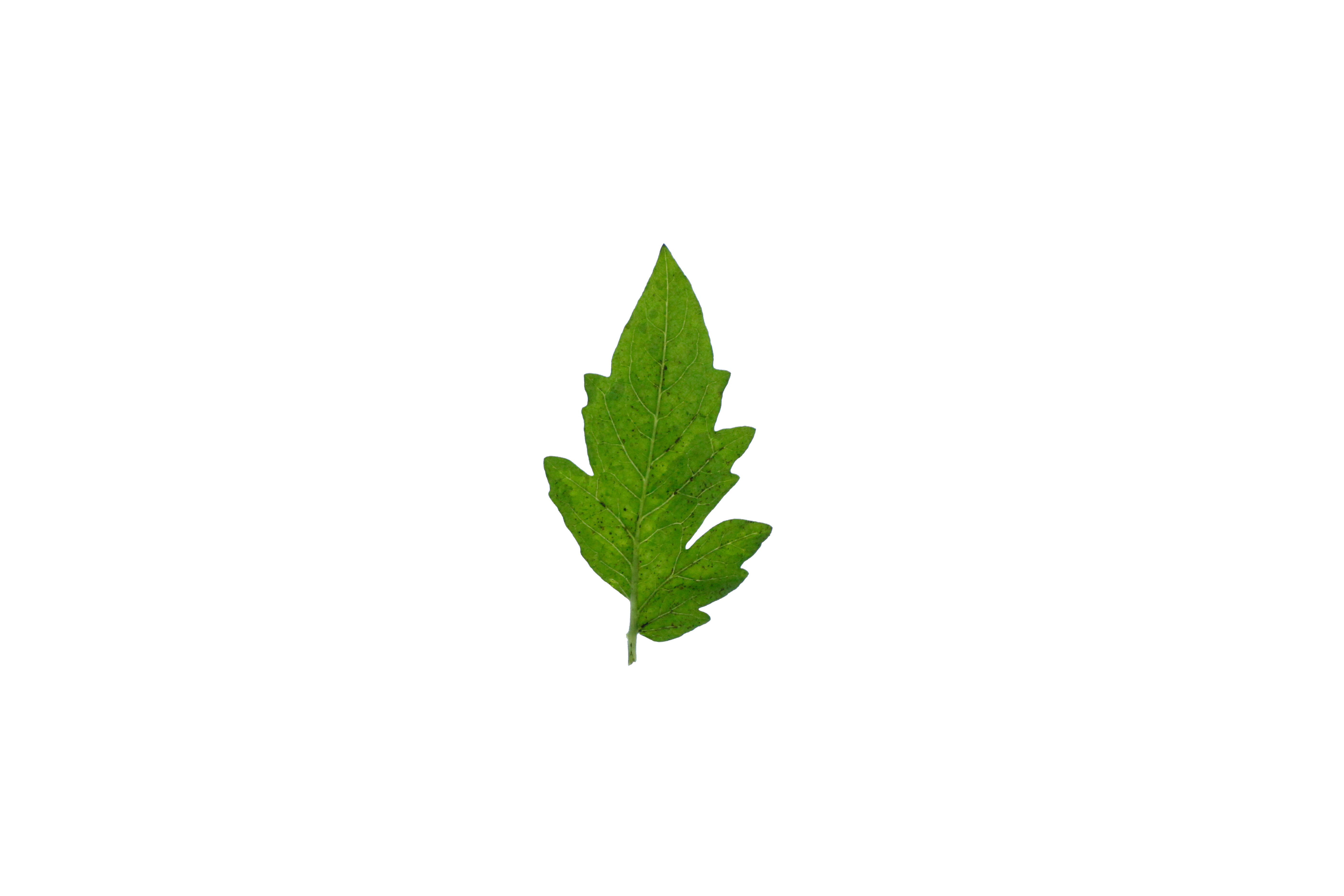

Supplement: Supplementary file 14 — Source data Fig. 5 [file 44318_2024_278_MOESM14_ESM.zip › Figure 5C/2_WT_TRV0 _PSK.jpg]

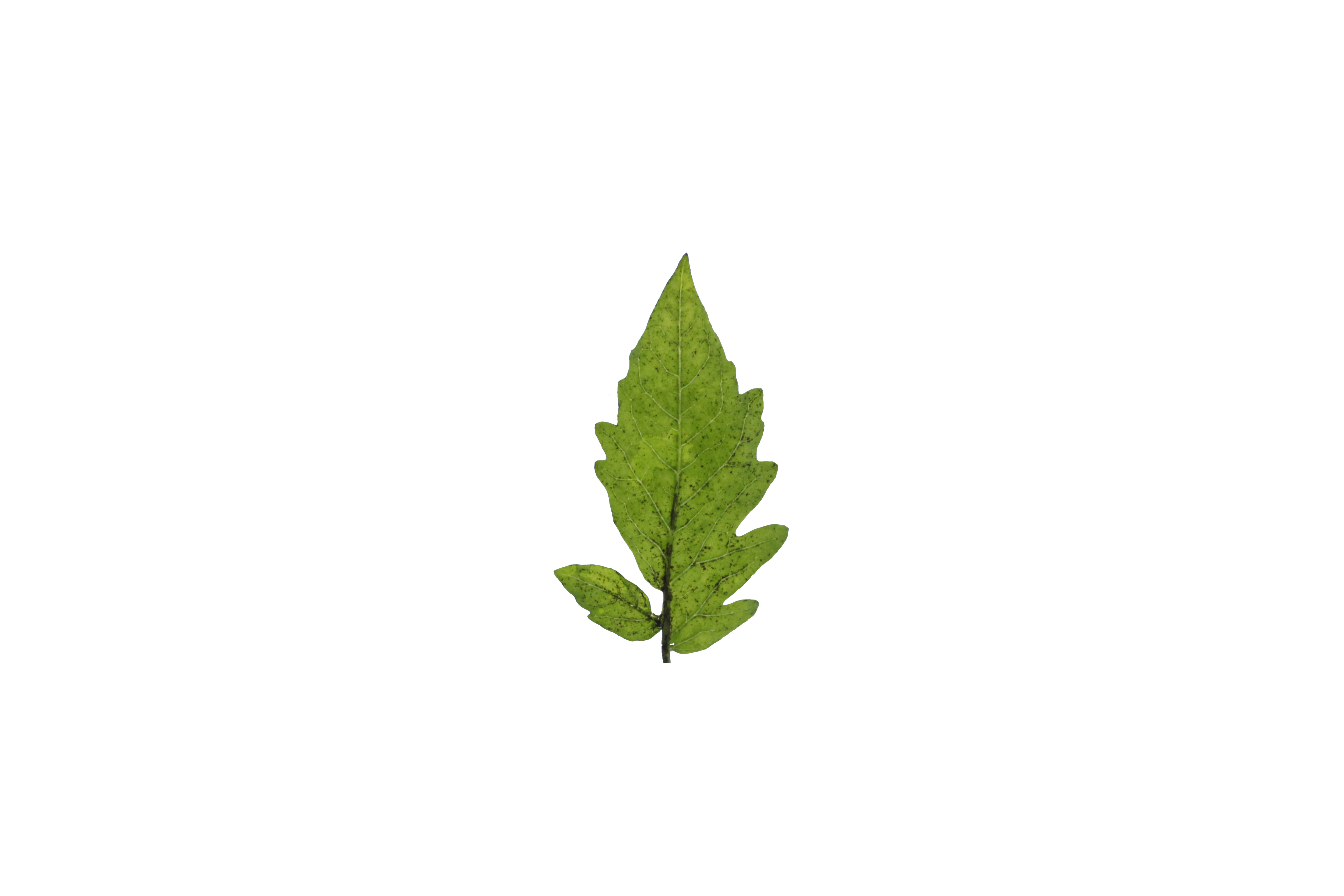

Supplement: Supplementary file 14 — Source data Fig. 5 [file 44318_2024_278_MOESM14_ESM.zip › Figure 5C/3_WT_TRVBRAK _H2O.jpg]

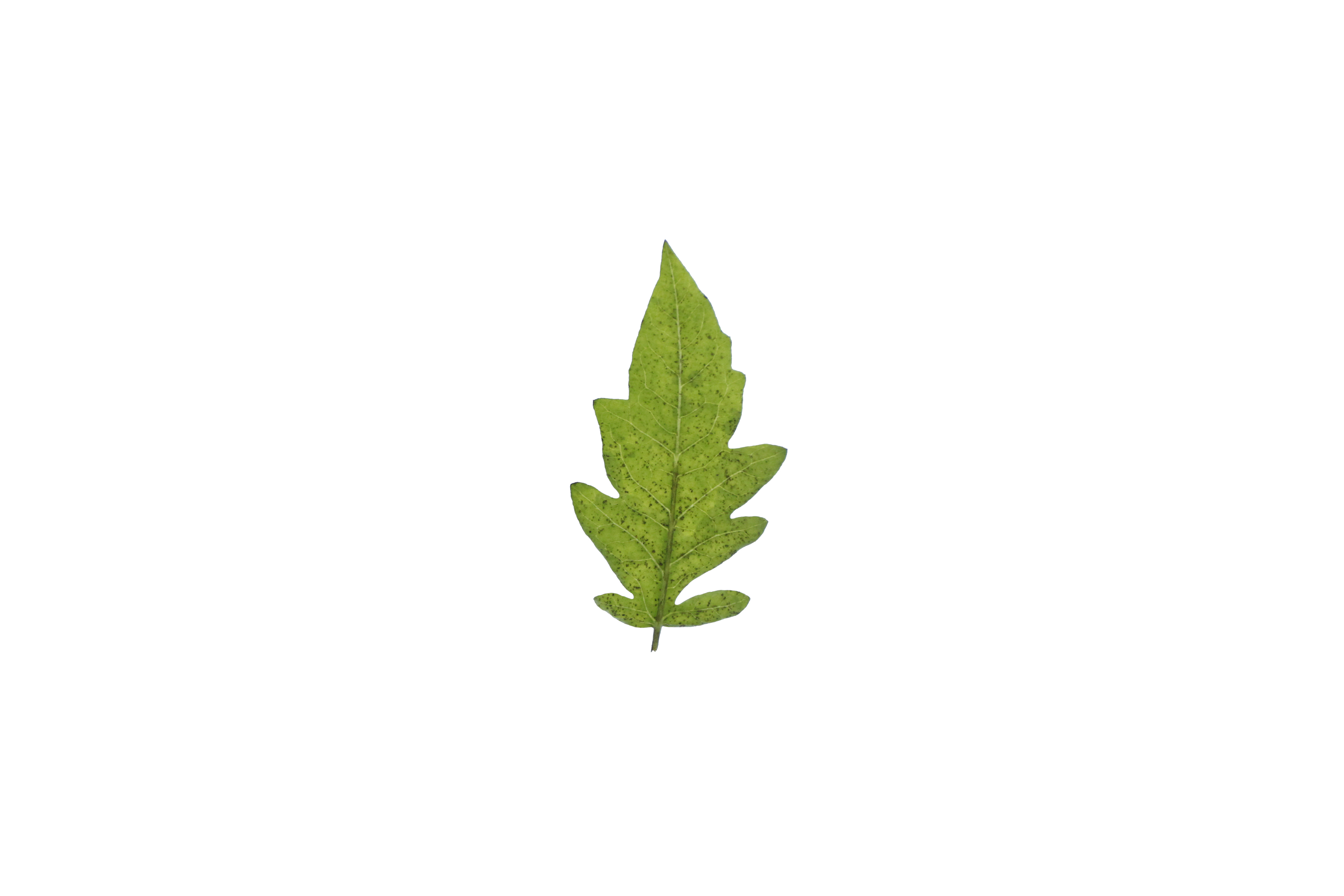

Supplement: Supplementary file 14 — Source data Fig. 5 [file 44318_2024_278_MOESM14_ESM.zip › Figure 5C/4_WT_TRVBRAK _PSK.jpg]

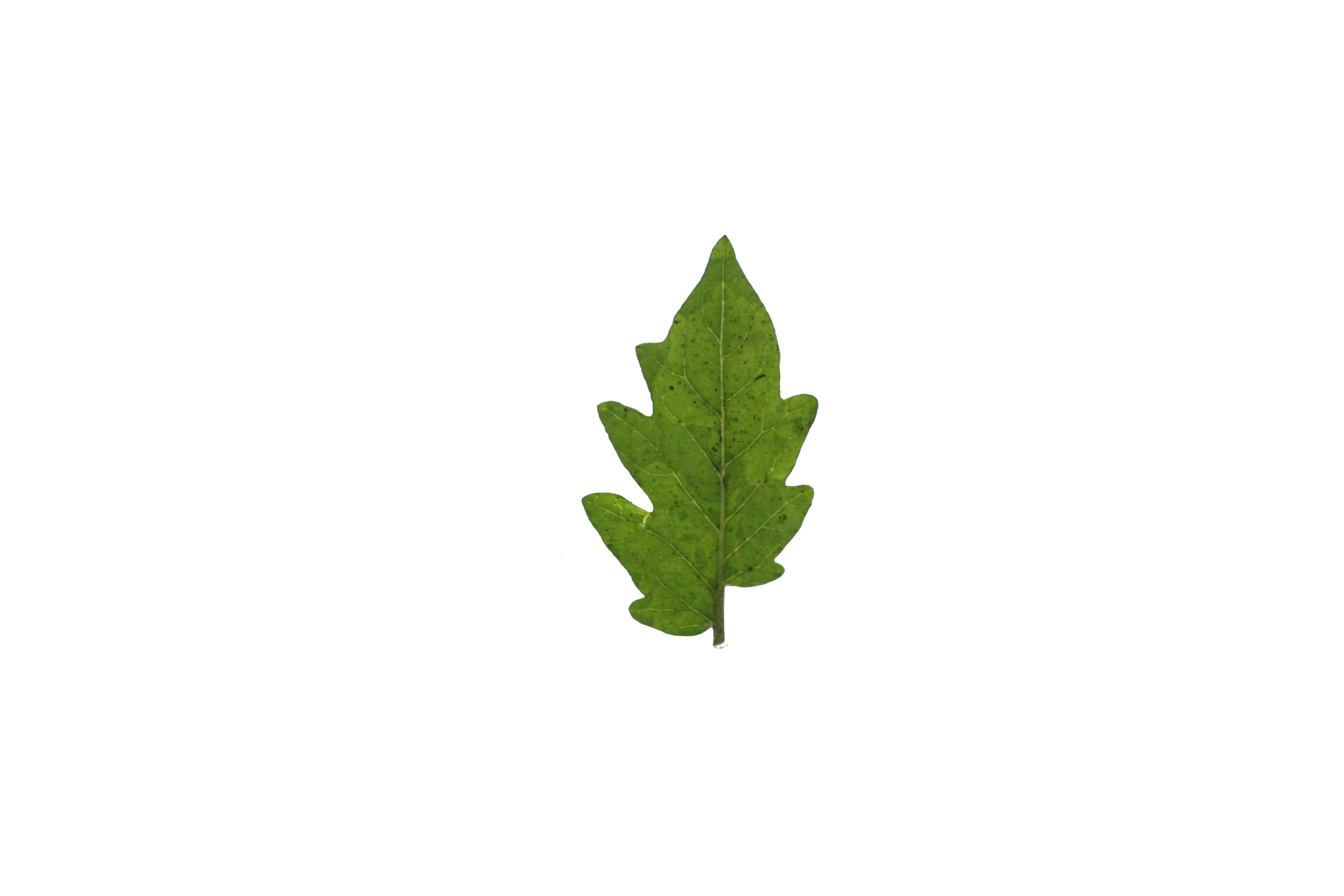

Supplement: Supplementary file 14 — Source data Fig. 5 [file 44318_2024_278_MOESM14_ESM.zip › Figure 5C/5_OEPSKR1_TRV0_H2O.jpg]
