# Supplementary figures and images for: A novel LRR receptor-like kinase BRAK reciprocally phosphorylates PSKR1 to enhance growth and defense in tomato (part 2 of 2)
Source: EMBO J. 2024 Oct 24;43(23):16. doi: 10.1038/s44318-024-00278-z (PMC11612273; doi:10.1038/s44318-024-00278-z)

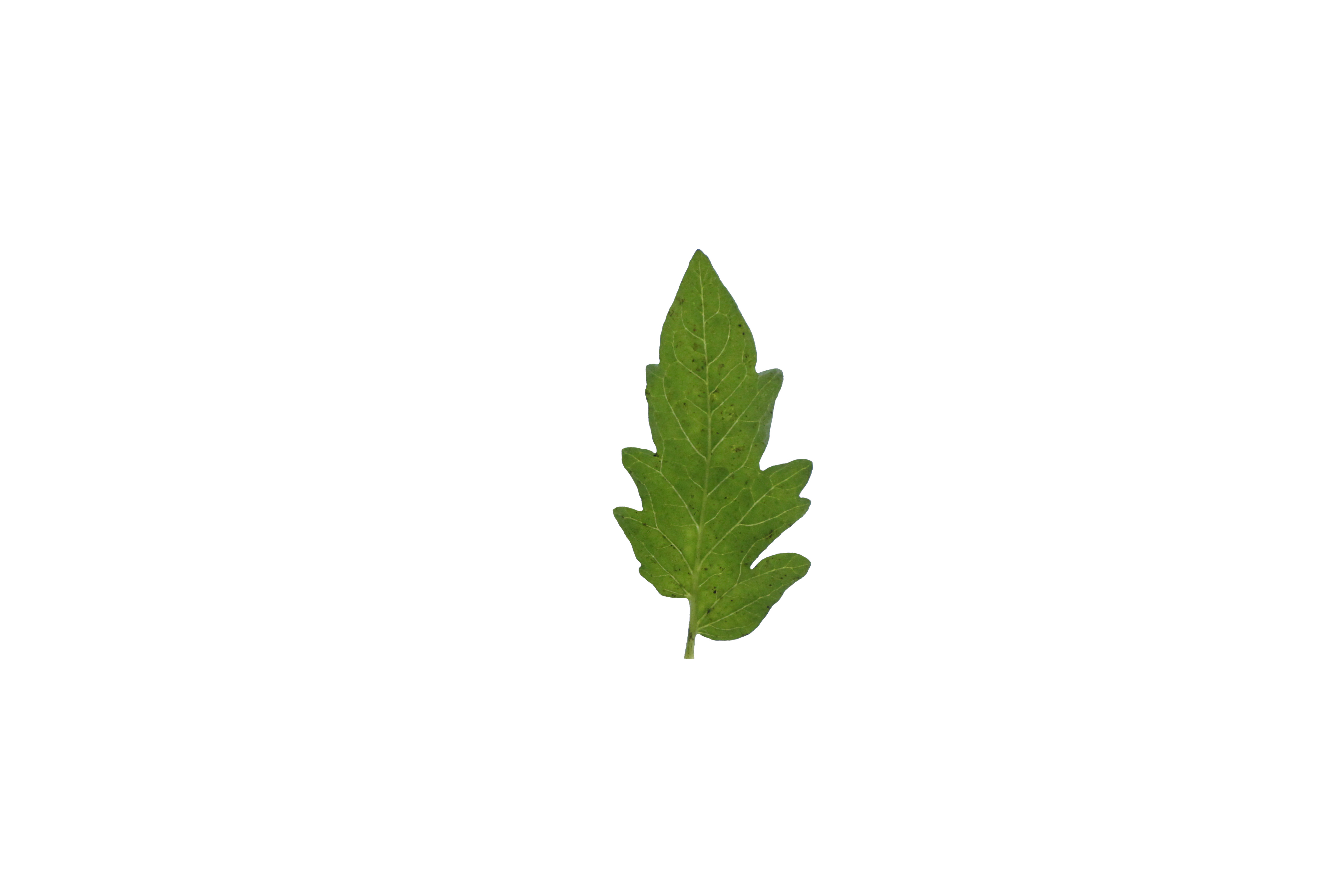

Supplement: Supplementary file 14 — Source data Fig. 5 [file 44318_2024_278_MOESM14_ESM.zip › Figure 5C/6_OEPSKR1_TRV0_PSK.jpg]

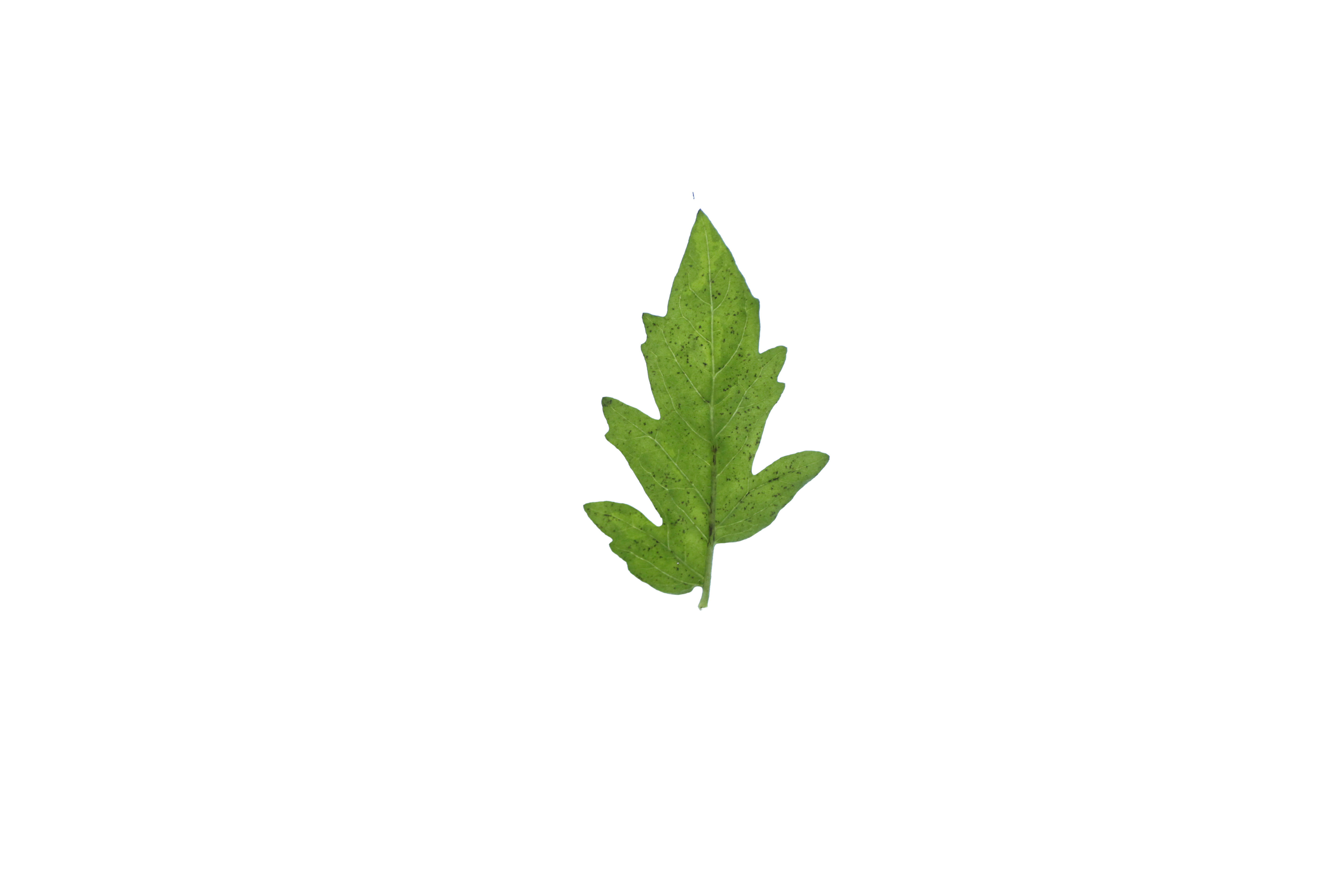

Supplement: Supplementary file 14 — Source data Fig. 5 [file 44318_2024_278_MOESM14_ESM.zip › Figure 5C/7_OEPSKR1 _TRVBRAK _H2O.jpg]

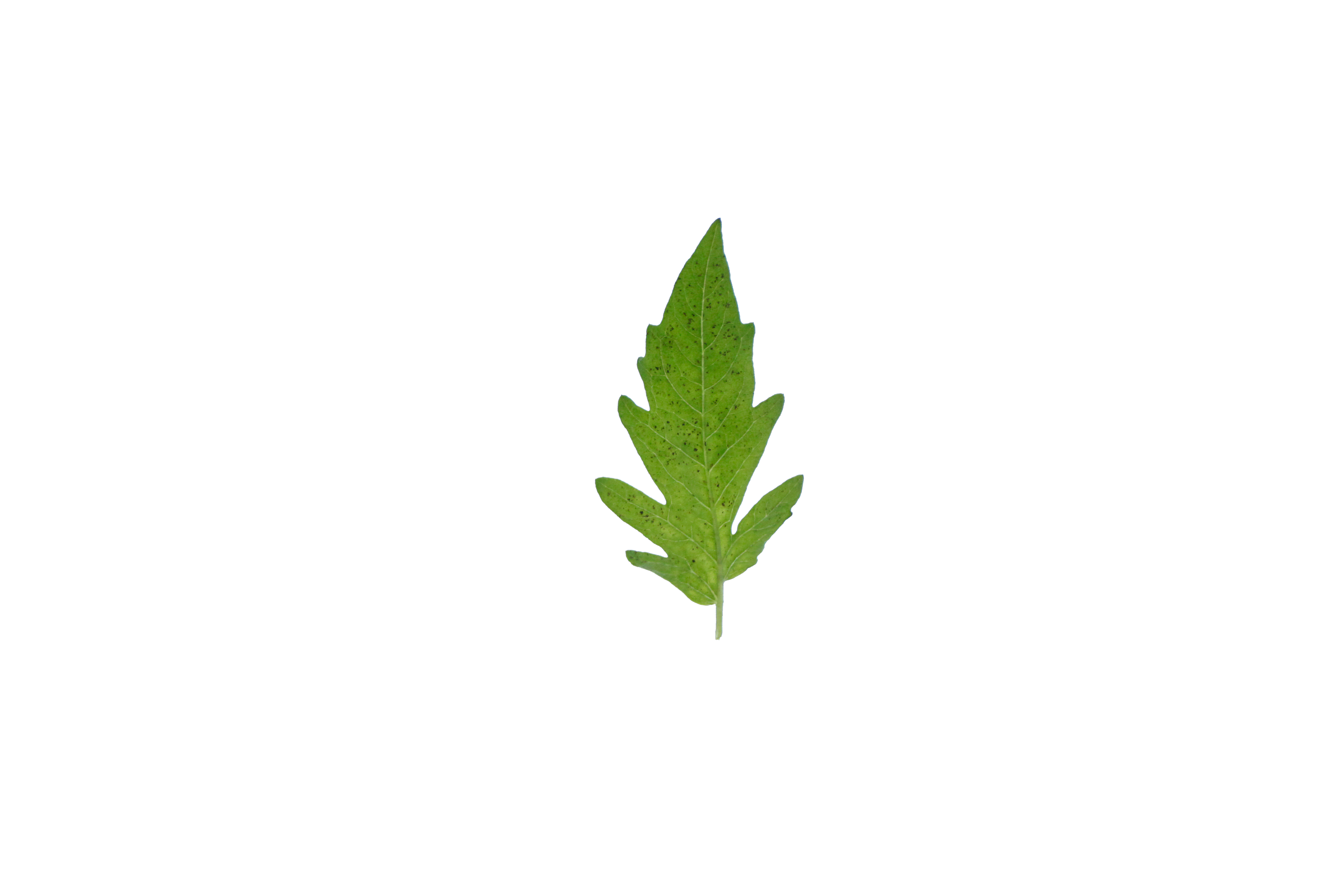

Supplement: Supplementary file 14 — Source data Fig. 5 [file 44318_2024_278_MOESM14_ESM.zip › Figure 5C/8_OEPSKR1 _TRVBRAK _PSK.JPG]

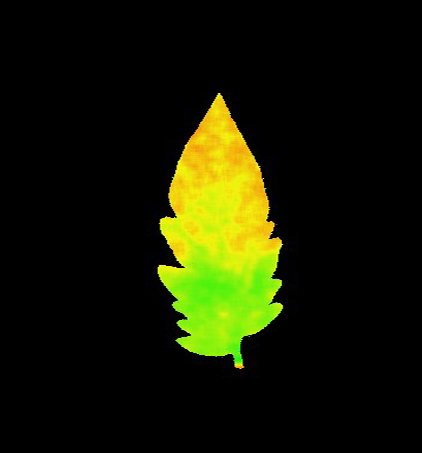

Supplement: Supplementary file 14 — Source data Fig. 5 [file 44318_2024_278_MOESM14_ESM.zip › Figure 5D/1_WT_TRV0 _H2O.jpg]

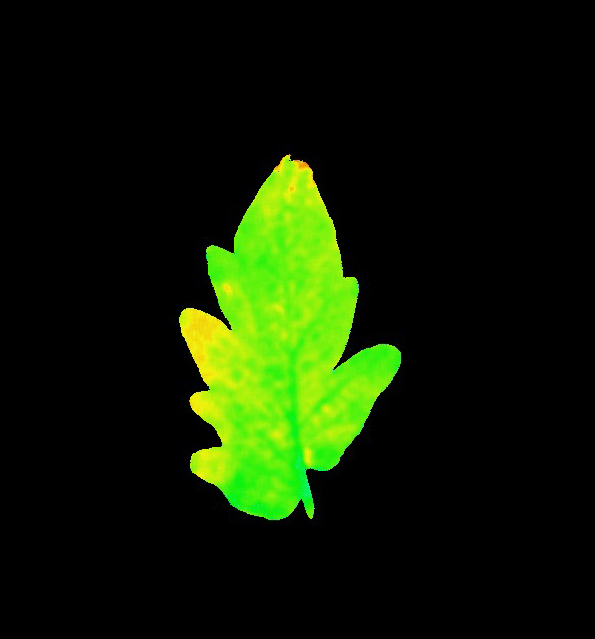

Supplement: Supplementary file 14 — Source data Fig. 5 [file 44318_2024_278_MOESM14_ESM.zip › Figure 5D/2_WT_TRV0 _PSK.jpg]

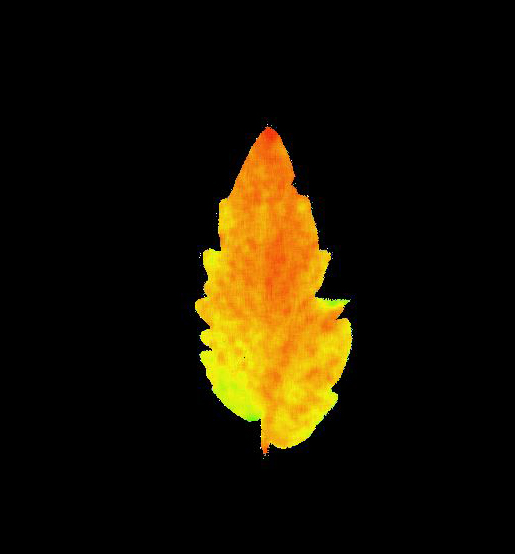

Supplement: Supplementary file 14 — Source data Fig. 5 [file 44318_2024_278_MOESM14_ESM.zip › Figure 5D/3_WT_TRVBRAK _H2O.jpg]

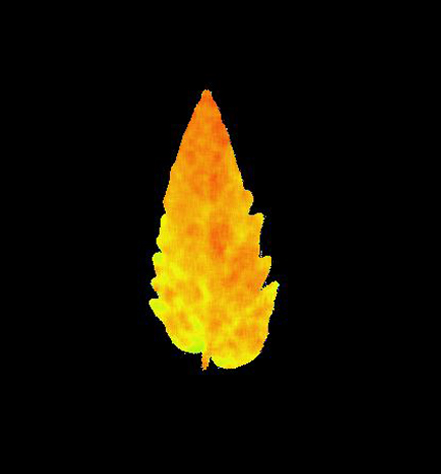

Supplement: Supplementary file 14 — Source data Fig. 5 [file 44318_2024_278_MOESM14_ESM.zip › Figure 5D/4_WT_TRVBRAK _PSK.jpg]

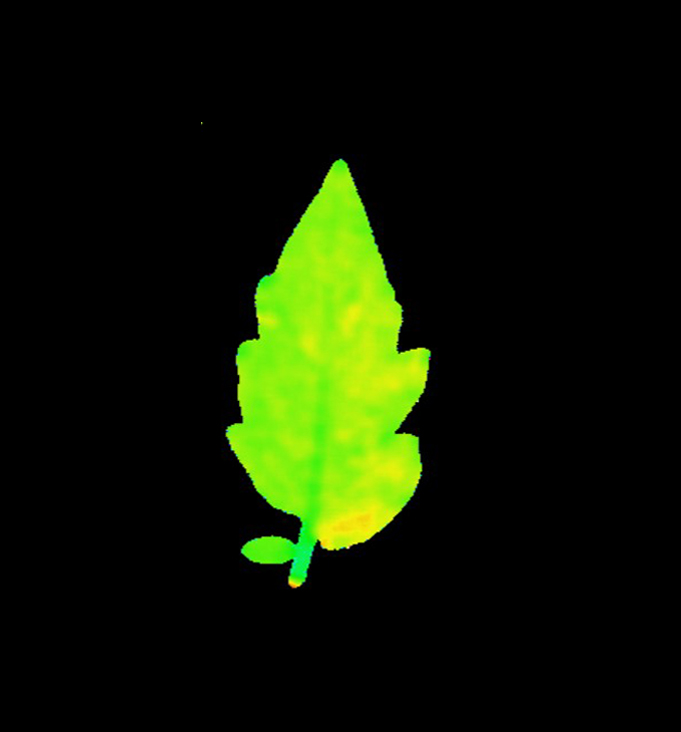

Supplement: Supplementary file 14 — Source data Fig. 5 [file 44318_2024_278_MOESM14_ESM.zip › Figure 5D/5_OEPSKR1_TRV0_H2O.jpg]

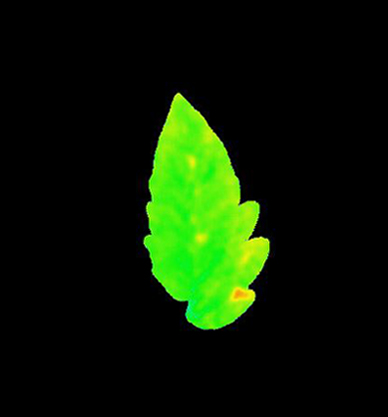

Supplement: Supplementary file 14 — Source data Fig. 5 [file 44318_2024_278_MOESM14_ESM.zip › Figure 5D/6_OEPSKR1_TRV0_PSK.jpg]

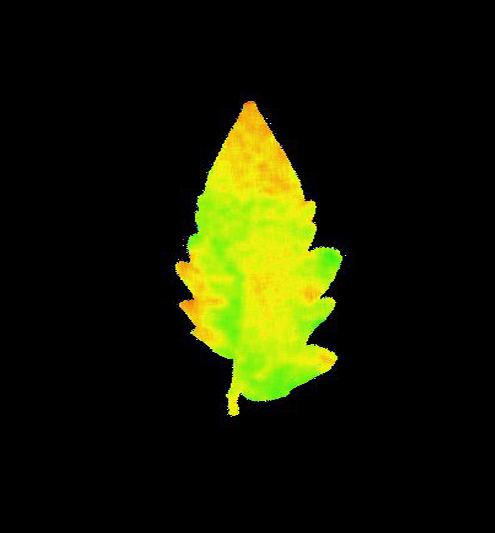

Supplement: Supplementary file 14 — Source data Fig. 5 [file 44318_2024_278_MOESM14_ESM.zip › Figure 5D/7_OEPSKR1 _TRVBRAK _H2O.jpg]

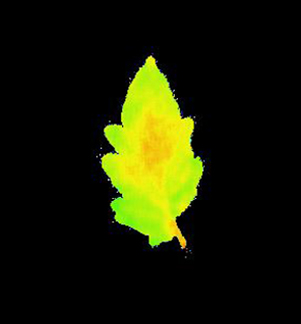

Supplement: Supplementary file 14 — Source data Fig. 5 [file 44318_2024_278_MOESM14_ESM.zip › Figure 5D/8_OEPSKR1 _TRVBRAK _PSK.jpg]

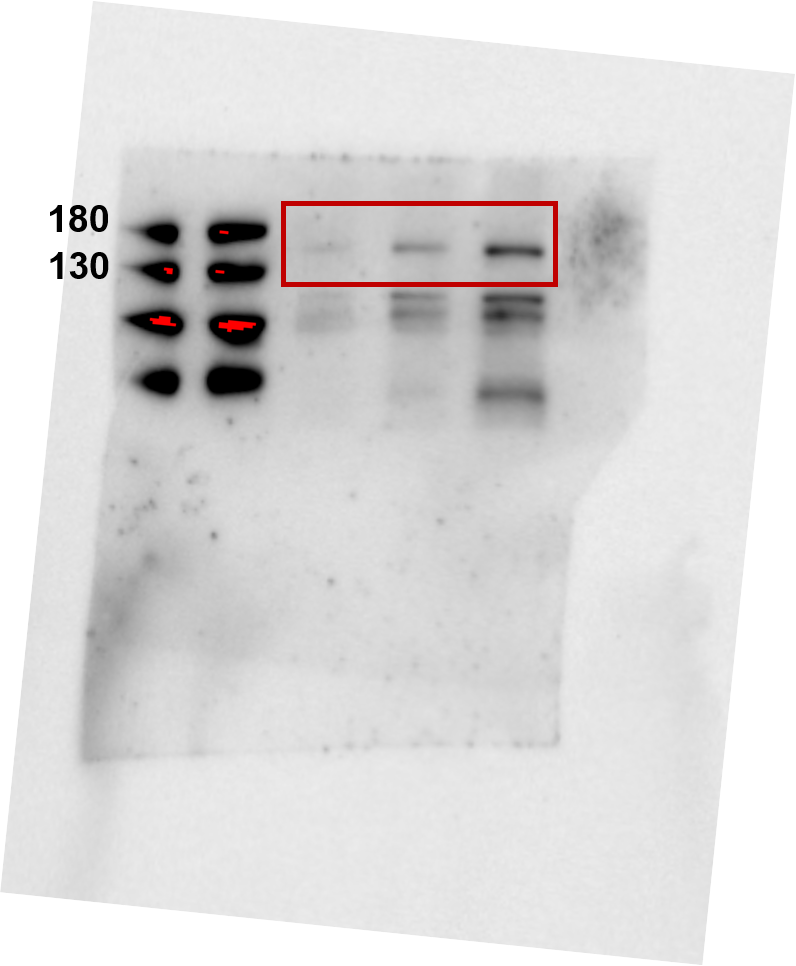

Supplement: Supplementary file 15 — Source data Fig. 6 [file 44318_2024_278_MOESM15_ESM.zip › Figure 6A/1-IP-GFP-anti-pSer-Thr.tif]

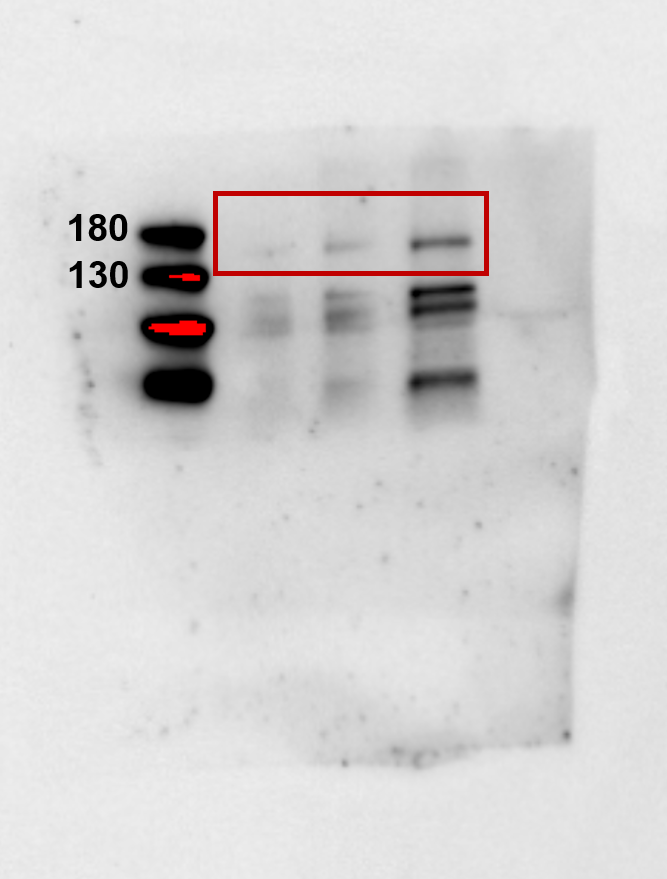

Supplement: Supplementary file 15 — Source data Fig. 6 [file 44318_2024_278_MOESM15_ESM.zip › Figure 6A/2-IP-GFP-anti-pTyr.tif]

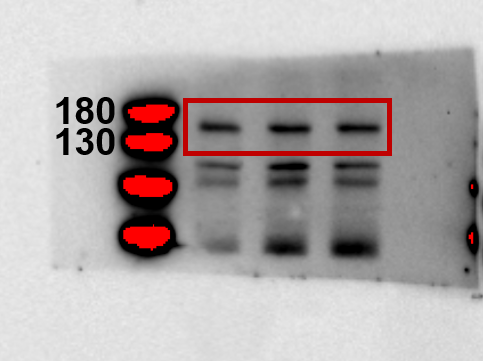

Supplement: Supplementary file 15 — Source data Fig. 6 [file 44318_2024_278_MOESM15_ESM.zip › Figure 6A/3-IP-GFP-anti-GFP.tif]

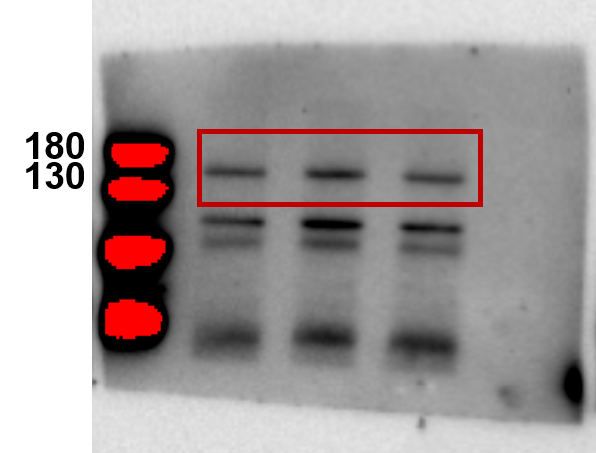

Supplement: Supplementary file 15 — Source data Fig. 6 [file 44318_2024_278_MOESM15_ESM.zip › Figure 6A/4-Input-anti-GFP.tif]

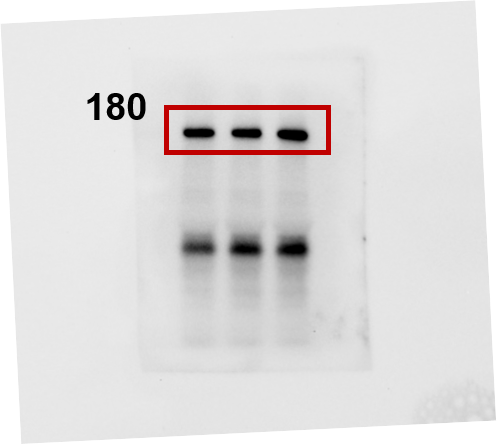

Supplement: Supplementary file 15 — Source data Fig. 6 [file 44318_2024_278_MOESM15_ESM.zip › Figure 6A/5-Input-anti-HA.tif]

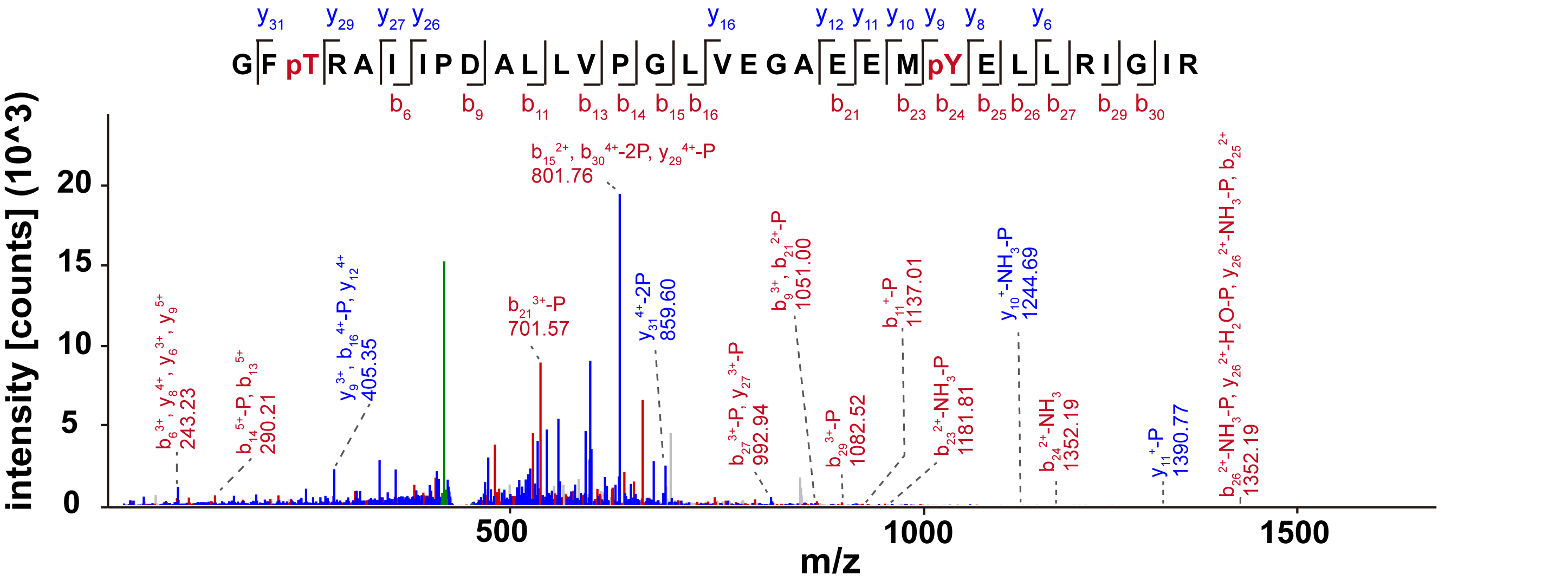

Supplement: Supplementary file 15 — Source data Fig. 6 [file 44318_2024_278_MOESM15_ESM.zip › Figure 6B/6B.tif]

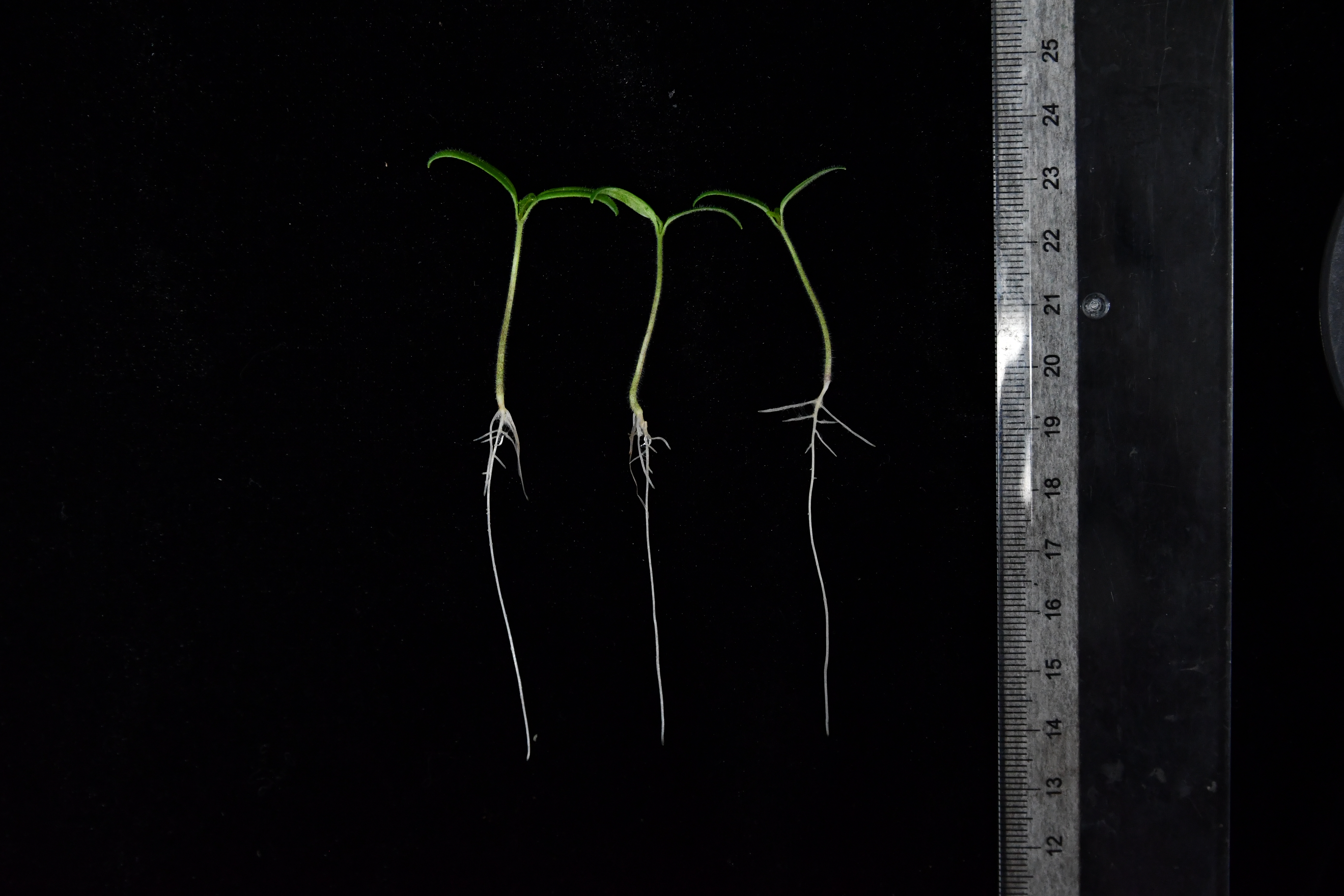

Supplement: Supplementary file 15 — Source data Fig. 6 [file 44318_2024_278_MOESM15_ESM.zip › Figure 6D/1_YFP_brak_H2O.JPG]

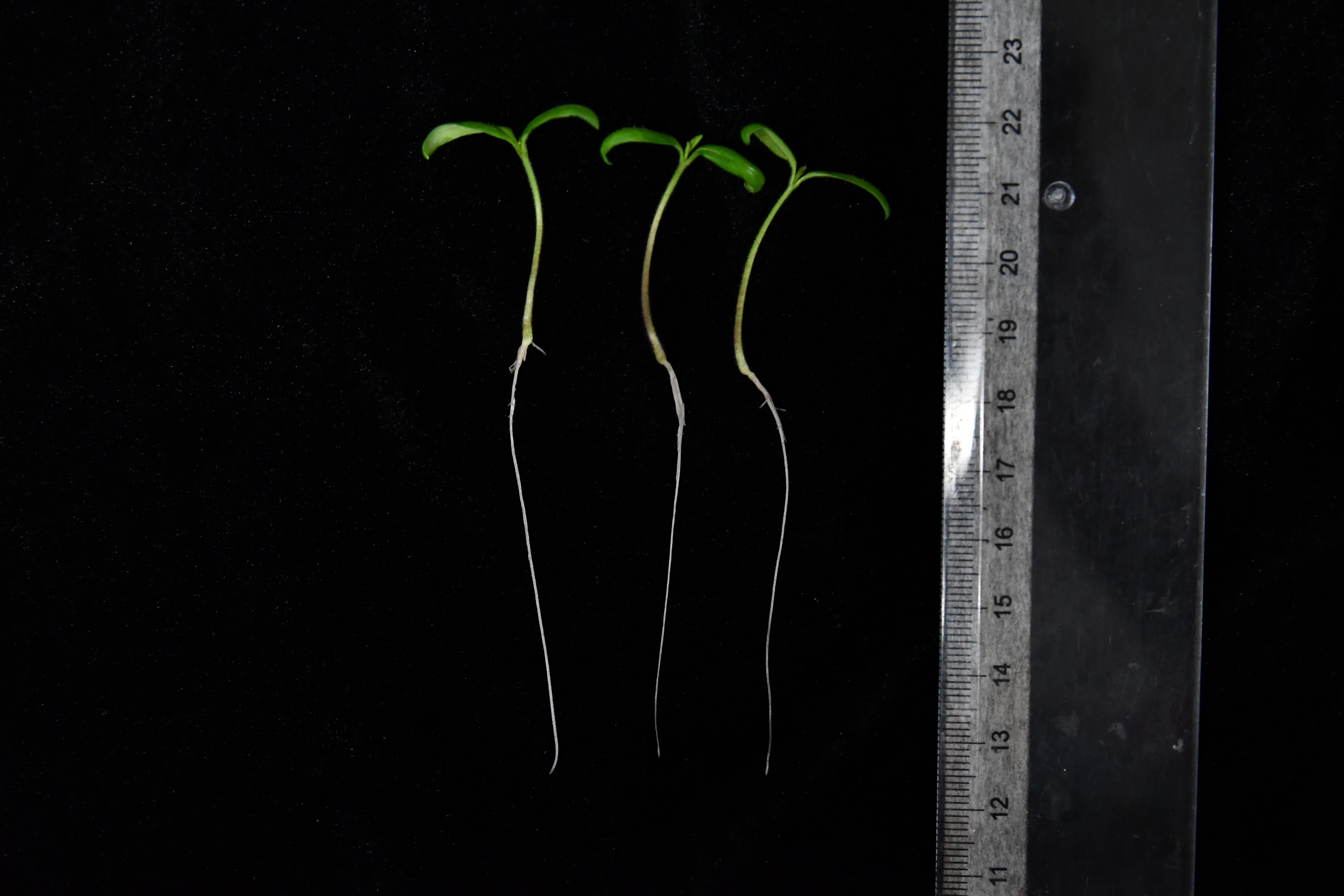

Supplement: Supplementary file 15 — Source data Fig. 6 [file 44318_2024_278_MOESM15_ESM.zip › Figure 6D/2_YFP_brak_PSK.JPG]

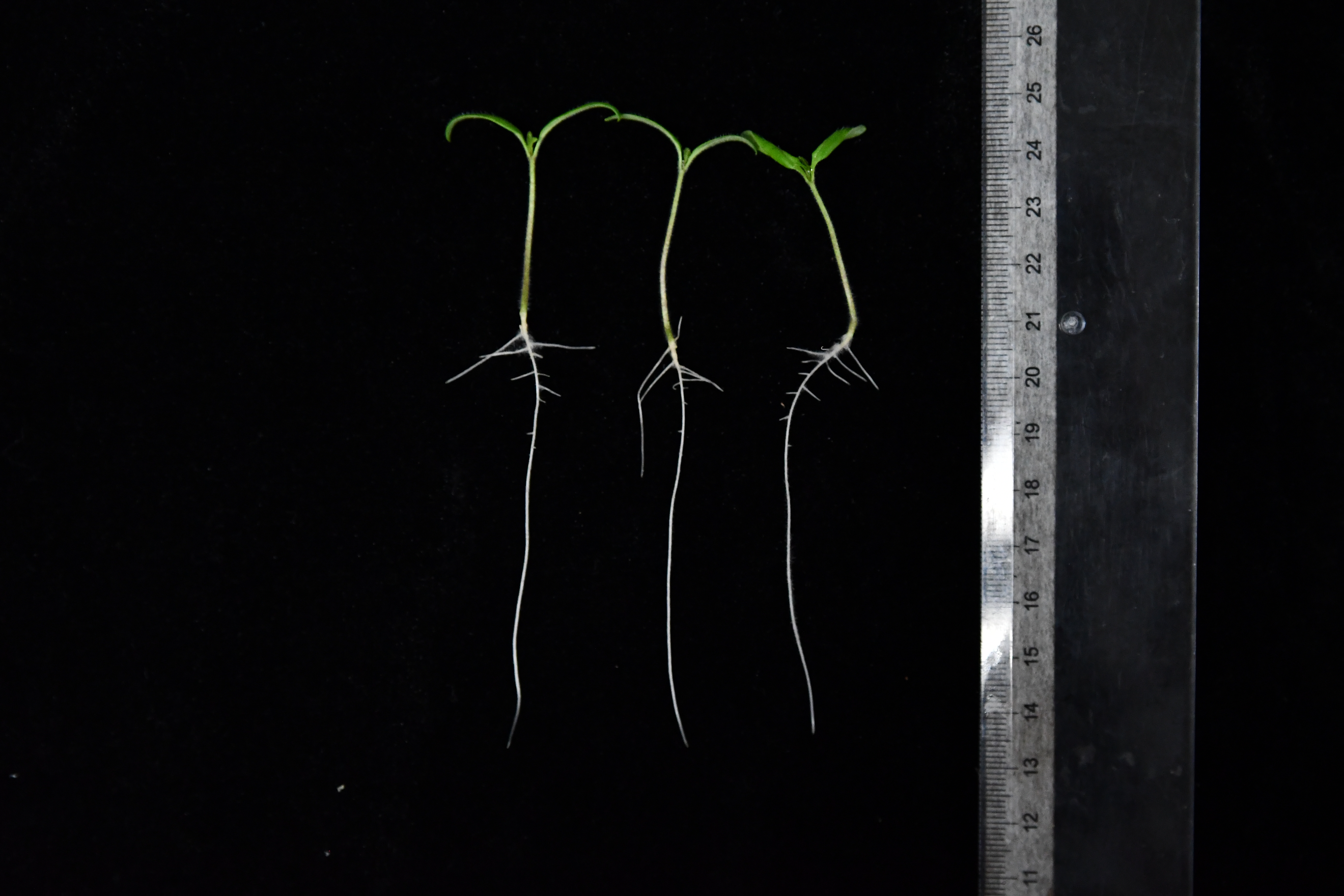

Supplement: Supplementary file 15 — Source data Fig. 6 [file 44318_2024_278_MOESM15_ESM.zip › Figure 6D/3_BRAK_brak_H2O.JPG]

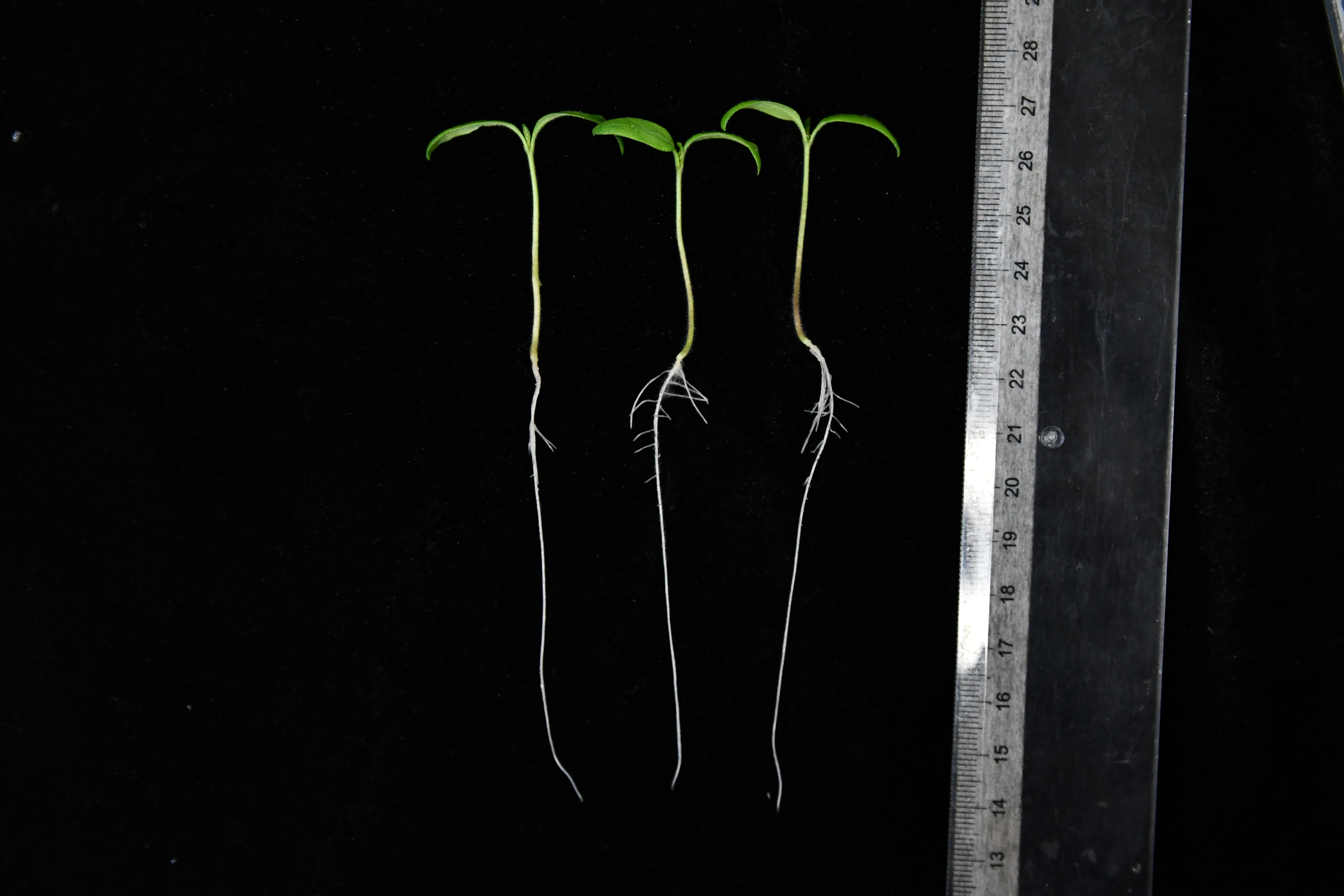

Supplement: Supplementary file 15 — Source data Fig. 6 [file 44318_2024_278_MOESM15_ESM.zip › Figure 6D/4_BRAK_brak_PSK.JPG]

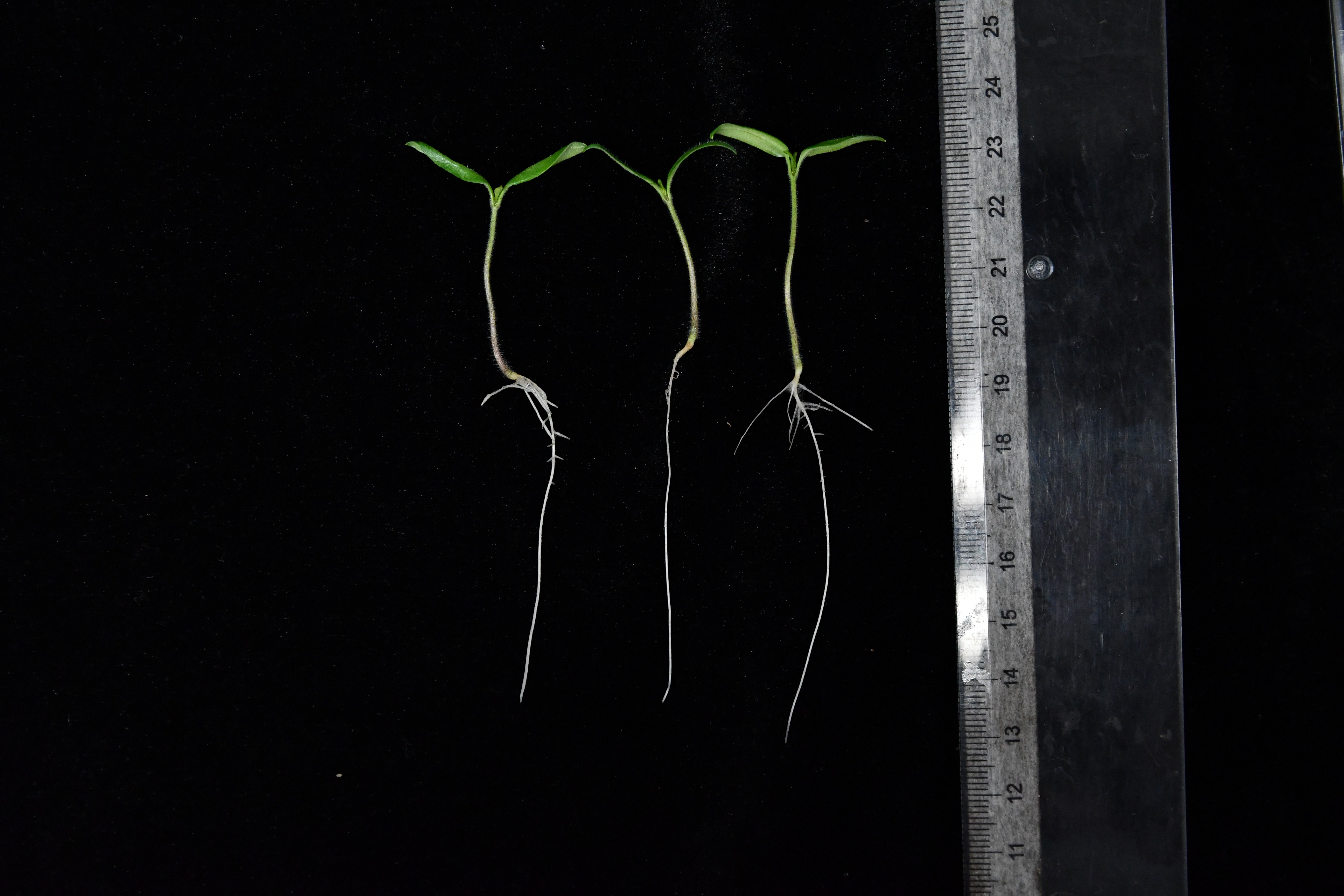

Supplement: Supplementary file 15 — Source data Fig. 6 [file 44318_2024_278_MOESM15_ESM.zip › Figure 6D/5_T1027A_brak_H2O.JPG]

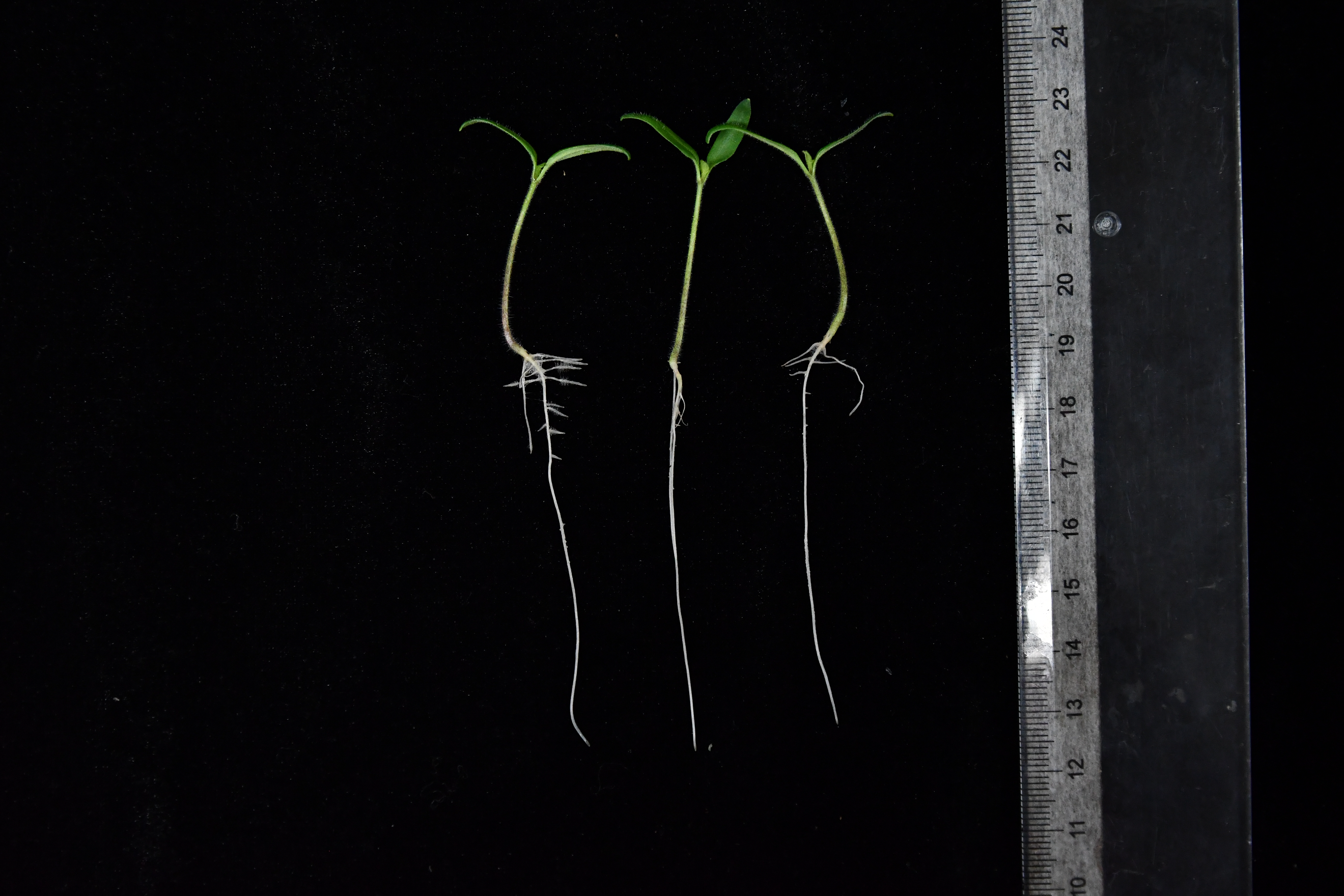

Supplement: Supplementary file 15 — Source data Fig. 6 [file 44318_2024_278_MOESM15_ESM.zip › Figure 6D/6_T1027A_brak_PSK.JPG]

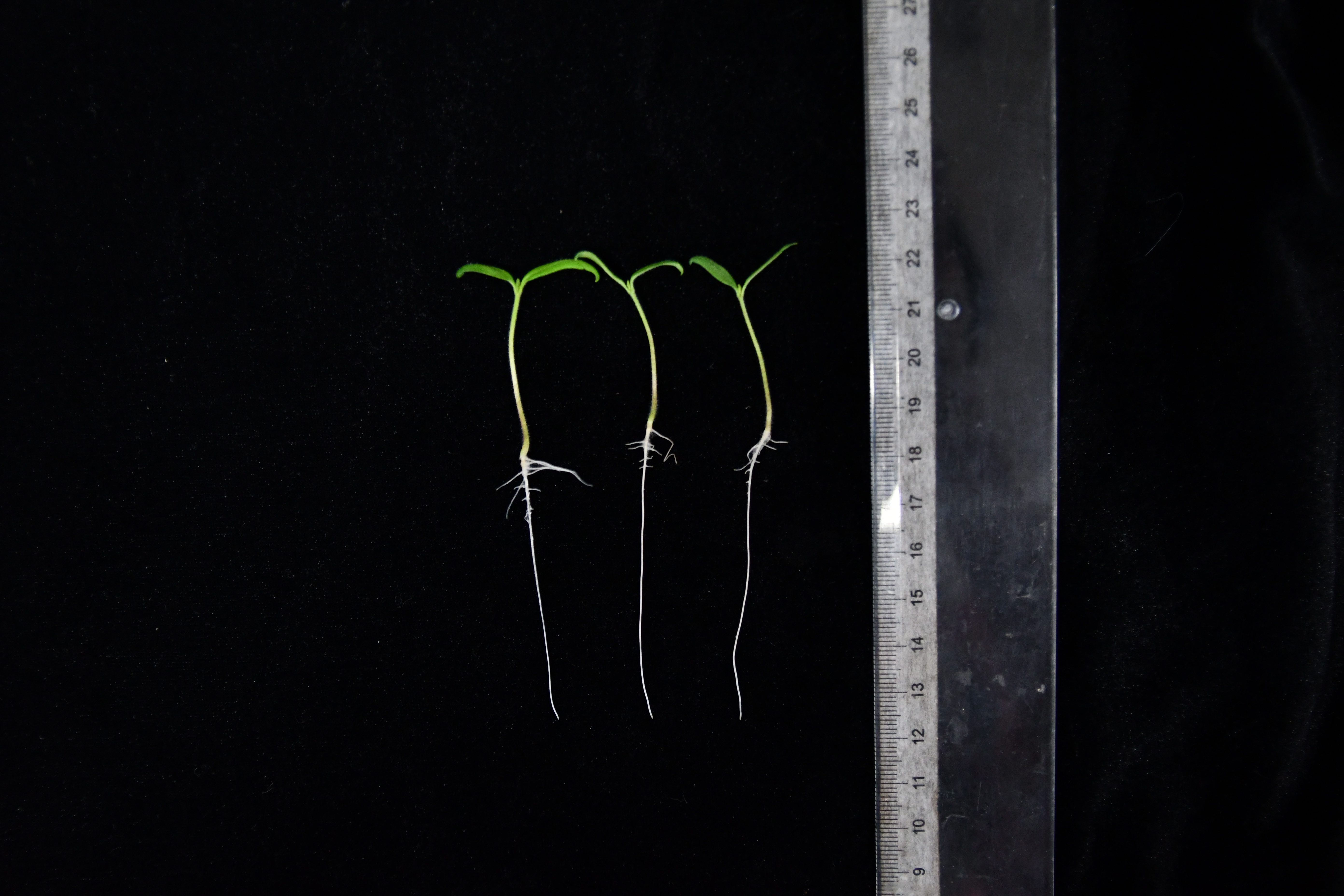

Supplement: Supplementary file 15 — Source data Fig. 6 [file 44318_2024_278_MOESM15_ESM.zip › Figure 6D/7_Y1048F_brak_H2O.JPG]

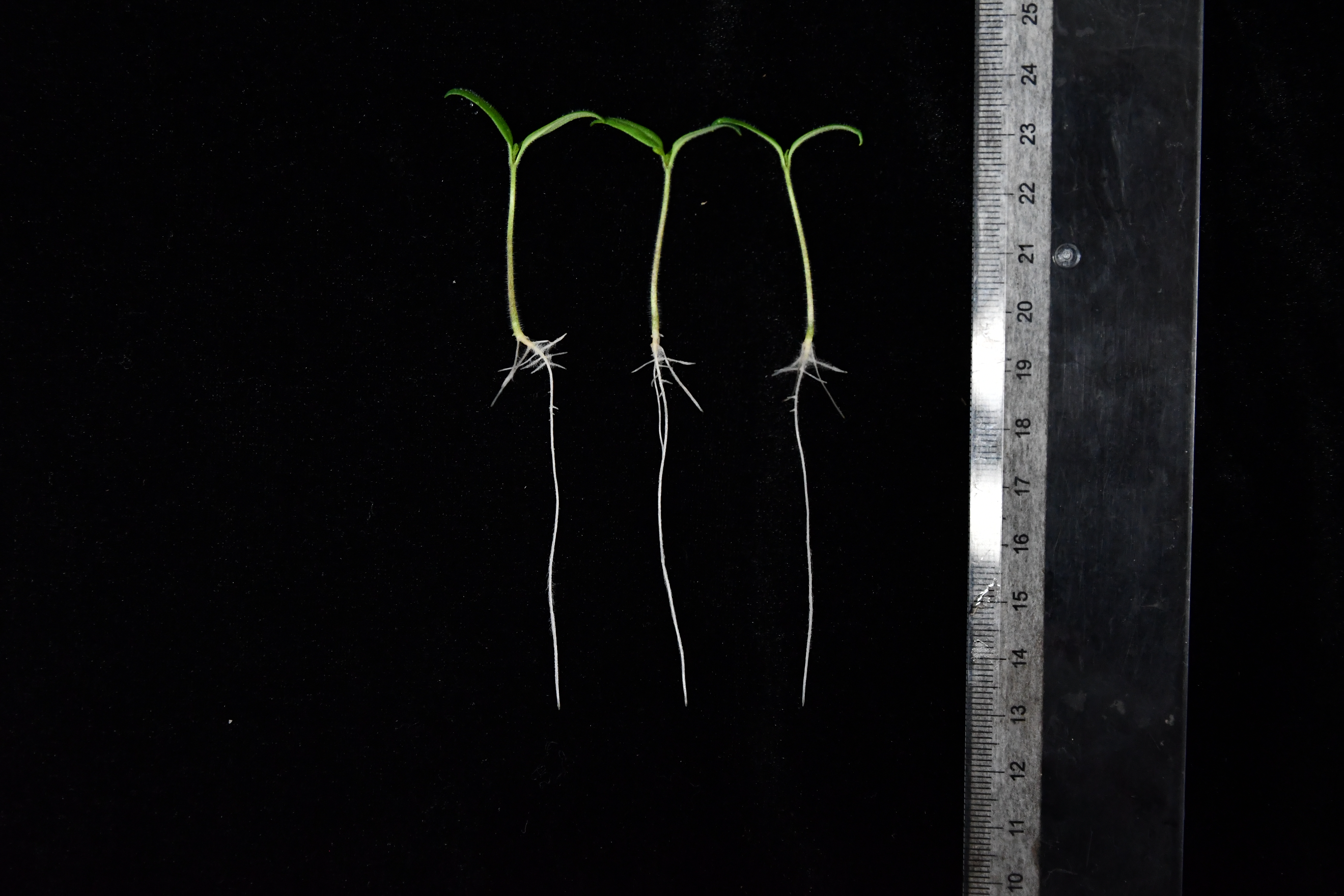

Supplement: Supplementary file 15 — Source data Fig. 6 [file 44318_2024_278_MOESM15_ESM.zip › Figure 6D/8_Y1048F_brak_PSK.JPG]

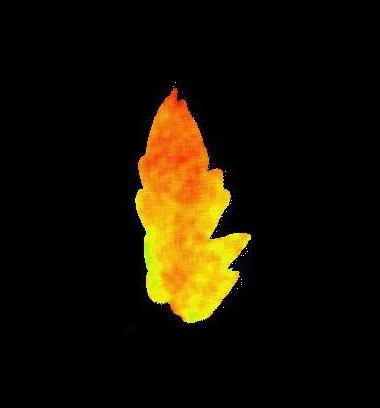

Supplement: Supplementary file 15 — Source data Fig. 6 [file 44318_2024_278_MOESM15_ESM.zip › Figure 6F/1_YFP_brak_H2O.jpg]

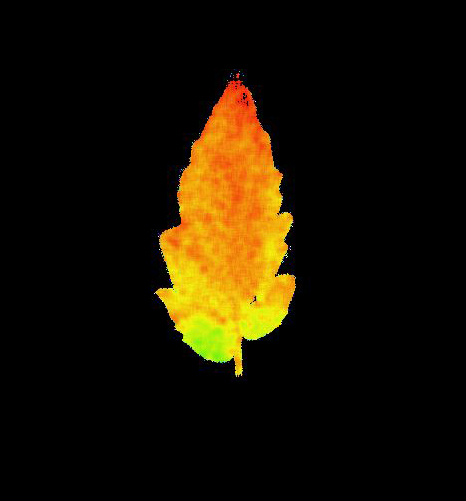

Supplement: Supplementary file 15 — Source data Fig. 6 [file 44318_2024_278_MOESM15_ESM.zip › Figure 6F/2_YFP_brak_PSK.jpg]

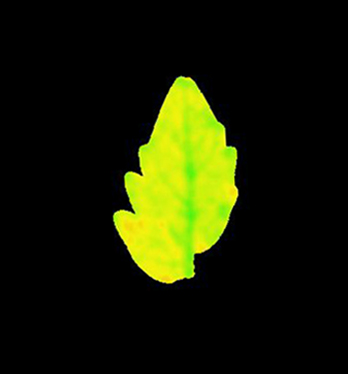

Supplement: Supplementary file 15 — Source data Fig. 6 [file 44318_2024_278_MOESM15_ESM.zip › Figure 6F/3_BRAK_brak_H2O.jpg]

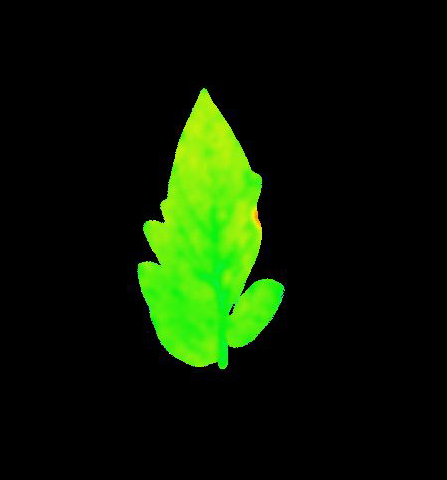

Supplement: Supplementary file 15 — Source data Fig. 6 [file 44318_2024_278_MOESM15_ESM.zip › Figure 6F/4_BRAK_brak_PSK.jpg]

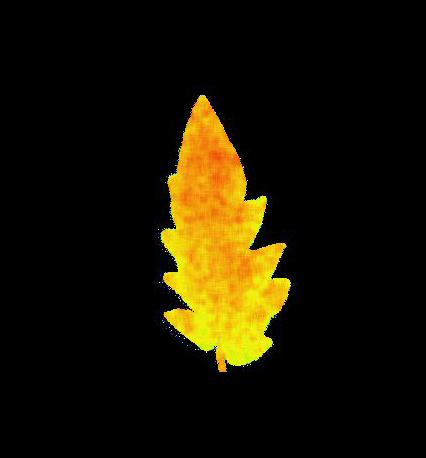

Supplement: Supplementary file 15 — Source data Fig. 6 [file 44318_2024_278_MOESM15_ESM.zip › Figure 6F/5_T1027A_brak_H2O.jpg]

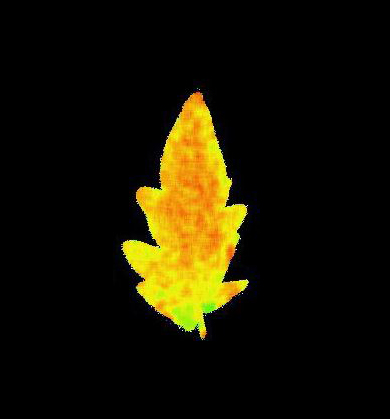

Supplement: Supplementary file 15 — Source data Fig. 6 [file 44318_2024_278_MOESM15_ESM.zip › Figure 6F/6_T1027A_brak_PSK.jpg]

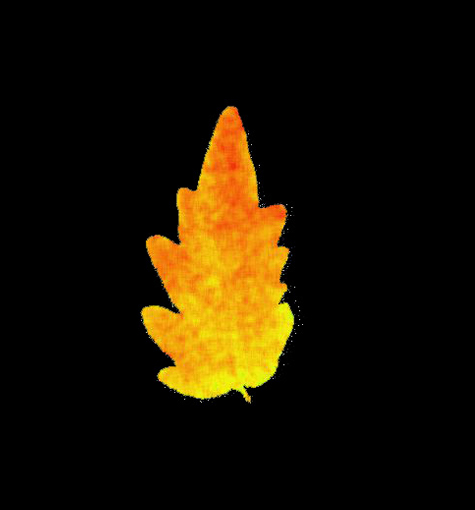

Supplement: Supplementary file 15 — Source data Fig. 6 [file 44318_2024_278_MOESM15_ESM.zip › Figure 6F/7_Y1048F_brak_H2O.jpg]

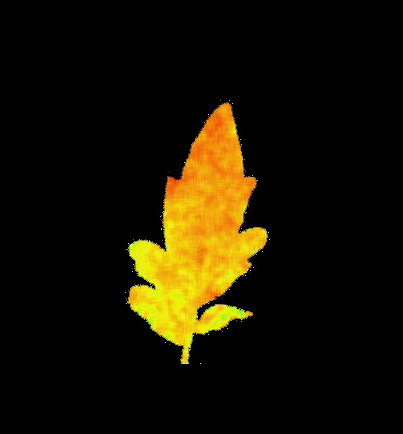

Supplement: Supplementary file 15 — Source data Fig. 6 [file 44318_2024_278_MOESM15_ESM.zip › Figure 6F/8_Y1048F_brak_PSK.jpg]

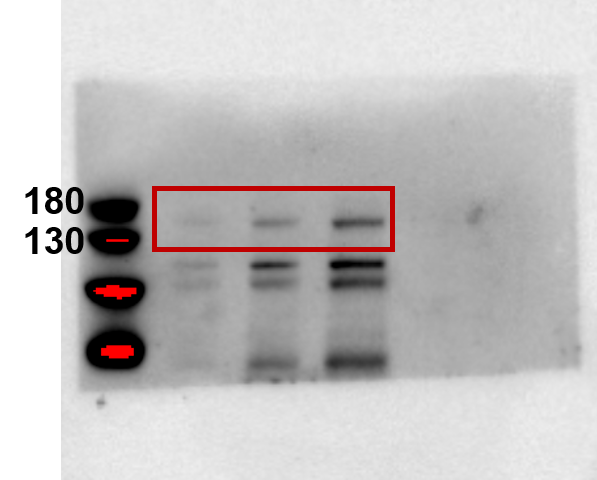

Supplement: Supplementary file 16 — Source data Fig. 7 [file 44318_2024_278_MOESM16_ESM.zip › Figure 7A/1. IP-anti-pSer-Thr.tif]

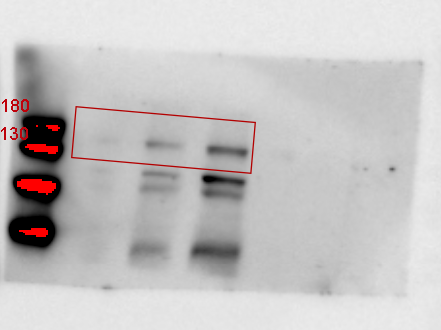

Supplement: Supplementary file 16 — Source data Fig. 7 [file 44318_2024_278_MOESM16_ESM.zip › Figure 7A/2. IP-anti-pTyr-1.tif]

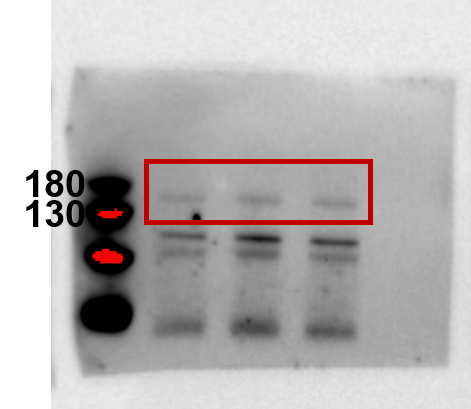

Supplement: Supplementary file 16 — Source data Fig. 7 [file 44318_2024_278_MOESM16_ESM.zip › Figure 7A/3. IP-anti-GFP.tif]

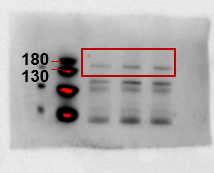

Supplement: Supplementary file 16 — Source data Fig. 7 [file 44318_2024_278_MOESM16_ESM.zip › Figure 7A/4. Input-anti-GFP.tif]

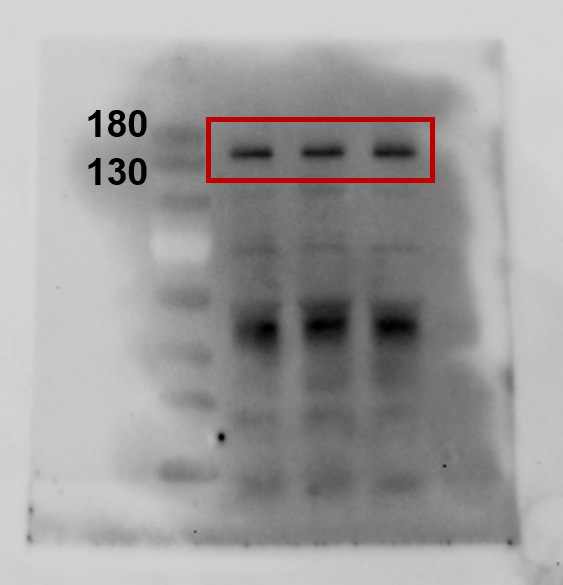

Supplement: Supplementary file 16 — Source data Fig. 7 [file 44318_2024_278_MOESM16_ESM.zip › Figure 7A/5. Input-anti-HA.tif]

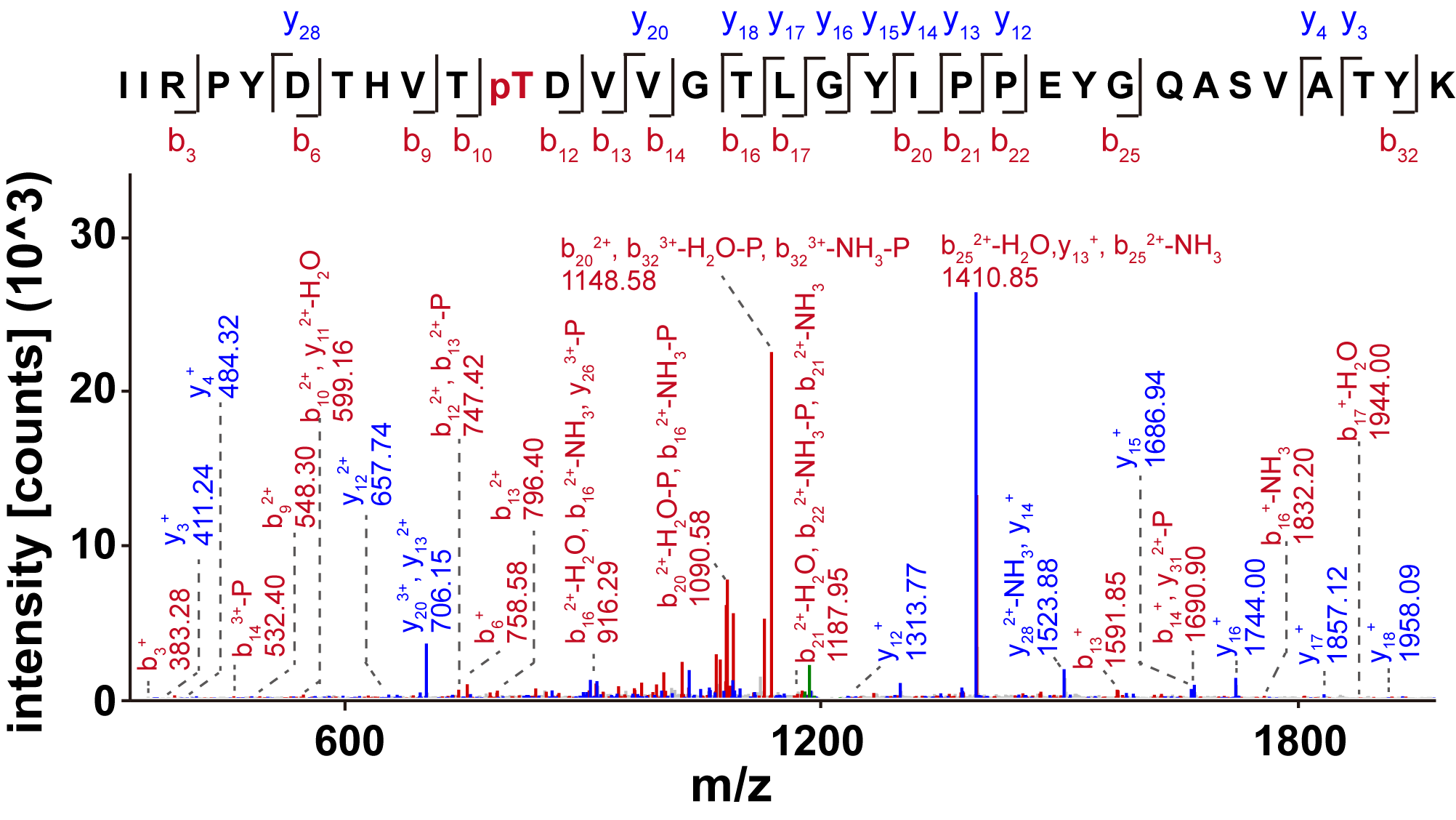

Supplement: Supplementary file 16 — Source data Fig. 7 [file 44318_2024_278_MOESM16_ESM.zip › Figure 7B/7B.tif]

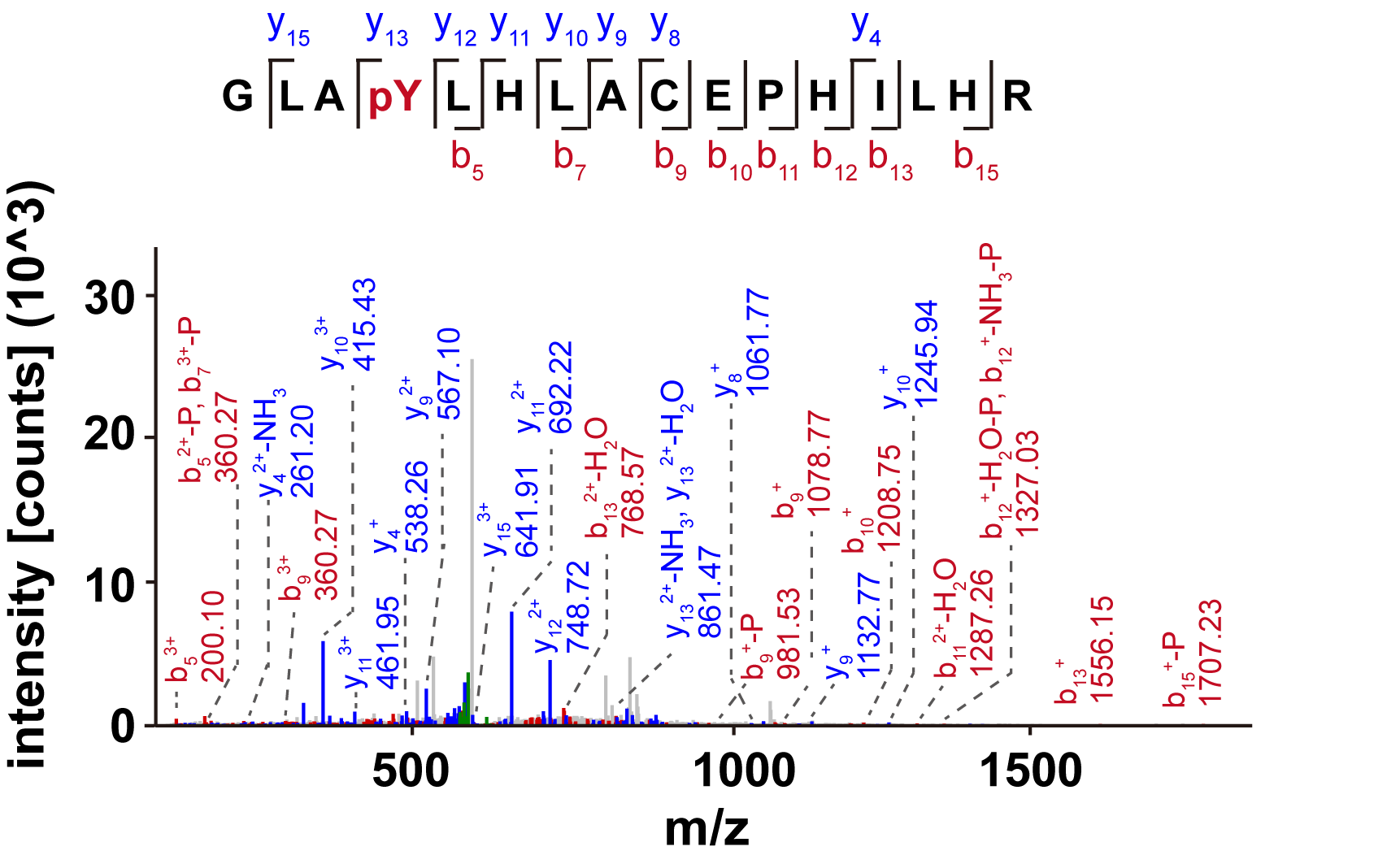

Supplement: Supplementary file 16 — Source data Fig. 7 [file 44318_2024_278_MOESM16_ESM.zip › Figure 7C/7C.tif]

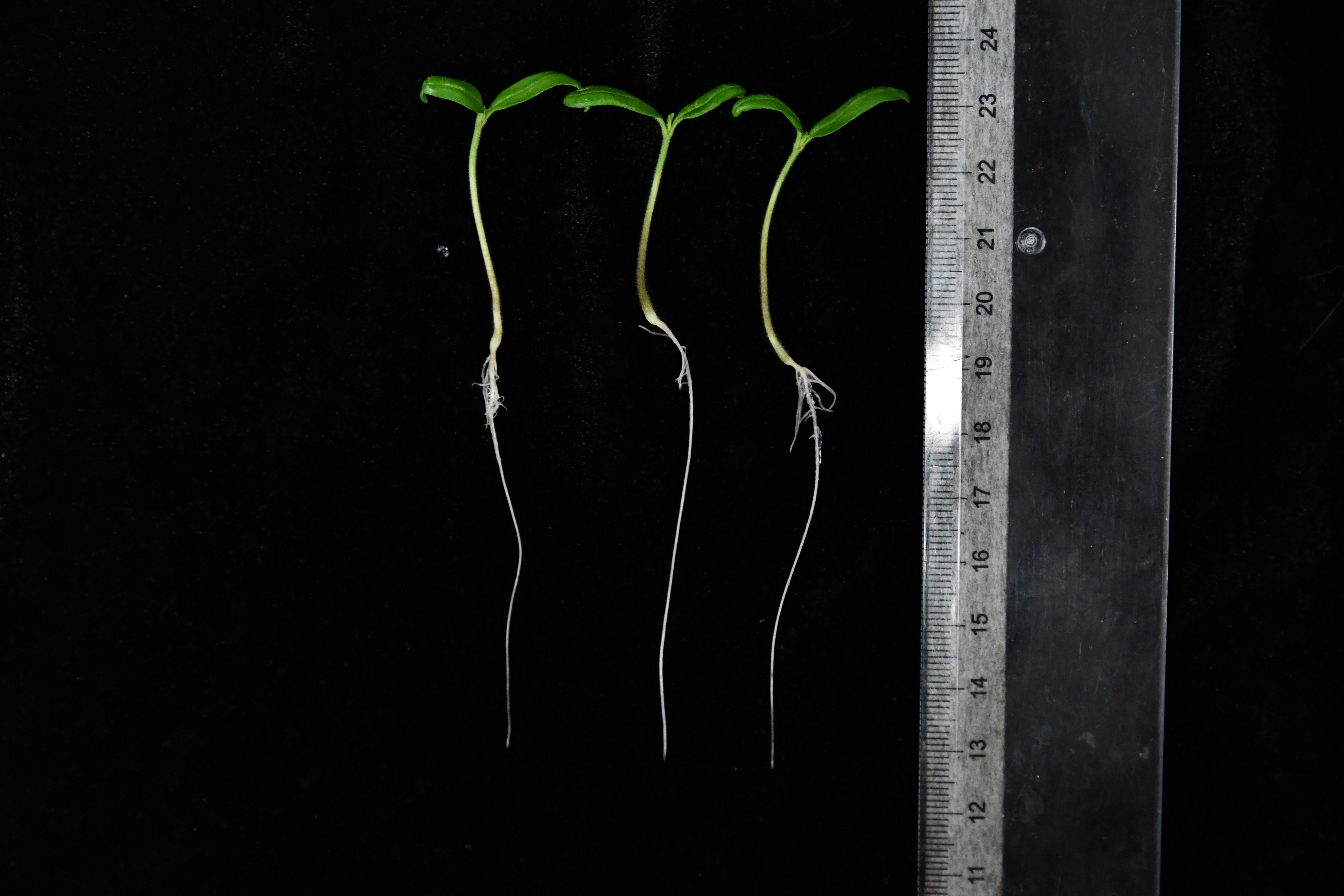

Supplement: Supplementary file 16 — Source data Fig. 7 [file 44318_2024_278_MOESM16_ESM.zip › Figure 7E/1_YFP_pskr1_H2O.JPG]

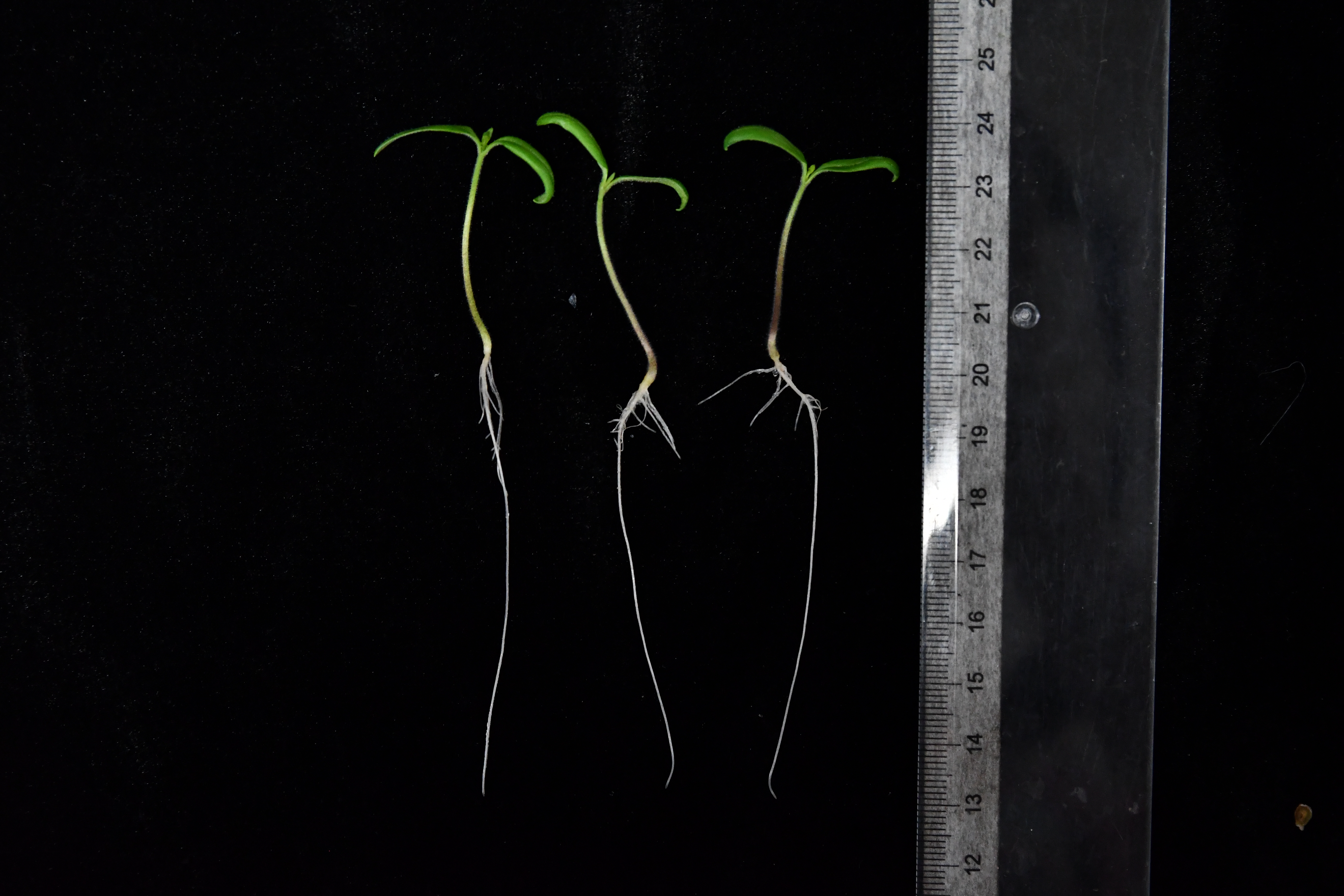

Supplement: Supplementary file 16 — Source data Fig. 7 [file 44318_2024_278_MOESM16_ESM.zip › Figure 7E/2_YFP_pskr1_PSK.JPG]

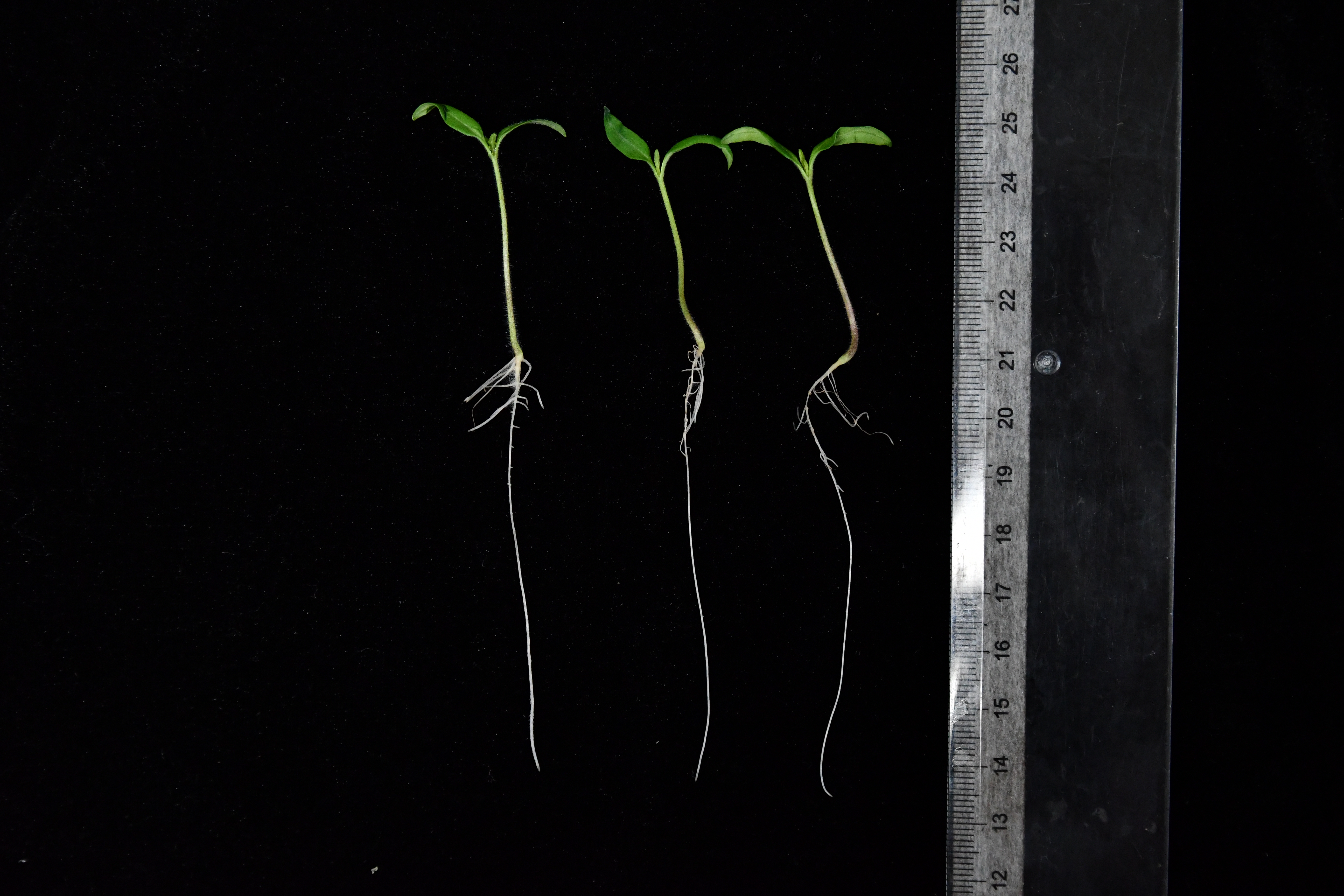

Supplement: Supplementary file 16 — Source data Fig. 7 [file 44318_2024_278_MOESM16_ESM.zip › Figure 7E/3_PSKR1_pskr1_H2O.JPG]

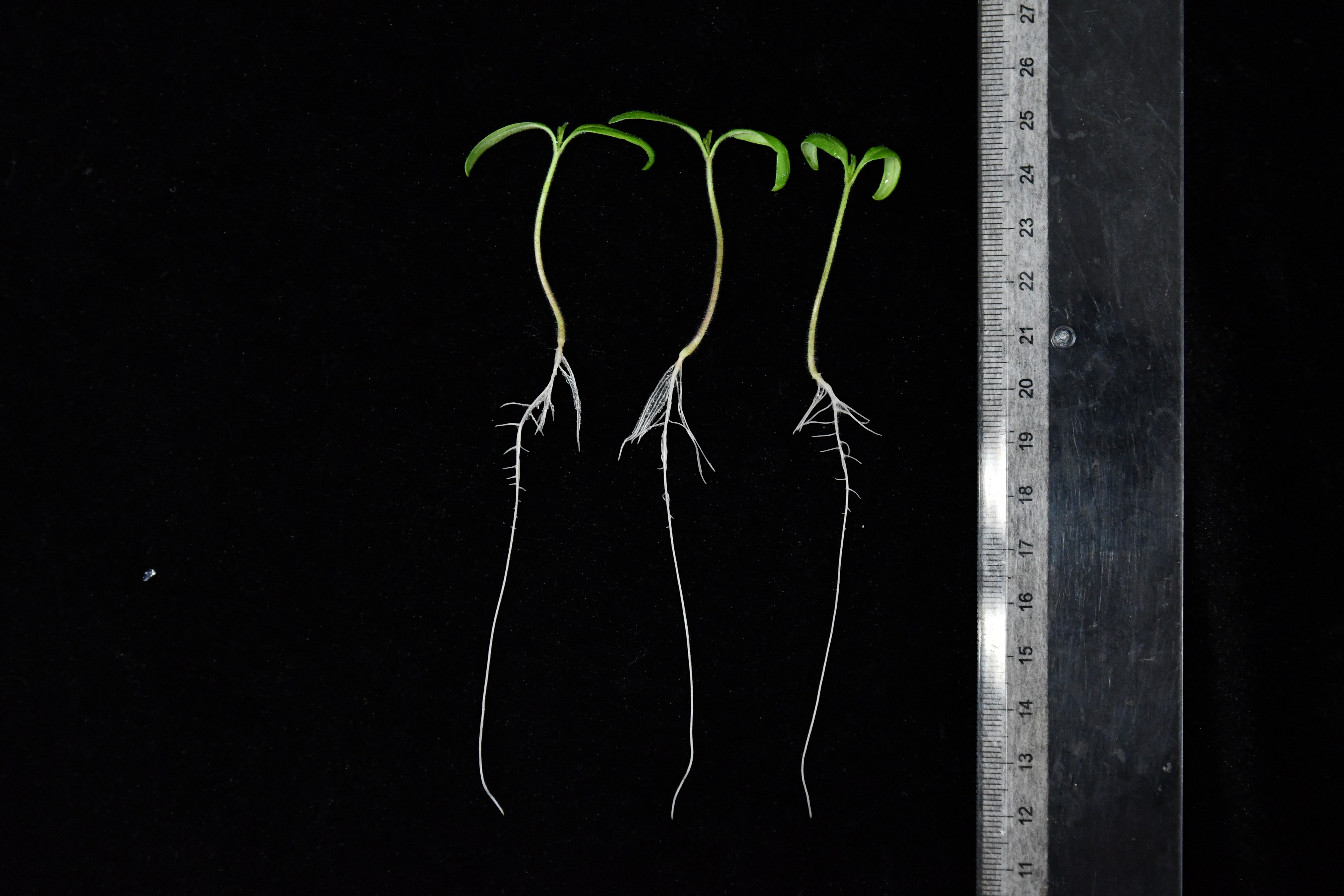

Supplement: Supplementary file 16 — Source data Fig. 7 [file 44318_2024_278_MOESM16_ESM.zip › Figure 7E/4_PSKR1_pskr1_PSK.JPG]

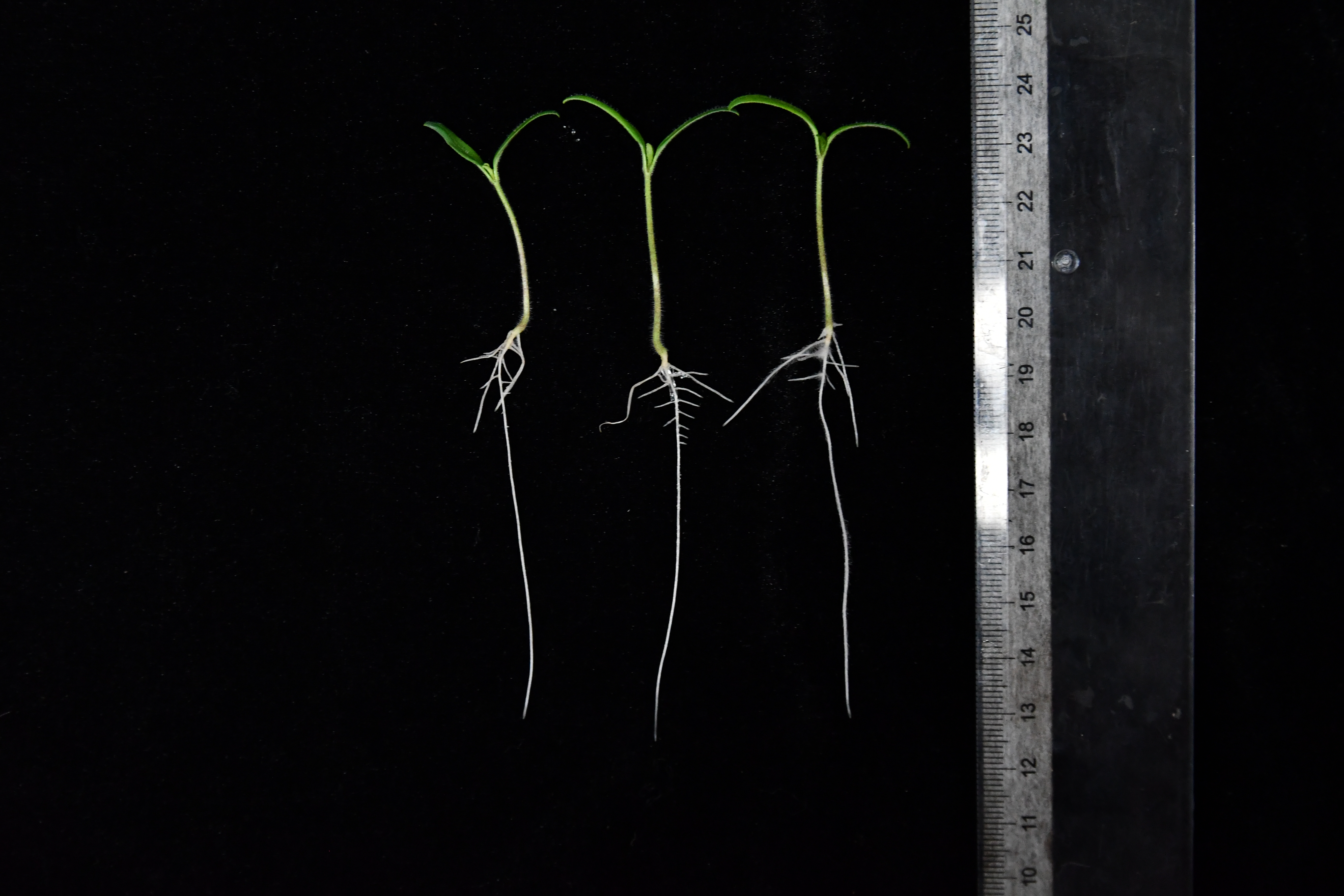

Supplement: Supplementary file 16 — Source data Fig. 7 [file 44318_2024_278_MOESM16_ESM.zip › Figure 7E/5_Y843F_pskr1_H2O.JPG]

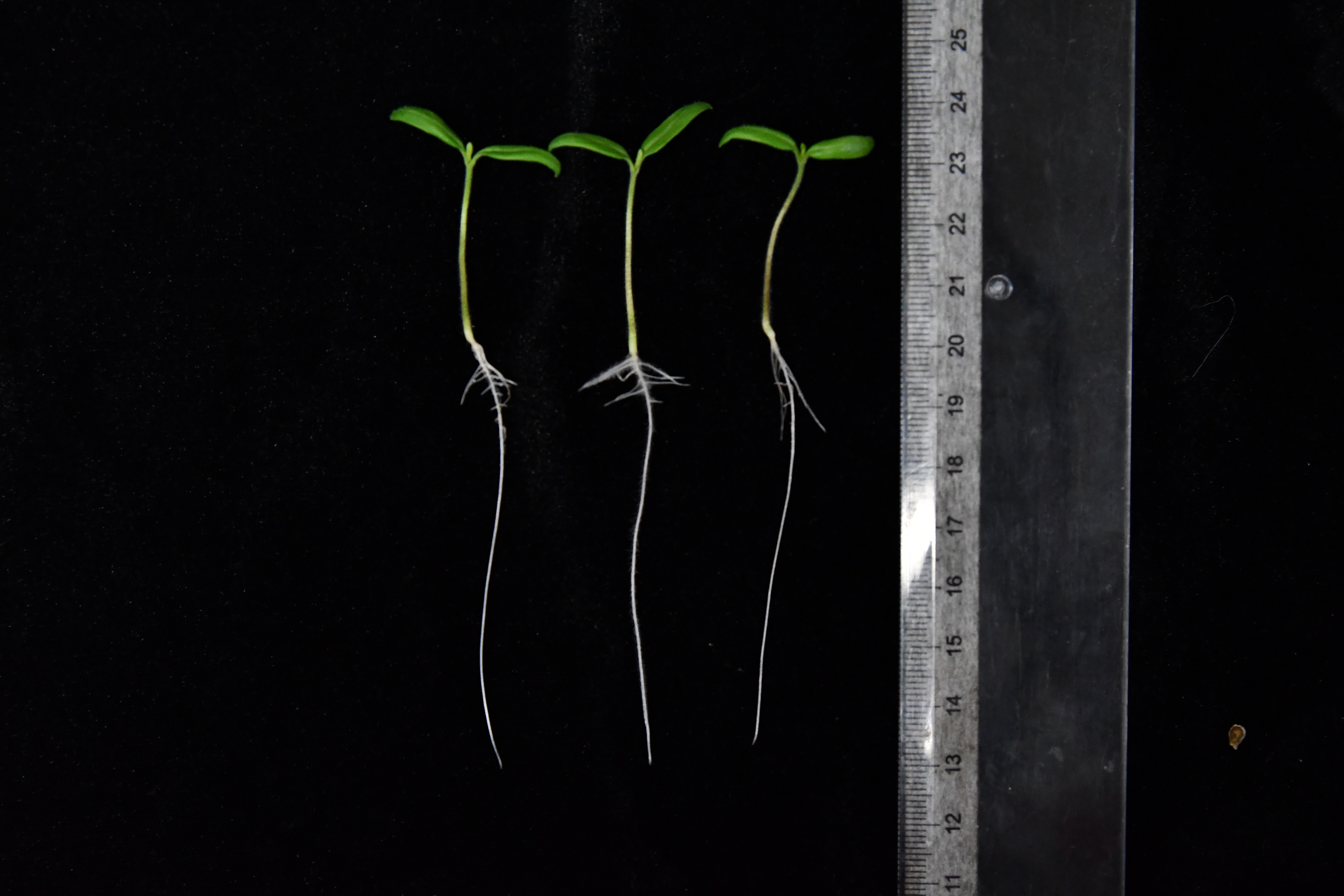

Supplement: Supplementary file 16 — Source data Fig. 7 [file 44318_2024_278_MOESM16_ESM.zip › Figure 7E/6_Y843F_pskr1_PSK.JPG]

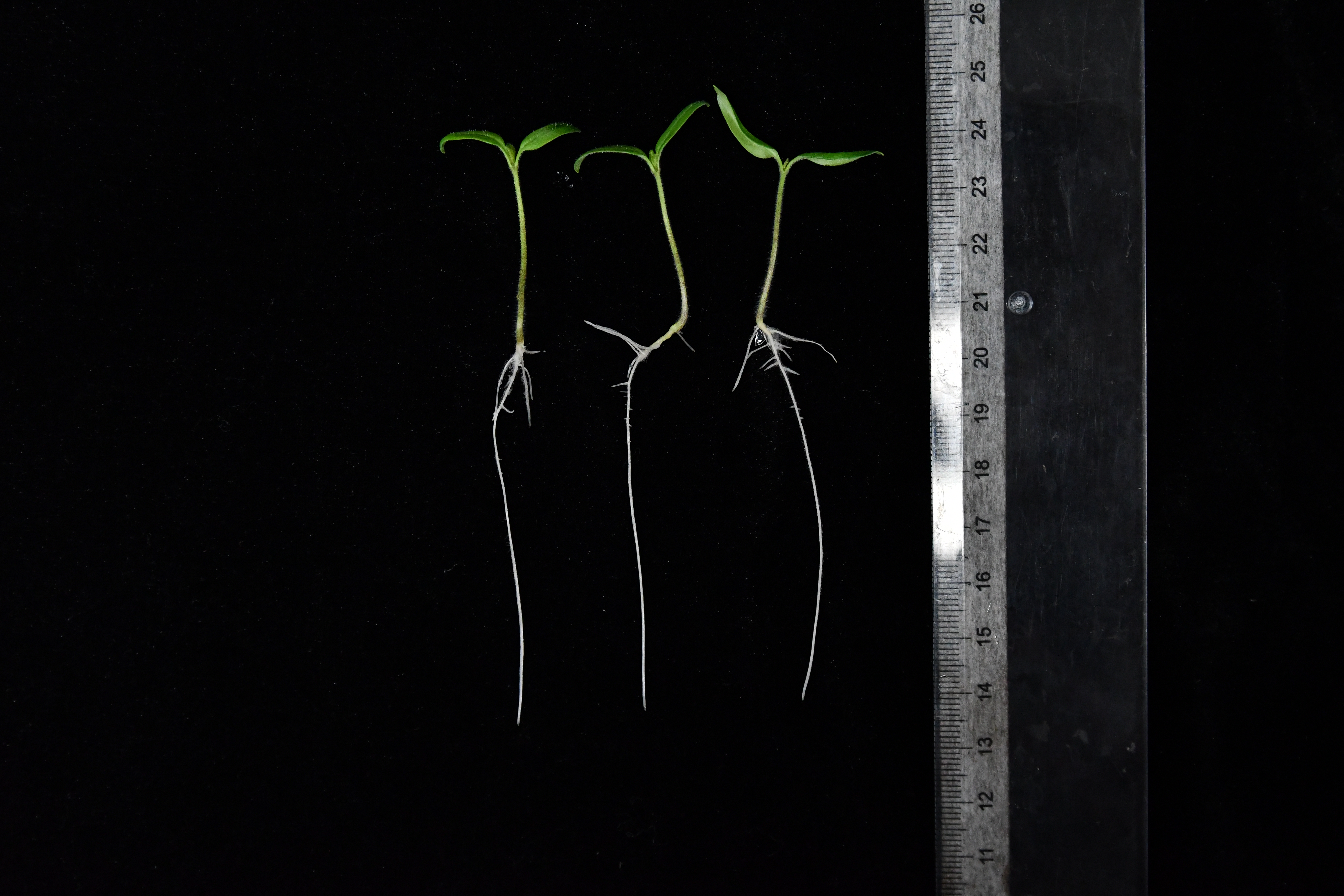

Supplement: Supplementary file 16 — Source data Fig. 7 [file 44318_2024_278_MOESM16_ESM.zip › Figure 7E/7_T890A_pskr1_H2O.JPG]

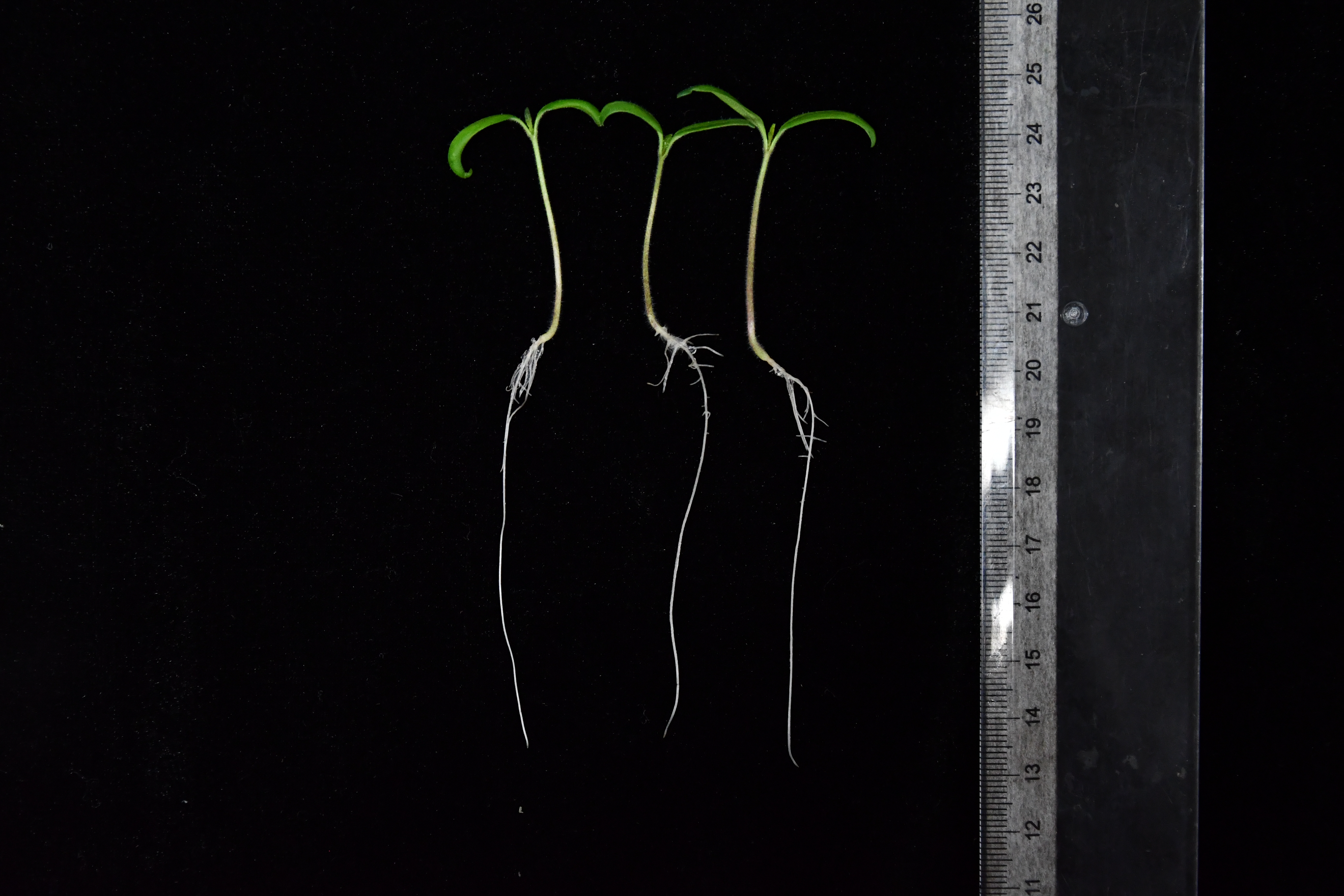

Supplement: Supplementary file 16 — Source data Fig. 7 [file 44318_2024_278_MOESM16_ESM.zip › Figure 7E/8_T890A_pskr1_PSK.JPG]

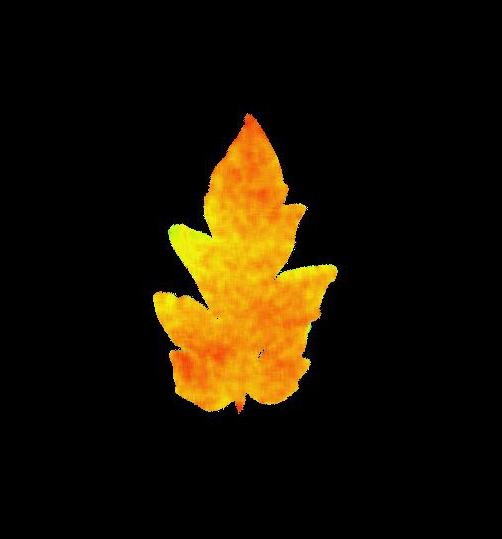

Supplement: Supplementary file 16 — Source data Fig. 7 [file 44318_2024_278_MOESM16_ESM.zip › Figure 7G/1_YFP_pskr1_H2O.jpg]

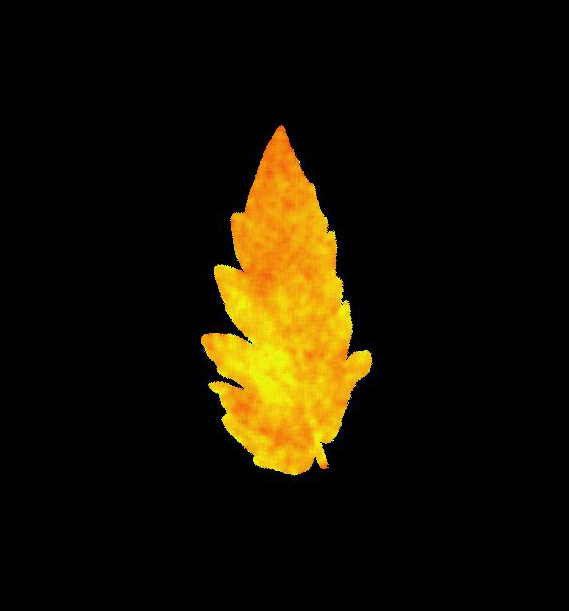

Supplement: Supplementary file 16 — Source data Fig. 7 [file 44318_2024_278_MOESM16_ESM.zip › Figure 7G/2_YFP_pskr1_PSK.jpg]

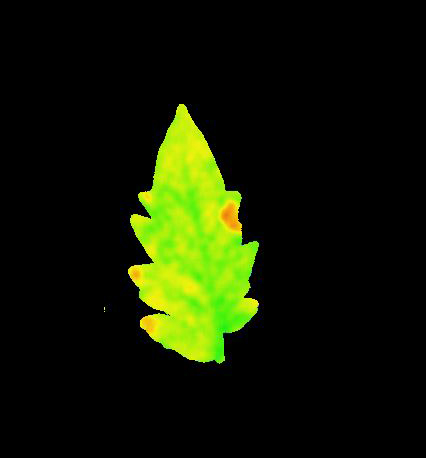

Supplement: Supplementary file 16 — Source data Fig. 7 [file 44318_2024_278_MOESM16_ESM.zip › Figure 7G/3_PSKR1_pskr1_H2O.jpg]

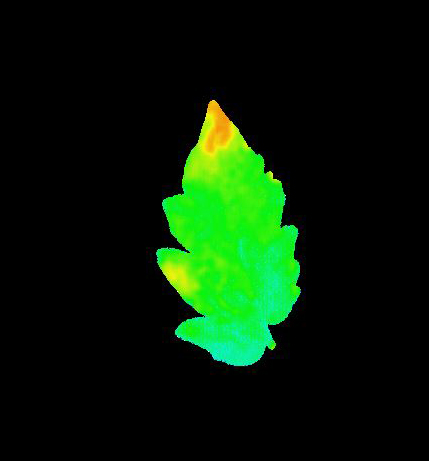

Supplement: Supplementary file 16 — Source data Fig. 7 [file 44318_2024_278_MOESM16_ESM.zip › Figure 7G/4_PSKR1_pskr1_PSK.jpg]

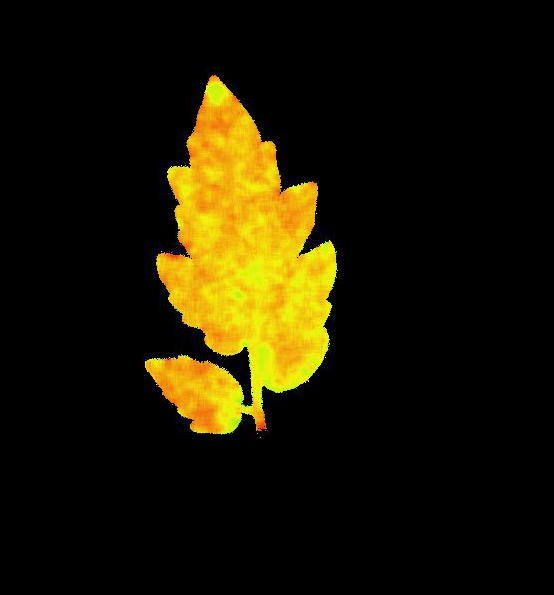

Supplement: Supplementary file 16 — Source data Fig. 7 [file 44318_2024_278_MOESM16_ESM.zip › Figure 7G/5_Y843F_pskr1_H2O.jpg]

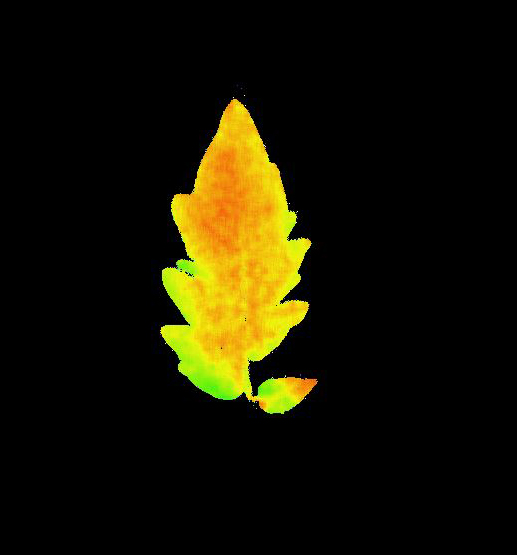

Supplement: Supplementary file 16 — Source data Fig. 7 [file 44318_2024_278_MOESM16_ESM.zip › Figure 7G/6_Y843F_pskr1_PSK.jpg]

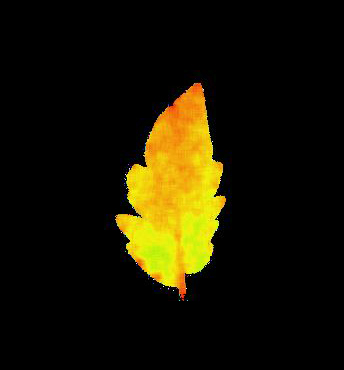

Supplement: Supplementary file 16 — Source data Fig. 7 [file 44318_2024_278_MOESM16_ESM.zip › Figure 7G/7_T890A_pskr1_H2O.jpg]

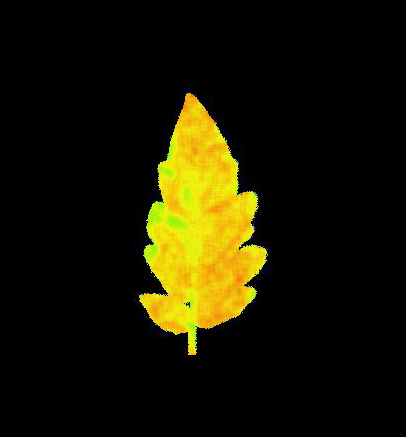

Supplement: Supplementary file 16 — Source data Fig. 7 [file 44318_2024_278_MOESM16_ESM.zip › Figure 7G/8_T890A_pskr1_PSK.jpg]

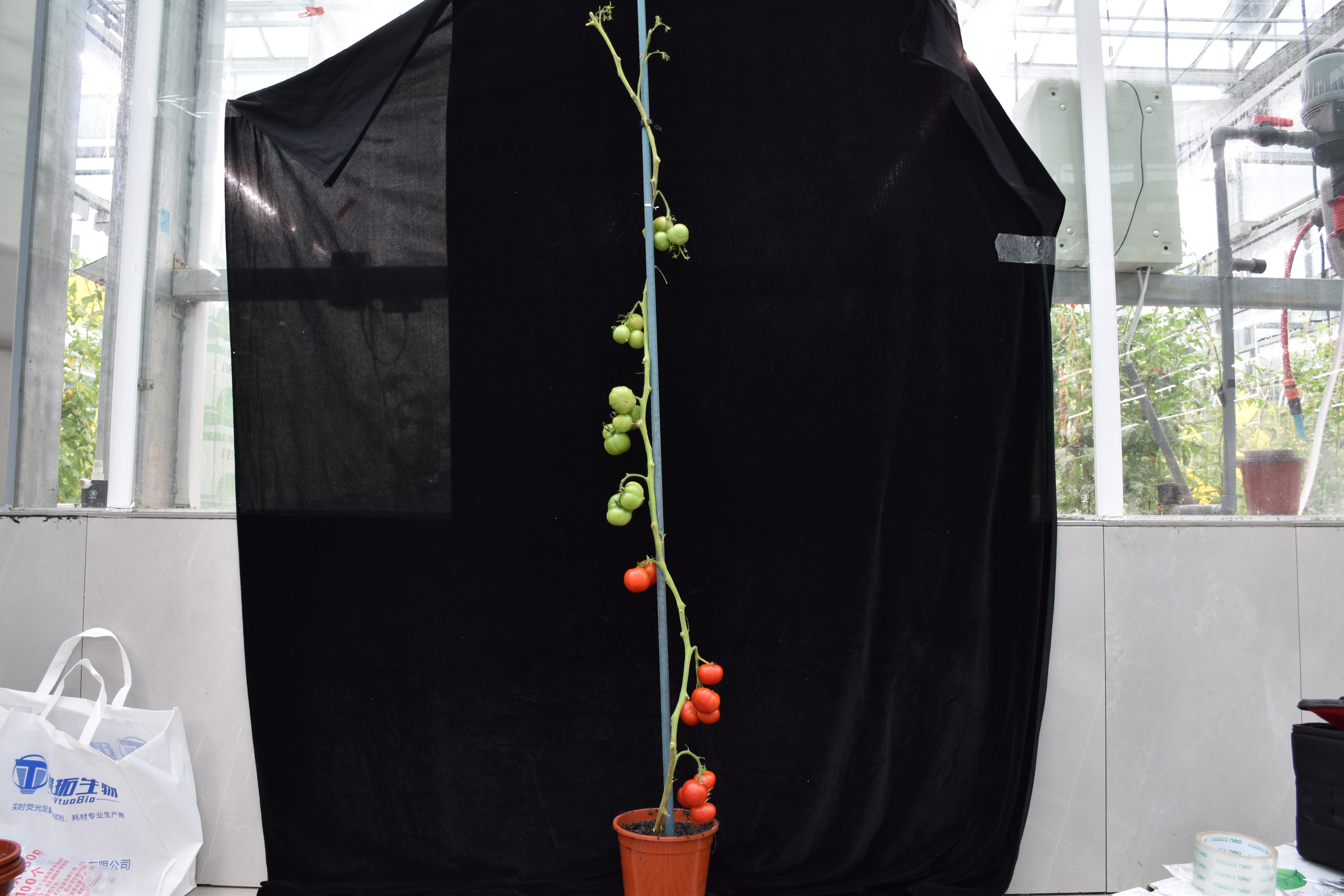

Supplement: Supplementary file 18 — Figure EV Source Data [file 44318_2024_278_MOESM18_ESM.zip › EMBOJ-2024-117048R_SourceDataForFigureEV1/Figure EV1A/1_WT.JPG]

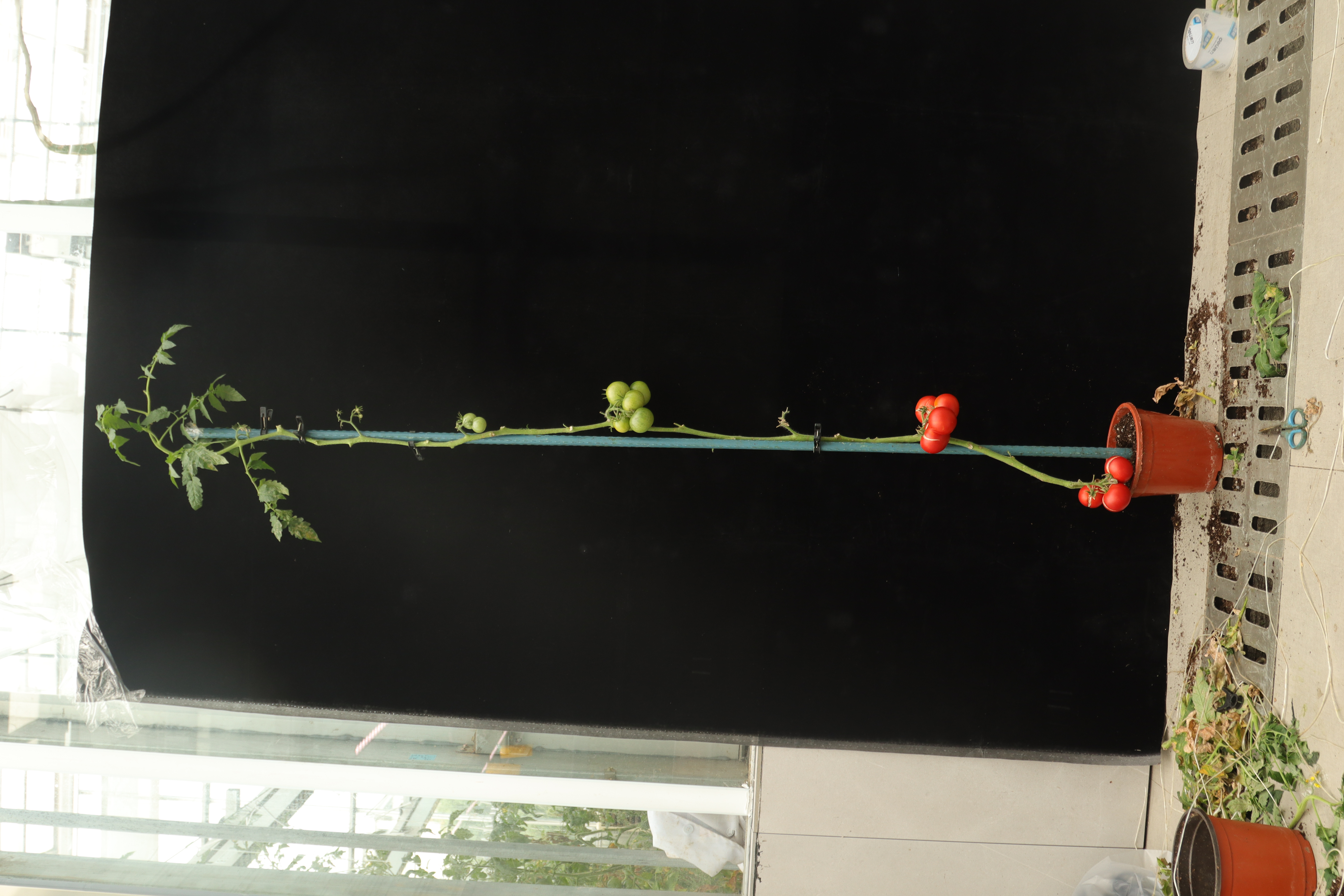

Supplement: Supplementary file 18 — Figure EV Source Data [file 44318_2024_278_MOESM18_ESM.zip › EMBOJ-2024-117048R_SourceDataForFigureEV1/Figure EV1A/2_pskr1#4.JPG]

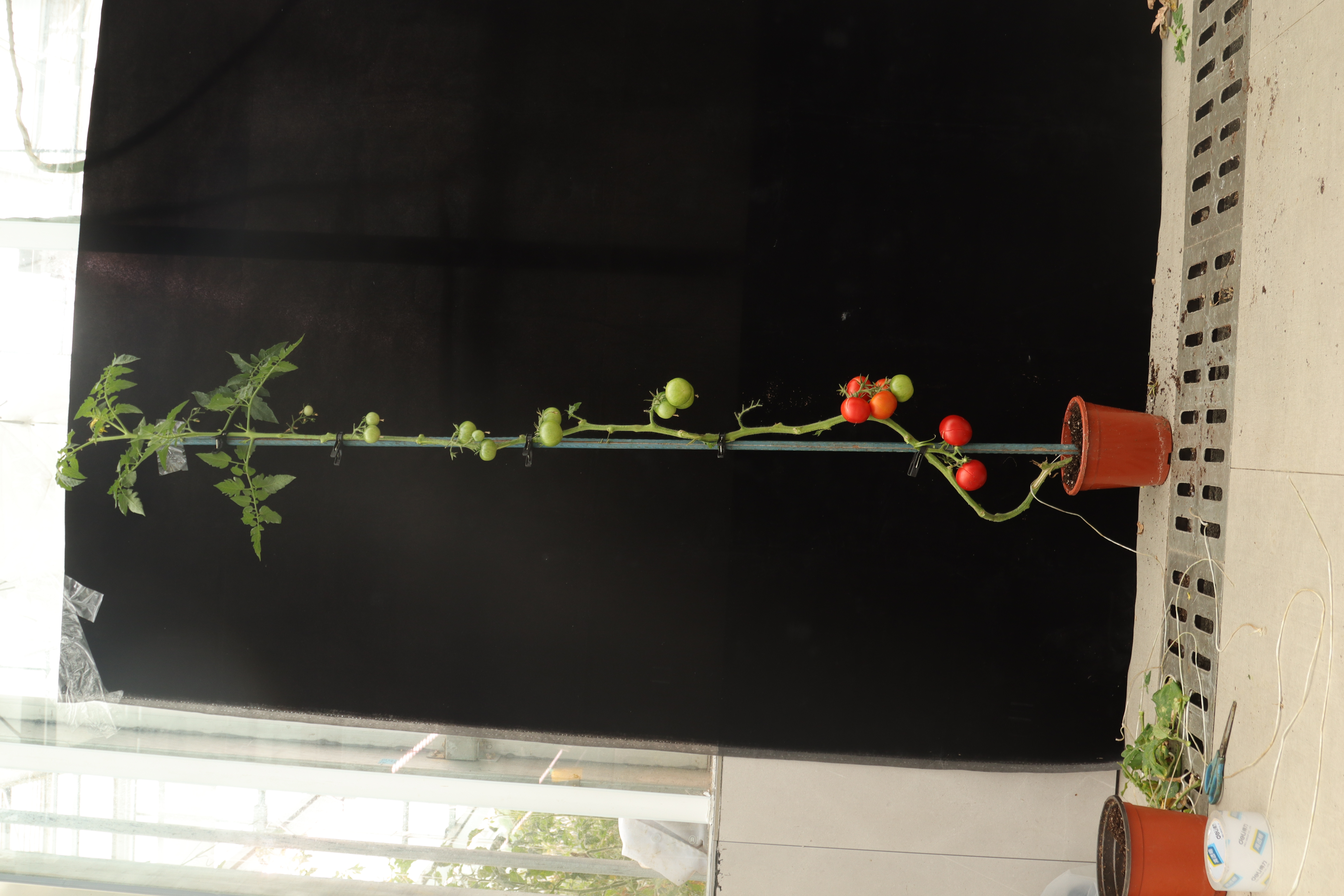

Supplement: Supplementary file 18 — Figure EV Source Data [file 44318_2024_278_MOESM18_ESM.zip › EMBOJ-2024-117048R_SourceDataForFigureEV1/Figure EV1A/3_pskr1#5.JPG]

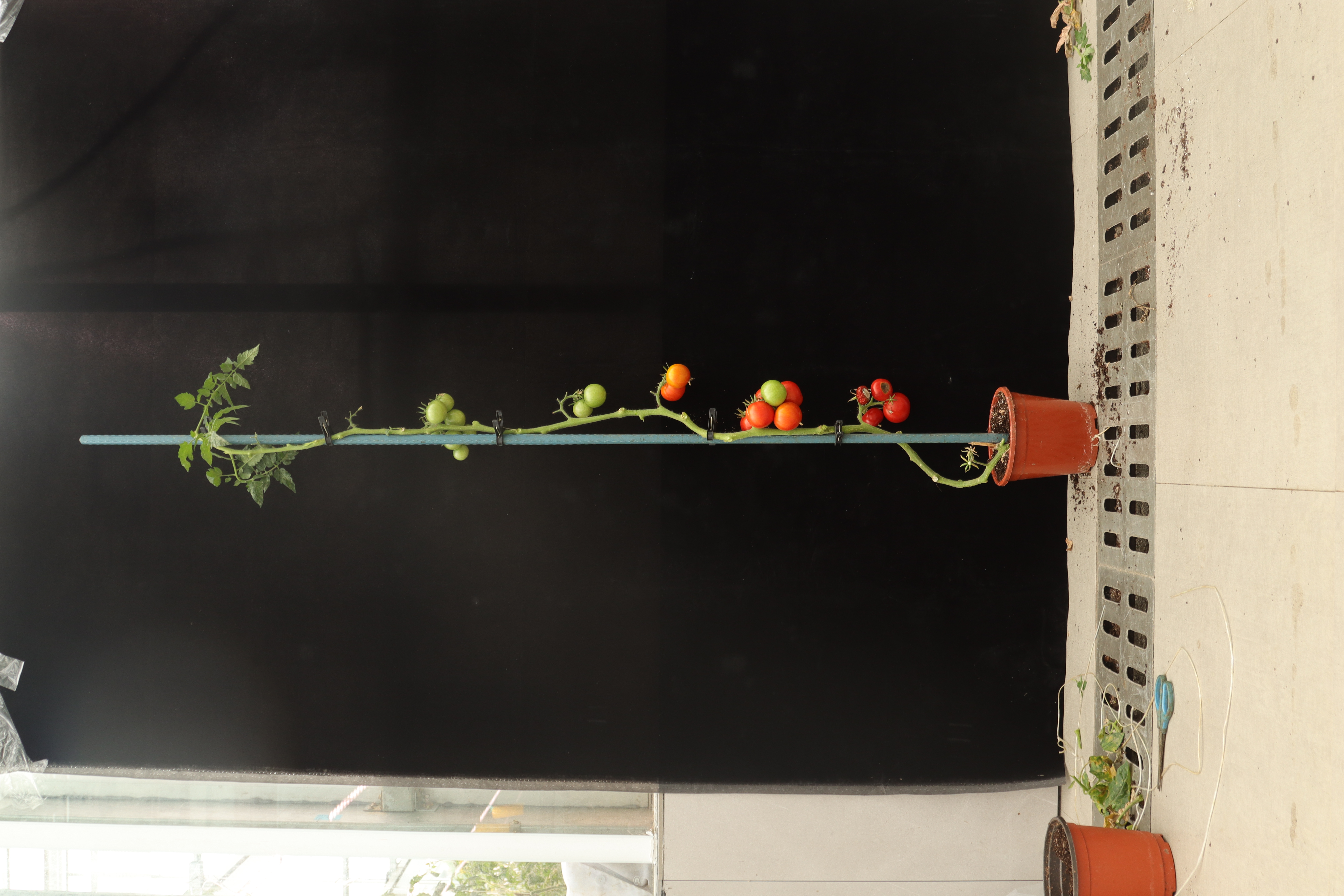

Supplement: Supplementary file 18 — Figure EV Source Data [file 44318_2024_278_MOESM18_ESM.zip › EMBOJ-2024-117048R_SourceDataForFigureEV1/Figure EV1A/4_OE-PSKR1#3.JPG]

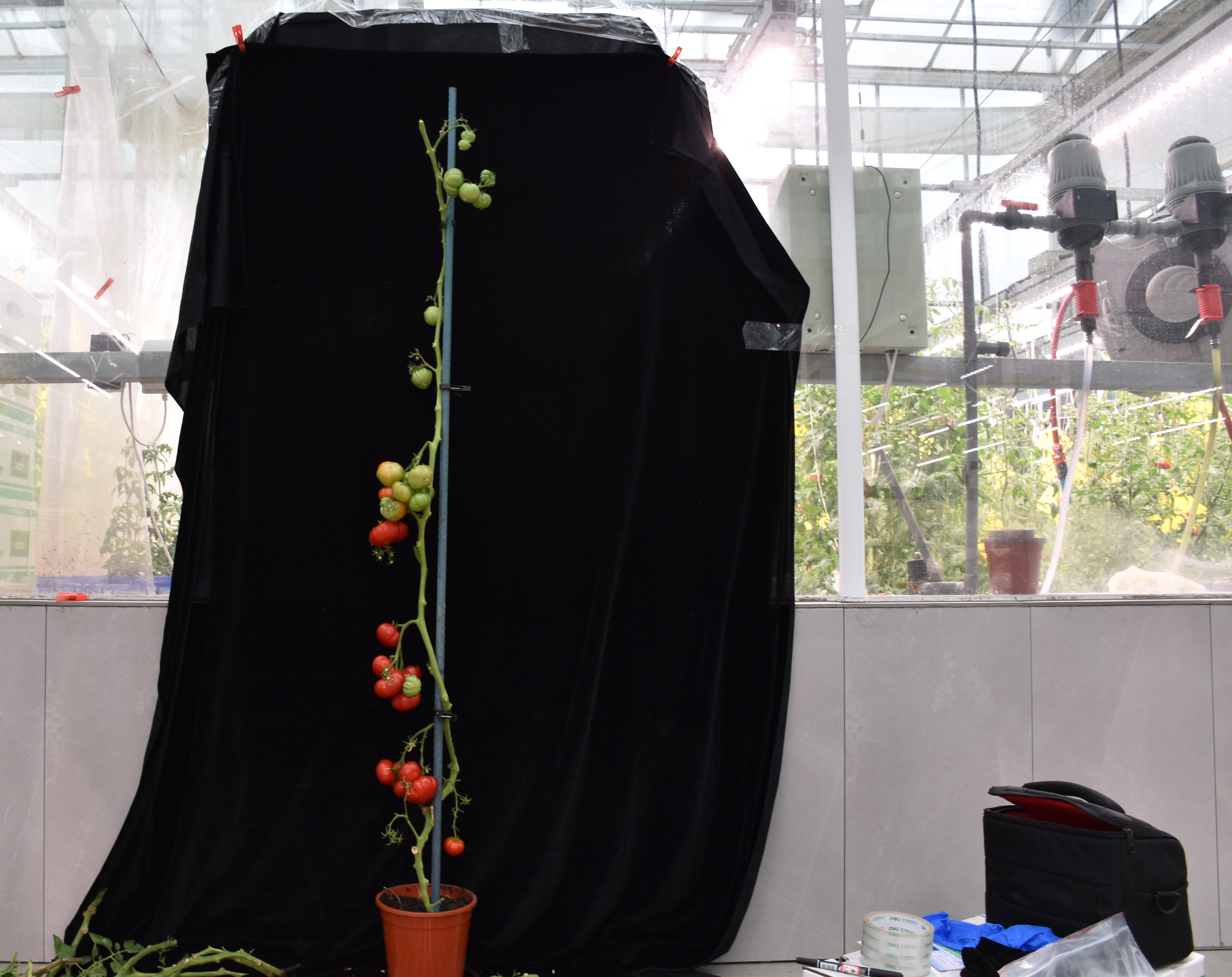

Supplement: Supplementary file 18 — Figure EV Source Data [file 44318_2024_278_MOESM18_ESM.zip › EMBOJ-2024-117048R_SourceDataForFigureEV1/Figure EV1A/5_OE_PSKR1#4.JPG]

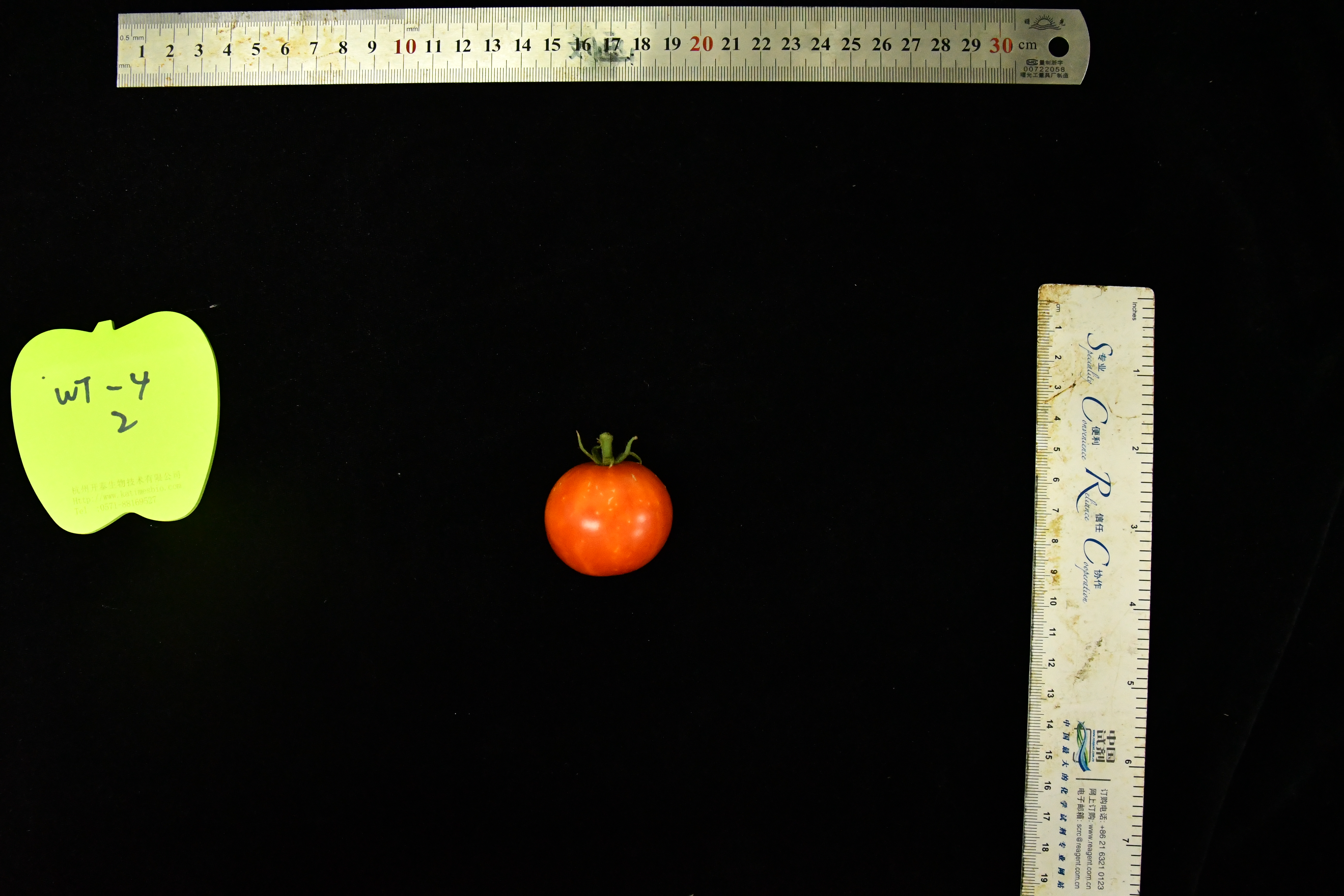

Supplement: Supplementary file 18 — Figure EV Source Data [file 44318_2024_278_MOESM18_ESM.zip › EMBOJ-2024-117048R_SourceDataForFigureEV1/Figure EV1B/1_WT.JPG]

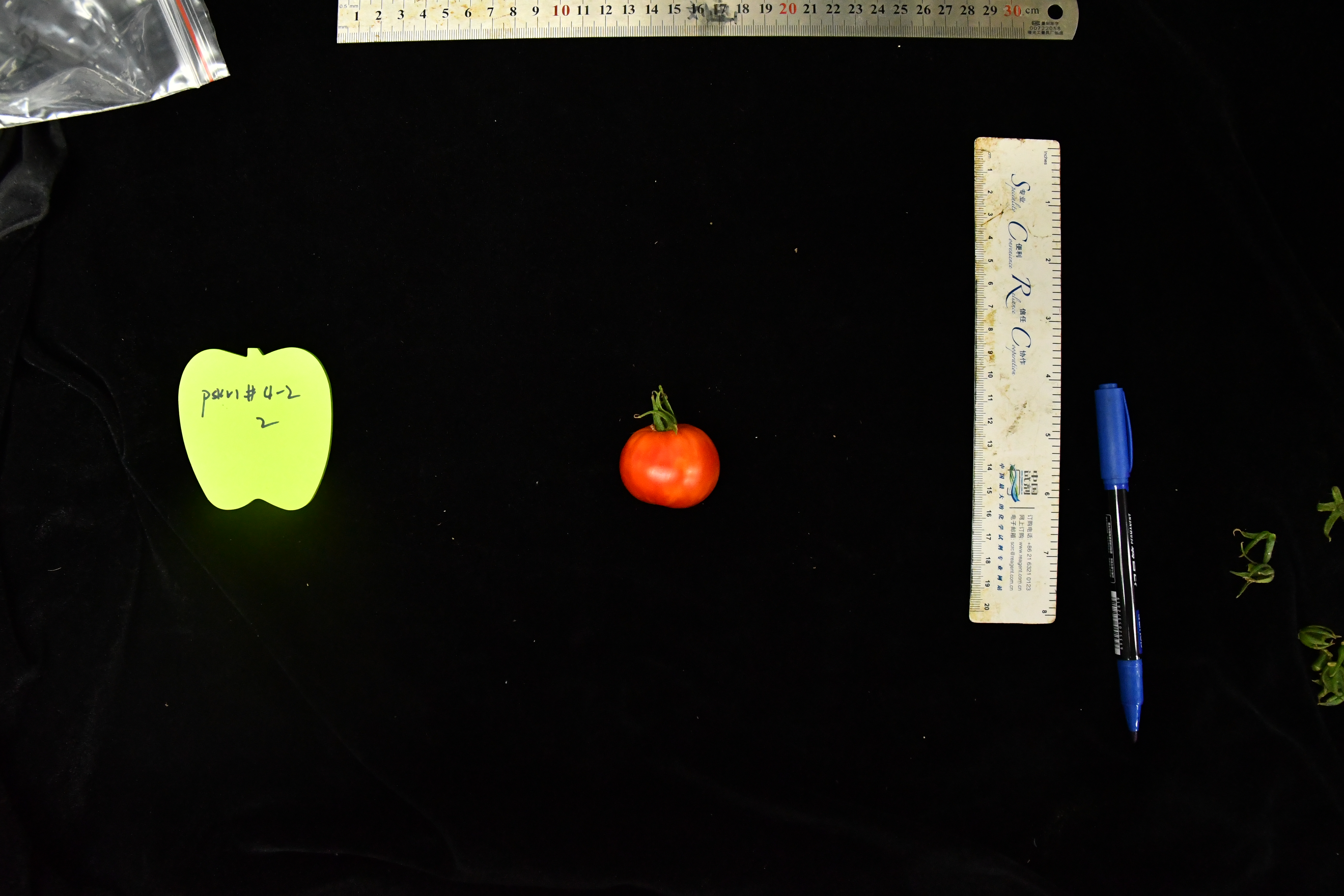

Supplement: Supplementary file 18 — Figure EV Source Data [file 44318_2024_278_MOESM18_ESM.zip › EMBOJ-2024-117048R_SourceDataForFigureEV1/Figure EV1B/2_pskr1#4.JPG]

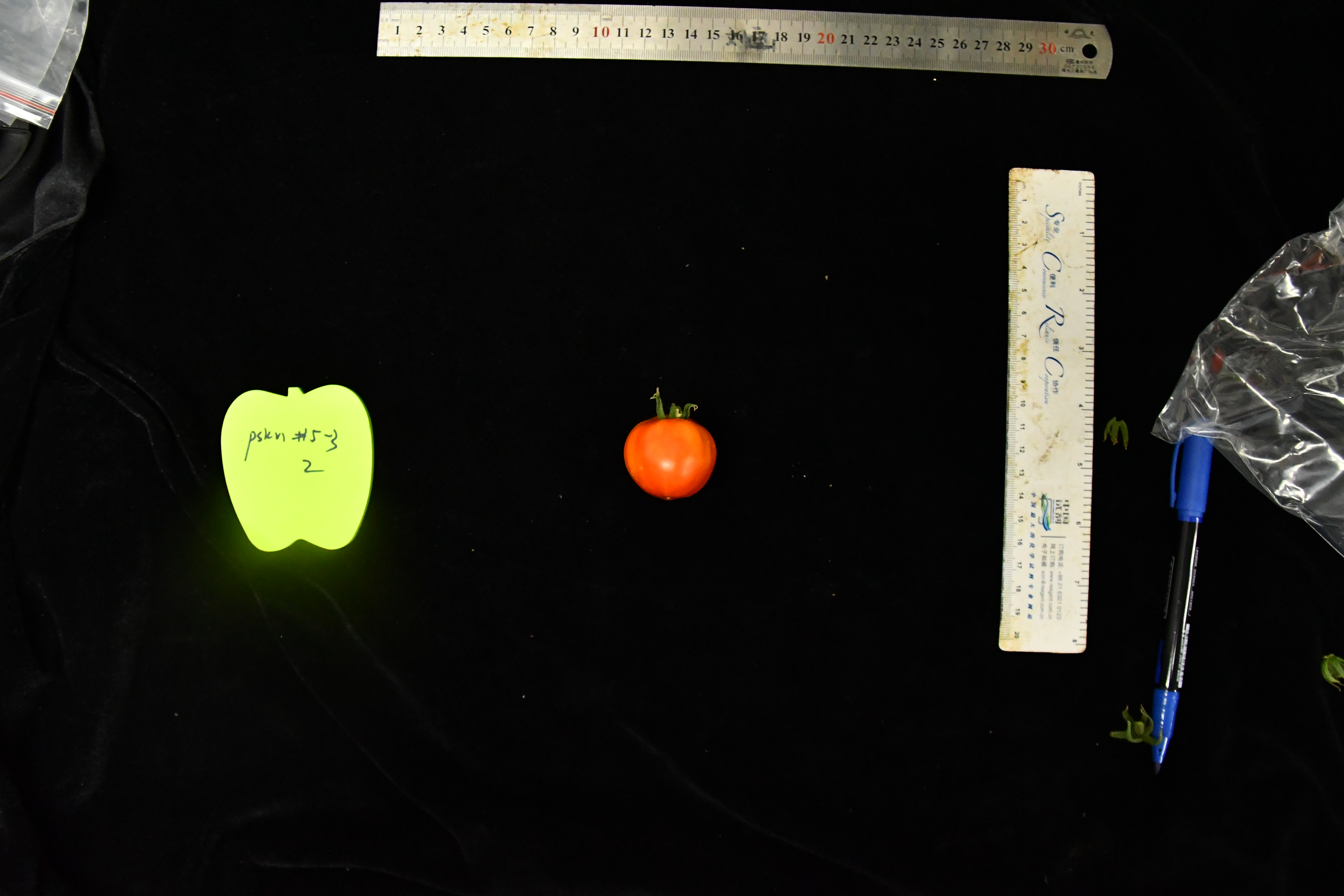

Supplement: Supplementary file 18 — Figure EV Source Data [file 44318_2024_278_MOESM18_ESM.zip › EMBOJ-2024-117048R_SourceDataForFigureEV1/Figure EV1B/3_pskr1#5.JPG]

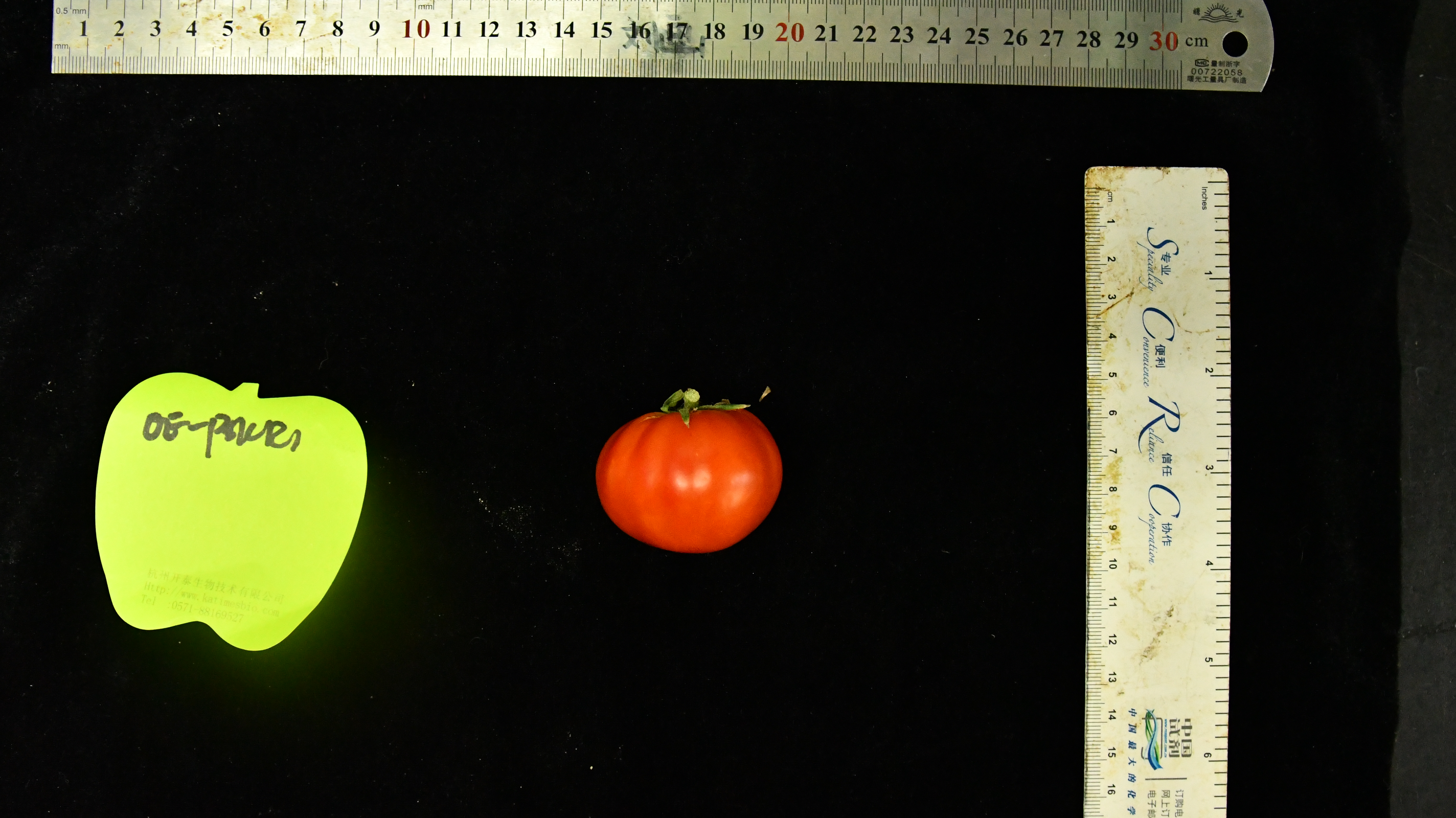

Supplement: Supplementary file 18 — Figure EV Source Data [file 44318_2024_278_MOESM18_ESM.zip › EMBOJ-2024-117048R_SourceDataForFigureEV1/Figure EV1B/4_OE_PSKR1#3.JPG]

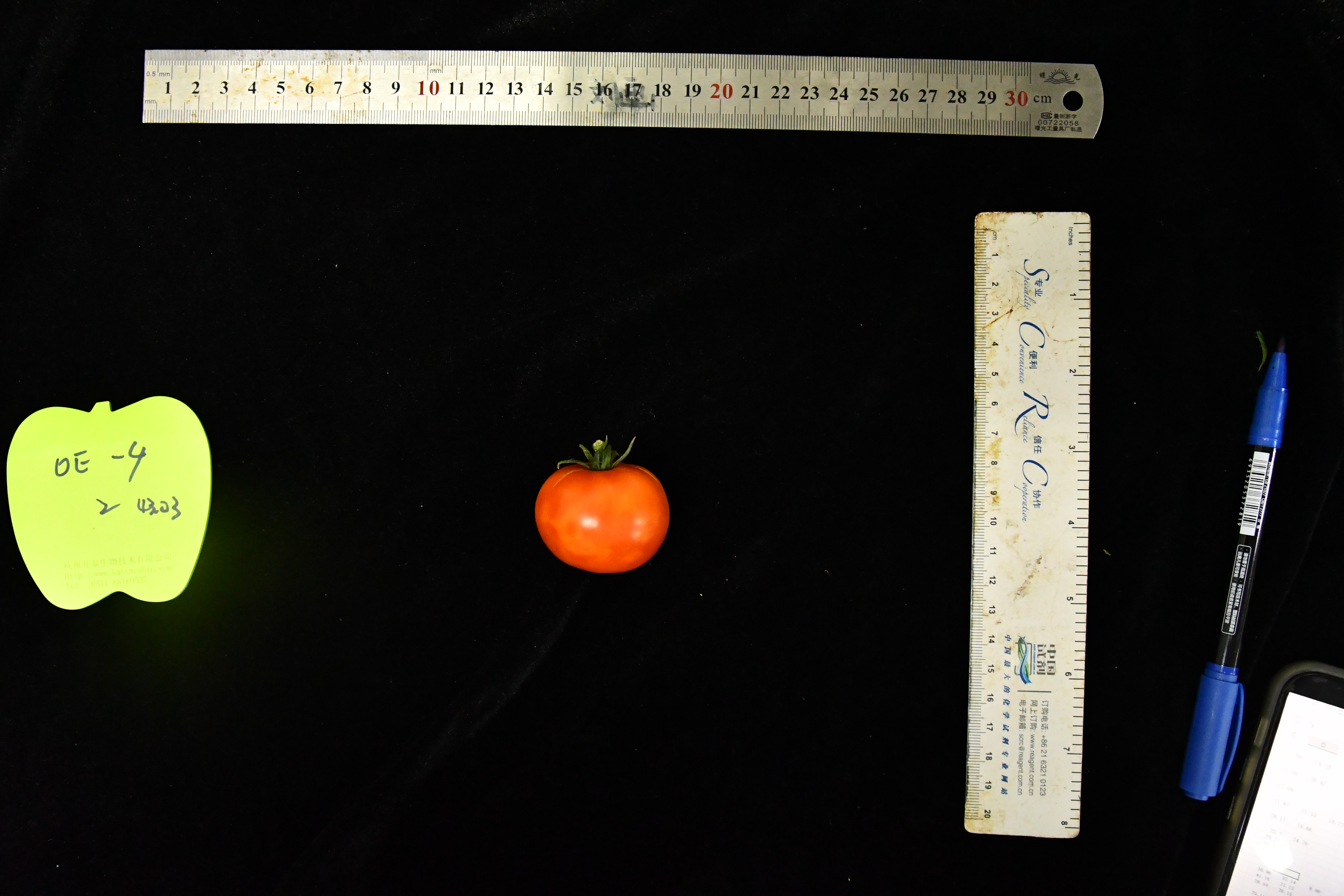

Supplement: Supplementary file 18 — Figure EV Source Data [file 44318_2024_278_MOESM18_ESM.zip › EMBOJ-2024-117048R_SourceDataForFigureEV1/Figure EV1B/5_OE_PSKR1#4.JPG]

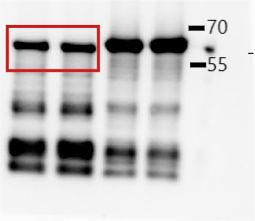

Supplement: Supplementary file 18 — Figure EV Source Data [file 44318_2024_278_MOESM18_ESM.zip › EMBOJ-2024-117048R_SourceDataForFigureEV2/Figure EV2B/anti- GST.jpg]

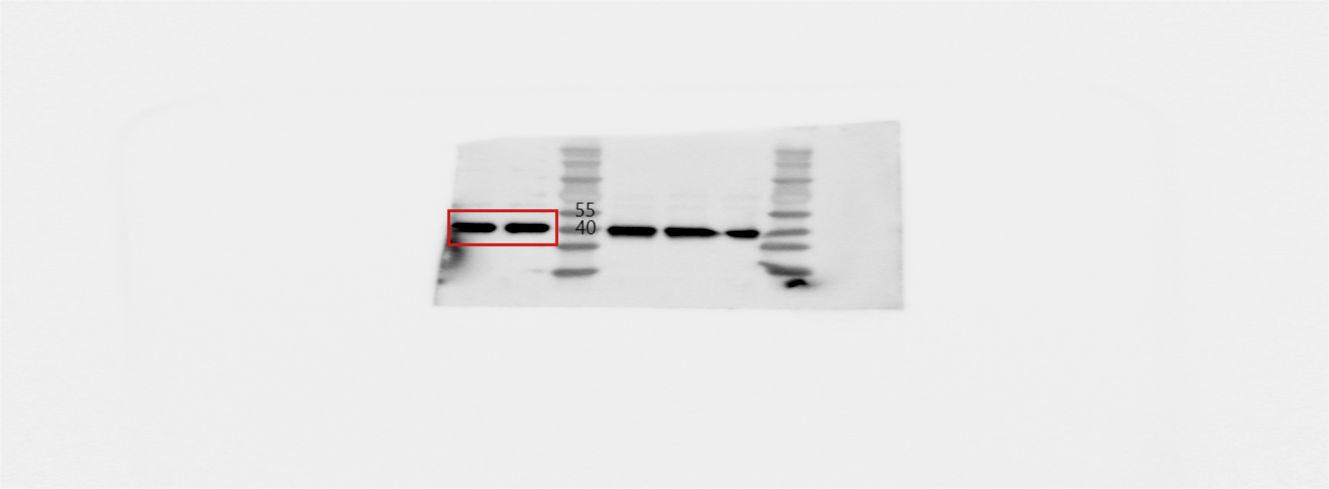

Supplement: Supplementary file 18 — Figure EV Source Data [file 44318_2024_278_MOESM18_ESM.zip › EMBOJ-2024-117048R_SourceDataForFigureEV2/Figure EV2B/anti-His.jpg]

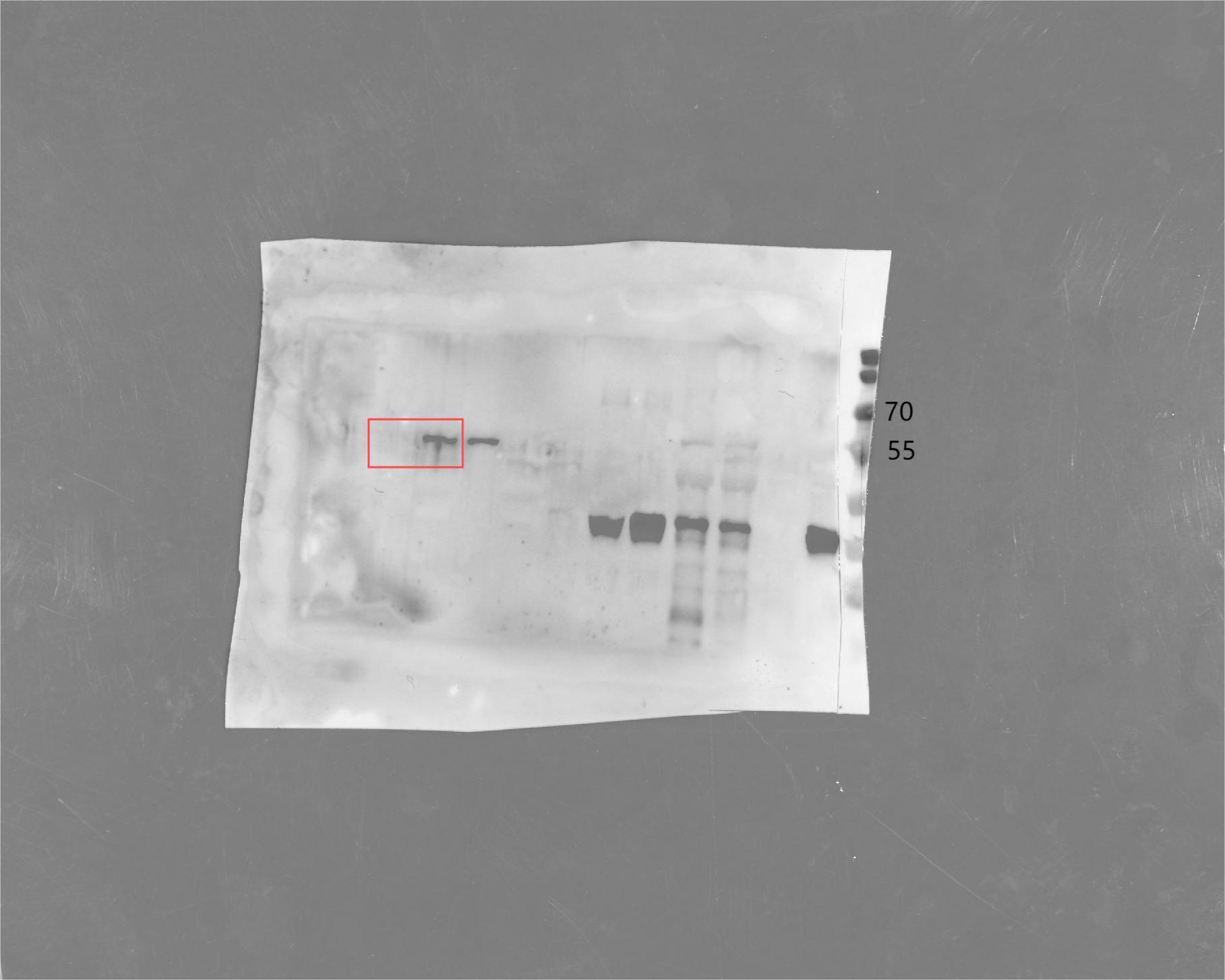

Supplement: Supplementary file 18 — Figure EV Source Data [file 44318_2024_278_MOESM18_ESM.zip › EMBOJ-2024-117048R_SourceDataForFigureEV2/Figure EV2B/anti-Thr Ser.jpg]

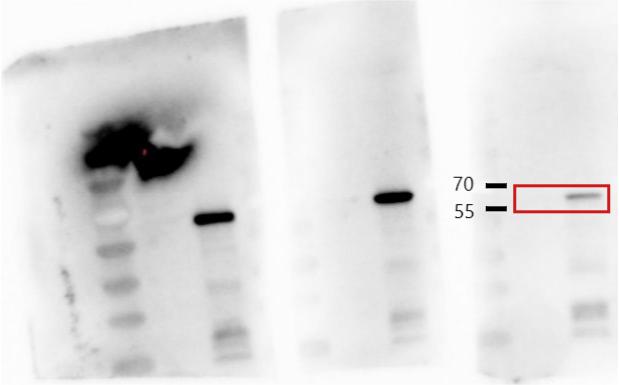

Supplement: Supplementary file 18 — Figure EV Source Data [file 44318_2024_278_MOESM18_ESM.zip › EMBOJ-2024-117048R_SourceDataForFigureEV2/Figure EV2B/anti-tyr.jpg]

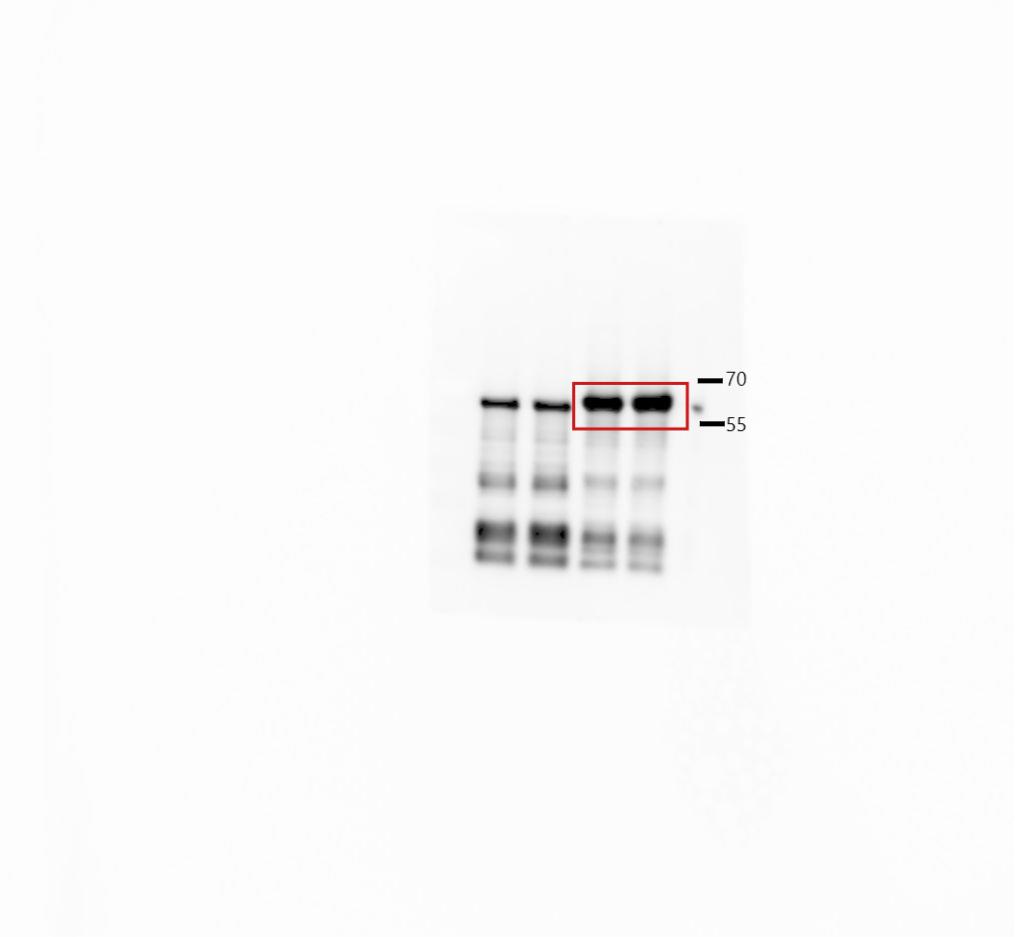

Supplement: Supplementary file 18 — Figure EV Source Data [file 44318_2024_278_MOESM18_ESM.zip › EMBOJ-2024-117048R_SourceDataForFigureEV2/Figure EV2C/anti- GST.jpg]

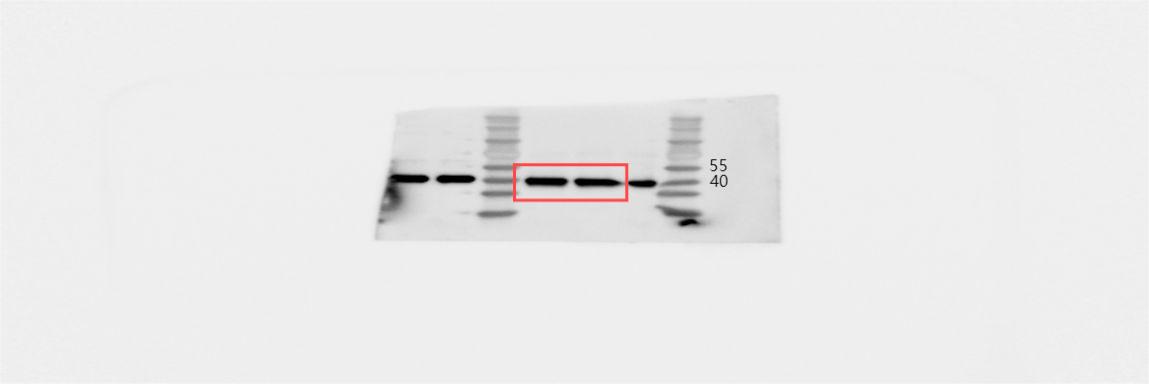

Supplement: Supplementary file 18 — Figure EV Source Data [file 44318_2024_278_MOESM18_ESM.zip › EMBOJ-2024-117048R_SourceDataForFigureEV2/Figure EV2C/anti- His.jpg]

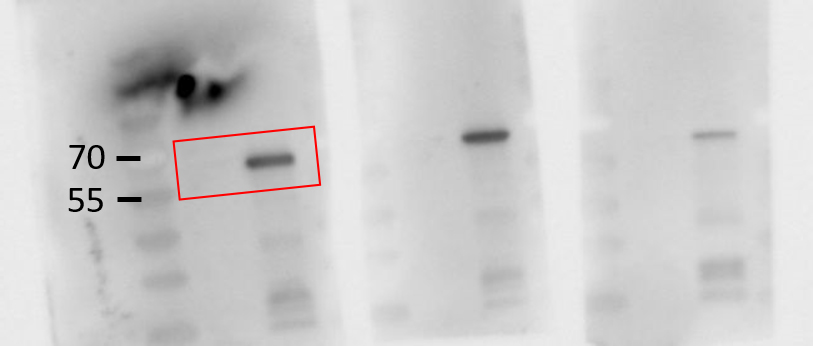

Supplement: Supplementary file 18 — Figure EV Source Data [file 44318_2024_278_MOESM18_ESM.zip › EMBOJ-2024-117048R_SourceDataForFigureEV2/Figure EV2C/anti-Thr Ser.jpg]

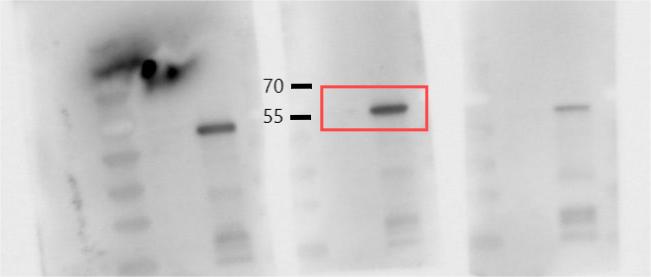

Supplement: Supplementary file 18 — Figure EV Source Data [file 44318_2024_278_MOESM18_ESM.zip › EMBOJ-2024-117048R_SourceDataForFigureEV2/Figure EV2C/anti-Tyr.jpg]

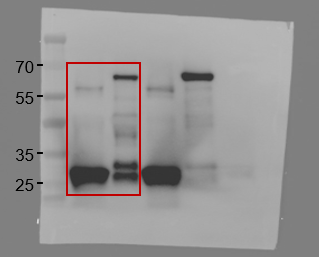

Supplement: Supplementary file 18 — Figure EV Source Data [file 44318_2024_278_MOESM18_ESM.zip › EMBOJ-2024-117048R_SourceDataForFigureEV2/Figure EV2D/anti-GST.tif]

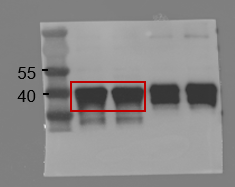

Supplement: Supplementary file 18 — Figure EV Source Data [file 44318_2024_278_MOESM18_ESM.zip › EMBOJ-2024-117048R_SourceDataForFigureEV2/Figure EV2D/anti-His.tif]

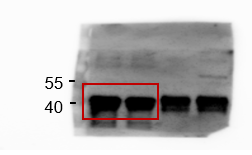

Supplement: Supplementary file 18 — Figure EV Source Data [file 44318_2024_278_MOESM18_ESM.zip › EMBOJ-2024-117048R_SourceDataForFigureEV2/Figure EV2D/anti-pSer-Thr.tif]

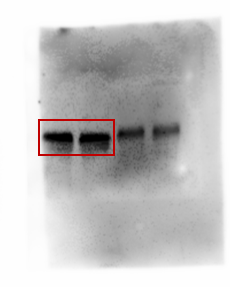

Supplement: Supplementary file 18 — Figure EV Source Data [file 44318_2024_278_MOESM18_ESM.zip › EMBOJ-2024-117048R_SourceDataForFigureEV2/Figure EV2D/anti-pTyr.tif]

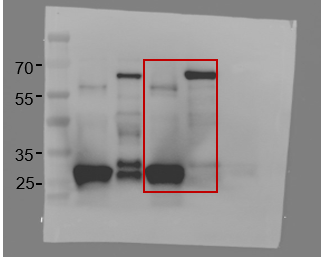

Supplement: Supplementary file 18 — Figure EV Source Data [file 44318_2024_278_MOESM18_ESM.zip › EMBOJ-2024-117048R_SourceDataForFigureEV2/Figure EV2E/anti-GST.tif]

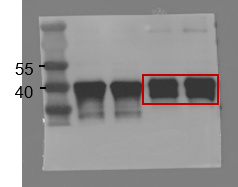

Supplement: Supplementary file 18 — Figure EV Source Data [file 44318_2024_278_MOESM18_ESM.zip › EMBOJ-2024-117048R_SourceDataForFigureEV2/Figure EV2E/anti-His.tif]

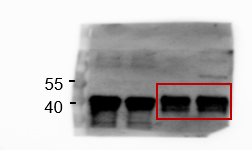

Supplement: Supplementary file 18 — Figure EV Source Data [file 44318_2024_278_MOESM18_ESM.zip › EMBOJ-2024-117048R_SourceDataForFigureEV2/Figure EV2E/anti-pSer-Thr.tif]

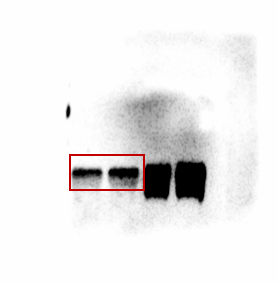

Supplement: Supplementary file 18 — Figure EV Source Data [file 44318_2024_278_MOESM18_ESM.zip › EMBOJ-2024-117048R_SourceDataForFigureEV2/Figure EV2E/anti-Tyr.tif]
