# Supplementary material for: A novel LRR receptor-like kinase BRAK reciprocally phosphorylates PSKR1 to enhance growth and defense in tomato
Source: EMBO J. 2024 Oct 24;43(23):16. doi: 10.1038/s44318-024-00278-z (PMC11612273; doi:10.1038/s44318-024-00278-z)
Supplement: Supplementary file 19 — Expanded View Figures [file 44318_2024_278_MOESM19_ESM.pdf]

Expanded View Figures

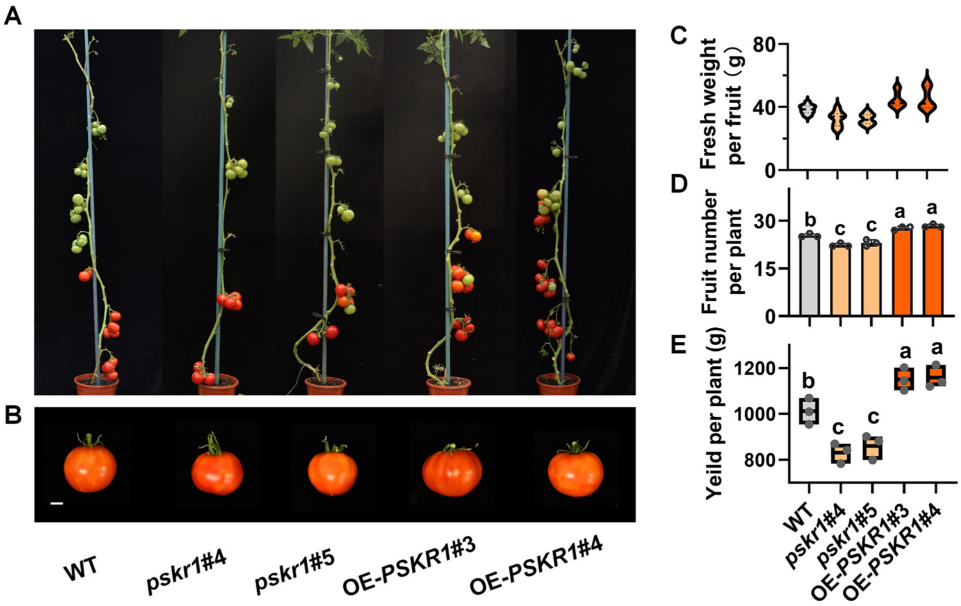

Figure EV1. The effect of PSKR1 on tomato fruit phenotype.

(**A**, **B**) Representative plant fruit phenotype in the reproductive stage. Bar, 1 cm (**B**). (**C–E**) Fresh weight per fruit (**C**), Fruit number per plant (**D**) and yield (**E**). Data in (**C–E**) are presented as mean values ± SD, $n = 10$ different tomato fruits in (**C**), $n = 3$ individual plants in (**D**), $n = 3$ individual plants in (**E**). For boxplots, the center line in the box indicates median, dots represent data, limits represent upper and lower quartiles. Different letters depict statistically significant differences, as analyzed by one-way ANOVA with Tukey's HSD post hoc test ($P < 0.05$). Plants were grown in the greenhouse. Source data are available online for this figure.

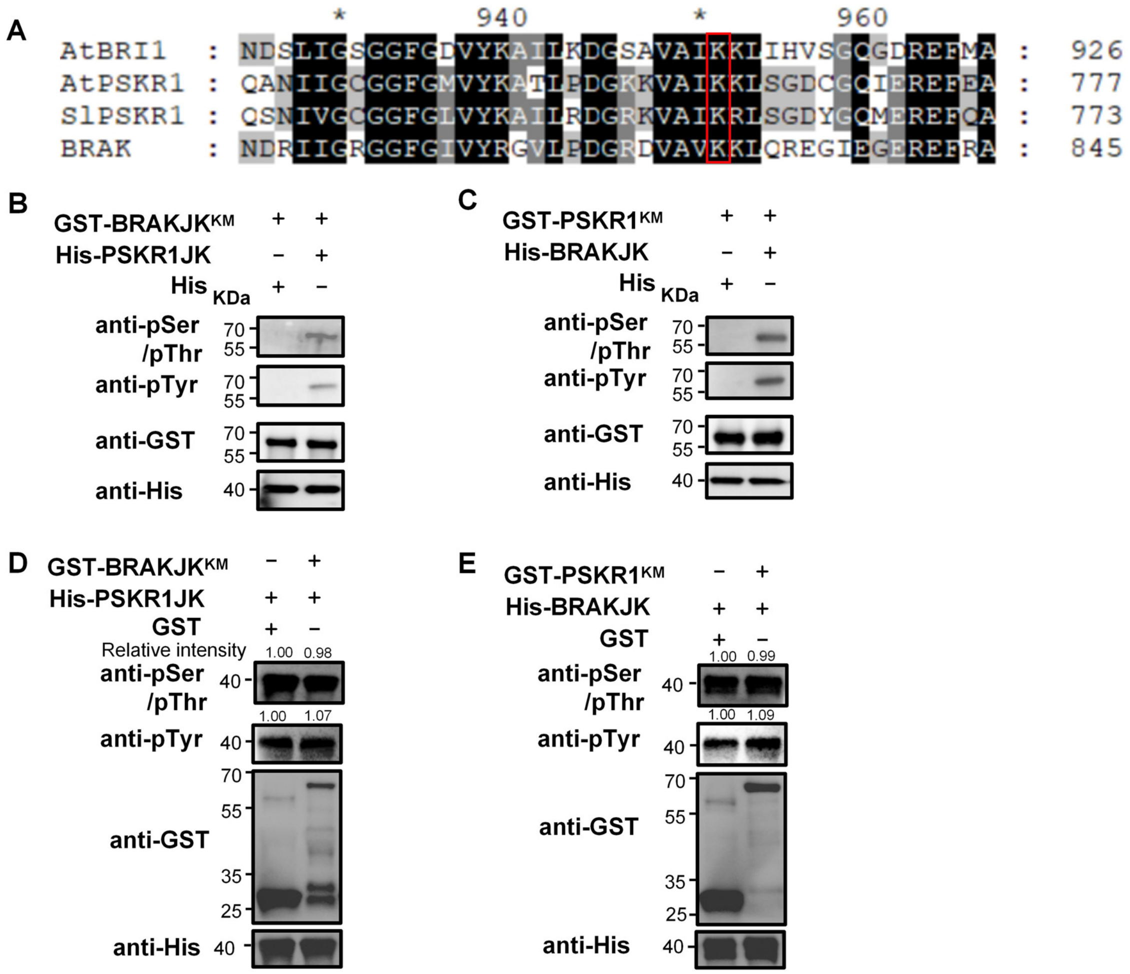

Figure EV2. BRAK and PSKR1 phosphorylate each other in vitro.

(**A**) Amino acid alignment among Arabidopsis BRI1, PSKR1, tomato PSKR1 and BRAK. The alignment was generated using Clustal X. Identical or similar amino acids are highlighted by black and gray backgrounds, respectively. The conserved lysine (K) in the ATP-binding site is boxed in red. (**B**) PSKR1 phosphorylate BRAK in vitro. The kinase assay was performed by incubating His or His-PSKR1JK as the kinase and GST-BRAKJK^{KM}. Phosphorylation is shown by western blot with anti-pSer/Thr and anti-pTyr antibodies. The protein loading control is shown by western blot with anti-GST and anti-His antibodies. (**C**) BRAK phosphorylate PSKR1 in vitro. The kinase assay was performed by incubating His or His-BRAKJK as the kinase and GST-PSKR1JK^{KM}. Phosphorylation is shown by western blot with anti-pSer/Thr and anti-pTyr antibodies. The protein loading control is shown by western blot with anti-GST and anti-His antibodies. (**D**) The effect of BRAK on the autophosphorylation of PSKR1 in vitro. The kinase assay was performed by incubating His-PSKR1JK and GST-BRAKJK^{KM}. Phosphorylation is shown by western blot with anti-pSer/Thr and anti-pTyr antibodies. The protein loading control is shown by western blot with anti-GST and anti-His antibodies. (**E**) The effect of PSKR1 on the autophosphorylation of BRAK in vitro. The kinase assay was performed by incubating His or His-BRAKJK as the kinase and GST-PSKR1JK^{KM}. Phosphorylation is shown by western blot with anti-pSer/Thr and anti-pTyr antibodies. The protein loading control is shown by western blot with anti-GST and anti-His antibodies. Source data are available online for this figure.

© The Author(s)
